# Solvent-controlled formation of alkali and alkali-earth-secured cucurbituril/guest trimers†

**DOI:** 10.1039/d3sc02032k

**Published:** 2023-08-15

**Authors:** Doroteja Lončarić, Fahimeh Movahedifar, Jakub Radek Štoček, Martin Dračínský, Josef Cvačka, Shanshan Guan, Benjamin J. Bythell, Ivana Císařová, Eric Masson, Jiří Kaleta

**Affiliations:** a Institute of Organic Chemistry and Biochemistry of the Czech Academy of Sciences Flemingovo nám. 2 160 00 Prague 6 Czech Republic jiri.kaleta@uochb.cas.cz; b Department of Organic Chemistry, Faculty of Science, Charles University in Prague 128 40 Prague 2 Czech Republic; c Department of Chemistry and Biochemistry, Ohio University Athens Ohio 45701 USA masson@ohio.edu; d Department of Inorganic Chemistry, Faculty of Science, Charles University in Prague 128 40 Prague 2 Czech Republic

## Abstract

Cucurbit[7]uril (CB[7]) encapsulates adamantyl and trimethylsilyl substituents of positively charged guests in dimethyl sulfoxide (DMSO). Unlike in water or deuterium oxide, addition of a selection of alkali and alkali-earth cations with van der Waals radii between 1.0 and 1.4 Å (Na^+^, K^+^, Ca^2+^, Sr^2+^, Ba^2+^ and Eu^3+^) to the CB[7]/guest complexes triggers their cation-mediated trimerization, a process that is very slow on the nuclear magnetic resonance (NMR) time scale. Smaller (Li^+^, Mg^2+^) or larger cations (Rb^+^, Cs^+^ or NH_4_^+^) are inert. The trimers display extensive CH–O interactions between the equatorial and pseudo-equatorial hydrogens of CB[7] and the carbonyl rim of the neighboring CB[7] unit in the trimer, and a deeply nested cation between the three interacting carbonylated CB[7] rims; a counteranion is likely perched in the shallow cavity formed by the three outer walls of CB[7] in the trimer. Remarkably, a guest must occupy the cavity of CB[7] for trimerization to take place. Using a combination of semi-empirical and density functional theory techniques in conjunction with continuum solvation models, we showed that trimerization is favored in DMSO, and not in water, because the penalty for the partial desolvation of three of the six CB[7] portals upon aggregation into a trimer is less unfavorable in DMSO compared to water.

## Abstract 2

Cucurbit[7]uril complexes aggregate into well-defined trimers in dimethyl sulfoxide in the presence of a selection of cations, as long as the host cavity is filled with a guest that leaves one carbonylated portal available for cation binding.

## Graphical abstract

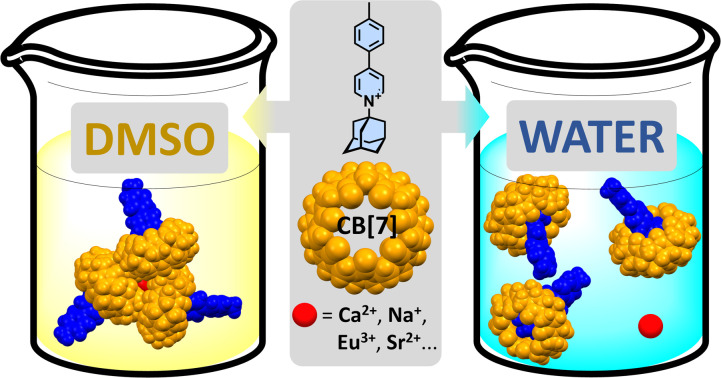

## Introduction

While the exquisite recognition properties of CB[*n*]s in water have been refined for the past 40 years,^1–11^ they have overshadowed exploration under non-aqueous conditions. To encapsulate very poorly water-soluble guests into CB[*n*]s, we recently showed that guests can be forced into their cavity by ball-milling a mixture of the host and guest in the solid state.^12^ Like Kaifer and co-workers before us,^13,14^ this brought us to also consider alternate, non-aqueous solvent systems that would allow encapsulation. Here we will show that CB[7] not only forms tight inclusion complexes with *N*-adamantyl- and *N*-trimethylsilylmethyl-pyridinium in DMSO-*d*_6_, but also undergoes quantitative complex trimerization in the presence of an exclusive selection of alkali and alkali-earth cations, as long as a guest occupies the cavity of CB[7]. We will present an in-depth justification for this new cluster formation.

## Results and discussion

**Fig. 1:**
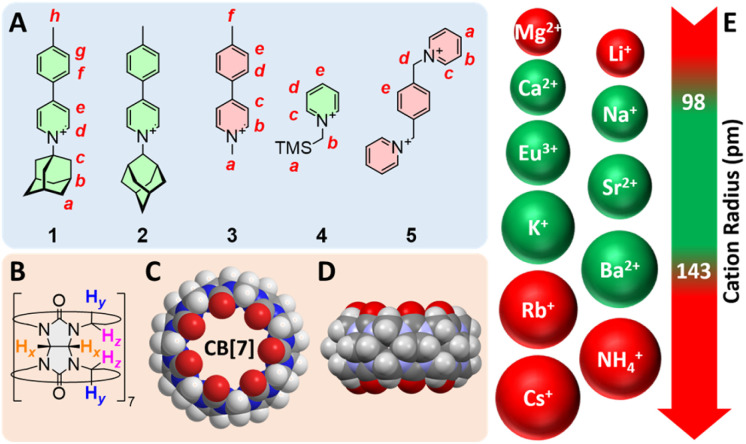
Structures of (A) guests 1–5 and (B) CB[7]. (C) Top and (D) side views of the X-ray crystal structure of CB[7].^15^ (E) List of evaluated cations with their atomic radii;^16,17^ green-colored structures afford trimeric assemblies.

**Fig. 2:**
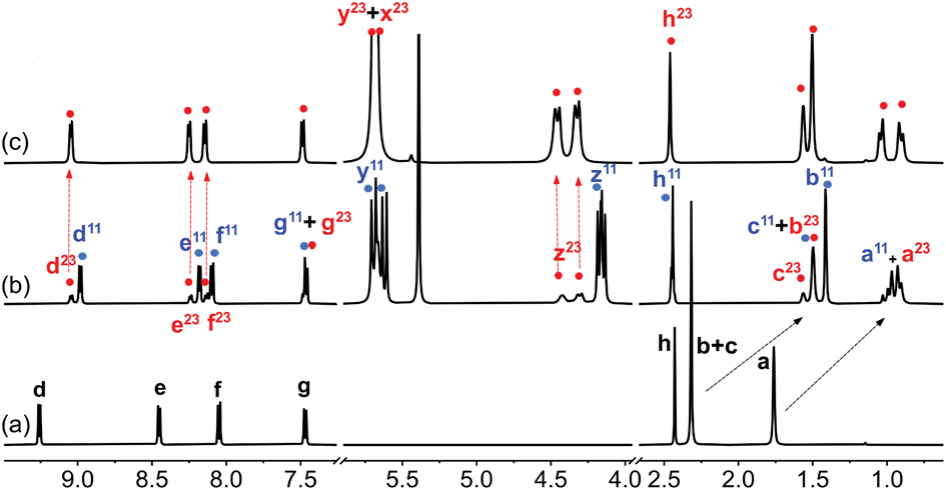
^1^H NMR spectrum of (a) guest 1 in DMSO-*d*_6_ (1.0 mM), (b) after addition of 1.0 equiv. CB[7], and (c) after subsequent addition of 2.3 equiv. NaCl. See Fig. 1 for hydrogen nuclei labeling. “11” and “23” exponents refer, respectively, to binary complex CB[7]·1 and to a new assembly discussed thereafter. Chemical shifts in ppm.

Compounds 1–5 were synthesized in up to three steps (see ESI† section for details) and used as our model guests (Fig. 1). Structures 1 and 2 are rigid rods, with the 1- or 2-adamantyl units being intended as CB[7] binding sites. Guest 3 is a control that lacks the binding site. We anticipated CB[7] would encapsulate the trimethylsilyl and xylylene units of guests 4 and 5, respectively. As CB[7] encapsulates the adamantyl unit of guest 1, the combination of pyridinium and tolyl units allows the monitoring of the recognition process by ^1^H nuclear magnetic resonance spectroscopy (NMR) over multiple signals at various chemical shifts (Fig. 2). Complex CB[7]·1 was readily formed in DMSO, with a solubility reaching 2.0 mM at 25 °C (the solubility of free CB[7] is 0.70 mM), as determined by ^1^H NMR spectroscopy in the presence of *N*,*N*-dimethylformamide used as an internal, inert standard present at a known concentration.

Adamantyl signals H^a^ and H^b^ underwent upfield shifts upon encapsulation (up to 0.83 ppm, see signals with the “11” label and highlighted in blue in Fig. 2) as expected. Pyridinium hydrogens H^d^ also underwent upfield shifts (0.28 ppm), while phenyl hydrogens H^f^ and H^g^, and methyl nuclei H^h^ barely shifted (up to 0.05 ppm downfield for hydrogens H^f^). An overall binding affinity of 1.0 (±0.1) × 10^4^ M^−1^ was determined by UV-Vis spectroscopy for guest 1 towards CB[7] in DMSO using a 1 : 1 binding model (see ESI† section for details). Surprisingly, however, a second assembly also formed in a 12 : 88 ratio together with complex CB[7]·1 (see signals with the “23” label and highlighted with red dots in Fig. 2). The appearance of broader signals at 4.31 and 4.43 ppm corresponding to the pseudo-equatorial^12^ methylene hydrogens of the CB[7] portals, downfield by up to 0.27 ppm compared to the same hydrogens in complex CB[7]·1 and 0.31 ppm compared to free CB[7], was particularly perplexing (see signals labeled “z” in red and blue respectively). In contrast, guest encapsulation in deuterium oxide affords complex CB[7]·1 quantitatively, and addition of at least 60 vol% D_2_O to the DMSO solution annihilates the minor assembly. Recording the ^1^H NMR spectrum of the DMSO sample at 100 °C did not result in any coalescence between both assemblies, thereby indicating a very slow (if at all present) exchange process.

To make it even more enigmatic, only guests 1, 2 and 4 afforded this assembly, although all five structures 1–5 were clearly complexed with CB[7] in DMSO. The fact that these three guests are forming the same type of complex (the binding site is encapsulated inside CB[7] while the rest of their structure sticks out of only one portal) implies that some specific steric requirements must be fulfilled to access this mysterious species.

Inspired by the work of Bardelang and coworkers,^18–22^ we then suspected that aggregation might take place and result in the formation of larger clusters of complex CB[7]·1 in DMSO. Bardelang and coworkers showed that in the presence of sodium cations (≥5.0 mM), CB[8]-bound nitroxide radicals undergo trimerization in D_2_O; they also observed a small amount of aggregation in the absence of added sodium, and attribute it to the trimer without its sodium core cation.^19^

While no aggregation is observed with guest 1 and CB[7] in D_2_O even in the presence of sodium chloride, addition of sodium cation to a DMSO solution of complex CB[7]·1 triggered its conversion to the other unknown assembly; quantitative conversion was observed in the presence of 2.3 mM Na^+^. Again, addition of at least 60% D_2_O quantitatively restored complex CB[7]·1. Diffusion-ordered spectroscopy (DOSY) of a mixture of both assemblies afforded diffusion coefficients *D* of 9.7 (±0.1) × 10^−11^ and 6.9 (±0.1) × 10^−11^ m^2^ s^−1^ for complex CB[7]·1 and the unknown assembly, respectively (log *D* −10.02 (±0.01) and −10.16 (±0.01), respectively). Power law (1), derived from the Stokes–Einstein equation with *M* being the molecular weight of the analyte,^23–25^ can be rearranged into eqn (2) and used to approximate the molecular weight of the unknown assembly relative to that of complex CB[7]·1 (Δ(log *M*); *m* ranges from approximately 1/3 for spherical structures to 0.6 for linear polymers).^23^

1 *D* ∝ *M*^−*m*^

2 Δ(log *D*) = −*m* × Δ(log *M*)

with *m* = 1/3, eqn (2) returns Δ(log *M*) equal to 0.45, which corresponds to a 2.8-fold increase in the molecular weight for the unknown assembly compared to complex CB[7]·1, in remarkably accurate agreement with a possible trimerization process.

The presence of small amounts of the possible trimer in the absence of added sodium cations remained perplexing and lead us to suspect a possible contamination of CB[7] sources with sodium cations, a very common occurrence whenever glassware is used. To test this hypothesis, we prepared a series of CB[7] solutions in water at known concentrations (CB[7] synthesized in our laboratories using known procedures^26^ and commercial sources), and determined their sodium content by inductively coupled plasma optical emission spectrometry (ICP-OES). All solutions returned a sodium content of *ca.* 0.065% which corresponds to one Na^+^ per *ca.* 30–35 CB[7] units. All efforts to remove sodium by dialysis failed to decrease sodium content; whether the latter might in fact originate from glassware etching during the preparation and storage of CB[7] remains an open question. To the best of our knowledge, unlike hydrochloric acid and water contaminations, sodium contamination of CB[*n*] batches has not been reported before. This is easily understandable as those cations typically do not significantly affect recognition processes in aqueous solution.

**Fig. 3:**
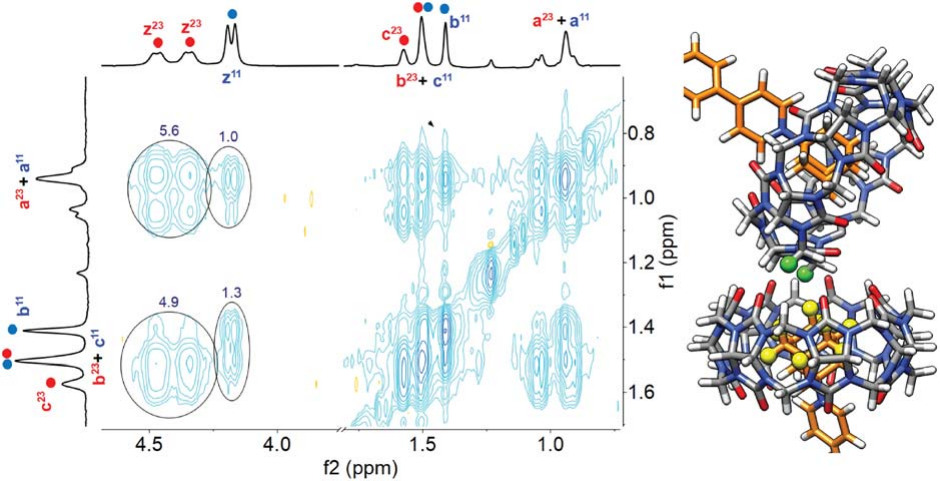
^1^H–^1^H NOESY spectrum of a 1 : 1 mixture of complex CB[7]·1 and putative aggregate (CB[7]·1)_3_Na. The correlations between adamantyl signals H^a^ and H^b^ with equatorial methylenes H^z^ at the CB[7] portals are highlighted. Section of an optimized structure of putative complex (CB[7]·1)_3_Na, with interacting hydrogens H^z^ highlighted in green, and H^a^ and H^b^ in yellow.

Aggregation of complex CB[7]·1 in the presence of sodium cations in DMSO was also confirmed by nuclear Overhauser effect spectroscopy (NOESY) using a sample containing a mixture of complex CB[7]·1 and its unknown parent. Adjusted for their respective concentrations, 5.6- and 3.8-fold increases were observed in the volume integrations of the cross-peaks between the pseudoequatorial methylene hydrogens H^z^ at the CB[7] portals and adamantyl hydrogens H^a^ and H^b^, respectively, in the unknown assembly compared to complex CB[7]·1 (see Fig. 3, contacts are highlighted in green and yellow). Enhanced crosspeaks are also observed with pseudoaxial methylene hydrogens H^y^ (see Fig. S71†). They highlight the proximity of the adamantyl hydrogens to the hydrogens of a neighboring CB[7] unit in the cluster.

**Fig. 4:**
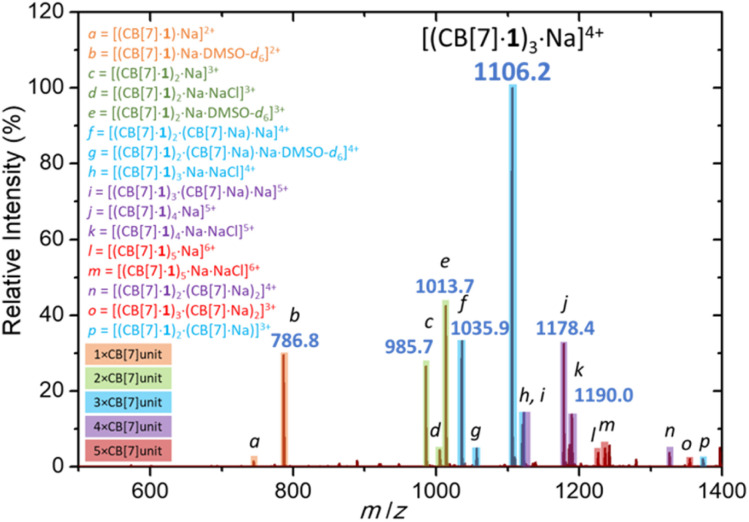
Electrospray ionization mass spectrum of a DMSO-*d*_6_ solution of [(CB[7]·1)_3_Na]Cl_4_ recorded in a positive mode.

Finally, sodium cation-promoted trimerization was confirmed by electrospray ionization mass spectrometry (Fig. 4). The most abundant signal corresponds to trimer [(CB[7]·1)_3_·Na]^4+^ (*m*/*z* 1106.2). The trimeric species were detected also as [(CB[7]·1)_2_·(CB[7]·Na)·Na]^4+^ (*m*/*z* 1035.9), [(CB[7]·1)_3_·Na·NaCl]^4+^ (*m*/*z* 1120.2) and [(CB[7]·1)_2_·(CB[7]·Na)·Na·DMSO-*d*_6_]^4+^ (*m*/*z* 1056.4). Other oligomeric species, such as [(CB[7]·1)_2_·Na]^3+^ (*m*/*z* 985.7), [(CB[7]·1)_4_·Na]^5+^ (*m*/*z* 1178.4), [(CB[7]·1)_5_·Na]^6+^ (*m*/*z* 1226.0) and monomer [(CB[7]·1)·Na]^2+^ (*m*/*z* 744.8) were significantly less abundant. We note the absence of coordinating DMSO in the trimer and higher oligomers, as the CB[7] portals fully occupy the first solvation shell of the sodium cation.

The collision-induced MS^*n*^ dissociation analysis of the parent ion [(CB[7]·1)_3_·Na]^4+^ also suggested high stability in the gas phase. The fragmentation pathway consists of three subsequent losses of the guest molecules 1, that were sequentially expelled from the CB[7] cavity ([(CB[7]·1)_3_·Na]^4+^ → [(CB[7]·1)_2_·CB[7]·Na]^3+^ → [(CB[7]·1)·CB[7]_2_·Na]^2+^ → [CB[7]_3_·Na]^+^). Remarkably, the trimeric cluster remained intact, and each following fragmentation required higher collision energies (Fig. S78–S83†).

Trimerization was observed with the chloride salts of sodium, potassium, calcium, strontium, and europium, but not lithium, rubidium, cesium, magnesium, cerium or ammonium; it was also observed with sodium dodecamethylcarba-*closo*-dodecaborate (NaCB_11_(CH_3_)_12_) and sodium 2,3,4,5,6,7,8,9,10,11-undecamethylcarba-*closo*-dodecaborate (NaHCB_11_(CH_3_)_11_), which have the unique advantage of allowing direct concentration determination of sodium by ^1^H NMR spectroscopy using the carborane hydrogen signals. Addition of calcium nitrate, calcium triflate and barium nitrate also afforded trimerization – the role of the anion will be discussed later. We also note that the sodium-secured trimer remains intact in the presence of an excess amount of 15-crown-5 (*i.e.* the latter does not compete with CB[7] for sodium binding). Similarly, adding a 5 : 1 mixture of 15-crown-5 and NaCl to complex CB[7]·1 affords the trimer.

To understand the mechanism of the trimerization, we first considered equilibrium (3) and its corresponding equilibrium constant *β*_13_ (with M being the added metal cation, see eqn (4)):

3 3(CB[7]·1) + M ⇄ (CB[7]·1)_3_M

4
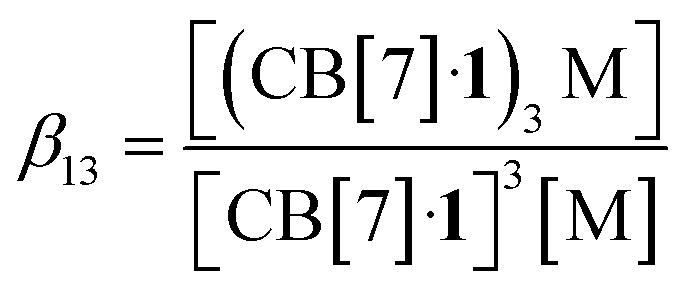


**Fig. 5:**
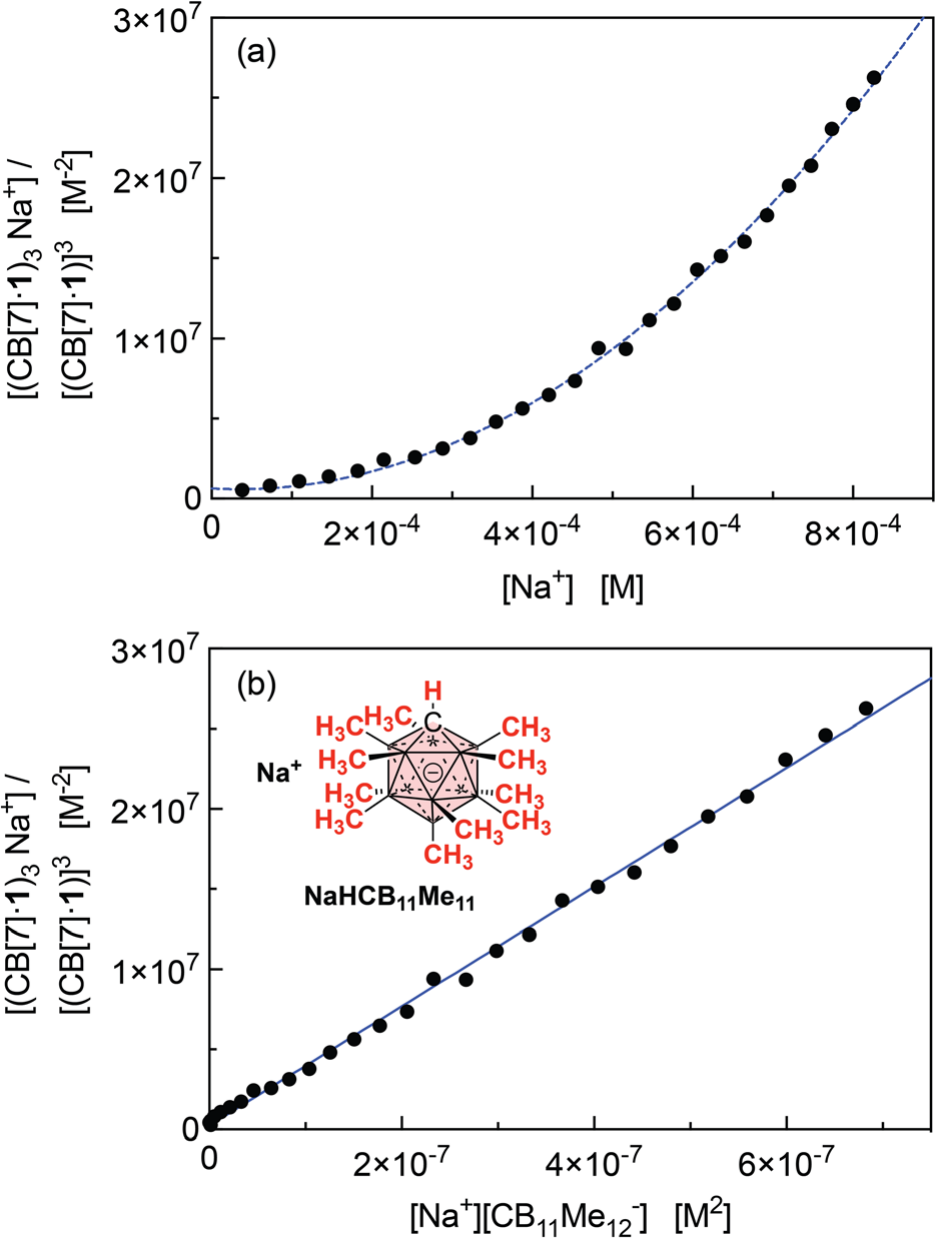
(a) Plot of [(CB[7]·1)_3_M]/[(CB[7]·1)]^3^ as a function of [M], for M = Na^+^ (counteranion CB_11_(CH_3_)_12_^−^). Formation of a sodium-secured trimer should afford a linear regression, not a quadratic one. (b) Plot of [(CB[7]·1)_3_M]/[(CB[7]·1)]^3^ as a function of [M][X], for M = Na^+^ and X = HCB_11_(CH_3_)_11_^−^, and linear regression.

**Fig. 6:**
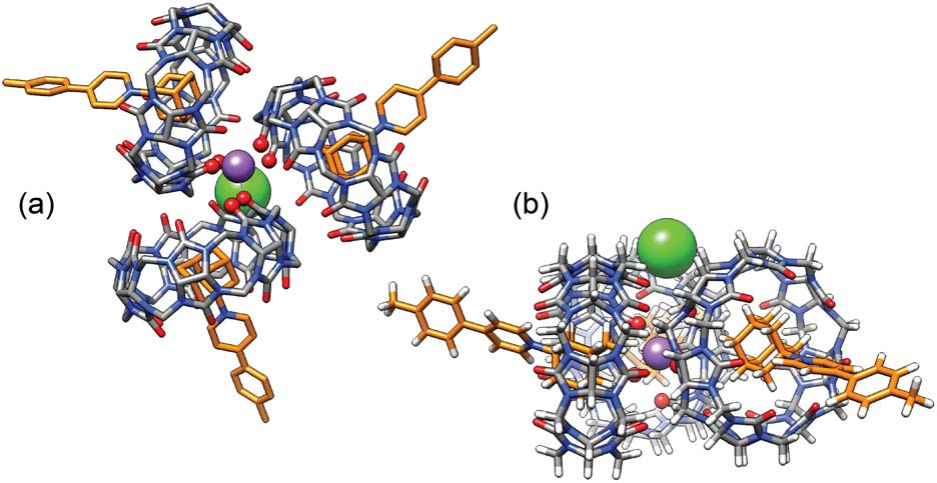
(a) “Top” and (b) “side” views of GFN2-xTB-optimized^27–29^ sodium-secured trimer (CB[7]·1)_3_M–X in conjunction with the ALPB solvation model for DMSO.^30^ The sodium cation is deeply nested between the CB[7] carbonylated portals and the chloride anion is perched into a shallow cavity between pseudoequatorial and pseudoaxial methylene groups. Hydrogen atoms omitted for clarity in the top view.

The concentrations of cation-secured trimer (CB[7]·1)_3_M and binary complex CB[7]·1 can be readily obtained from the integration of their respective NMR signals. The concentration of free cation M is obtained by subtracting the concentration of the cation-secured trimer from the total cation concentration in solution. Should equilibrium (4) be valid, a plot of [(CB[7]·1)_3_M]/[(CB[7]·1)]^3^ as a function of [M] should return a straight line. This is not the case (see Fig. 5a for an illustrative example with NaHCB_11_(CH_3_)_11_)! Straight lines were not obtained either when considering the putative formation of cation-secured dimers (CB[7]·1)_2_M or simple adducts CB[7]·1·M or a combination thereof. The excellent goodness-of-fit of the quadratic trend in Fig. 5a led us to question the role of the counteranion in the assembly process, as we had also noticed that sodium fluoride does not afford any trimer. We then considered equilibrium (5) and its corresponding equilibrium constant *β*_23_ (eqn (6)) in which an ion-pair (solvent separated or not) is formed between anion X and the cation-secured trimer (see Fig. 6 for structures optimized with the semi-empirical GFN2-xTB method^27–29^ in conjunction with the ALPB solvation model^30^ for DMSO).

5 3(CB[7]·1) + M + X ⇄ (CB[7]·1)_3_M–X

6
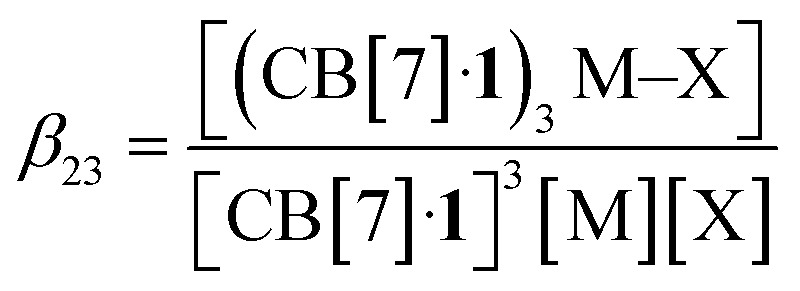


Plotting [(CB[7]·1)_3_M]/[(CB[7]·1)]^3^ as a function of [M][X] (with [M] = [X] in the case of monovalent cations) returns excellent linear correlations (*R*^2^ = 0.998 for NaHCB_11_(CH_3_)_11_, Fig. 5b)! As those pairs cannot be observed by mass spectrometry (Fig. 4), the ion-pairing is likely loose, or even solvent-separated in solution.

**Fig. 7:**
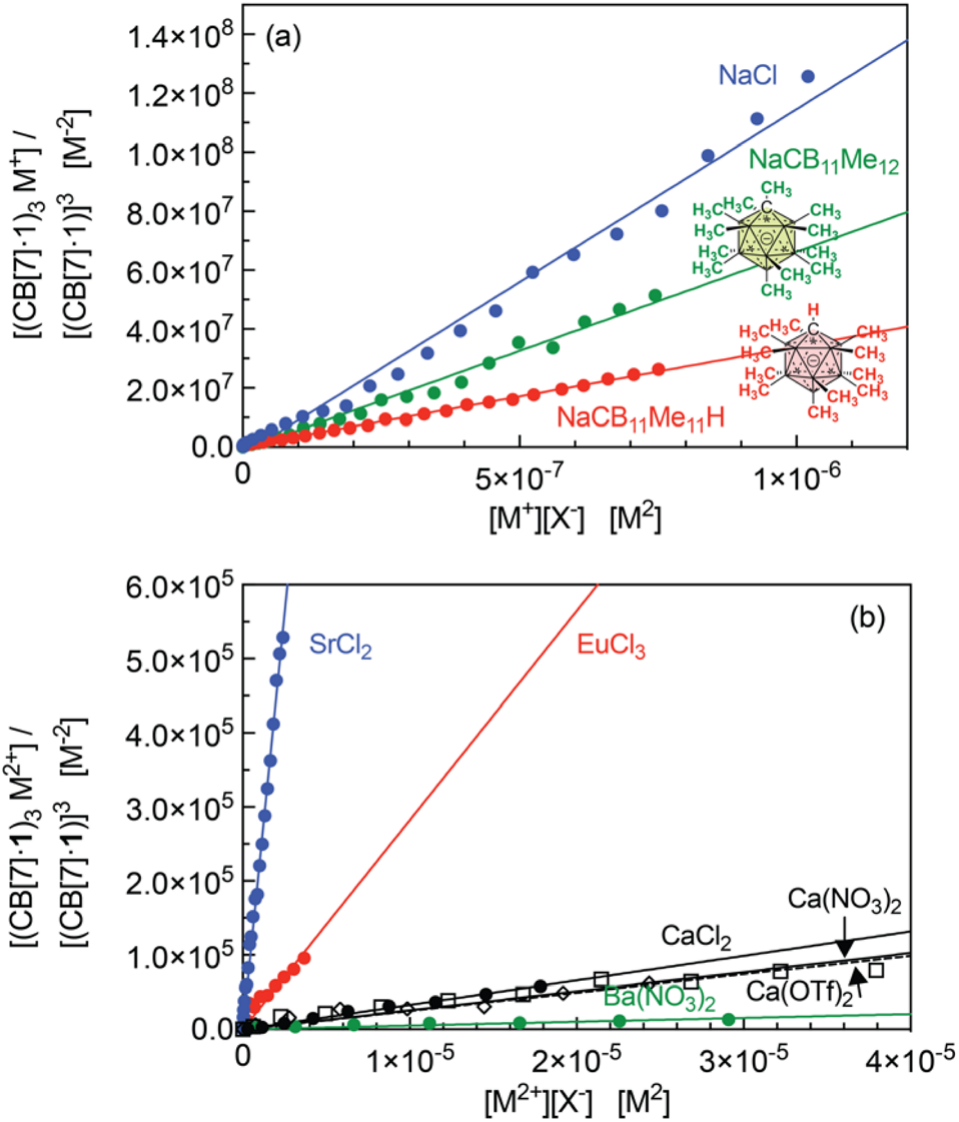
Plot of [(CB[7]·1)_3_M]/[(CB[7]·1)]^3^ as a function of [M][X], for (a) alkali cations M^+^ and (b) alkali-earth cations M^2+^ as well as Eu^3+^, with linear regressions. Titration affording the best coefficient of determination for each series.

Equilibrium constant *β*_23_ (in M^−4^) for all cations that afford the trimeric aggregate are listed in Table 1 after extraction from the slopes of the linear regressions presented in Fig. 7. On average, the sodium cation affords the highest constant (up to 1.1 × 10^14^ M^−4^); a 2.4-fold decrease is measured with potassium chloride compared to sodium chloride. Alkali-earth cations afford much lower equilibrium constants (up to 1.9 × 10^11^ M^−4^ for strontium), followed by an approximately 80-fold decrease with calcium and further 15-fold decrease with barium. In each series, ionic radii of 1.0–1.3 Å return the highest equilibrium constants, while significantly smaller cations (like lithium and magnesium) or larger cations (like rubidium and cesium) do not allow trimerization.

**Table 1:** Binding constants *β*_23_ for the formation of cation-secured CB[7]·1 trimers with various cations and counteranions^a^

	*r* b	*β*_23_ [M^−4^]		*r* b	*β*_23_ [M^−4^]
NaCl^c^	1.0	1.1 (±0.1) × 10^14^	CaCl_2_	1.0	3.2 (±0.1) × 10^9^
NaCB_11_Me_11_H^c^	1.0	3.4 (±0.2) × 10^13^	Ca(NO_3_)_2_	1.0	2.1 (±0.2) × 10^9^
NaCB_11_Me_12_	1.0	6.7 (±0.2) × 10^13^	Ca(OTf)_2_	1.0	1.9 (±0.2) × 10^9^
KCl^d^	1.3	4.4 (±0.1) × 10^13^	SrCl_2_	1.3	1.9 (±0.4) × 10^11^
			Ba(NO_3_)_2_	1.4	1.6 (±0.5) × 10^8^
			EuCl_3_	0.95	2.4 (±0.2) × 10^10^

a In M^−4^; average over duplicates unless mentioned otherwise.

b Ionic radii in Å.

c Average of 6 titrations.

d Equilibrium constant *β*_23_ obtained by competition experiment with the Na-secured CB[7]·1 trimer (chloride anions).

We note that the equilibrium constant for the formation of the potassium-secured trimer was obtained using a competition experiment between the sodium-secured trimer and potassium chloride; both aggregates can be readily identified (Fig. S73†).

7 (CB[7]·1) + M ⇄ (CB[7]·1)M

8 (CB[7]·1) + (CB[7]·1)M ⇄ (CB[7]·1)_2_M

9 (CB[7]·1) + (CB[7]·1)_2_M ⇄ (CB[7]·1)_3_M

10 (CB[7]·1)_3_M + X ⇄ (CB[7]·1)_3_M–X

To rationalize the formation of the cation-secured trimers, we decompose equilibrium (5) into separate binding events 7–10, with binding constants *K*_1_, *K*_2_, *K*_3_ and *K*_*X*_, respectively. As assemblies (CB[7]·1)_3_·M–X and CB[7]·1 are the preponderant species in solution, extreme cooperativity must be present with equilibrium constant *K*_3_ becoming far greater than *K*_2_ and *K*_1_. Evidence for cooperativity is provided by the ^1^H NMR titration experiments. If *K*_1_, *K*_2_ and *K*_3_ were similar and significantly above 10^3^ M^−1^, trimer (CB[7]·1)_3_M would be the dominant species upon addition of 0.33 equiv. MCl, dimer (CB[7]·1)_2_M after addition of 0.67 equiv. MCl and finally complex CB[7]·1·M in the presence of at least 1.0 equiv. MCl (as ion pairs or not). This is clearly not the case here: for example, upon addition of 0.33 to 1.0 equiv. NaCl, the ratio of trimer (CB[7]·1)_3_·M–X *vs.* complex CB[7]·1 increases steadily from 0.5 to 3.3.

When the cation-secured trimer is not formed, *i.e.* in water and with the relevant cations in DMSO, cation binding to complex CB[7]·1 can be fitted with a 1 : 1 binding model using equilibrium (7). In those cases, constant *K*_1_ must be significantly higher than *K*_2_ and *K*_3_, *i.e.* negative cooperativity is observed. We do not have evidence for the formation of ion pairs in these cases. As complexes CB[7]·1 and CB[7]·1·M show distinct UV-Vis absorption features in both solvents, a collection of spectra obtained upon addition of aliquots of salts could be fitted with a 1 : 1 binding model to extract equilibrium constant *K*_1_, assuming no cooperativity between both CB[7] portals in the absence of guest, and no trimer formation (see Table 2).

**Table 2:** Logarithmic binding constants for 1 : 1 complexation of inorganic cations with CB[7] in H_2_O and DMSO

Cation	*r* (Å)	H_2_O				DMSO			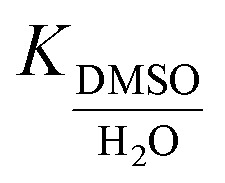	
		CB[7]^a^		CB[7]·1^b^	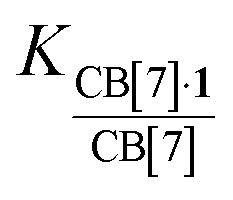	CB[7]^a^	CB[7]·1^e^	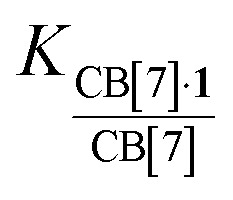	CB[7]	CB[7]·1
		UV-Vis^c^	ITC^d^	UV-Vis^e^		UV-Vis^b^	UV-Vis^b^			
Li^+^	0.78	1.4 (±0.3)	2.34	1.72 (±0.08)	2.4 × 10^−1^	2.01 (±0.01)	3.23 (±0.01)	17	4.7 × 10^−1^	32
Na^+^	0.98	2.2 (±0.1)	3.41	2.79 (±0.01)	2.4 × 10^−1^	2.98 (±0.09)	g		3.7 × 10^−1^	
K^+^	1.33	1.9 (±0.6)	3.46	2.75 (±0.01)	1.9 × 10^−1^	1.90 (±0.02)	g		2.8 × 10^−2^	
Rb^+^	1.49	2.8 (±0.5)	3.43	2.23 (±0.02)	6.3 × 10^−2^	2.60 (±0.01)	2.46 (±0.01)	0.72	1.5 × 10^−1^	1.7
Cs^+^	1.65	2.8 (±0.5)	3.50	1.8 (±0.1)	2.0 × 10^−2^	2.21 (±0.02)	2.91 (±0.01)	5.0	5.1 × 10^−2^	13
Mg^2+^	0.72	1.6 (±0.5)	3.24	2.50 (±0.02)	1.8 × 10^−1^	1.47 (±0.02)	1.91 (±0.05)	2.8	1.7 × 10^−2^	0.26
Ca^2+^	1.06	3.2 (±0.1)	4.25	2.63 (±0.06)	2.4 × 10^−2^	1.77 (±0.02)	g		3.3 × 10^−3^	
Sr^2+^	1.27	3.6 (±0.4)	4.79	1.72 (±0.01)	8.5 × 10^−4^	2.10 (±0.02)	g		2.0 × 10^−3^	
Ba^2+^	1.43		5.28	3.17 (±0.03)	7.8 × 10^−3^	2.00 (±0.01)	g		5.2 × 10^−4^	
Eu^3+^	0.95	3.3 (±0.2)	4.92^e^^,^^f^	3.54 (±0.01)	4.2 × 10^−2^	1.86 (±0.02)	g		8.7 × 10^−4^	
NH_4_^+^	1.61		2.82	2.03 (±0.04)	1.6 × 10^−1^	1.63 (±0.01)	2.33 (±0.02)	5.0	6.5 × 10^−2^	2.0

a log *K* for equilibrium CB[7] + M ⇄ CB[7]·M.

b log *K*_1_ for equilibrium (7).

c From ref. 29.

d Isothermal titration calorimetry; from ref. 30.

e This study.

f ITC binding affinity not reported; log *K* = 4.92 (±0.01) obtained by UV-Vis spectroscopy in this study.

g Formation of cation-secured trimer.

Binding affinities of most alkali and alkali-earth cations towards CB[7] in water have been measured in earlier studies, either by UV-Vis titrations^31^ or by isothermal titration calorimetry (ITC; values used for the discussion below).^32^ Binding affinities of alkali cations towards CB[7] in water range from log *K* = 2.3–3.5, and doubly charged alkali-earth cations from log *K* = 3.2–5.3 (see Table 2). A pronounced decrease in binding affinity towards cations is measured upon encapsulation of guest 1 (130-fold on average, see Table 2, with *K*(CB[7]·1)/*K*(CB[7]) consistently lower than 1). We attribute this difference to weak interactions between the cations and the hydrophobic adamantyl unit upon cation binding to the CB[7] rim. To the contrary, in the absence of any guest, the few water molecules present in the CB[7] cavity must provide additional solvation to the cation. Cation binding affinities for CB[7] in DMSO are significantly lower than in water (down to 1900-fold for Ba^2+^ but the median decrease is approximately 36-fold). However, remarkably, encapsulation of guest 1 enhances the affinity of the assembly towards cations in DMSO (up to 17-fold, 6-fold on average, see Table 2). Furthermore, affinities of complex CB[7]·1 towards cations are higher in DMSO than in water (by up to 32-fold, 10-fold on average, see Table 2). We conclude here that the encapsulated adamantyl group provides a better, albeit weak and essentially dispersive stabilization to the cations compared to encapsulated DMSO molecules, whose orientations or positions relative to the cation might just not be suitable for any significant interaction.

**Fig. 8:**
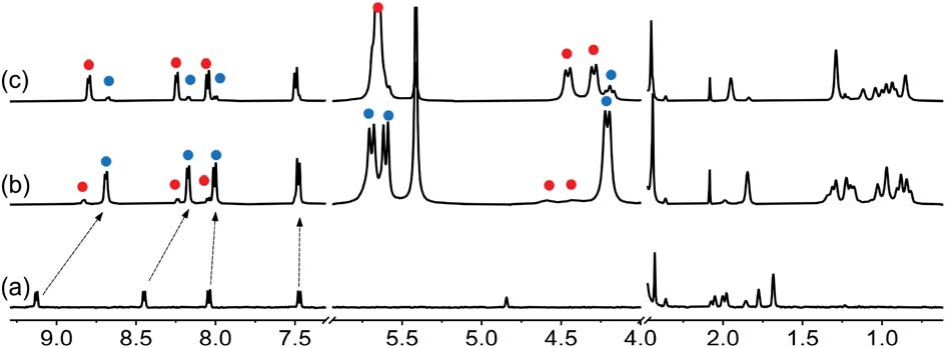
^1^H NMR spectra of (a) guest 2, (b) complex CB[7]·2 upon addition of 1.0 equiv. CB[7], and (c) assembly (CB[7]·2)_3_Na upon addition of NaCl (2.4 equiv.) in DMSO-d_6_. Chemical shifts in ppm.

**Fig. 9:**
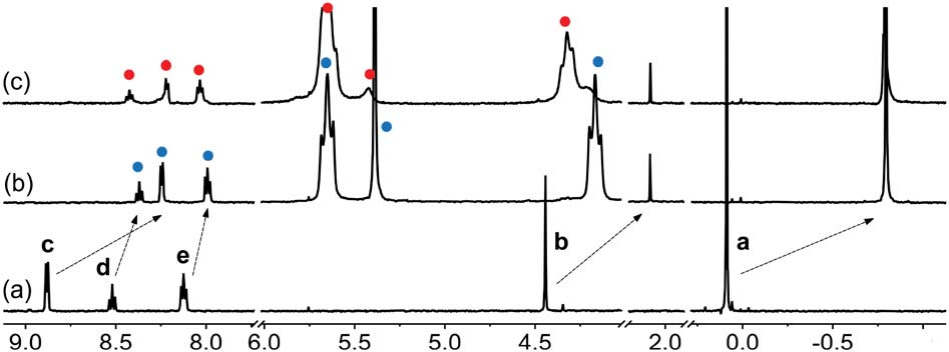
^1^H NMR spectra of (a) guest 4, (b) complex CB[7]·4 upon addition of 2.0 equiv. CB[7], and (c) assembly (CB[7]·4)_3_Na upon addition of NaCl (3.0 equiv.) in DMSO-*d*_6_. See Fig. 1 for signal labeling. Chemical shifts in ppm.

A set of 4 additional CB[7] guests were tested for cation-secured trimer formation in DMSO (Fig. 1, guests 2–5). Guests 2 and 4 form the assembly (Fig. 8 and 9); monitoring its formation is particularly straightforward using the trimethylsilyl unit of guest 4, as the methyl hydrogens undergo upfield shifts (by 0.89 ppm) upon encapsulation into CB[7], and a subsequent downfield shift (by only 0.01 ppm) upon trimer formation. We note that the formation of complex CB[7]·4 requires 2.0 equiv. CB[7] for quantitative formation, due to its weak affinity in DMSO, in stark contrast with the sub-nanomolar affinity reported in D_2_O for parent guest (trimethylsilyl)methanamine (8.9 × 10^8^ M^−1^).^8^

**Fig. 10:**
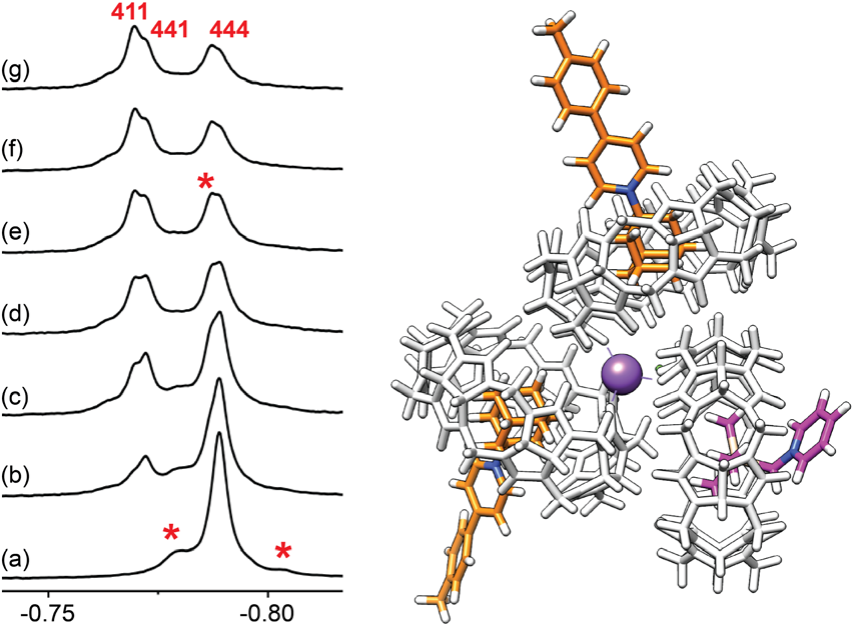
^1^H NMR spectra of (a) assembly (CB[7]·4)_3_Na–Cl (labeled “444”), and after addition of (b) 0.25, (c) 0.50, (d) 0.75, (e) 1.00, (f) 1.25 and (g) 1.50 equiv. of assembly (CB[7]·1)_3_Na–Cl. Hetero-assemblies (labeled “441” and “411”, see above) can be readily identified. Other larger assemblies are also observed, albeit in very small quantities (flagged with an asterisk). GFN2-xTB-optimized structure of complex (CB[7]·1)_2_(CB[7]·4)Na–Cl (“411”). Chemical shifts in ppm.

To further test the formation of cation-secured trimers, we carried out self-sorting experiments by adding aliquots of assembly (CB[7]·1)_3_Na–Cl to assembly (CB[7]·4)_3_Na–Cl (up to 1.5 equiv., labelled “444” in Fig. 10). While slow on the NMR time scale, guest/CB[7] exchanges and ingression/egression of CB[7]/guest complexes allow the rapid formation of hetero-assemblies (CB[7]·1)_2_(CB[7]·4)Na–Cl, (labelled “411”), and (CB[7]·1)(CB[7]·4)_2_Na–Cl (labelled “441”). Three ^1^H NMR signals for the trimethylsilyl units corresponding to homo-assembly (CB[7]·4)_3_Na–Cl and the pair of hetero-assemblies can be isolated (−0.788, −0.772 and −0.770 ppm, respectively, see Fig. 10).

Finally, we used guest 5 as a negative control, as both CB[7] portals in complex CB[7]·5 interact with the guest's pyridinium groups. Complex CB[7]·5 was indeed insensitive to the addition of NaCl and the cation-secured trimer did not form.

A key feature of the trimerization is its solvent-dependency: it is only observed in DMSO and not in D_2_O. To justify this effect, we assessed equilibrium (11) by computational methods using neutral adamantane (6) instead of charged guest 1, as errors on solvation energies, which are critical to explain the mechanism, are significantly lower with neutral or singly charged species compared to polycationic ones like trimer [(CB[7]·1)_3_Na]^4+^.

11 3(CB[7]·6) + Na^+^ ⇄ [(CB[7]·6)_3_Na]^+^

Computational methods allow the separation of (1) the electronic contributions to the equilibrium (*i.e.* at 0 K) in the gas phase Δ*E*, (2) the enthalpic and entropic correction at 25 °C *δ*Δ*G*, and (3) the solvation contributions Δ*G*_solv_ (see eqn (12)).^33^

12 Δ*G* = Δ*E* + *δ*Δ*G* + Δ*G*_solv_

Complex CB[7]·6 and sodium-secured trimer [(CB[7]·6)_3_Na]^+^ were optimized in the gas phase using the semi-empirical method GFN2-xTB developed by Grimme and coworkers to extract the electronic term Δ*E*.^27–29^ Vibrational analysis was carried out with the same method to extract term *δ*Δ*G*. Single-point density functional theory calculations at the bp/def2-TZVP level with and without the COSMO solvation model were used as input to calculate the solvation energies Δ*G*_solv_ of the assemblies in water and DMSO using CosmoTherm.^34^ Using single-point calculations on pre-optimized structures is a minor simplification of the procedure developed by Klamt and coworkers^35^ which involves a re-optimization of the structures at the DFT level with and without the COSMO model, *i.e.* we neglect the likely very small geometry adjustment of the assemblies when transferred from the gas phase to the continuum solvation environment. The free solvation energies for Na^+^, K^+^ and NH_4_^+^, which are notoriously challenging to calculate or measure,^36^ were taken from a study by Truhlar and coworkers.^37,38^

In the absence of cation, GFN2-xTB calculations show that trimerization is favorable in the gas phase (by 108.4 kcal mol^−1^ at 0 K and by 63.6 kcal mol^−1^ at 25 °C, that includes the mostly entropic penalty for aggregation, see Table 3). This is supported by MS experiments carried out by Da Silva^39^ and Dearden^40^ who have shown significant aggregation of CB[*n*]s in the gas phase. The penalty for the partial desolvation of 3 (out of 6) CB[7] portals during the trimerization is overwhelming, however (106.9 and 98.1 kcal mol^−1^ in water and DMSO, respectively, see Table 3), which makes the trimerization unfavorable in solution, in agreement with our experiments. Securing the trimer with a sodium cation is extremely favorable in the gas phase (−214.6 kcal mol^−1^) due to coulombic interactions between the cation and at least 2 carbonyl groups of each CB[7] macrocycle. The desolvation penalty of the sodium cation and of half of all CB[7] portals is of course very penalizing (210.8 and 200.3 kcal mol^−1^ in water and DMSO, respectively), but still makes the trimerization process slightly favorable in solution (by 3.8 and 14.3 kcal mol^−1^ in water and DMSO, respectively). While we do not observe trimerization in water, a 4 kcal mol^−1^ error on such large favorable or unfavorable contributions is perfectly acceptable; furthermore, calculations correctly predict that the trimerization is more favorable in DMSO! Calculations with potassium afford an unfavorable trimerization in water (by 10.9 kcal mol^−1^), and a favorable one in DMSO (by 8.1 kcal mol^−1^, see Table 3), but slightly less so than with sodium (14.3 kcal mol^−1^), in sharp agreement with experimental results (see Table 2). A significant decrease in coulombic interactions is observed with the ammonium cation (−160.1 *vs.* −214.6 kcal mol^−1^ with Na^+^), yet the desolvation penalty is just as strong; the overall trimerization is thus unfavorable in either solvent, again in agreement with experiments.

**Table 3:** Calculated energy terms associated with the trimerization of CB[7]-bound adamantane (6) [kcal mol^−1^]

	Δ*E*^a^	*δ*Δ*G*^b^	Δ*G*_gas_^c^	Δ*G*_solv_^d^		ΔΔ*G*_solv_^e^	Δ*G*^f^	
				Water	DMSO		Water	DMSO
No cation	−108.4	44.9	−63.6	106.9	98.1	−8.8	43.4	34.6
Na^+^	−270.4	55.8	−214.6	210.8	200.3	−10.5	−3.8	**−14.3**
K^+^	−258.1	55.4	−202.7	213.6	194.7	−18.9	**10.9**	**−8.1**
NH_4_^+^	−217.8	57.6	−160.1	209.8	194.5	−15.4	49.7	34.3

a Electronic contributions to equilibrium (11) (*i.e.* at 0 K) in the gas phase.

b Enthalpic and entropic corrections at 25 °C.

c Free energy of reaction for equilibrium (11) in the gas phase.

d Solvation contributions to equilibrium (11).

e Solvation contributions in DMSO relative to water.

f Free energy of reaction for equilibrium (11) in solution (water or DMSO).

The calculations provide us with the root cause of the preference for trimerization in DMSO: the penalty for the partial desolvation of three of the six CB[7] portals upon aggregation is less unfavorable in DMSO compared to water. Cluster (CB[7]·6)_3_, with 3 partially shielded CB[7] portal is less solvated (*i.e.* less stabilized) in DMSO compared to water by 35.6 kcal mol^−1^, but three complexes CB[7]·6, with six solvent-exposed portals, are less solvated by 44.4 kcal mol^−1^, bringing the solvation balance to 8.8 kcal mol^−1^ in favor of the trimerization in DMSO (Table 2). This is expected, as DMSO, unlike water, is not a hydrogen-bond donor to the carbonylated portals of CB[7]. When sodium is added, (CB[7]·6)_3_Na is less solvated (*i.e.* less stabilized) in DMSO compared to water by just 28.2 kcal mol^−1^, but again, complex three complexes CB[7]·6 are less solvated by 44.4 kcal mol^−1^, bringing the solvation balance to 16.2 kcal mol^−1^ in favor of the trimerization in DMSO; however, the desolvation penalty of the sodium cation is *more* penalizing in DMSO^37,38^ (108.9 kcal mol^−1^*vs.* 103.2 kcal mol^−1^ in water, *i.e.* a 5.7 kcal mol^−1^ difference for Na^+^), bringing back the solvation balance to just 10.5 kcal mol^−1^, again in favor of the trimerization in DMSO (see Table 3; *vs.* 8.8 kcal mol^−1^ in the absence of sodium). The DMSO/water balance is even more shifted towards trimerization in DMSO with potassium (by 18.9 kcal mol^−1^, Table 3).

Continuum solvation models do not allow a proper determination of solvation energies for macrocycles with deep and small cavities that can only accommodate a few discrete solvent molecules. Therefore we cannot use the methodology proposed above to explain why free CB[7] does not afford trimers in any solvent, regardless of the nature of the cation. Using equilibrium (13), where G is either a guest like a non-polar adamantyl unit, or an encapsulated polar solvent molecule, we tentatively propose that the solvent molecule would better stabilize three assemblies [CB[7]·G·Na]^+^ (three sodium cations interacting with three polar solvent molecules) compared to assembly [(CB[7]·G)_3_·Na]^+^ (only one sodium cation interacting with three polar solvent molecules). This effect would significantly shift equilibrium (13) away from trimerization.

13 3[CB[7]·G·Na]^+^ ⇄ [(CB[7]·G)_3_·Na]^+^ + 2Na^+^

One can also readily rationalize why some cations promote trimerization and some do not based on their cationic radii. The virtual (CB[7]·G)_3_ trimer essentially behaves as a cryptand with exceptional affinity for cations of adequate volumes in DMSO. All cations that promote trimerization have cationic radii ranging from 0.98 Å (Na^+^) to 1.43 Å (Ba^2+^), while Li^+^, Mg^2+^, Rb^+^, Cs^+^ and NH_4_^+^ (radii 0.69, 0.79, 1.49, 1.65 and 1.61 Å, respectively) are either too small or too large to fit within the trimer central pocket (see Fig. 1).^41,42^

Finally, calculations support the formation of an ion pair between assembly [(CB[7]·6)_3_·Na]^+^ and the chloride anion using equilibrium (5). The solvation energy of both Na^+^ and Cl^−^ anions from the gas phase to DMSO (−171.6 kcal mol^−1^)^37^ was obtained from the lattice energy of NaCl in the solid state and its solubility in DMSO. The error on the calculated solvation energy of the ion-paired assembly (−231 kcal mol^−1^) is likely high, as the anion remains partially exposed to the solvent, and the COSMO-RS formalism is not designed to return accurate solvation energies for charged species directly exposed to the solvent. Calculations return 32 kcal mol^−1^ for the strength of the ion-pair interaction, which is obviously a vast overestimation; but it certainly does not invalidate its formation.

## Conclusions

We have shown that CB[7] forms well-defined trimeric assemblies in DMSO, as long as (1) a selection of alkali- (Na^+^, K^+^), alkali-earth (Ca^2+^, Sr^2+^, Ba^2+^) or at least one cation of the lanthanide series (Eu^3+^) is added to the solution, and (2) the cavity of CB[7] is filled with a guest that leaves one carbonylated portal available for cation binding. In other terms, the CB[7]/guest trimer, with its carbonyl groups pointing towards its inner core, acts as a cryptand with exceptional affinity to these cations in DMSO. The driving forces for the trimerization are (1) coulombic interactions between the 3 carbonylated CB[7] rims and the cations, and (2) favorable CH–O interactions between the carbonylated rim of a CB[7] macrocycle and the equatorial and pseudoequatorial hydrogens of a neighboring CB[7] unit. In addition to the entropically unfavorable aggregation, free energy penalties are (1) the desolvation of the cations upon nesting into the trimer, and (2) the desolvation of 3 out of 6 CB[7] carbonylated rims upon trimerization. The latter is significantly less unfavorable in DMSO compared to water or deuterium oxide, hence the solvent-selective process. This study paves the way for the design, and subsequent assessment of their recognition properties, of well-defined two- and three-dimensional CB[*n*]-containing clusters in organic solvents.

## Data availability

All analytical data is provided in the narrative and in the ESI.†

## Author contributions

J. K. and E. M. conceived the project. D. L., F. M., J. Š. and M. D. carried out all experiments. S. G., B. J. B. and J. C. provided mass spectrometry support. I. C. determined all single-crystal X-ray diffraction structures. E. M. carried out all computational work. E. M. and J. K. wrote the manuscript through contributions of all authors.

## Conflicts of interest

There are no conflicts to declare.

## Supplementary Material

SC-014-D3SC02032K-s001

SC-014-D3SC02032K-s002
