# Supplementary material for: Solvent-controlled formation of alkali and alkali-earth-secured cucurbituril/guest trimers
Source: Chem Sci. 2023 Aug 15;14(35):9258–66. doi: 10.1039/d3sc02032k (PMC10498720; doi:10.1039/d3sc02032k)
Supplement: SC-014-D3SC02032K-s001 [file SC-014-D3SC02032K-s001.pdf]

Electronic Supplementary Material (ESI) for Chemical Science.
This journal is © The Royal Society of Chemistry 2023

Supporting Information

Solvent-Controlled Formation of Alkali and Alkali-Earth-Secured Cucurbituril/guest Trimers

Doroteja Lončarić,[a,b,‡] Fahimeh Movahedifar,[c,‡] Jakub Radek Štoček,[a,b] Martin Dračínský,[a] Josef Cvačka,[a] Shanshan Guan,[c] Benjamin J. Bythell,[c] Ivana Císařová,[d] Eric Masson,*[,c] Jiří Kaleta*[,a]

[a] Institute of Organic Chemistry and Biochemistry of the Czech Academy of Sciences, Flemingovo nám. 2, 160 00 Prague 6, Czech Republic. [b] Department of Organic Chemistry, Faculty of Science, Charles University in Prague, 128 40 Prague 2, Czech Republic. [c] Department of Chemistry and Biochemistry, Ohio University, Athens, Ohio 45701, United States. [d] Department of Inorganic Chemistry, Faculty of Science, Charles University in Prague, 128 40 Prague 2, Czech Republic.

‡ *Equal contribution.*

Table of Contents

1. General Information S1
2. Synthetic Procedures S2
3. NMR Spectra of Prepared Compounds S6
   3.1. Compound **1** S6
   3.2. Compound **2** S12
   3.3. Compound **3** S18
   3.3. Compound **4** S24
   3.4. Compound **5** S26
3. Preparation of **CB[7]·1** and **[(CB[7]·1)$_3$·Na]Cl** S28
4. ^{1}H NMR Titration Experiments S30
   4.1. Titrations of Guests with **CB[7]** S30
   4.2. Titrations of Guest **CB[7]·1** with Alkali and Earth-Alkali Metal Salts S37
   4.3. ^{1}H NMR Titration of **[(CB[7]·1)$_3$·K]Cl** with **NaCl** S54
   4.4 Addition of D_2O to a Mixture of **CB[7]·1** and **[(CB[7]·1)$_3$·Na]Cl** S55
   4.5 Competition Experiments with 15-Crown-5 .... S56
5. MS Analysis S59
6. UV-Vis Titration Experiments S62
7. X-ray Crystallographic Data S75
Table S1 Parameters of Single Crystals of **1**, **2**, **3**, and **7** S75
   7.1. Compound **1** S76
   7.2. Compound **2** S78
   7.3. Compound **3** S80
   7.3. Compound **7** S82
8. References S84

Corresponding Authors
Jiří Kaleta (🖂 jiri.kaleta@uochb.cas.cz)
Eric Masson (🖂 masson@ohio.edu)

1. General Information

Standard Schlenk and vacuum line techniques were employed for all manipulations of air- or moisture-sensitive compounds. Yields refer to isolated and spectroscopically homogeneous materials.

Starting material used for synthesis of guests and titration experiments was purchased from following sources: abcr (Karlsruhe, Germany), Combi-Blocks (San Diego, CA), Sigma-Aldrich (St. Louis, MO; Prague, Czech Republic), TCI America (Portland, OR), Fisher Scientific (Fair Lawn, NJ), Oakwood Chemical (Estill, SC), Strem Chemicals (Newburyport, MA) and used without further purification. Anhydrous dimethyl sulfoxide (DMSO) was used as solvent in UV-Vis titrations. Solvents used in preparation of samples for NMR measurements were purchased from Cambridge Isotope Laboratories (Andover, MA) and Eurisotop. Deuterated dimethyl sulfoxide (DMSO-d_6) was distilled over calcium hydride as needed. Cucurbit[7]uril (CB[7]) was prepared using known procedures.[1] The apparent molar mass of CB[7] was determined by recording its ^{1}H NMR spectrum with *p*-toluidine hydrochloride as an internal standard. Sodium dodecamethylcarba-*closo*-dodecaborate ($NaCB_{11}Me_{12}$) and sodium 2,3,4,5,6,7,8,9,10,11,12-undecamethylcarba-*closo*-dodecaborate ($NaHCB_{11}Me_{11}$) were prepared by cation exchange from cesium salts of the same anions,[2] which were synthesized according to the literature procedures.[2,3] Solvents were of analytical grade and either used as purchased or dried according to previously described procedures.[4] 1-Adamantylamine and 2-adamantylamine were isolated from hydrochloride salt following the published procedure.[5]

Characterization of prepared guests by Nuclear Magnetic Resonance spectroscopy (NMR) was carried out using a Bruker Avance III^{TM} HD 400MHz Prodigy spectrometer at the Institute of Organic Chemistry and Biochemistry, Prague. ^{1}H NMR titration experiments, varying temperature experiments, DOSY and NOESY experiments were carried out using a Bruker Avance II^{TM} 500MHz and JEOL JNM-EZCR 500 MHz spectrometers at the Institute of Organic Chemistry and Biochemistry, Prague, and Bruker Ascend 500 MHz at the department of Chemistry and Biochemistry, Ohio University. ^{1}H and ^{13}C NMR chemical shifts are reported in parts per million (ppm) and are referenced to the residual signal of solvent. Coupling constants (J) are reported in hertz (Hz). Standard abbreviations were used to indicate multiplicity: s = singlet, d = doublet, t = triplet, q = quartet, m = multiplet, dd = doublet of doublets.

HRMS-ESI(+) spectra were collected using a Thermo Fisher Scientific Q Exactive Plus hybrid quadrupole–Orbitrap mass spectrometer. Absorption spectra were recorded using an Agilent Cary 8454 Diode Array UV-Visible Spectrophotometer (1 nm resolution, 0.5 s integration time). Measurements were obtained in a 1 × 1 cm quartz cuvette at 25 °C. ReactLabTM EQUILIBRIUM PRO software (Jplus Consulting Pty Ltd of Australia) was used to for the determination of binding constants based on UV-Vis titrations.

2. Synthetic Procedures

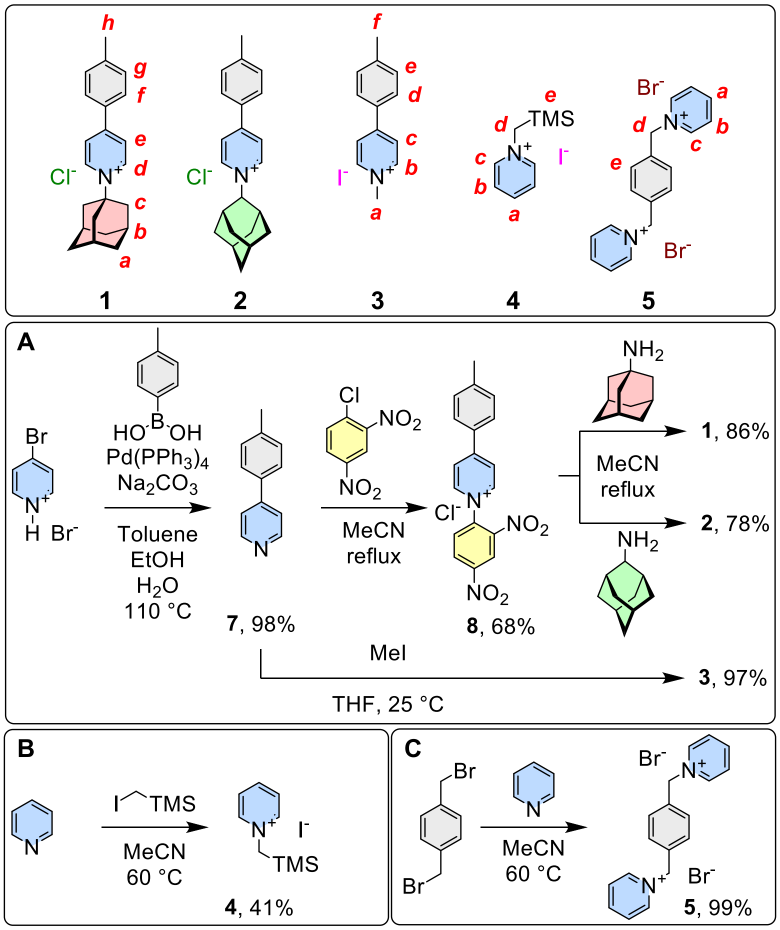

Figure S1. Synthesis of guests **1-3** (A), **4** (B), and **5** (C).

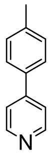

4-(4-Tolyl)pyridine (**7**) was prepared following modified procedure.[6] 4-Bromopyridine hydrochloride (2.2 g, 12 mmol, 1.2 eq) was dissolved in toluene (80 mL). Solution of *p*-tolylboronic acid (1.3 g, 9.6 mmol, 1.0 eq) in ethanol (56 mL) was added, followed by addition of $NaHCO_3$ (4.1 g, 39 mmol, 4.1 eq) in water (24 mL). The mixture was degassed for 10 min using nitrogen. Lastly, $Pd(PPh_3)_4$ (540 mg, 0.5 mmol, 0.05 eq) was added. The reaction mixture was stirred for 17 hours at 70 °C under nitrogen atmosphere. The progress was monitored by ^{1}H NMR. Sample was prepared by taking 1 mL aliquot from the reaction mixture, evaporating the solvent and dissolving the residue in deuterated solvent. After completion solvents were evaporated, the crude mixture was suspended in water (50 mL) and extracted with dichloromethane (3 × 50 mL). Subsequently, hydrochloric acid (2.8 M, 20 mL) was added to the combined organic phases. Following separation, aqueous phase was transferred to Erlenmeyer flask equipped with magnetic stir bar and cooled in an ice bath. Powdered Na_2CO_3 was added until pH value of the solution was 12. The solution was then extracted with dichloromethane (3 × 35 mL). The organic phase was dried with anhydrous $MgSO_4$, filtered through cotton and evaporated giving **7** as a white powder (1.6 g, 9.4 mmol, 98%).

Mp 93 - 95 °C. ^{1}H NMR (400 MHz, DMSO-d_6): δ 2.36 (s, 3H), 7.33 (d, J = 6.2 Hz, 2H), 7.68 (d, J = 8.2 Hz, 2H), 7.70 (d, J = 6.2 Hz, 2H), 8.60 (d, J = 8.0 Hz, 2H). ^{13}C {^{1}H} NMR (100 MHz, DMSO-d_6): δ 21.2, 121.4, 127.1, 130.3, 134.7, 139.4, 147.3, 150.7. IR (KBr): 3035, 2919, 1914, 1675, 1614, 1598, 1583, 1541, 1521, 1488, 1457, 1414, 1404, 1387, 1332, 1317, 1235, 1212, 1131, 1112, 1094, 1073, 1028, 991, 962, 801, 736, 710, 668, 638, 563, 556, 474 cm^{-1}. MS, m/z (%): 171.1 (11, M+H), 170.1 (100, M), 169.1 (11). HRMS (+ESI) m/z: $[M + H]^+$ calcd for $C_{12}H_{12}N^+$ 170.0964; found 170.0963. Anal. calcd. for $C_{12}H_{11}N$: C, 85.17; H, 6.55; N, 8.29. Found: C, 84.97; H, 6.58; N 8.25.

1-(2,4-Dinitrophenyl)-4-(4-tolyl)pyridin-1-ium chloride (**8**) was prepared following modified procedure.[7] 4-(4-Tolyl)pyridine (0.80 g, 4.8 mmol, 1 eq) and 1-chloro-2,4-dinitrobenzene (3.2 g, 16 mmol, 3.4 eq) were dissolved in anhydrous acetonitrile (25 mL). The reaction mixture was refluxed for 2 days under nitrogen atmosphere. A dense precipitate was formed. The precipitate was filtered through sinter funnel and washed with acetonitrile (4 × 2 mL), hexane (3 × 2 mL) and pentane (3 × 2 mL). All volatiles were removed under reduced pressure. The solid residue was washed with acetonitrile (4 × 2 mL), hexane (3 × 2 mL) and pentane (3 × 2 mL). Both solid fractions were combined affording **8** as a yellow material (1.2 g, 3.3 mmol, 68%).

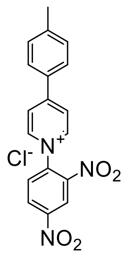

Mp 200 - 203 °C. ^{1}H NMR (400 MHz, DMSO-d_6): δ 2.46 (s, 3H), 7.53 (d, J = 7.9 Hz, 2H), 8.20 (d, J = 8.4 Hz, 2H), 8.46 (d, J = 8.6 Hz, 1H), 8.81 (d, J = 7.2 Hz, 2H), 8.99 (dd, J_1 = 8.7 Hz, J_2 = 2.6 Hz, 1H), 9.14 (d, J = 2.6 Hz, 1H), 9.38 (d, J = 7.1 Hz, 2H). ^{13}C {^{1}H} NMR (100 MHz, DMSO-d_6): δ 21.1, 121.5, 123.5, 128.7, 130.1, 130.2, 130.6, 132.1, 138.6, 143.3, 144.1, 145.7, 149.1, 157.2. IR (KBr): 3383, 3269, 3197, 3132, 3112, 3083, 3017, 2986, 2924, 1635, 1602, 1549, 1498, 1484, 1465, 1409, 1347, 1321, 1301, 1283, 1234, 1214, 1195, 1153, 1127, 1075, 1035, 1008, 970, 918, 912, 897, 880, 849, 838, 819, 771, 758, 743, 725, 707, 690, 671, 654, 635, 620, 594, 559, 540, 508 cm^{-1}. MS, m/z (%): 336.1 (100, M^+), 290.1 (25, $M^+ - NO_2$), 244 (50, $M^+ - 2 \times NO_2$). HRMS (+ESI) m/z: $[M]^+$ calcd for $C_{18}H_{14}O_4N_3^+$ 336.0979; found 336.0977. Anal. calcd. for $C_{18}H_{14}O_4N_3Cl$: C, 58.15; H, 3.80; N, 11.30. Found: C, 56.03; H, 3.98; N, 10.57.

1-(Adamantan-1-yl)-4-(4-tolyl)-pyridin-1-ium chloride (1). Compound **8** (0.37 g, 0.51 mmol, 1.0 eq) and 1-adamantylamine[5] (0.13 g, 0.83 mmol, 1.6 eq) were dissolved in anhydrous acetonitrile (15 mL). The reaction mixture was refluxed under nitrogen atmosphere for 2 hours. Solvent was removed under reduced pressure. Column chromatography on a silica gel (MeOH/CH_2Cl_2: 1-30%) yielded **1** as a yellow crystalline material (0.15 g, 0.71 mmol, 86%).

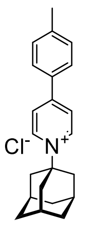

Mp 211 - 215 °C. ^{1}H NMR (400 MHz, DMSO-d_6): δ 1.76 (s, 6.21H), 2.32 (s, 9H), 2.43 (s, 3H), 7.47 (d, J = 7.8 Hz, 2H), 8.06 (d, J = 8.3 Hz, 2H), 8.46 (d, J = 7.3 Hz, 2H), 9.30 (d, J = 7.3 Hz, 2H). ^{13}C {^{1}H} NMR (100 MHz, DMSO-d_6): δ 21.0, 39.5, 34.5, 41.0, 68.3, 123.7, 128.2, 130.3, 130.4, 141.7, 142.8, 154.2. IR (KBr): 3432, 2919, 1631, 1604, 1449, 1127, 1102, 1050, 1028, 1009, 833, 817, 774, 742, 503, 470 cm^{-1}. MS, m/z (%): 304.2 (100, M^+). HRMS (+ESI) m/z: $[M]^+$ calcd for $C_{22}H_{26}N^+$ 304.2060; Found 304.2058. Anal. Calcd. for $C_{22}H_{26}NCl$: C, 77.74; H, 7.71; N, 4.13. Found: C, 76.44; H, 7.61; N, 4.07.

1-(Adamantan-2-yl)-4-(4-tolyl)-pyridin-1-ium chloride (2). Compound **8** (0.37 g, 1.0 mmol, 1.0 eq) and 2-adamantylamine[5] (0.26 g, 1.7 mmol, 1.7 eq) were dissolved in anhydrous acetonitrile (25 mL). The reaction mixture was refluxed under nitrogen atmosphere for 2 hours. Solvent was removed under reduced pressure. Column chromatography on silica gel (MeOH/CH_2Cl_2: 1-15%) afforded **2** that was contaminated with small amount of 2-adamantylamine hydrochloride, which was subsequently sublimed off using Kugelrohr distillation apparatus (230 °C, 750 mTorr). Compound **2** was isolated as a yellowish crystalline material (0.14 g, 1.3 mmol, 78%).

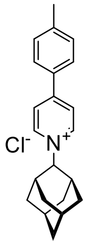

Mp 117 – 120 °C. ^{1}H NMR (400 MHz, DMSO-d_6): δ 1.68 (bs, 4H), 1.77 (bs, 2H), 1.86 (bs, 1H), 1.98-2.07 (m, 5H), 2.43 (s, 3H), 2.84 (bs, 2H), 4.87 (bs, 1H), 7.47 (d, J = 7.9 Hz, 2H), 8.05 (d, J = 8.4 Hz, 2H), 8.47 (d, J = 6.8 Hz, 2H), 9.17 (d, J = 6.8 Hz, 2H). ^{13}C {^{1}H} NMR (100 MHz, DMSO-d_6): δ 21.1, 26.0, 26.1, 30.1, 30.5, 36.3, 36.8, 70.9, 123.8, 128.2, 130.4, 130.4, 142.8, 143.3, 154.1. IR (KBr): 3113, 3058, 3027, 2908, 2853, 1633, 1607, 1550, 1534, 1497, 1456, 1407, 1369, 1346, 1296, 1244, 1211, 1136, 1117, 1100, 1075, 1035, 1012, 987, 967, 906, 824, 811, 793, 767, 735, 492 cm^{-1}. MS, *m/z* (%): 304.2 (100, M$^+$). HRMS (+ESI) *m/z*: [M]$^+$ calcd for $C_{22}H_{26}N^+$ 304.2060; Found 304.2059. Anal. calcd. for $C_{22}H_{26}NCl$: C, 77.74; H, 7.71; N, 4.13. Found: C, 75.70; H, 7.68; N, 3.86.

1-Methyl-4-(4-tolyl)pyridin-1-ium iodide (3) was prepared following modified procedure.[8] Compound **7** (420 mg, 2.5 mmol, 1 eq) was dissolved in anhydrous tetrahydrofuran (15 mL). Methyl iodide (1.4 mL, 22.5 mmol, 9 eq) was added. Reaction mixture was stirred under nitrogen for 90 hours. Formation of precipitate was observed. Product was filtered through sinter funnel, washed with THF (4 × 2 mL), hexane (3 × 2 mL) and pentane (3 × 2 mL), and subsequently dried under reduced pressure. 750 mg of yellow crystalline substance was obtained (2.4 mmol, 97%).

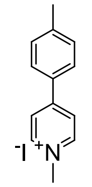

Mp 108 - 111 °C. ^{1}H NMR (400 MHz, DMSO-d_6): δ 2.42 (s, 3H), 4.31 (s, 3H), 7.46 (d, J = 8.0 Hz, 2H), 8.00 (d, J = 8.4 Hz, 2H), 8.48 (d, J = 6.7 Hz, 2H), 8.97 (d, J = 6.6 Hz, 2H). ^{13}C {^{1}H} NMR (100 MHz, DMSO-d_6): δ 21.0, 47.0, 123.5, 128.0, 130.3, 130.6, 142.6, 145.5, 154.1. IR (KBr): 3488, 3023, 1640, 1611, 1558, 1533, 1501, 1471, 1412, 1346, 1320, 1295, 1271, 1236, 1214, 1185, 1129, 1116, 1051, 1027, 979, 856, 842, 810, 784, 723, 487, 442 cm^{-1}. MS, *m/z* (%): 184.1 (100, M$^+$), 168.1 (14, M$^+$ – CH_3 - 1). HRMS (+ESI) *m/z*: [M]$^+$ Calcd for $C_{13}H_{14}N^+$ 184.1121; Found 184.1120. Anal. calcd. for $C_{13}H_{14}NI$: C, 50.18; H, 4.54. Found: C, 49.55; H, 4.56; N 4.39.

(Trimethylsilylmethyl)-pyridin-1-ium iodide (4) was prepared according to the previously reported procedure.[9] Pyridine (0.16 mL, 2.0 mmol) and (iodomethyl)-trimethylsilane (0.30 mL, 2.0 mmol) were mixed in dry acetonitrile (2.0 mL) and were subsequently stirred at 60 ºC for 7 h. A dense solid formed. The reaction mixture was filtered, the solid residue was washed with acetonitrile (3 × 2 mL) and finally thoroughly dried under reduced pressure. After evaporation of the solvent, the product was obtained as a brown-reddish solid (176 mg, 0.60 mmol, 30%).

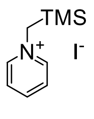

Mp 127 - 129 ºC. ^{1}H NMR (500 MHz, DMSO-d_6) δ 0.09 (s, 9H), 4.44 (s, 2H), 8.12 (t, J = 7.0 Hz, 2H), 8.52 (t, J = 7.8 Hz, 1H), 8.92 – 8.85 (m, 2H). ^{13}C {^{1}H} NMR (126 MHz, DMSO-d_6): δ -3.0, 53.9, 128.8, 144.2, 144.4. HRMS (+ESI) *m/z*: [M]$^+$ calcd for $C_9H_{16}INSi$ 166.1047; found 166.1047.

1,1'-[1,4-Phenylenebis(methylene)]bis(pyridin-1-ium) bromide (**5**) was prepared by dissolving 1,4-bis(bromomethyl)benzene (1.48 g, 5.6 mmol) in acetonitrile (10 mL), followed by addition of pyridine (1.0 mL, 12.4 mmol) to the solution. The reaction mixture was heated at 60 °C for 1 h and then at 40 °C overnight. A dense white solid precipitated. The solids were collected by filtration on a frit, washed with cold acetone (3 × 4 mL) and finally thoroughly dried under reduced pressure. Compound **5** was isolated as a white crystalline solid (1.92 g, 5.5 mmol, 99%).

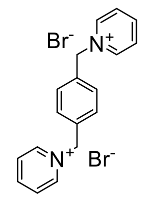

^{1}H NMR (500 MHz, DMSO-d_6) δ, 5.88 (s, 4H), 7.61 (s, 4H), 8.16 – 8.22 (m, 4H), 8.64 (tt, J_1 = 7.8, J_2 =1.4 Hz, 2H) 9.19 – 9.23 (m, 4H). ^{13}C {^{1}H} NMR (126 MHz, DMSO-d_6) δ 63.2, 129.0, 130.1, 135.8, 145.4, 146.6.

3. NMR Spectra of Prepared Compounds

3.1. Compound 1

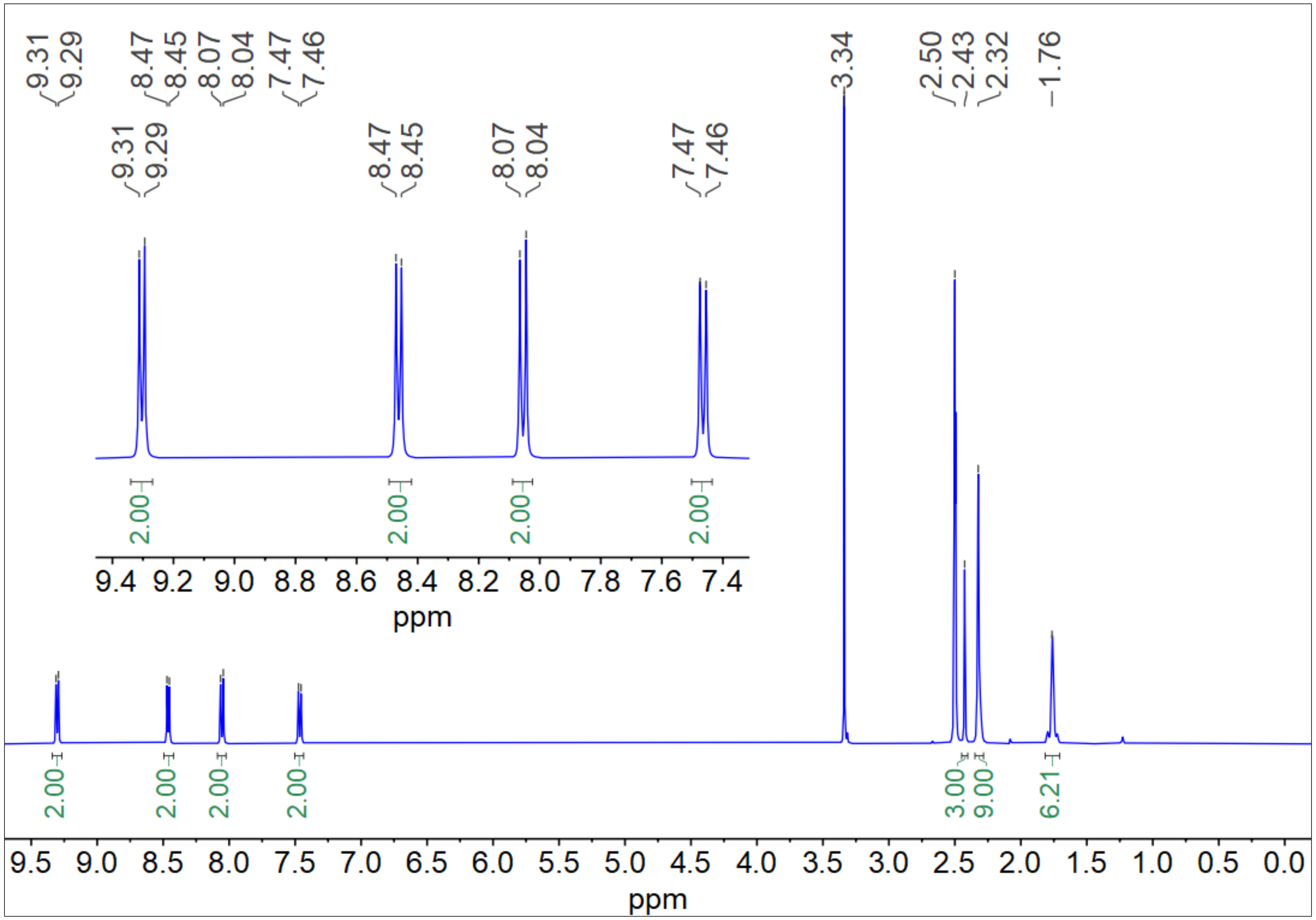

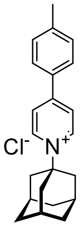

Figure S2. ^{1}H NMR (400 MHz, DMSO-d_6): 1-(Adamantan-1-yl)-4-(4-tolyl)-pyridin-1-ium chloride (**1**).

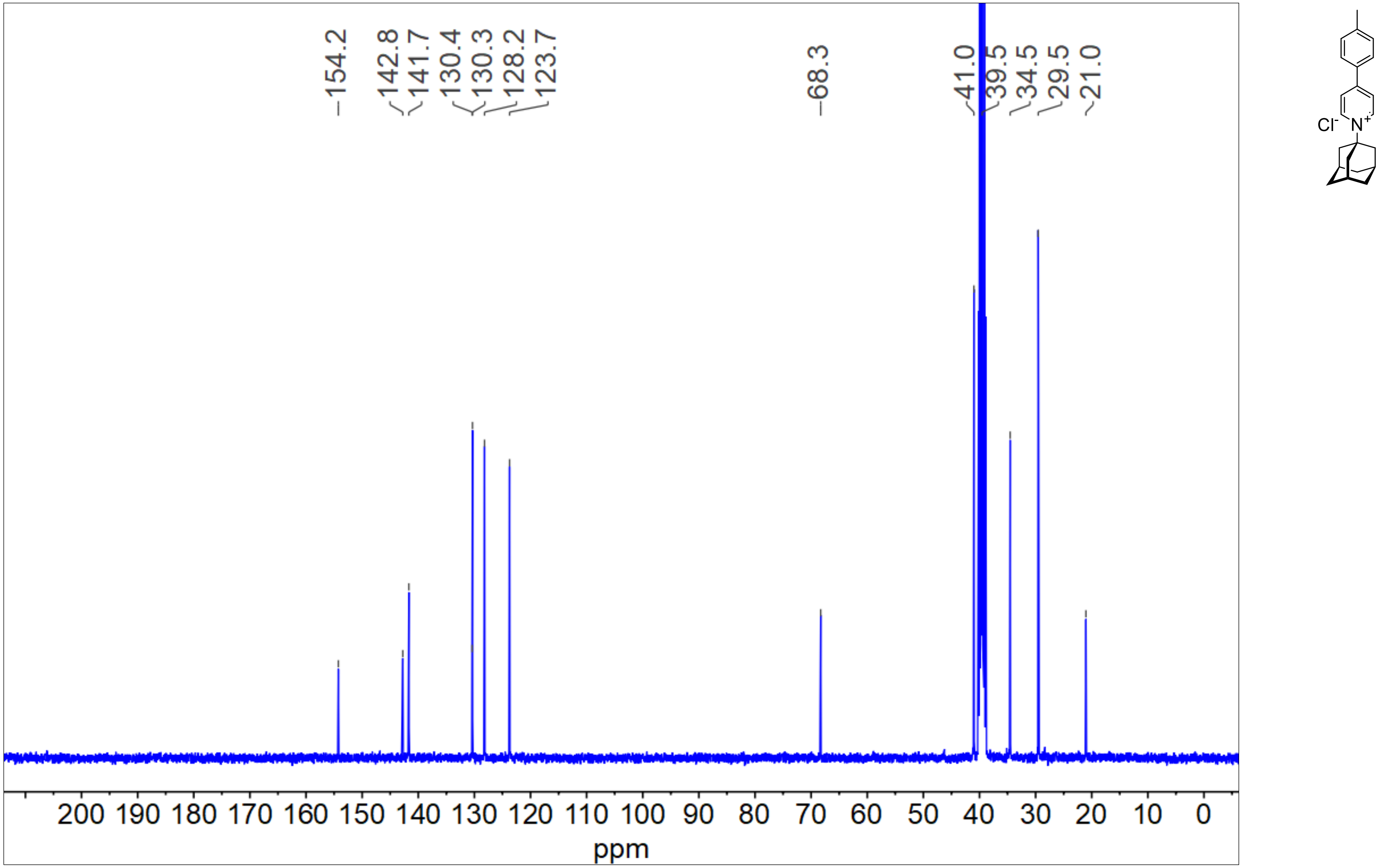

Figure S3. ^{13}C {^{1}H} NMR (100 MHz, DMSO-d_6): 1-(Adamantan-1-yl)-4-(4-tolyl)-pyridin-1-ium chloride (**1**).

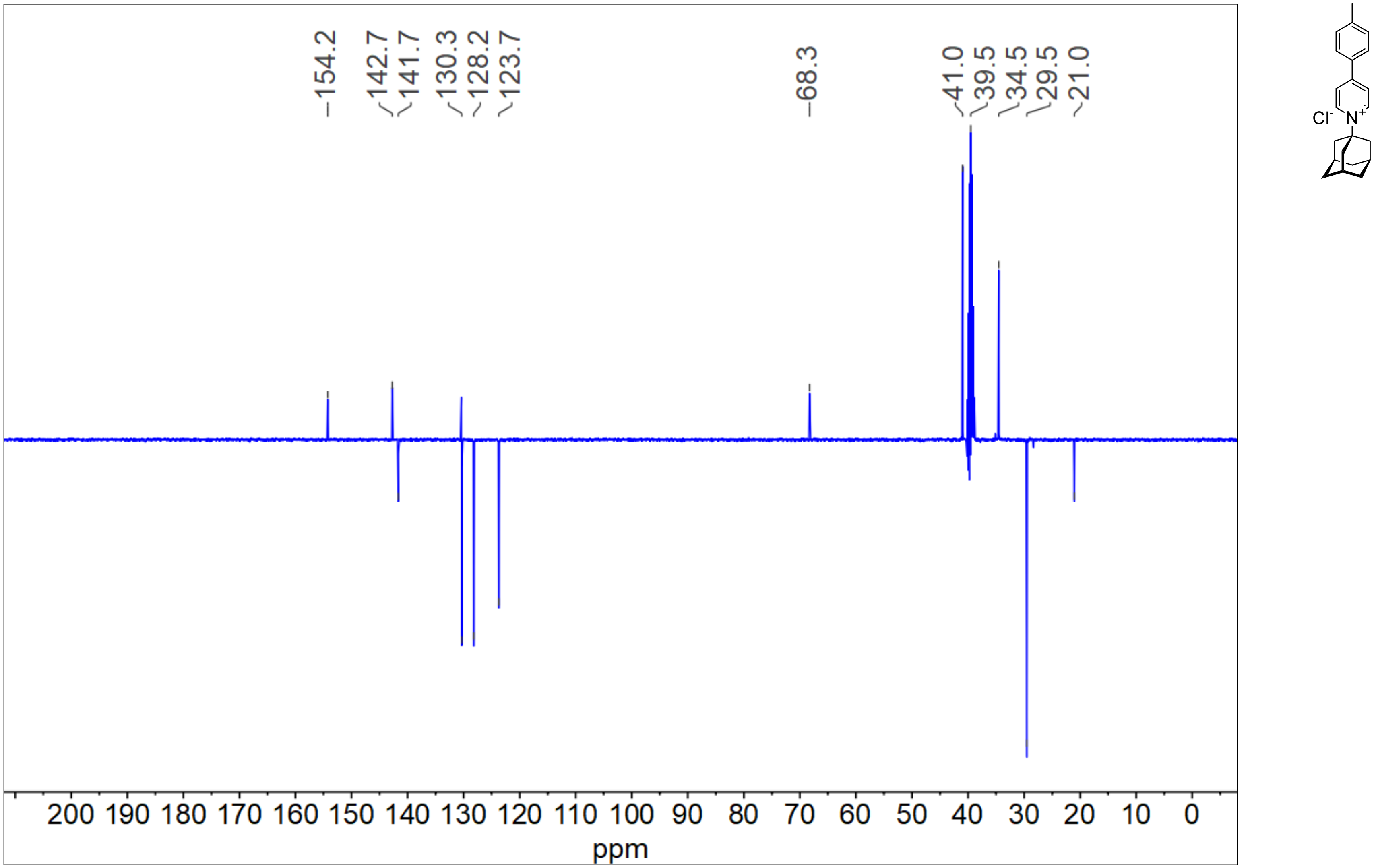

Figure S4. ^{13}C {^1H} APT NMR (100 MHz, DMSO-d_6): 1-(Adamantan-1-yl)-4-(4-tolyl)-pyridin-1-ium chloride (**1**).

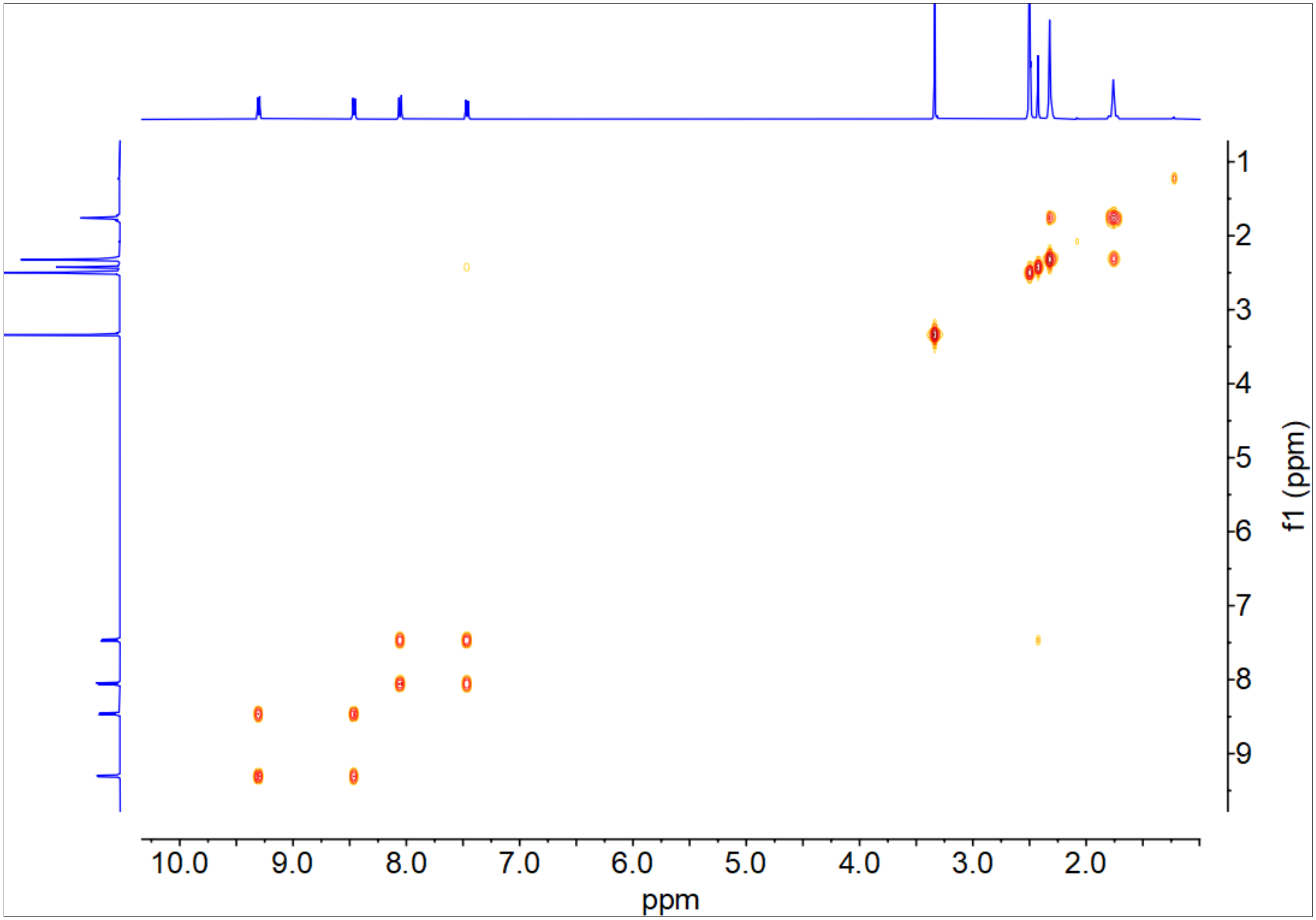

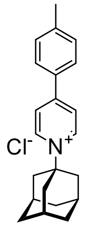

Figure S5. ^{1}H - ^{1}H COSY NMR (DMSO-d_6): 1-(Adamantan-1-yl)-4-(4-tolyl)-pyridin-1-ium chloride (**1**).

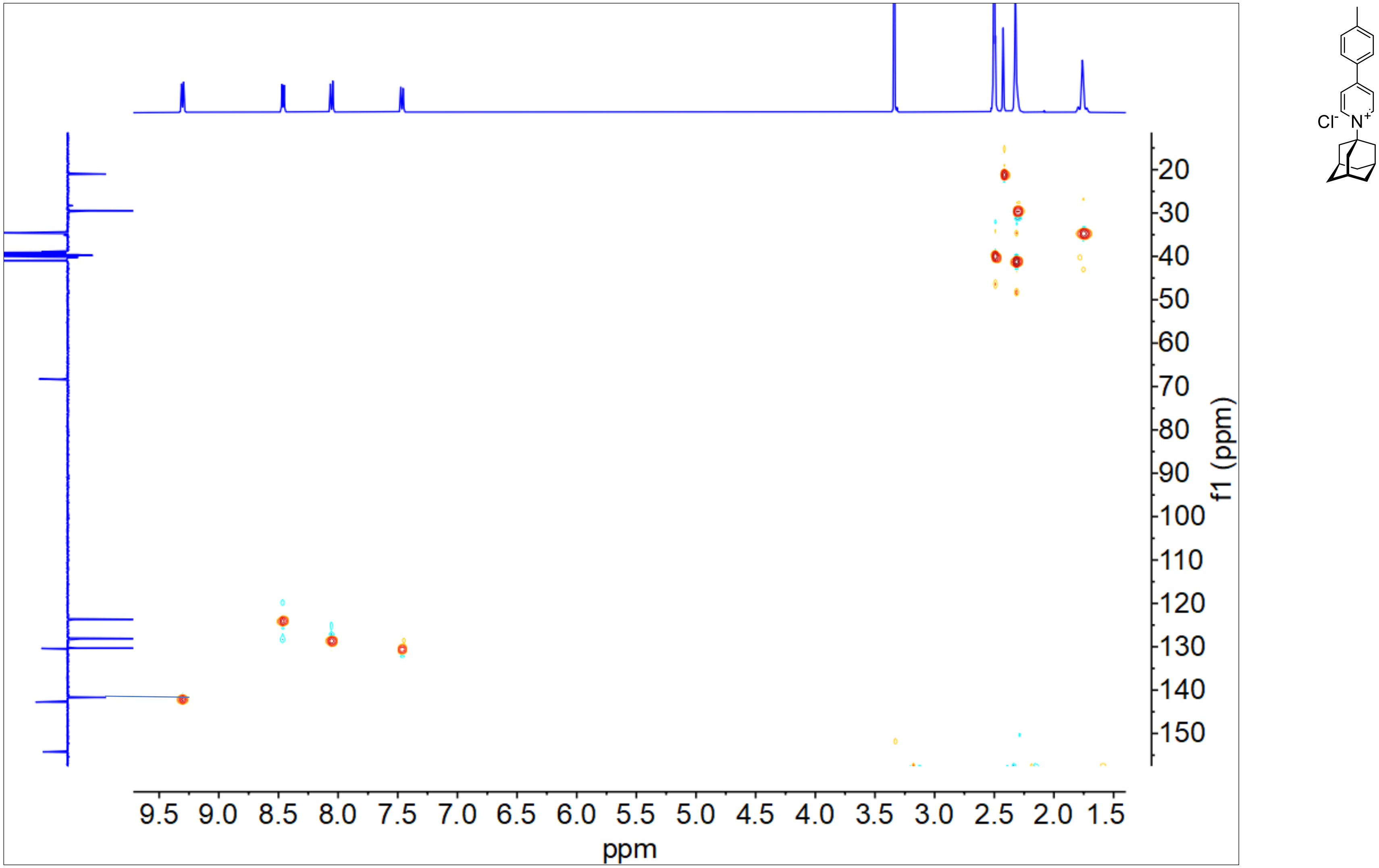

Figure S6. ^{1}H – ^{13}C HSQC NMR (DMSO-d_6): 1-(Adamantan-1-yl)-4-(4-tolyl)-pyridin-1-ium chloride (**1**).

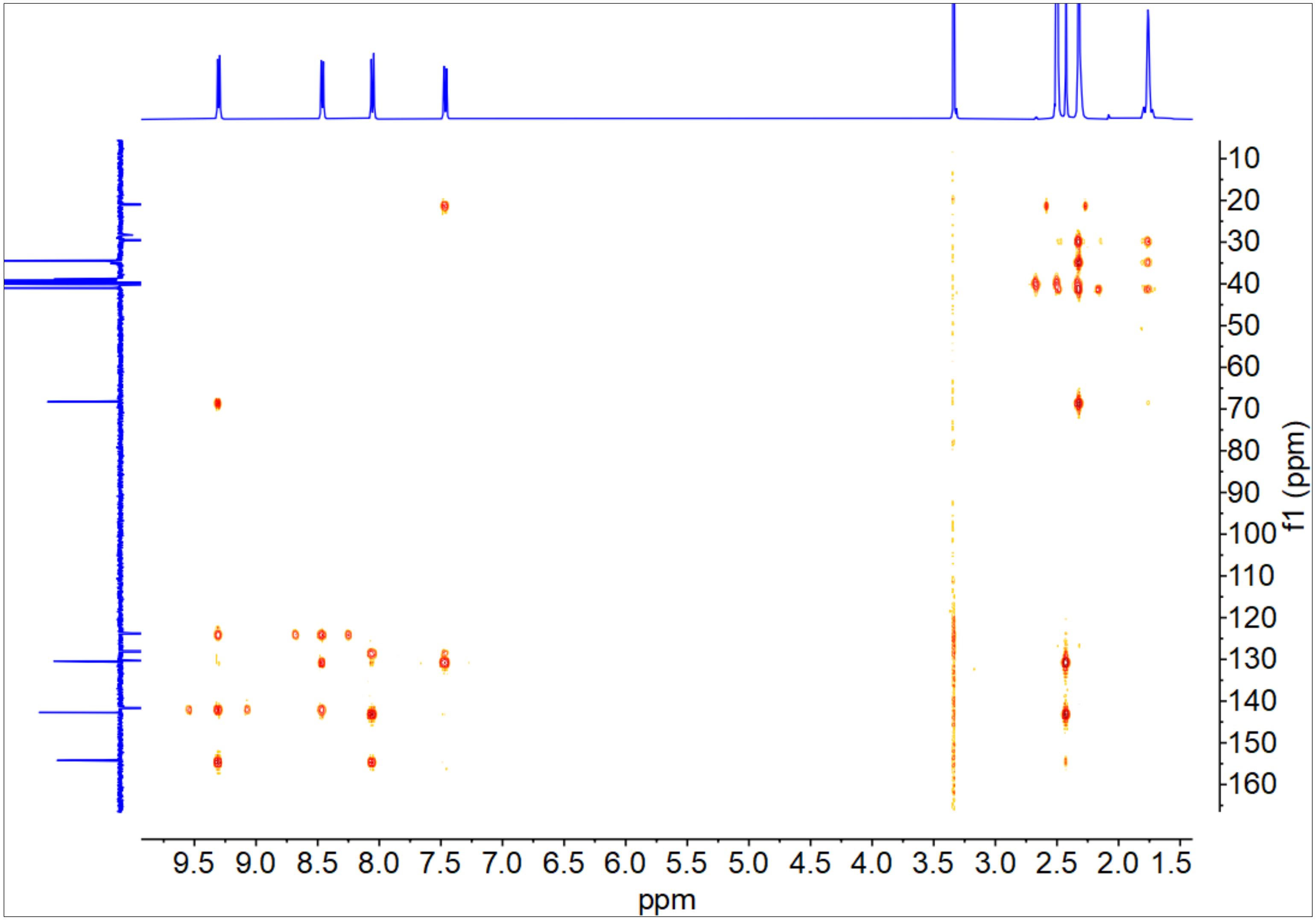

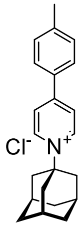

Figure S7. $^{1}H - ^{13}C$ HMBC NMR (DMSO-d_6): 1-(adamantan-1-yl)-4-(4-tolyl)-pyridin-1-ium chloride (**1**).

3.2. Compound 2

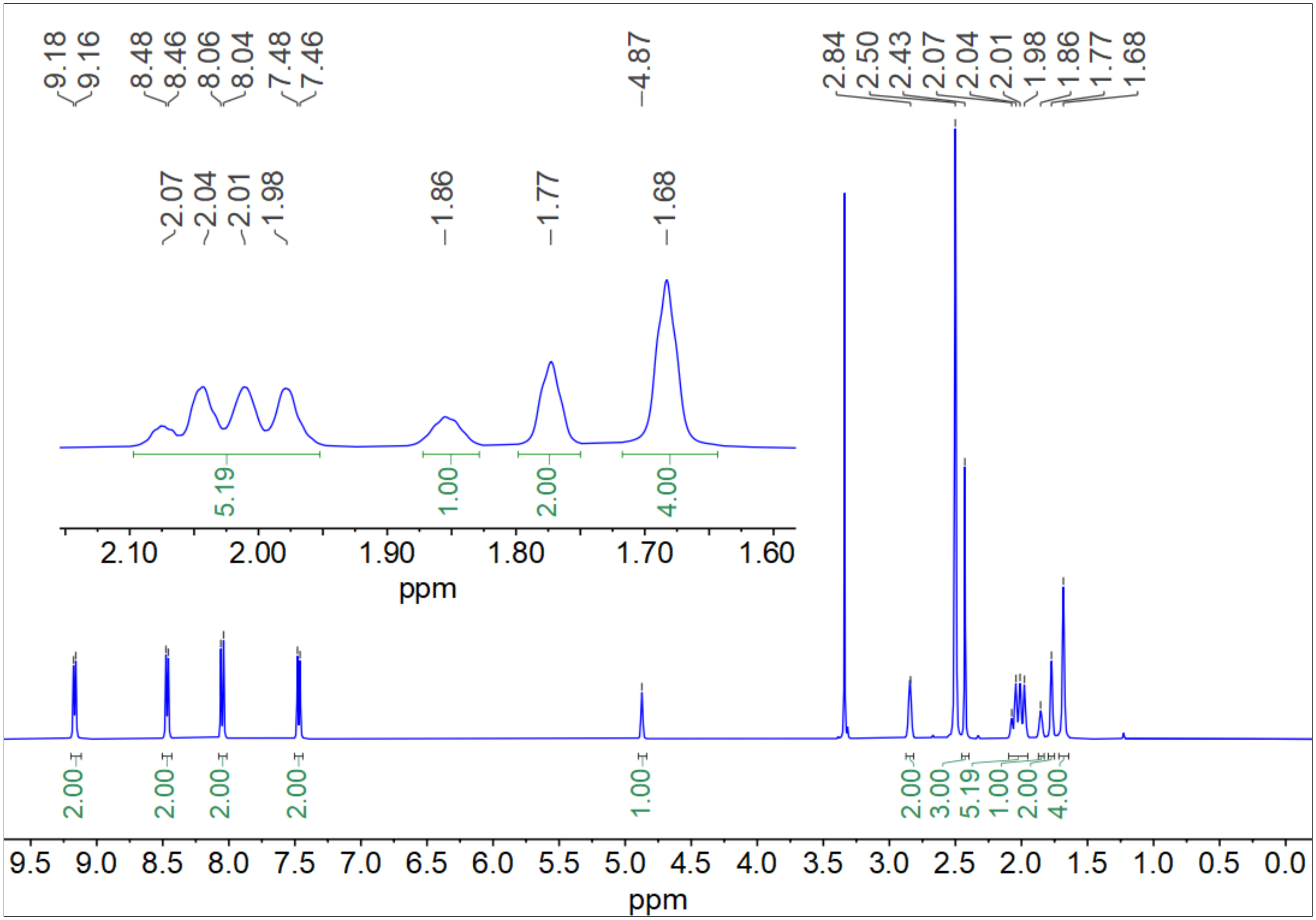

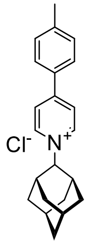

Figure S8. ^{1}H NMR (400 MHz, DMSO-d_6): 1-(Adamantan-2-yl)-4-(4-tolyl)-pyridin-1-ium chloride (**2**).

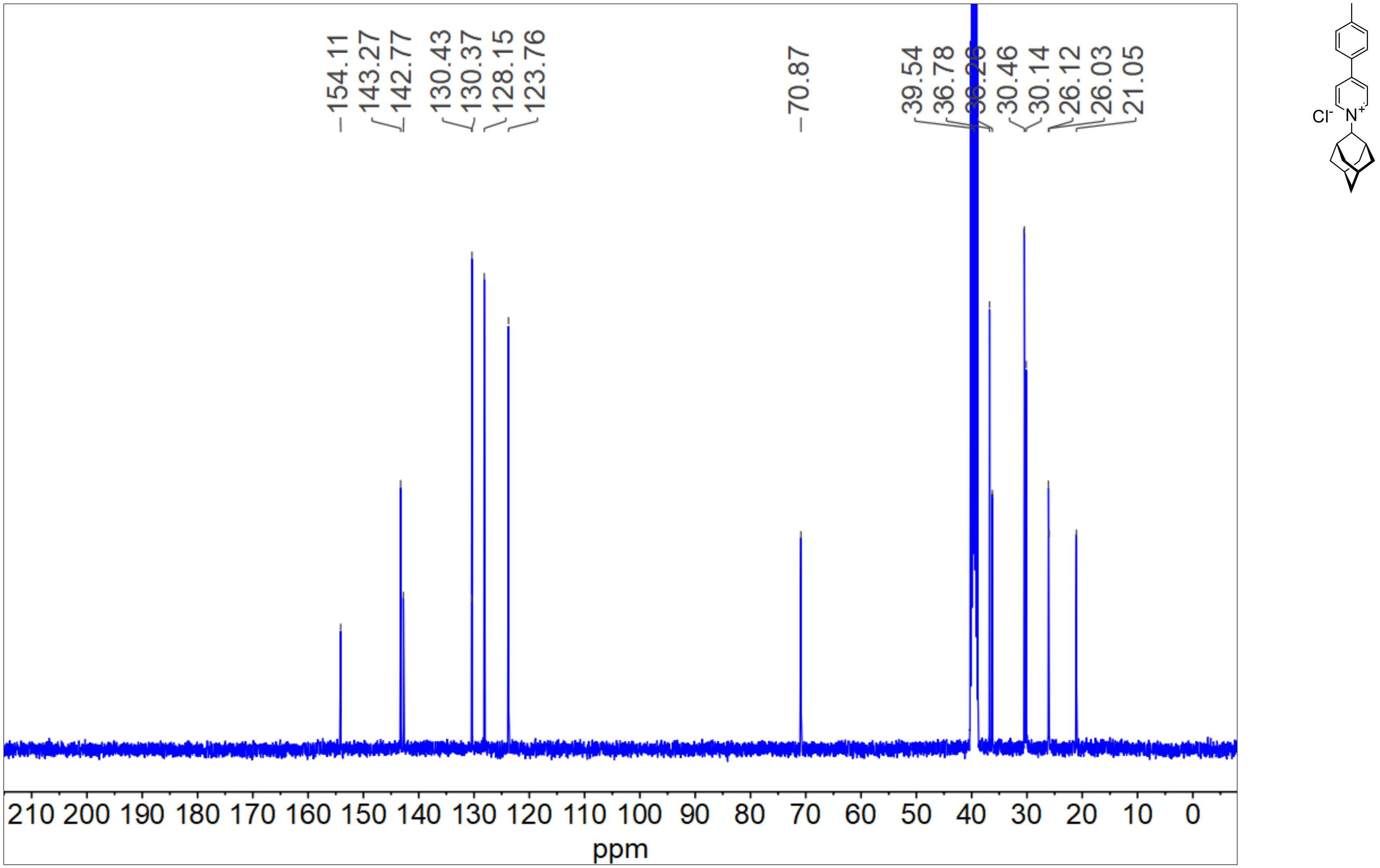

Figure S9. $^{13}C\ \{^{1}H\}$ NMR (100 MHz, DMSO-d_6): 1-(Adamantan-2-yl)-4-(4-tolyl)-pyridin-1-ium chloride (**2**).

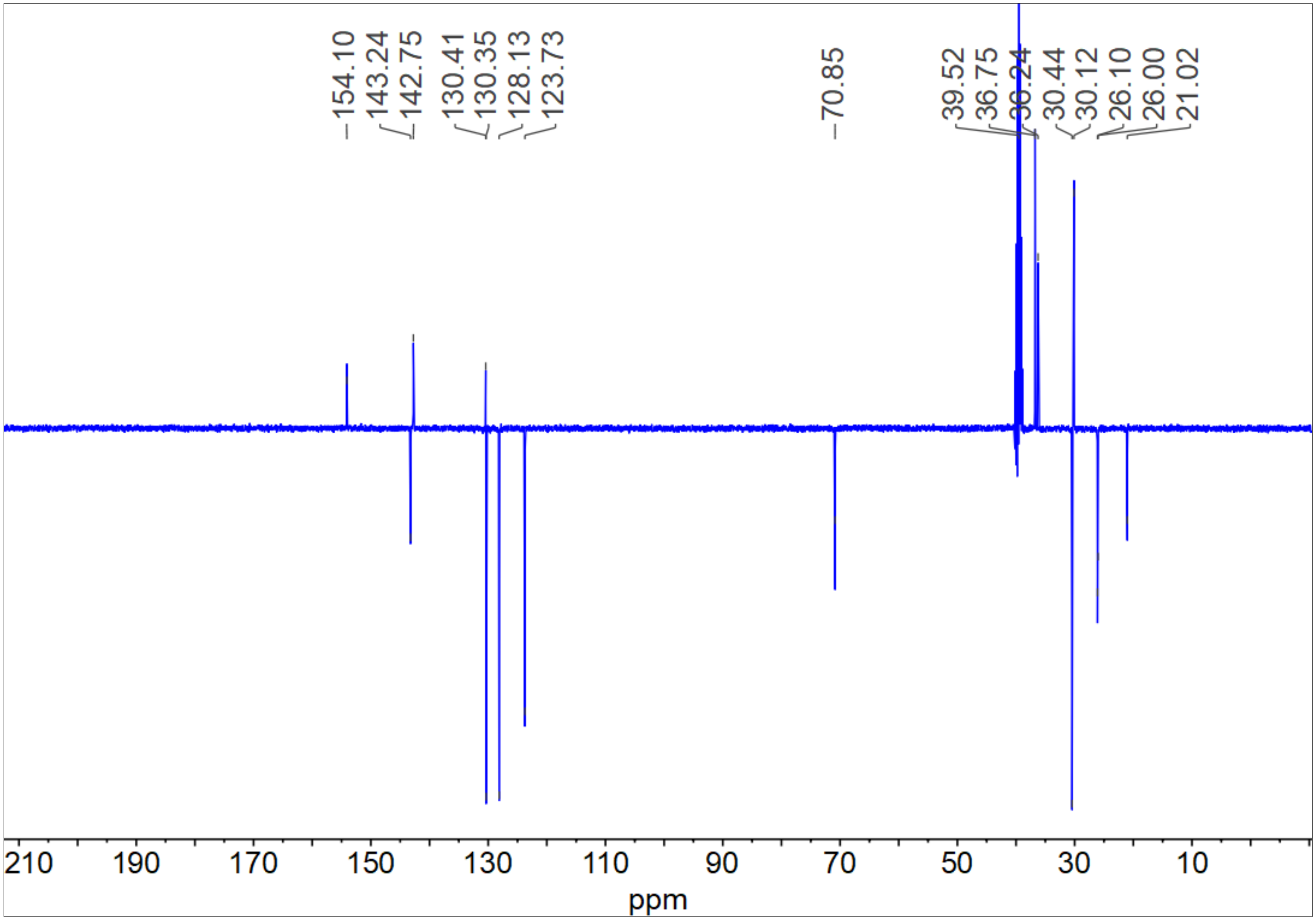

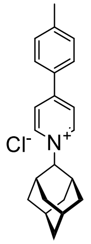

Figure S10. ^{13}C {^{1}H} APT NMR (100 MHz, DMSO-d_6): 1-(Adamantan-2-yl)-4-(4-tolyl)pyridin-1-ium chloride (**2**).

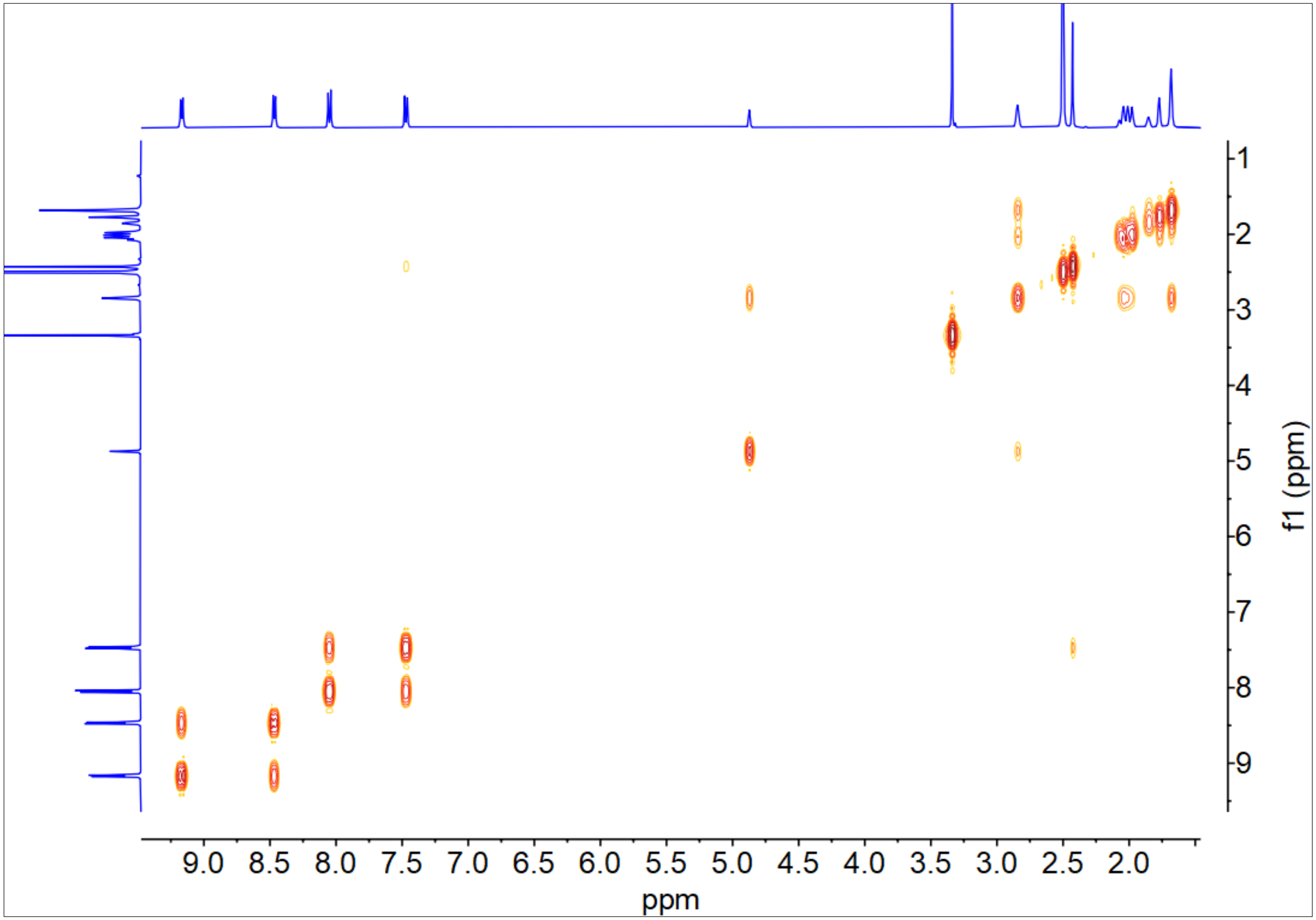

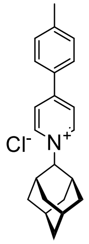

Figure S11. ^{1}H - ^{1}H COSY NMR (DMSO-d_6): 1-(Adamantan-2-yl)-4-(4-tolyl)-pyridin-1-ium chloride (**2**).

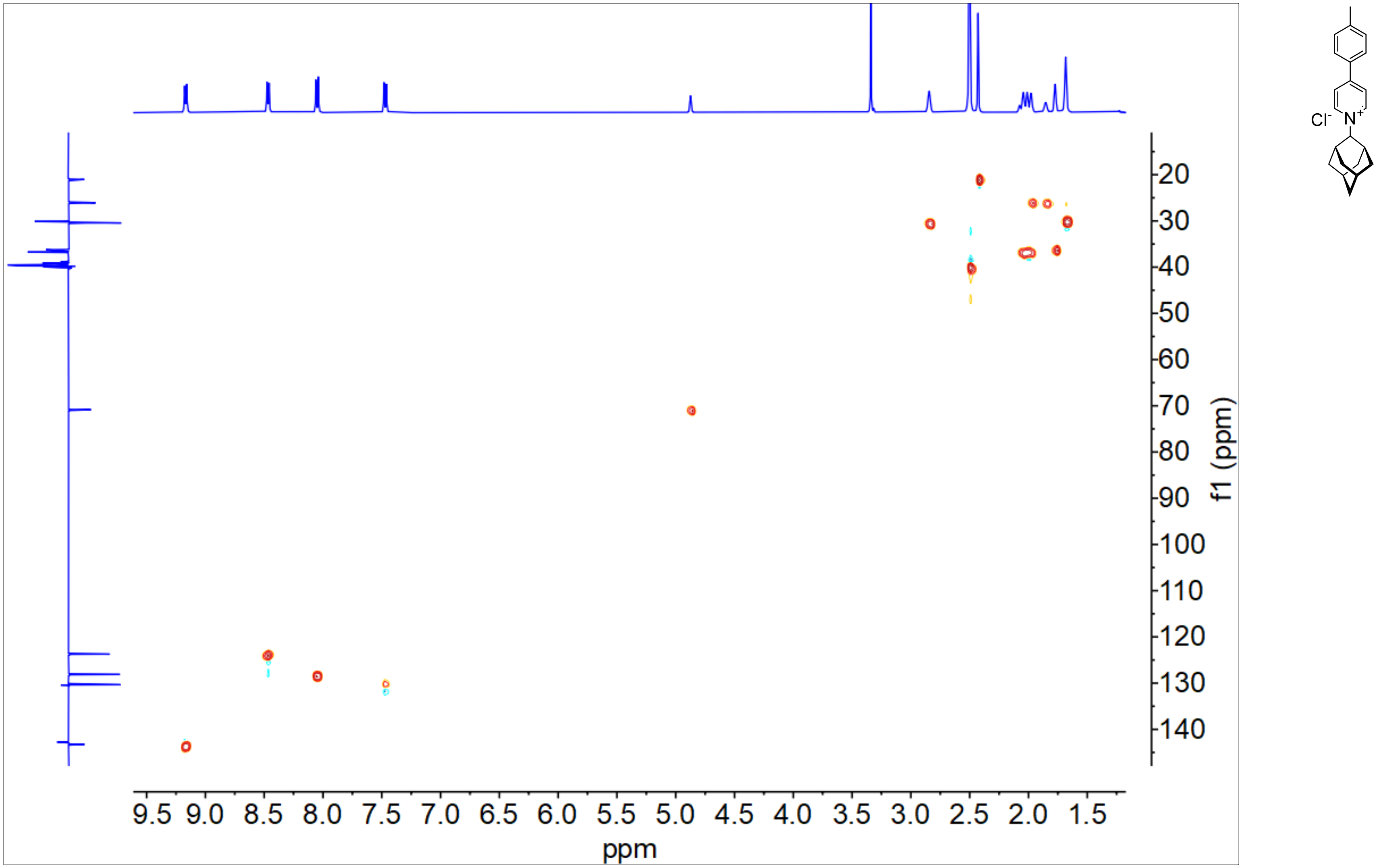

Figure S12. $^{1}H - ^{13}C$ HSQC NMR (DMSO-d_6): 1-(Adamantan-2-yl)-4-(4-tolyl)-pyridin-1-ium chloride (**2**).

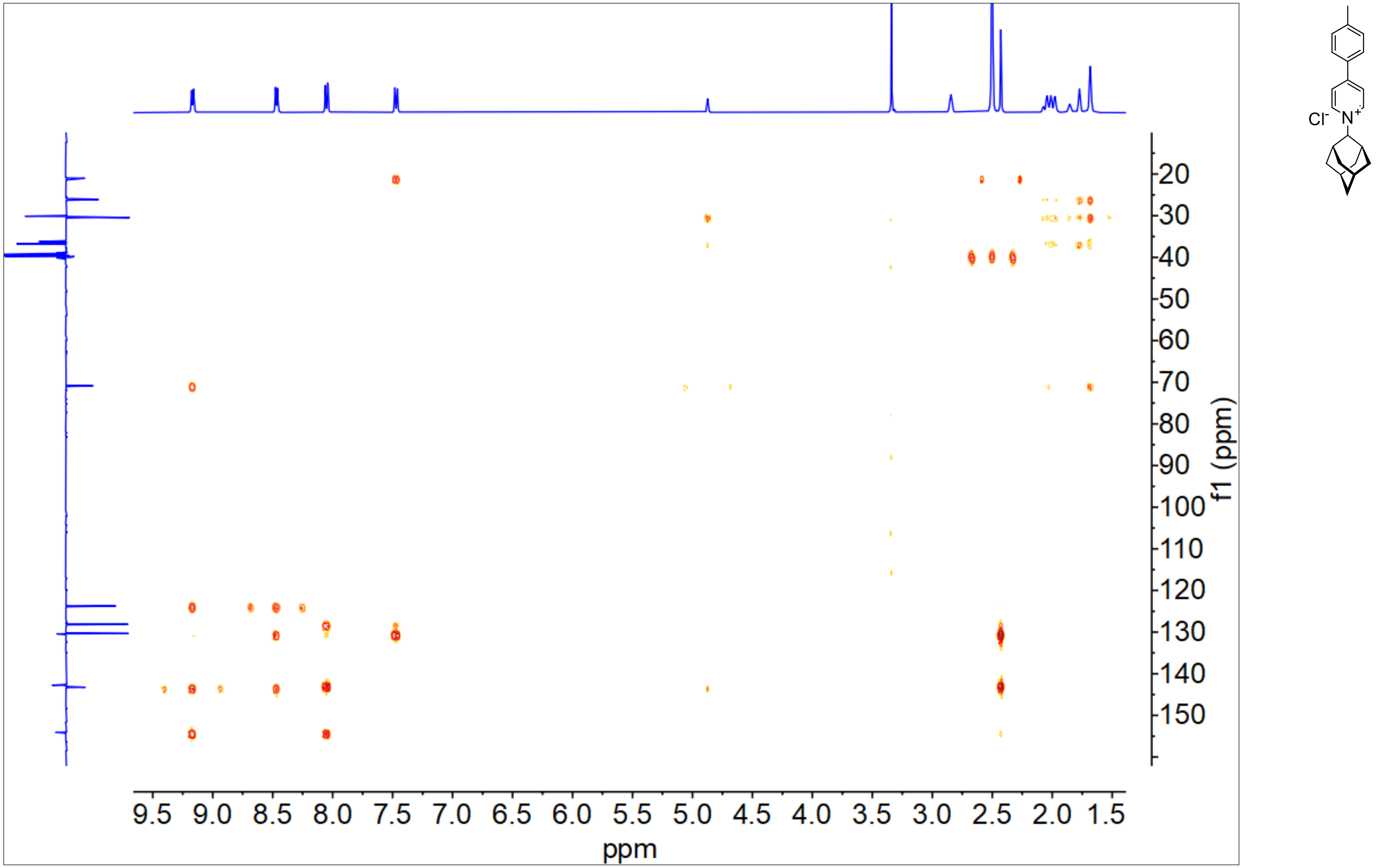

Figure S13. ^{1}H – ^{13}C HMBC NMR (DMSO-d_6): 1-(Adamantan-2-yl)-4-(4-tolyl)-pyridin-1-ium chloride (**2**).

3.3. Compound 3

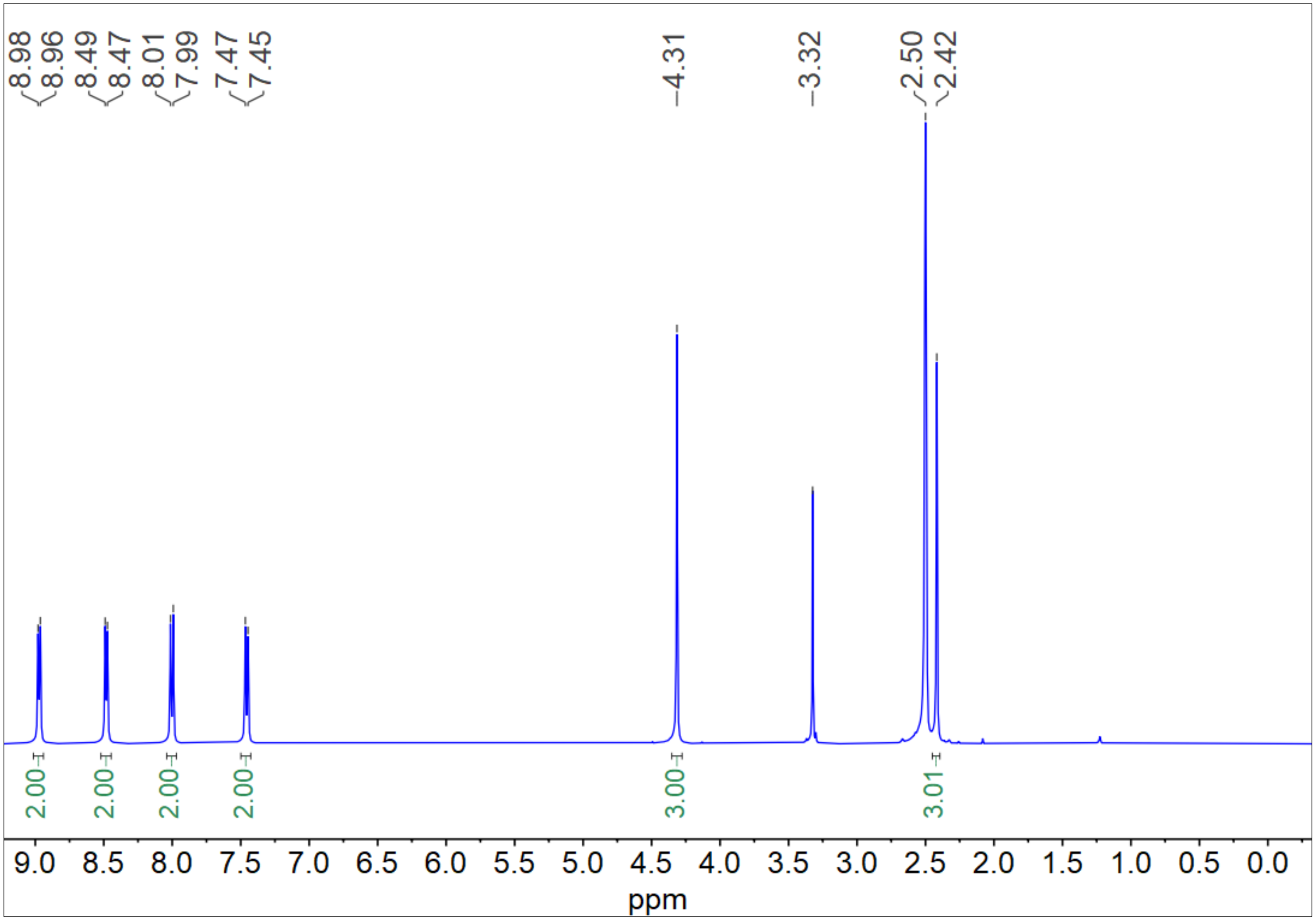

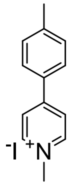

Figure S14. ^{1}H NMR (400 MHz, DMSO-d_6): 1-Methyl-4-(4-tolyl)-pyridin-1-ium iodide (**3**).

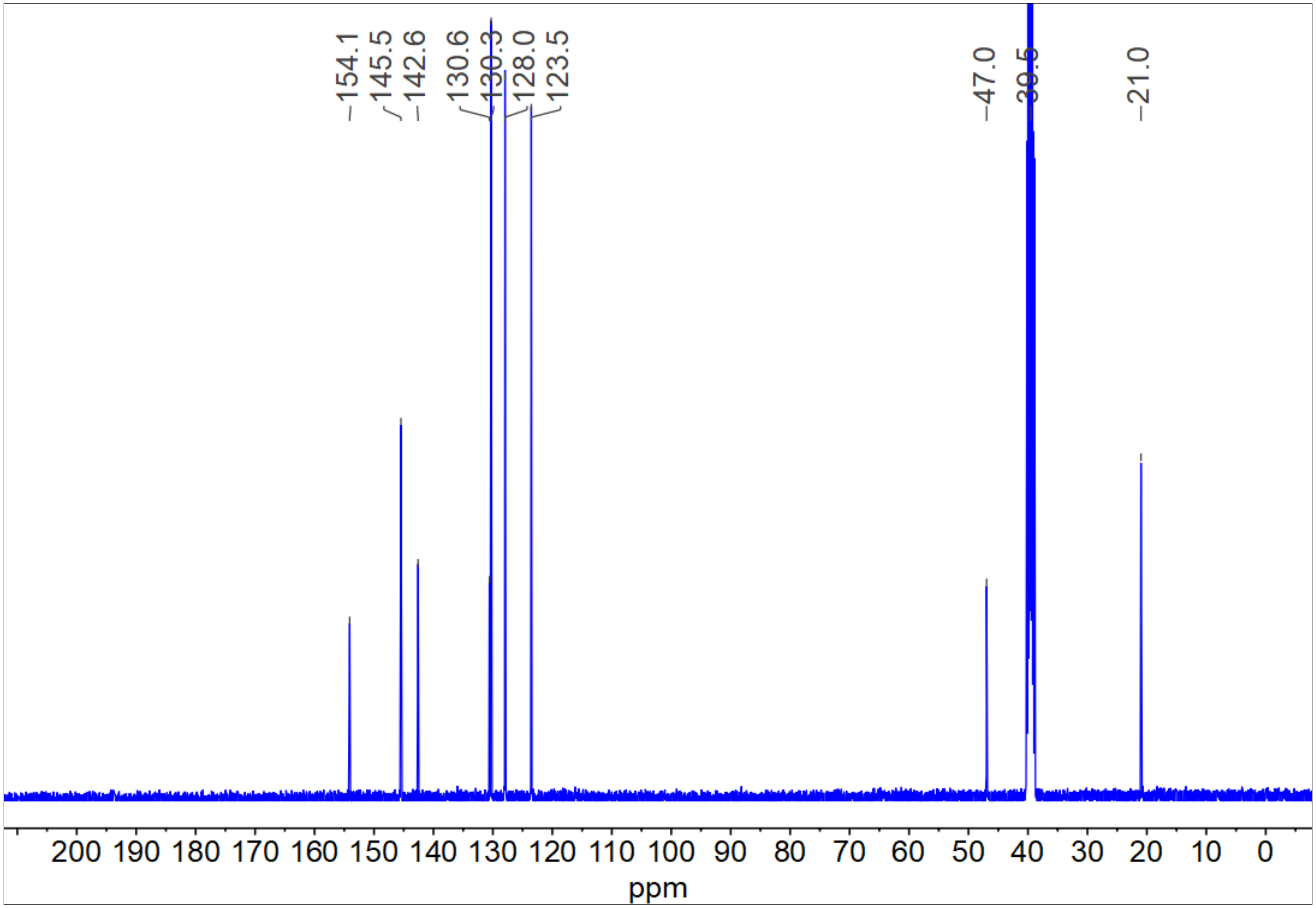

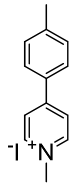

Figure S15. ^{13}C NMR (100 MHz, DMSO-d_6): 1-Methyl-4-(4-tolyl)-pyridin-1-ium iodide (**3**).

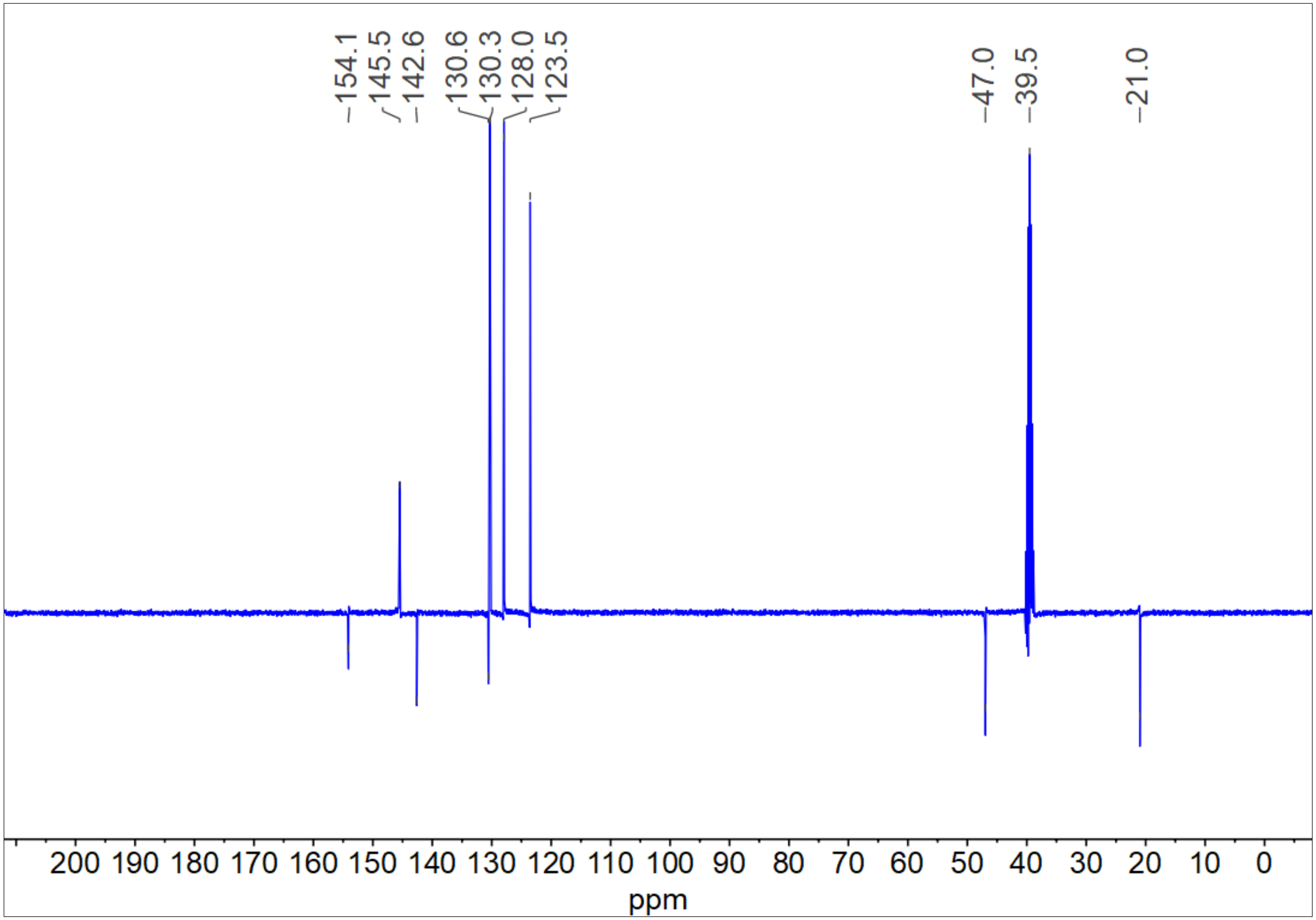

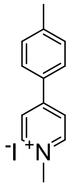

Figure S16. ^{13}C APT NMR (100 MHz, DMSO-d_6): 1-Methyl-4-(4-tolyl)-pyridin-1-ium iodide (**3**).

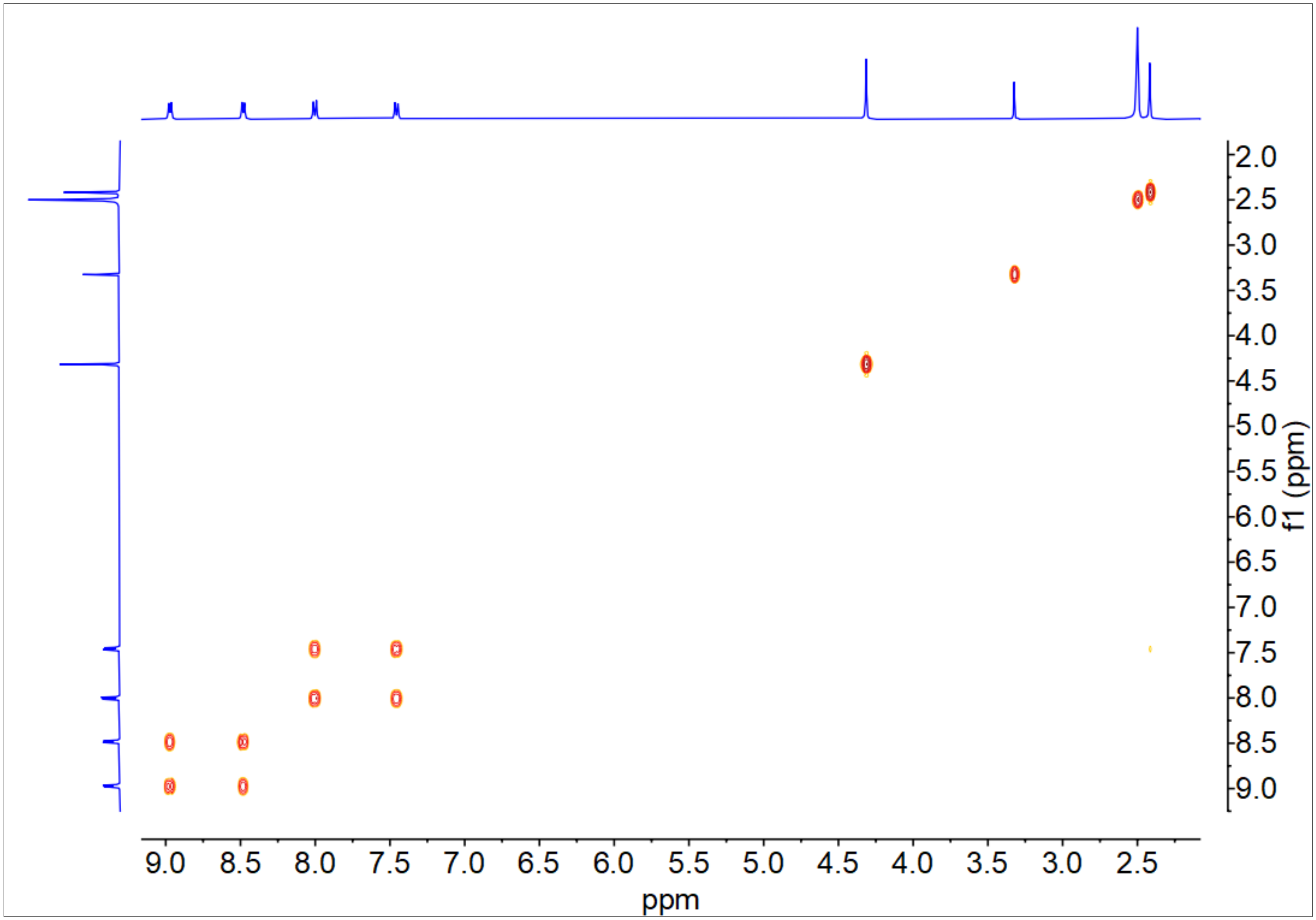

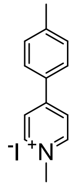

Figure S17. ^{1}H - ^{1}H COSY NMR (DMSO-d_6): 1-Methyl-4-(4-tolyl)-pyridin-1-ium iodide (**3**).

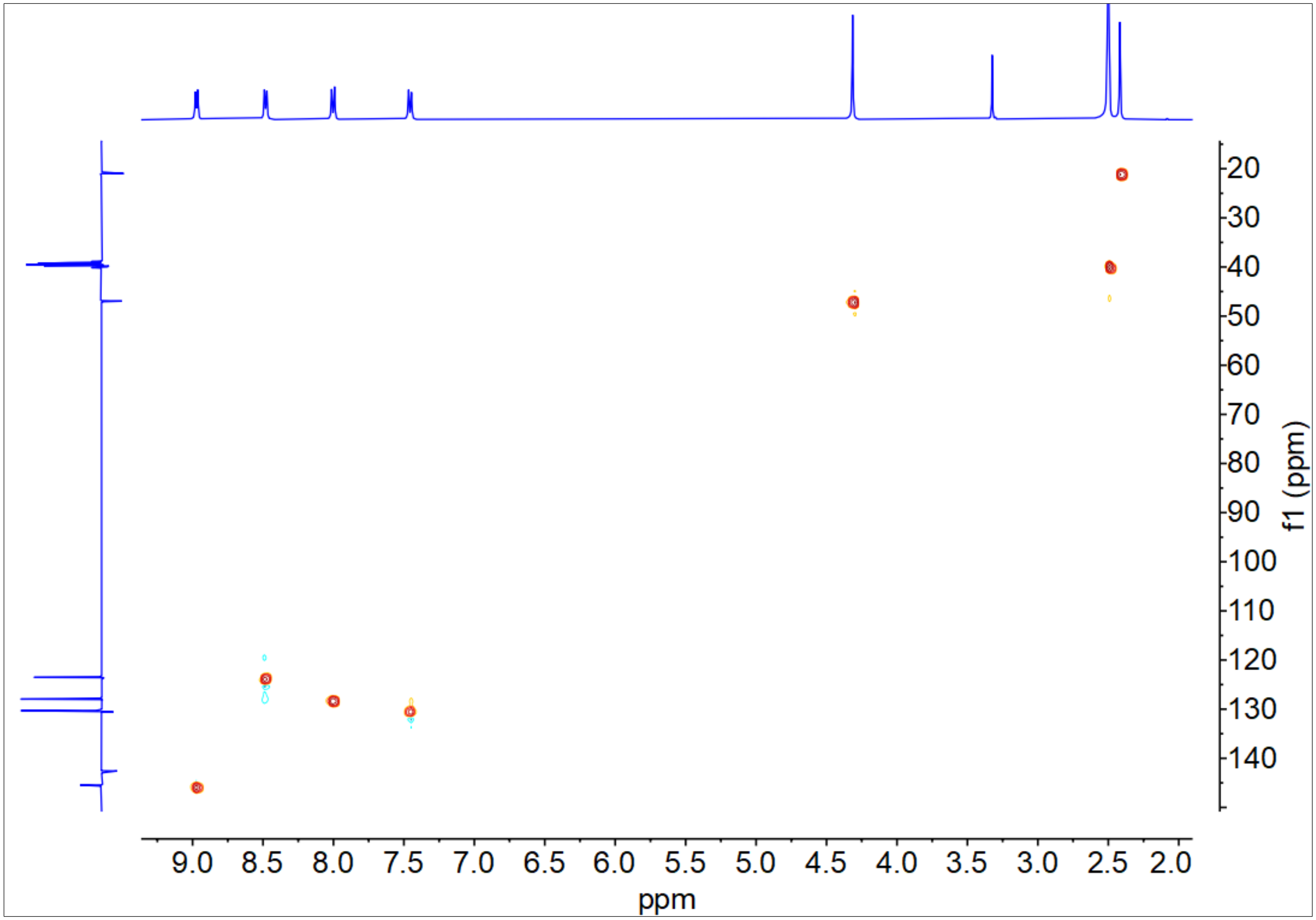

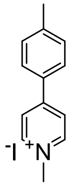

Figure S18. ^{1}H – ^{13}C HSQC NMR (DMSO-d_6): 1-Methyl-4-(4-tolyl)-pyridin-1-ium iodide (**3**).

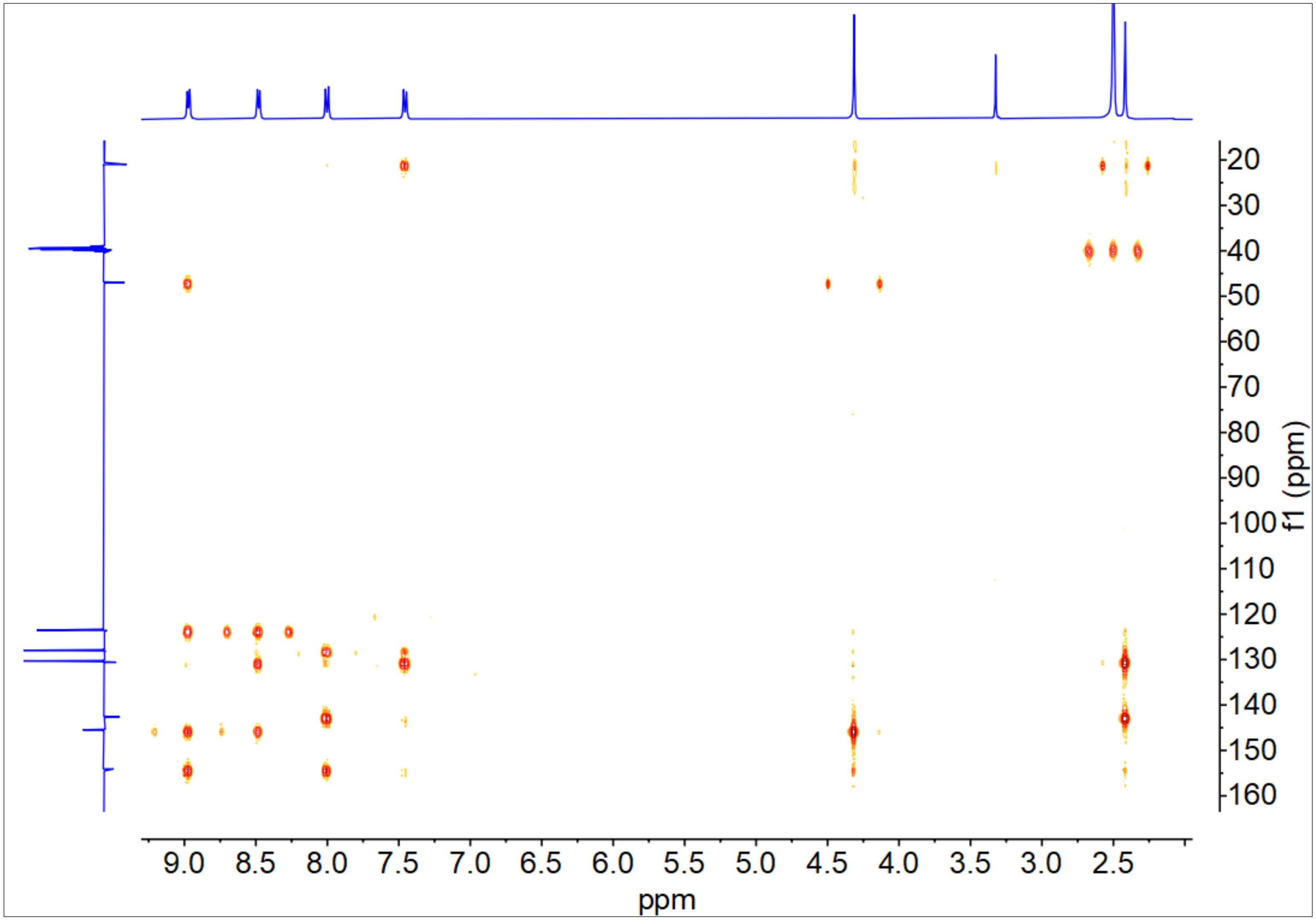

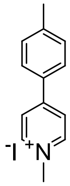

Figure S19 ^{1}H – ^{13}C HMBC NMR (400 MHz, DMSO-d_6): 1-Methyl-4-(4-tolyl)-pyridin-1-ium iodide (**3**).

3.3. Compound 4

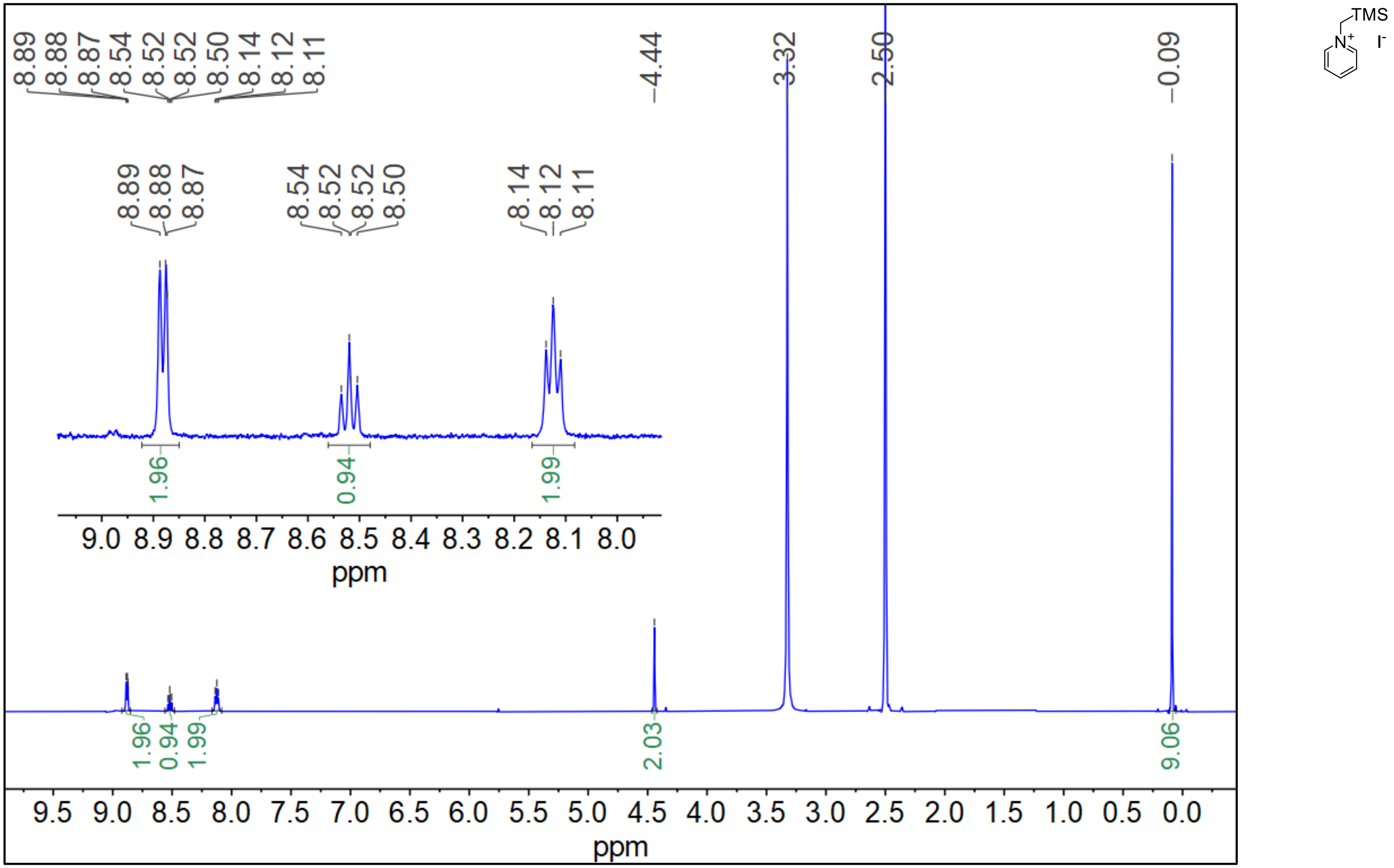

Figure S20. ^{1}H NMR (500 MHz, DMSO-d_6): (Trimethylsilylmethyl)-pyridin-1-ium iodide (**4**).

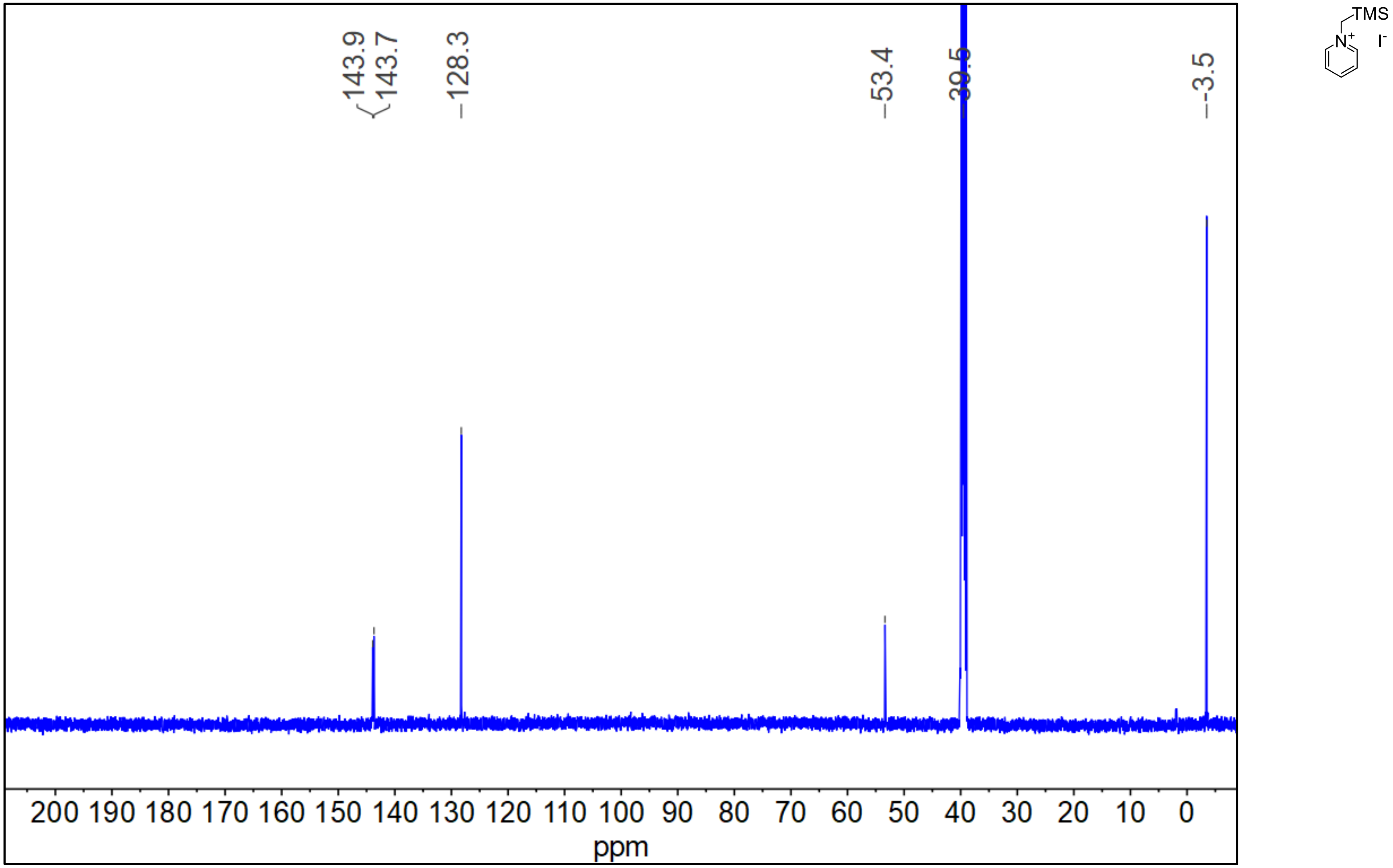

Figure S21. ^{13}C NMR (126 MHz, DMSO-d_6): (Trimethylsilylmethyl)-pyridin-1-ium iodide (**4**).

3.4. Compound 5

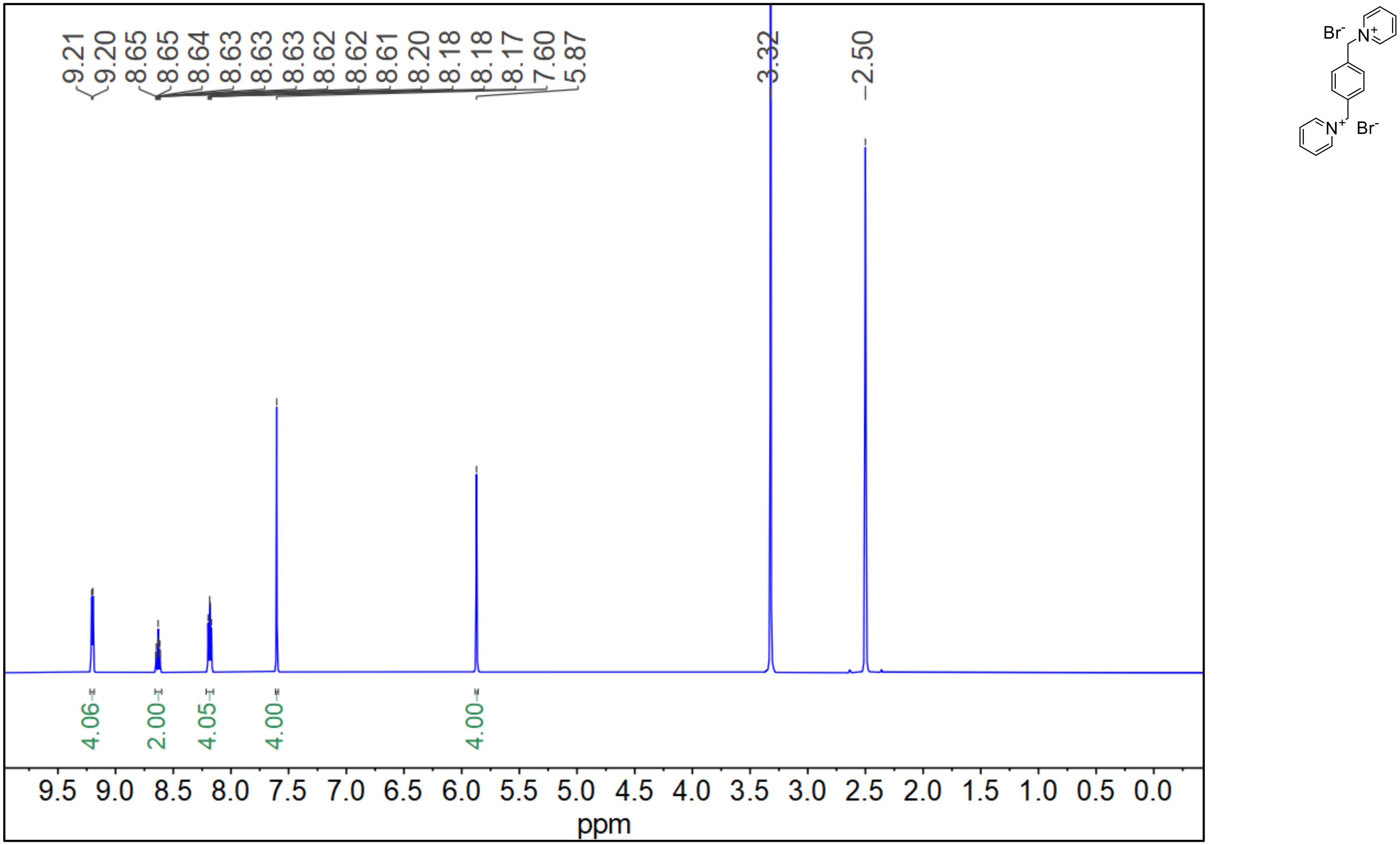

Figure S22. ^{1}H NMR (500 MHz, DMSO-d_6): 1,1'-[1,4-Phenylenebis(methylene)]bis(pyridin-1-ium**)** bromide (**5**).

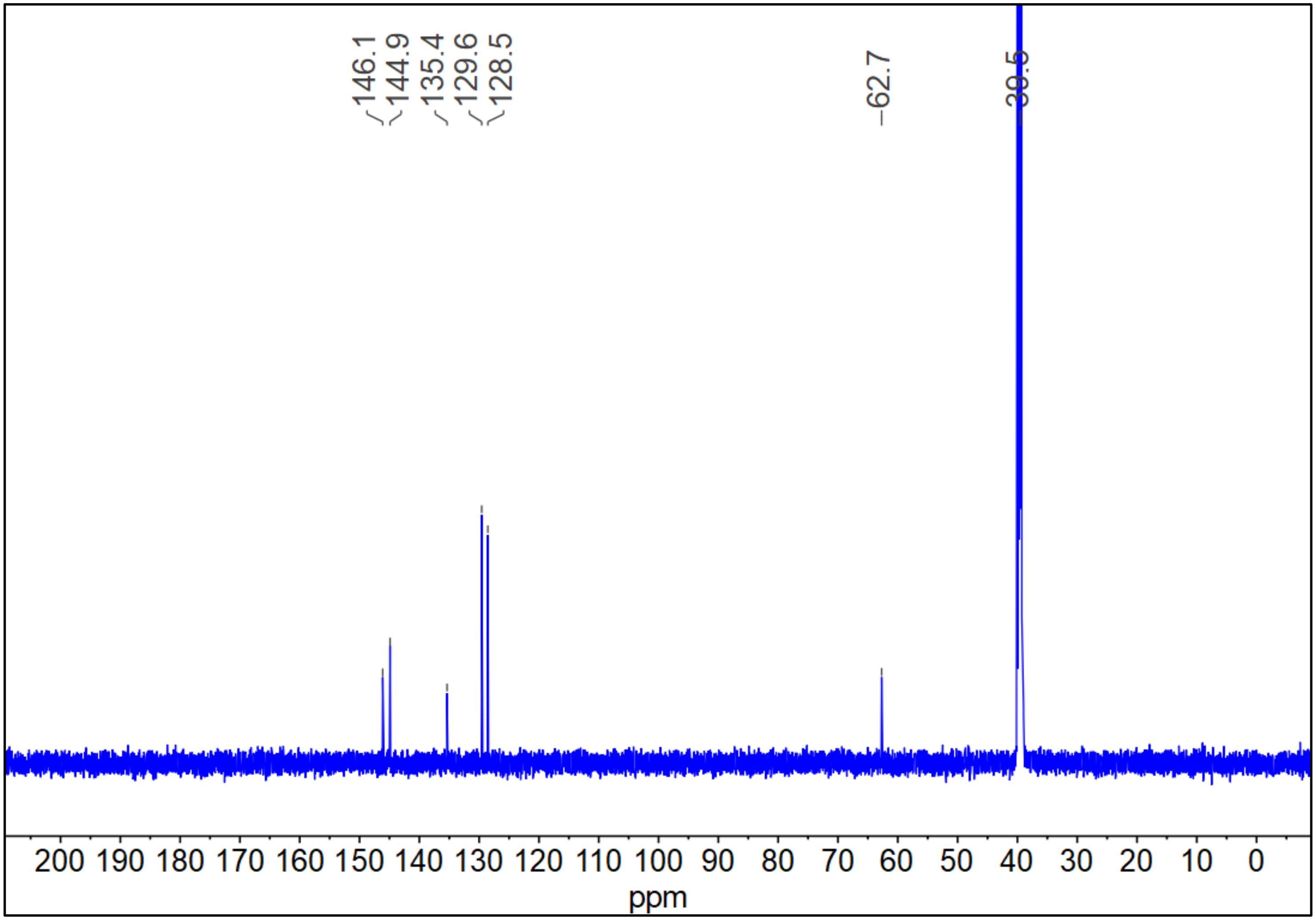

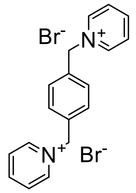

Figure S23. ^{13}C {^{1}H} NMR (126 MHz, DMSO-d_6): 1,1'-[1,4-Phenylenebis(methylene)]bis(pyridin-1-ium) bromide (**5**).

3. Preparation of CB[7]·1 and [(CB[7]·1)$_3$·Na]Cl

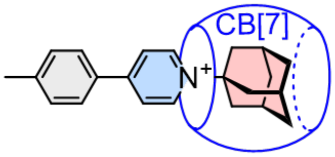

Guest **1** (6.3 mg, 0.02 mmol) was dissolved in deionized water (5 mL) in a round bottom flask. A solution of cucurbit[7]uril (1 mmol) in deionized water (28 mL) was prepared in a separate flask. One equivalent of CB[7]solution was added portion-wise to the solution of guest, while stirring with magnetic stir bar at room temperature. Water was removed under reduced pressure and the resulting solid was dried on a Kugelrohr distillation apparatus (60 °C, 750 mTorr) yielding **CB[7]·1** as a white crystalline solid (30 mg, 0.2 mmol).
CB[7]·1: ^{1}H NMR (400 MHz, D_2O): δ 1.05 (d, J = 12.5 Hz, 3H), 1.33 (d, J = 12.7 Hz, 3H), 1.55-1.57 (m, 6H), 1.63 (bs, 3H), 2.46 (s, 3H), 4.24 (d, J = 15.5 Hz, 14H), 5.54 (s, 14H), 5.74 (d, J = 15.3 Hz, 7H), 5.80 (d, J = 15.4 Hz, 7H), 7.52 (d, J = 7.9 Hz, 2H), 8.00 (d, J = 8.2 Hz, 2H), 8.33 (d, J = 7.2 Hz, 2H), 8.90 (d, J = 7.1 Hz, 2H). ^{1}H NMR (400 MHz, DMSO-d_6): δ 0.90-1.00 (m, 6H), 1.41 (bs, 6H), 1.50 (bs, 3H), 2.44 (s, 3H), 4.18 (m, 14H), 5.39 (bs, 14H), 5.62 (d, J = 14.6 Hz, 7H), 5.68 (d, J = 14.7 Hz, 7H), 7.46 (d, J = 8.44 Hz, 2H), 8.10 (d, J = 8.15 Hz, 2H), 8.18 (d, J = 7.4 Hz, 2H), 8.98 (d, J = 7.3 Hz, 2H).

Acquiring the ^{1}H NMR spectrum in DMSO-d_6 revealed that 10-20% of **CB[7]·1** was already assembled to **[(CB[7]·1)$_3$·Na]Cl** (Figure S18). The NMR titration using Na^+ salt in DMSO-d_6 converted **CB[7]·1** nearly quantitatively to **[(CB[7]·1)$_3$·Na]Cl** (Figure S19).
[(CB[7]·1)$_3$·Na]Cl: ^{1}H NMR (400 MHz, DMSO-d_6): δ 0.89 (d, J = 11.9 Hz, 3H), 1.03 (d, J = 11.2 Hz, 3H), 1.49 (bs, 6H), 1.55 (bs, 3H), 2.45 (s, 3H), 4.28 (d, J_1 = 14 Hz, 7H), 4.41 (d, J_1 = 14 Hz, 7H), 5.70-5.62 (m, 28H), 7.48 (d, J = 8.20 Hz, 2H), 8.13 (d, J = 8.15 Hz, 2H), 8.24 (d, J = 6.6 Hz, 2H), 9.03 (d, J = 7.0 Hz, 2H).

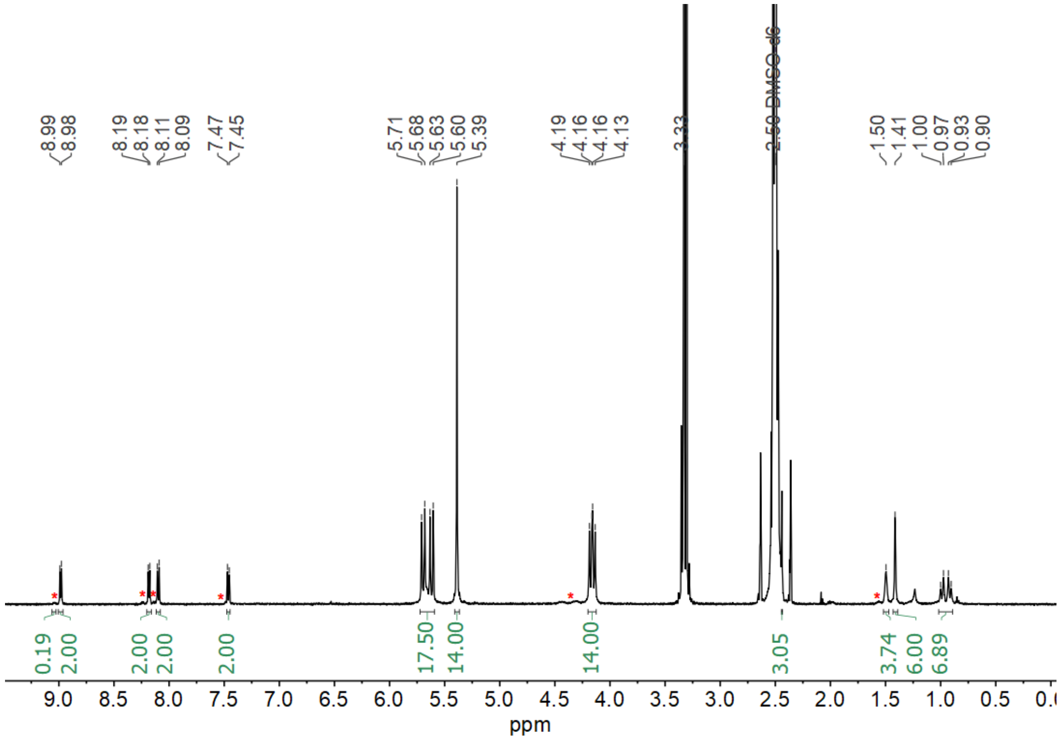

Figure S24. ^{1}H NMR spectrum of **CB[7]·1** complex in DMSO-d_6 at 25 °C. The red asterisks indicate presence of **[(CB[7]·1)$_3$·Na]Cl**, where signals are visible

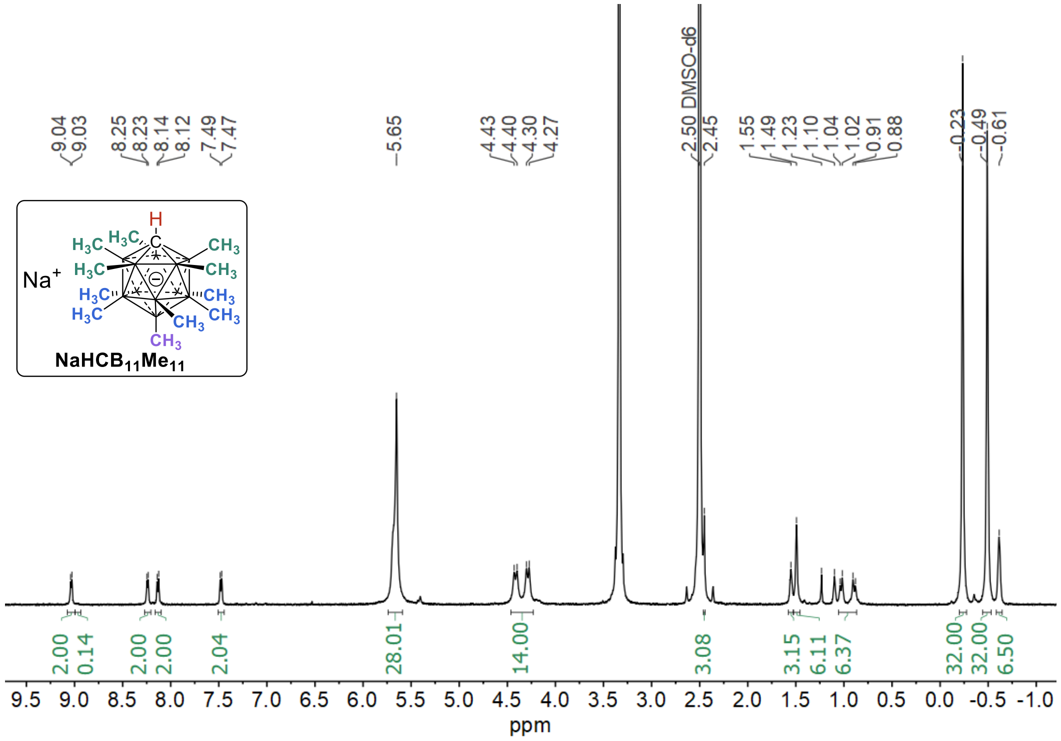

Figure S25. ^{1}H NMR of **[(CB[7]·1)$_3$·Na]HCB$_{11}$Me$_{11}$**, which was prepared by mixing **CB[7]·1** with 2 equivalents of NaHCB$_{11}$Me$_{11}$ in DMSO-d_6 at 25 °C. Signals below 0 ppm, as well as signal at 1.10 ppm, belong to the methyls of carborane anion.

4. ^{1}H NMR Titration Experiments

4.1. Titrations of guests with CB[7]

^{1}H NMR titrations of guests with **CB[7]** were performed by step-wise addition of 1 equivalent of saturated **CB[7]** solution (c = 0.70 mmol L^{-1} in DMSO-d_6,) to guest solution in NMR tube by either micropipette or a Hamilton syringe at 25 °C.

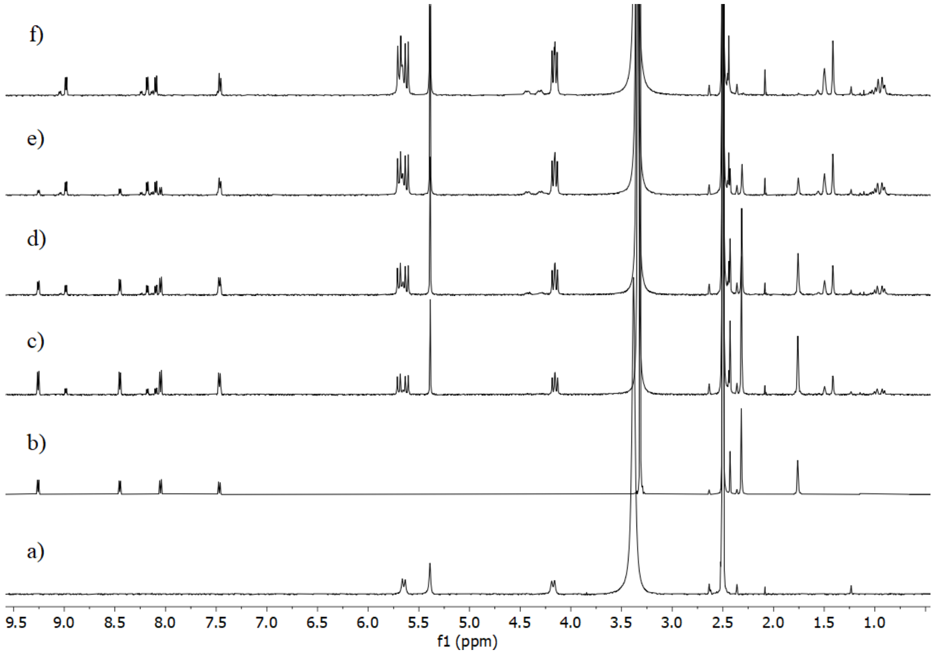

Figure S26. ^{1}H NMR spectra of: a) neat **CB[7]**; b) neat guest **1** (c = 1 mmol L^{-1}) before and after addition of c) 0.25; d) 0.5; e) 0.75 and f) 1.0 equivalent of **CB[7]** in DMSO-d_6.

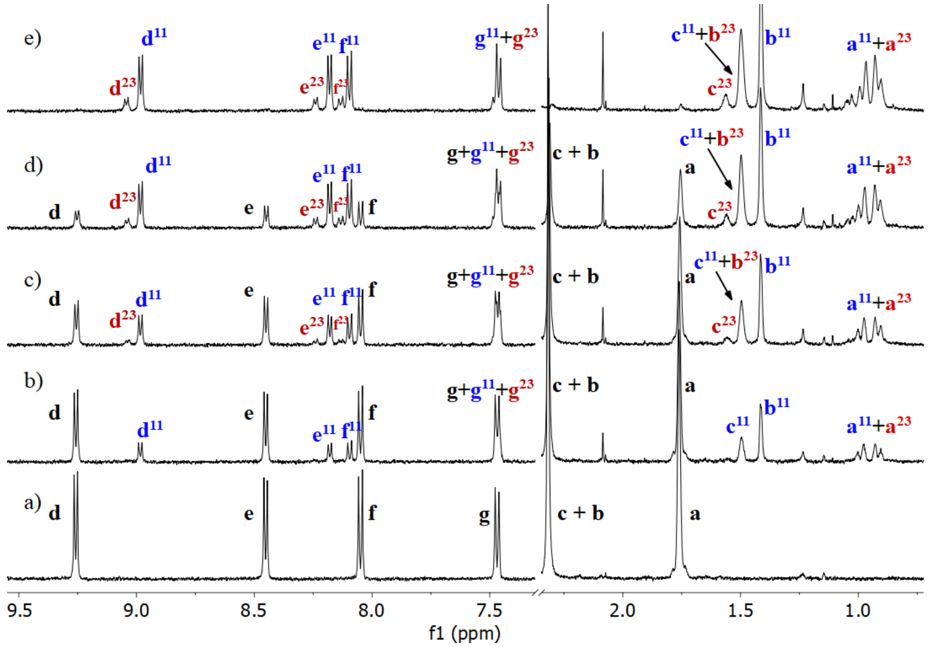

Figure S27. Aromatic and aliphatic regions of ^{1}H NMR spectra of: a) neat guest **1** (c = 1 mmol L^{-1}) before and after addition of b) 0.25; c) 0.5; d) 0.75 and e) 1.0 equivalent of **CB[7]** in DMSO-d_6.

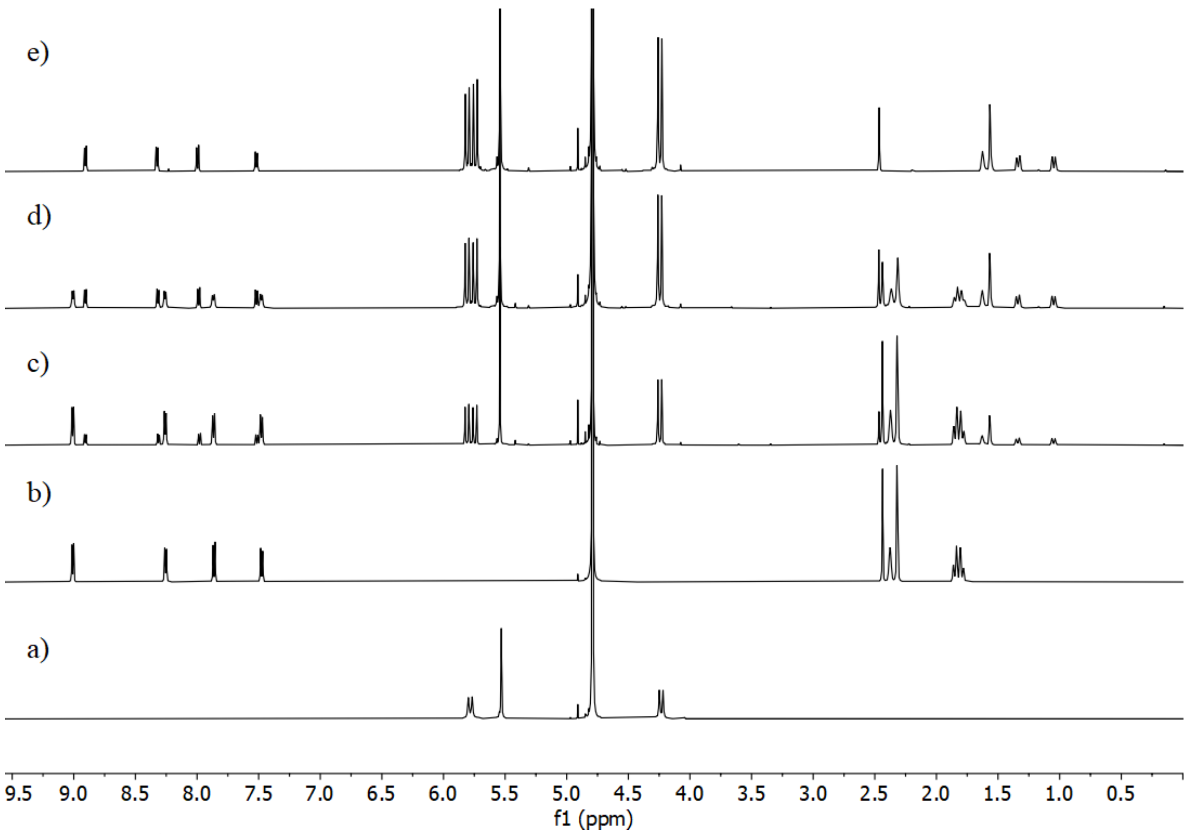

Figure S28. ^{1}H NMR spectra of: a) neat **CB[7]**; b) solution of guest **1** (c = 7.6 mmol L^{-1}) before and after addition of c) 0.2; d) 0.4 and e) 1.0 equivalent of **CB[7]** in D_2O.

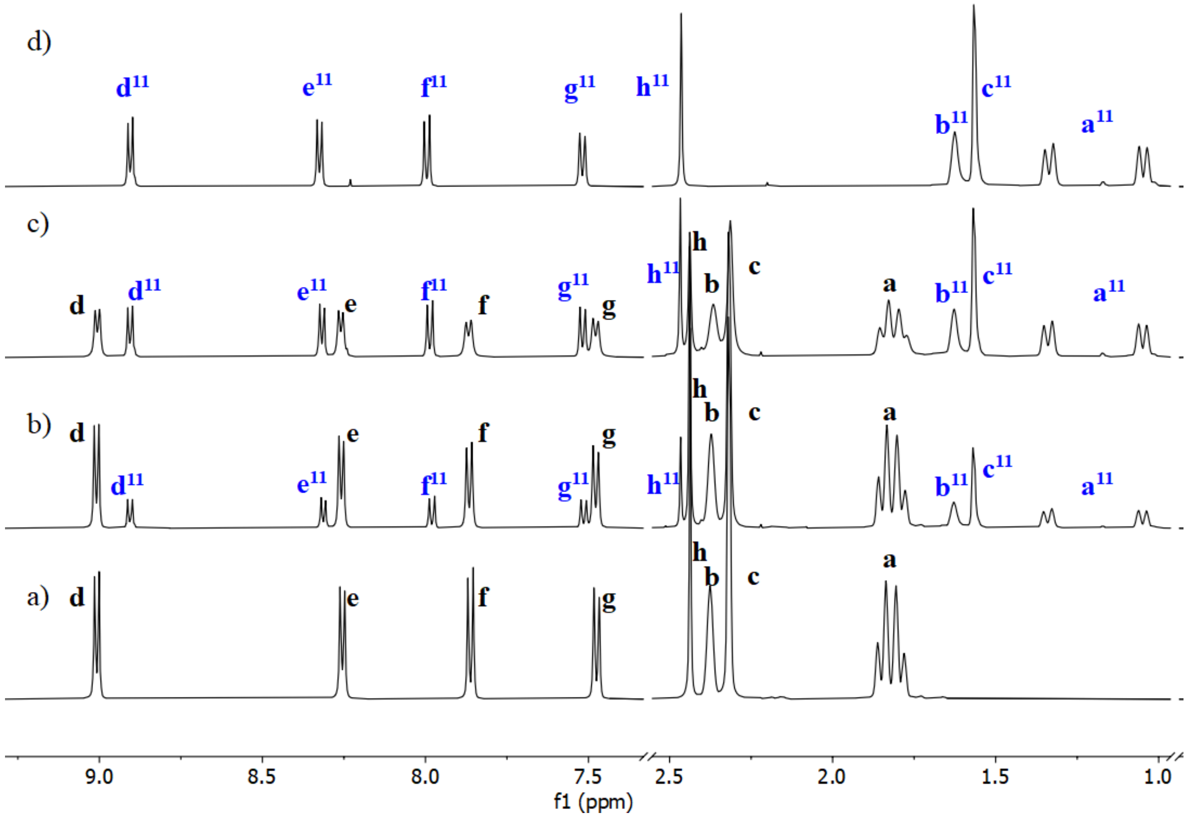

Figure S29. Aromatic and aliphatic regions of ^{1}H NMR spectra of: a) neat guest **1** (c = 7.6 mmol L^{-1}) before and after addition of c) 0.2; d) 0.4 and e) 1.0 equivalent of **CB[7]** in D_2O.

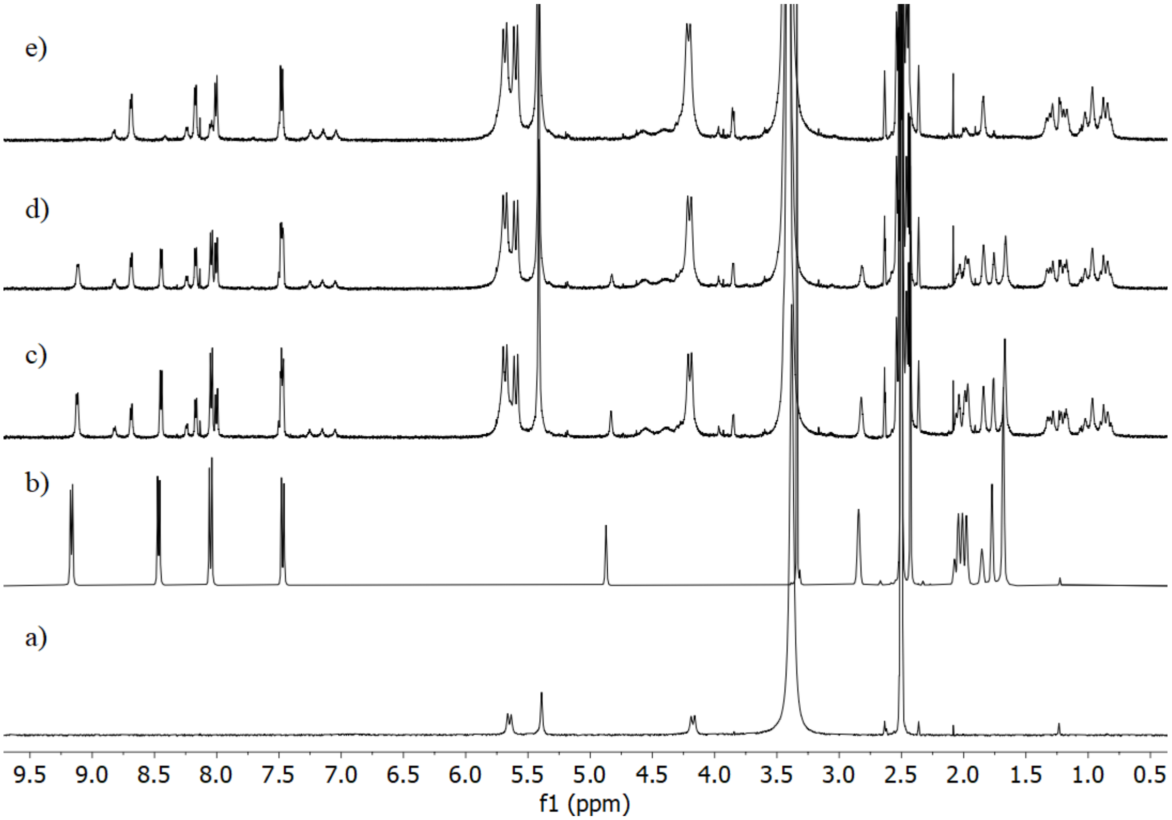

Figure S30. ^{1}H NMR spectra of: a) neat **CB[7]**; b) neat guest **2** (c = 5.5 mmol L^{-1}) before and after addition of c) 0.3; d) 0.6; e) 0.8 and f) 1.3 equivalent of **CB[7]** in DMSO-d_6.

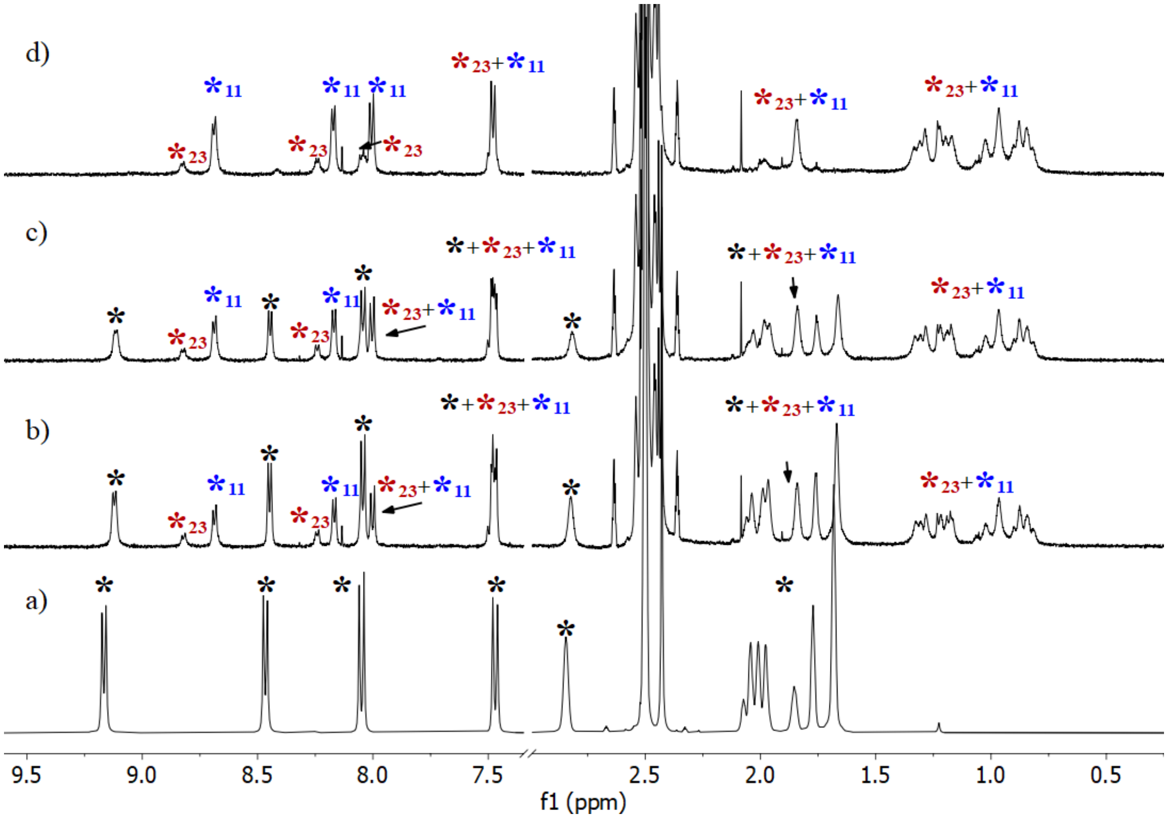

Figure S31. Aromatic and aliphatic regions of ^{1}H NMR spectra of: a) neat guest **2** (c = 5.5 mmol L^{-1}) before and after addition of b) 0.3; c) 0.6; d) 0.8 and e) 1.3 equivalent of **CB[7]** in DMSO-d_6.

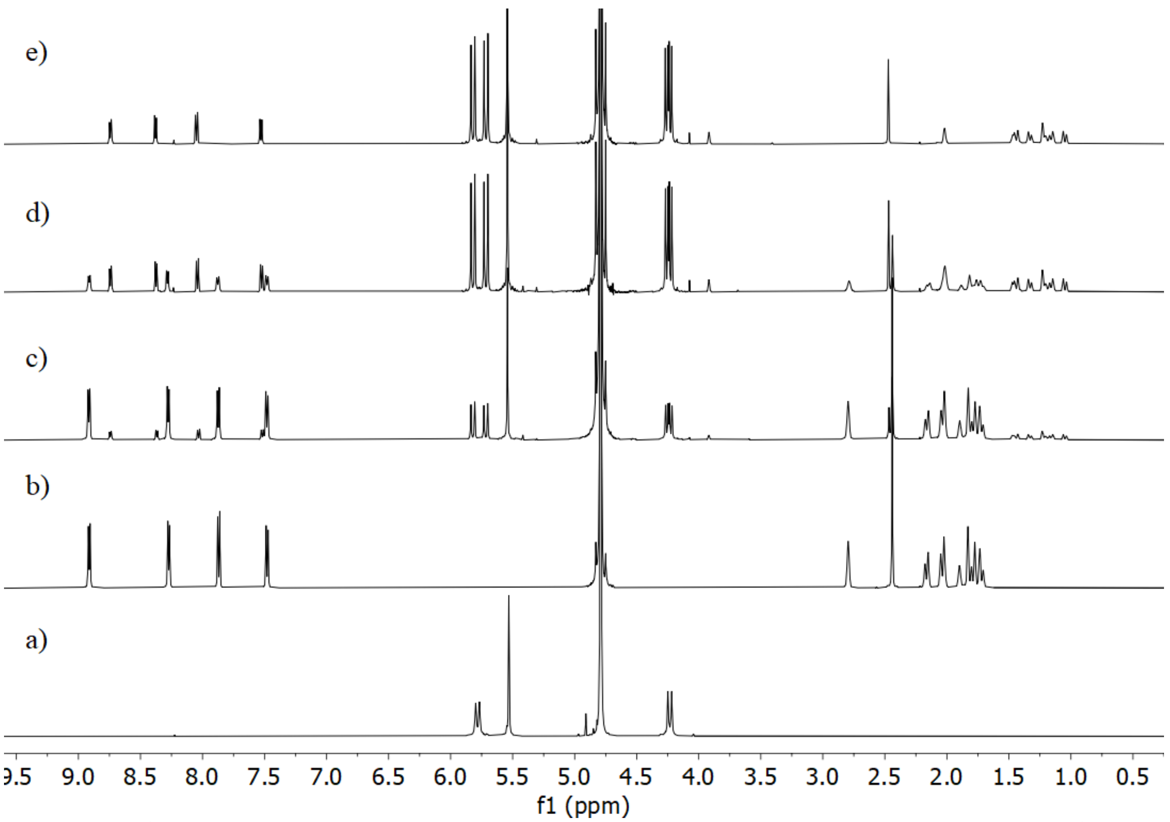

Figure S32. ¹H NMR spectra of: a) neat **CB[7]**; b) solution of guest **2** (c = 6.6 mmol L^{-1}) before and after addition of c) 0.2; d) 0.6 and e) 1.1 equivalent of **CB[7]** solution in D_2O.

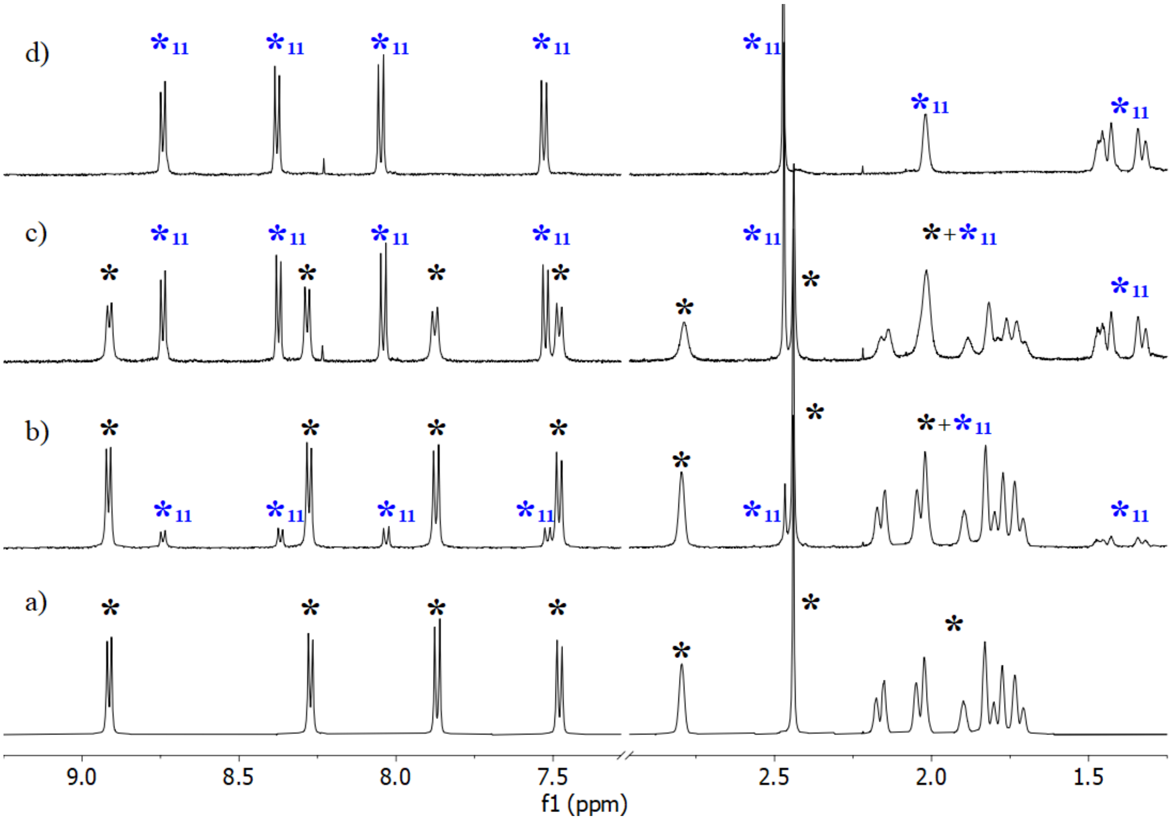

Figure S33. Aromatic and aliphatic regions of ¹H NMR spectra of: a) neat guest **2** (c = 6.6 mmol L^{-1}) before; and after addition of c) 0.2; d) 0.6 and e) 1.1 equivalent of **CB[7]** solution in D_2O.

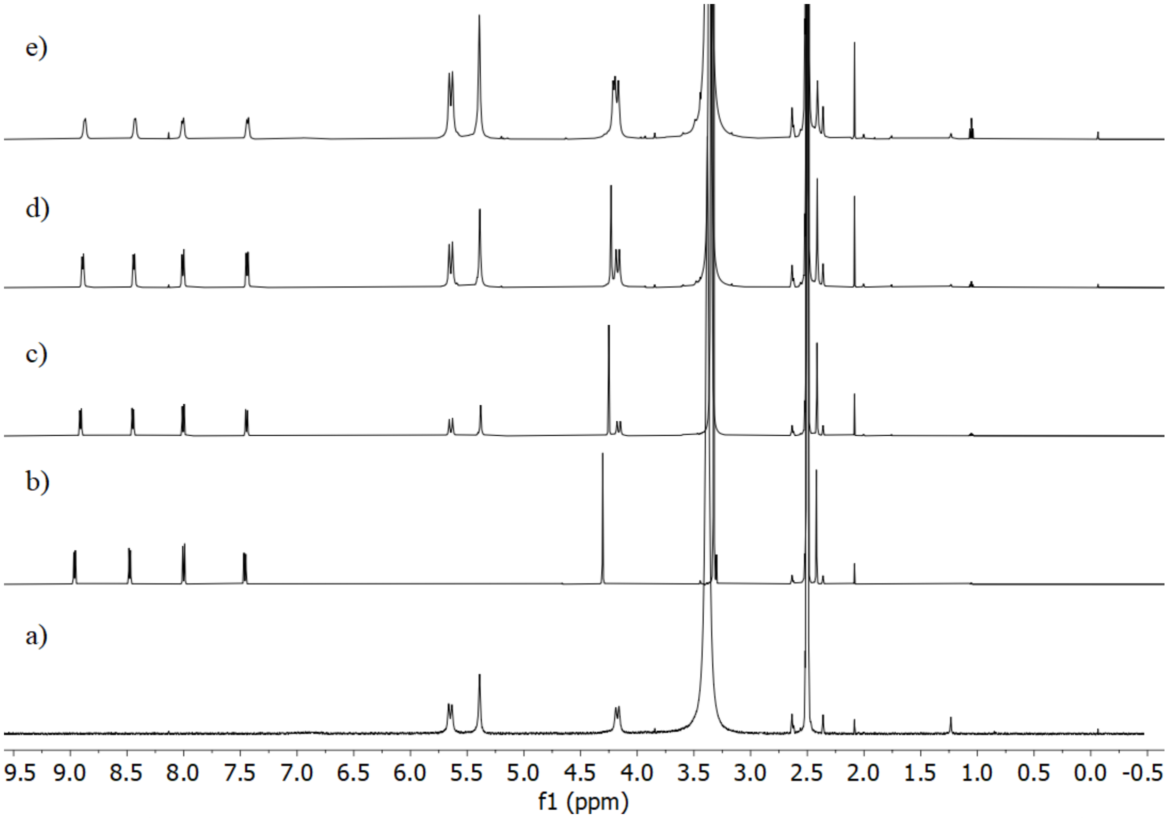

Figure S34. ^{1}H NMR spectra of: a) neat **CB[7]**; b) solution of guest **3** (c = 5.4 mmol L^{-1}) before and after addition of c) 0.2; d) 0.4 and e) 1 equivalent of **CB[7]** solution in DMSO-d_6.

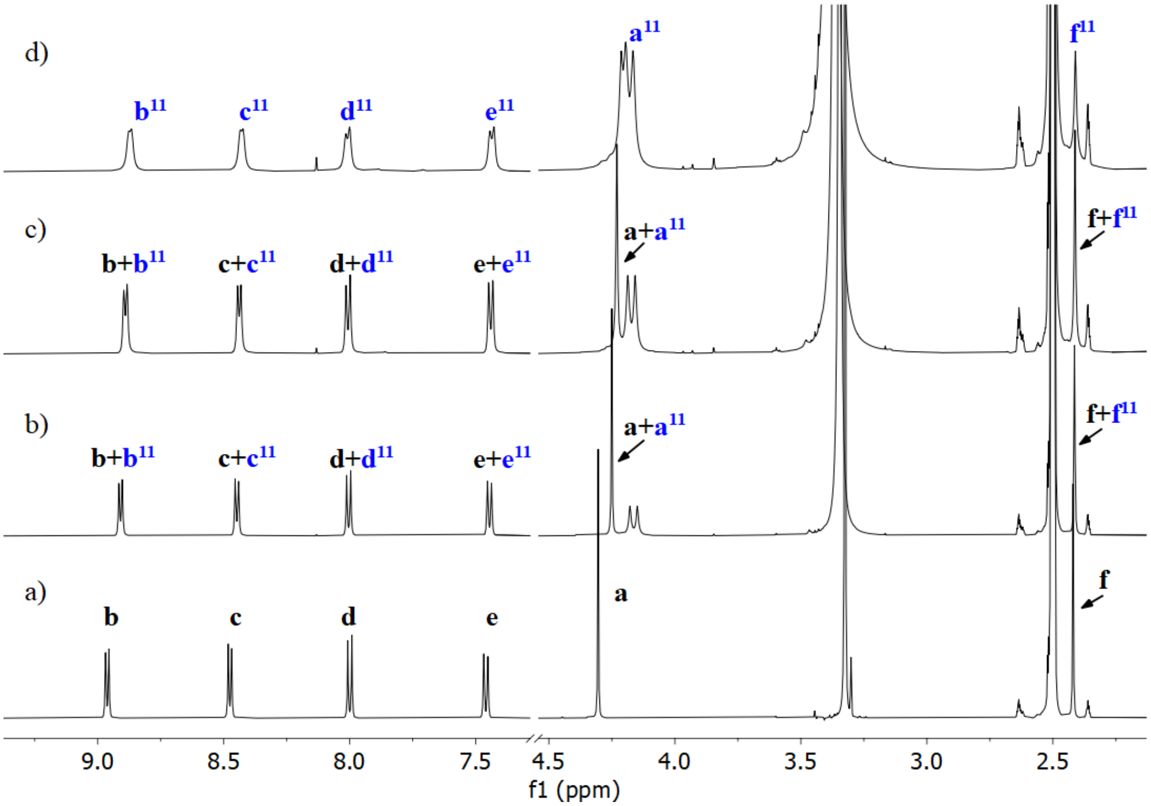

Figure S35. Aromatic and aliphatic regions of ^{1}H NMR spectra of: a) neat guest **3** (c = 5.4 mmol L^{-1}) before and after addition of b) 0.2; c) 0.4; and d) 1 equivalent of **CB[7]** in DMSO-d_6.

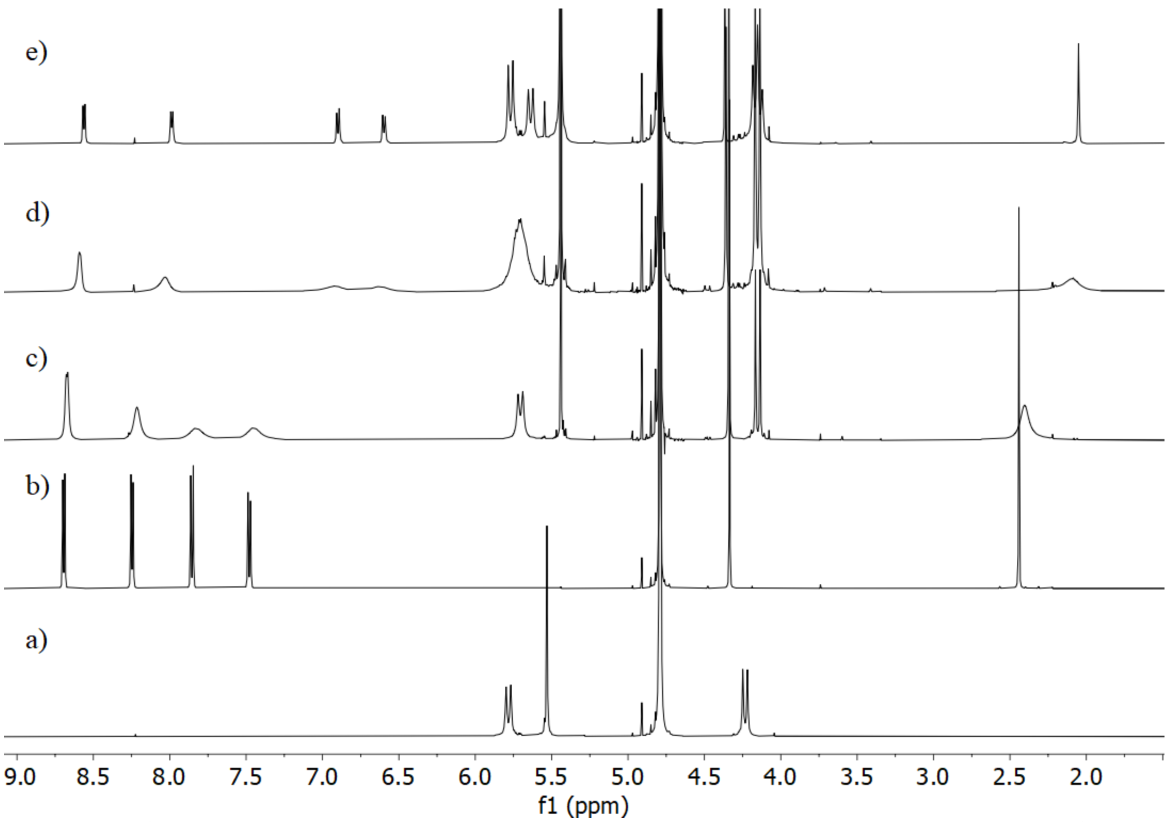

Figure S36. ¹H NMR spectra of: a) neat **CB[7]**; b) solution of guest **3** (c = 4.4 mmol L^{-1}) before and after addition of c) 0.2; d) 0.8 and e) 1.2 equivalent of **CB[7]** solution in D_2O.

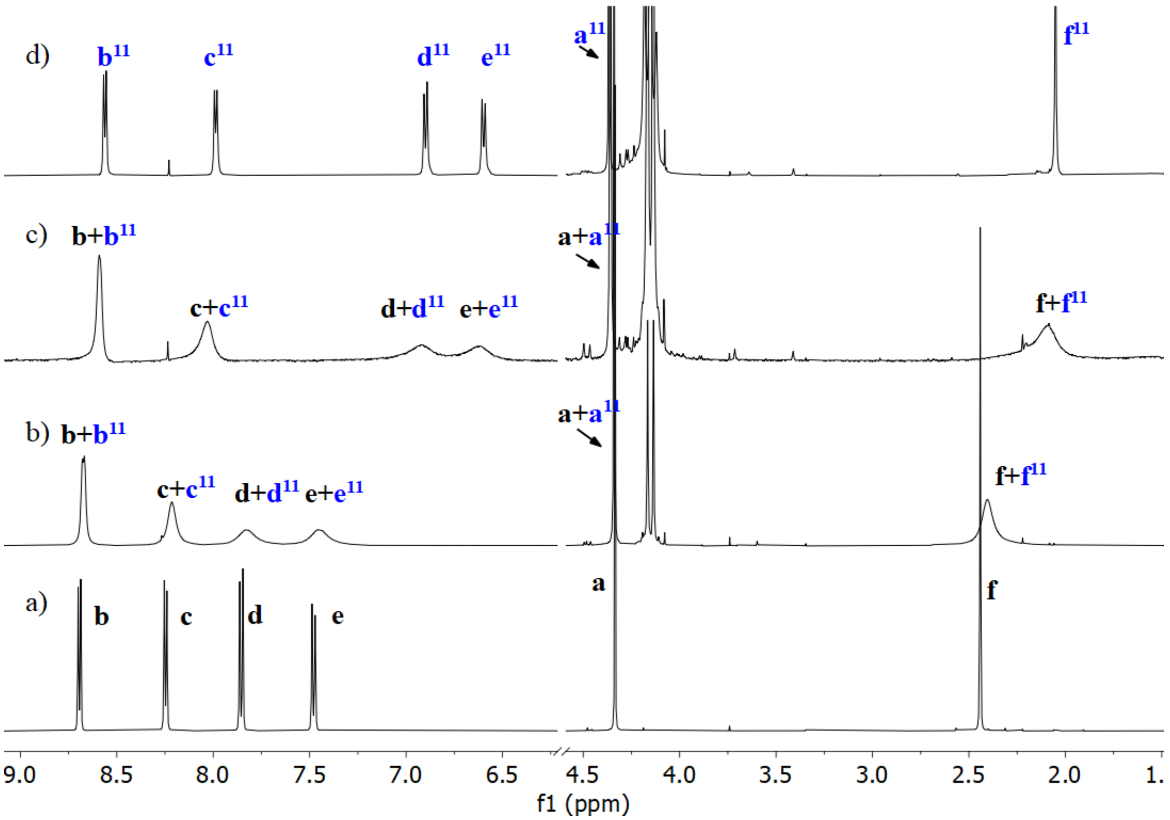

Figure S37. Aromatic and aliphatic regions of ¹H NMR spectra of: a) neat guest **3** (c = 4.4 mmol L^{-1}) before; and after addition of c) 0.2; d) 0.8 and e) 1.2 equivalent of **CB[7]** solution in D_2O.

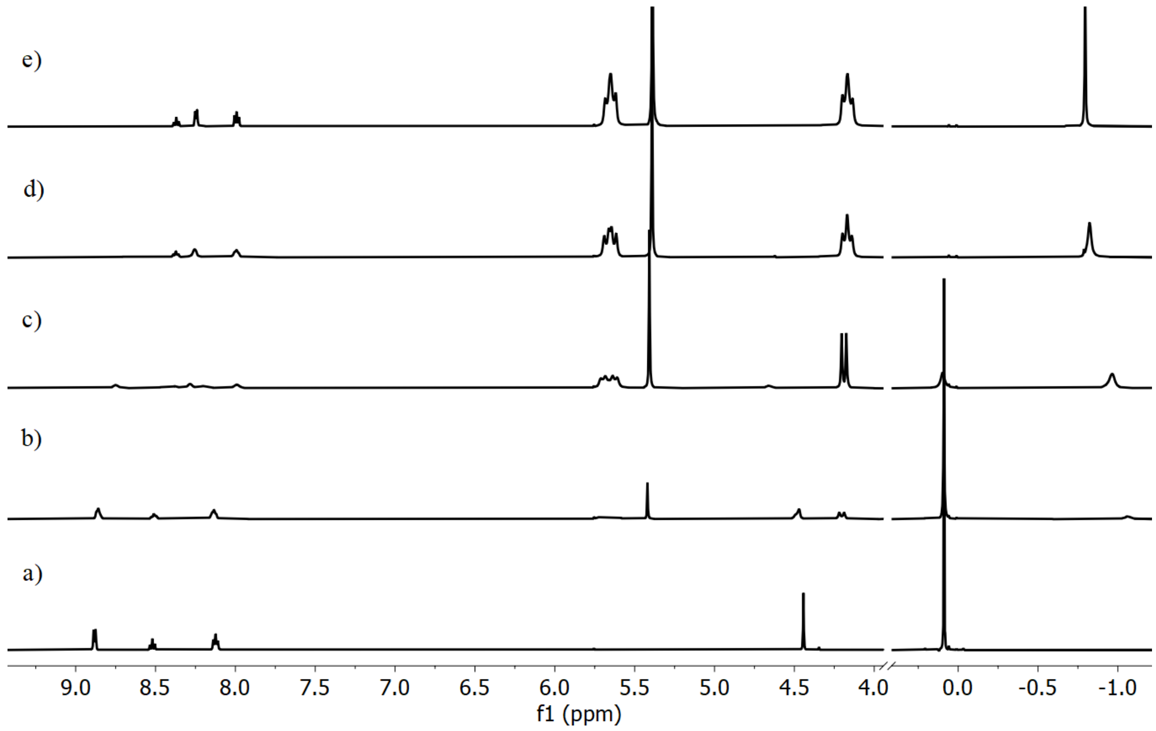

Figure S38. Aromatic and aliphatic regions of ^{1}H NMR spectra of: a) neat guest **4** (c = 1 mmol L^{-1}); and after addition of b) 0.5; c) 1.0; d) 1.5 and e) 2.0 equivalent of **CB[7]** in DMSO-d_6.

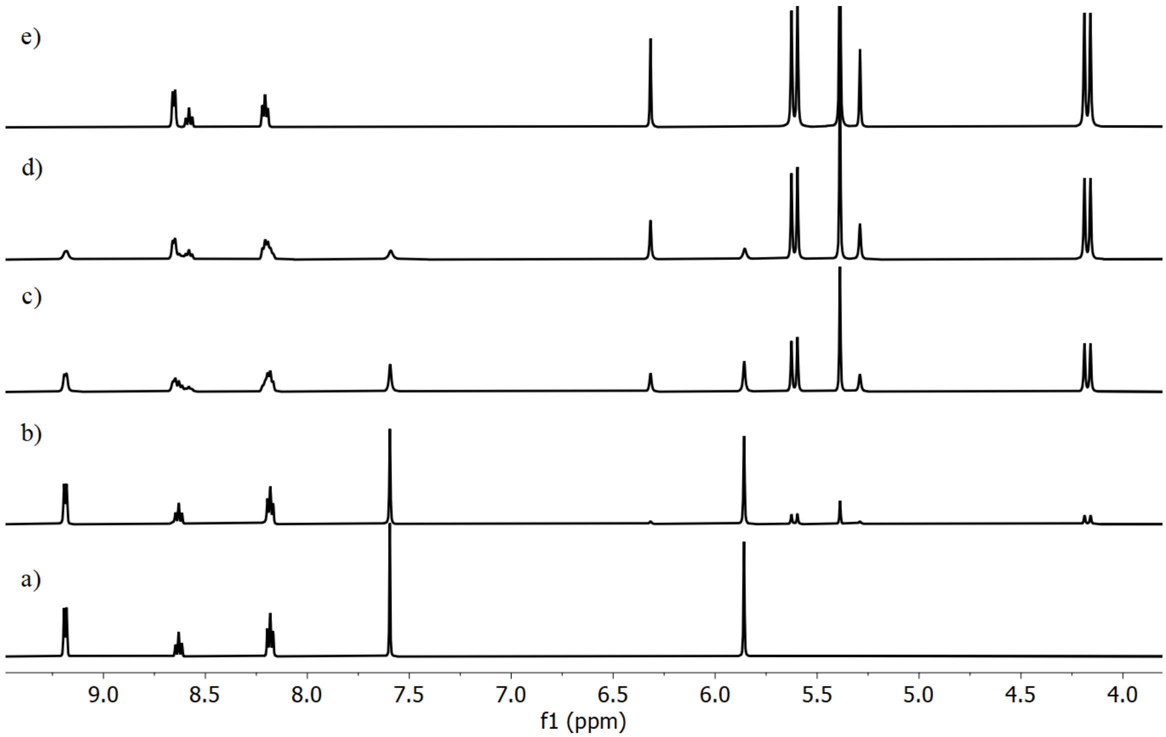

Figure S39. Aromatic and aliphatic regions of ^{1}H NMR spectra of: a) neat guest **5** (c = 1 mmol L^{-1}), and after addition of b) 0.25; c) 0.5; d) 0.75 and e) 2.0 equivalent of **CB[7]** in DMSO-d_6.

4.2. Titrations of guest CB[7]·1 with alkali and earth-alkali metal salts

^{1}H NMR titrations of assembly **CB[7]·1** ith alkali and alkali-earth salts were performed by stepwise addition of up to two equivalents of salt solution (concentrations ranging from 10-50 mM) to solutions of assembly **CB[7]·1** (0.70 mM, 500 µL) in NMR tubes using either micropipettes or Hamilton syringes. The experiments were repeated at least in duplicate for every salt, using newly prepared **CB[7]·1** solutions in each experiment. All of the solutions of complex **CB[7]·1**, as well as salt solutions, were prepared by weighing out at least 6 mg of sample and dissolving it in the appropriate amount of distilled solvent. All the experiments were carried out in DMSO-d_6 at 25 °C.

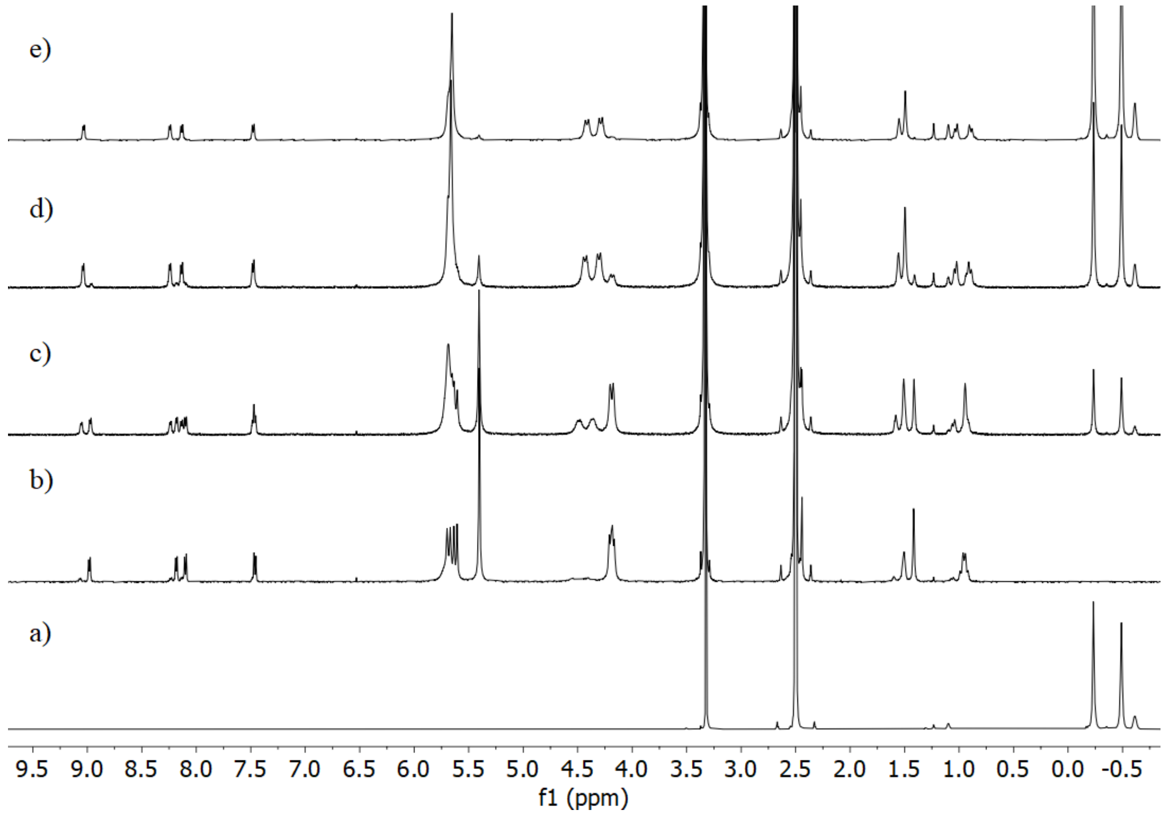

Figure S40. ^{1}H NMR spectra of: a) neat **$NaHCB_{11}Me_{11}$**; b) neat **CB[7]·1** before and c) after addition of 0.2; d) 0.7 and e) 1.0 equivalents of **$NaHCB_{11}Me_{11}$**.

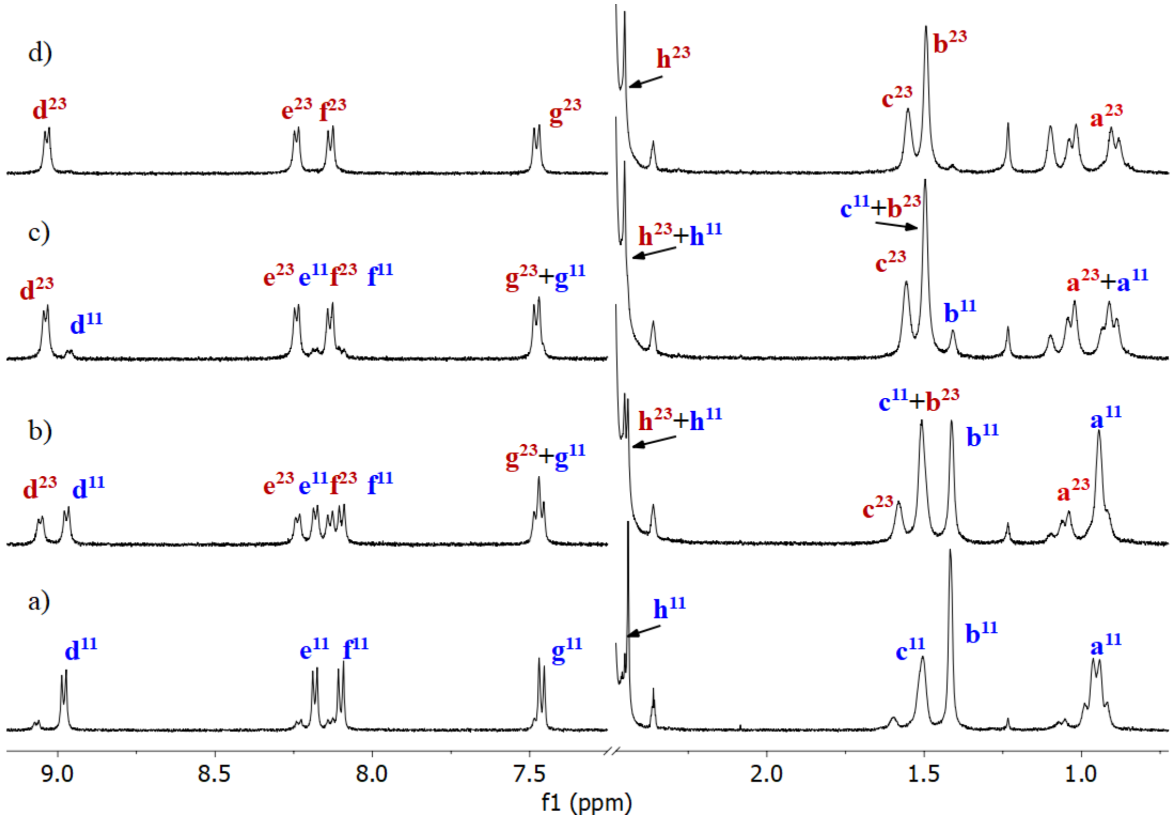

Figure S41. Aromatic and aliphatic regions of ^{1}H NMR spectra of: a) neat **CB[7]·1** before and b) after addition of 0.2, c) 0.7 and d) 1.0 equivalents of **NaHCB$_{11}$Me$_{11}$**.

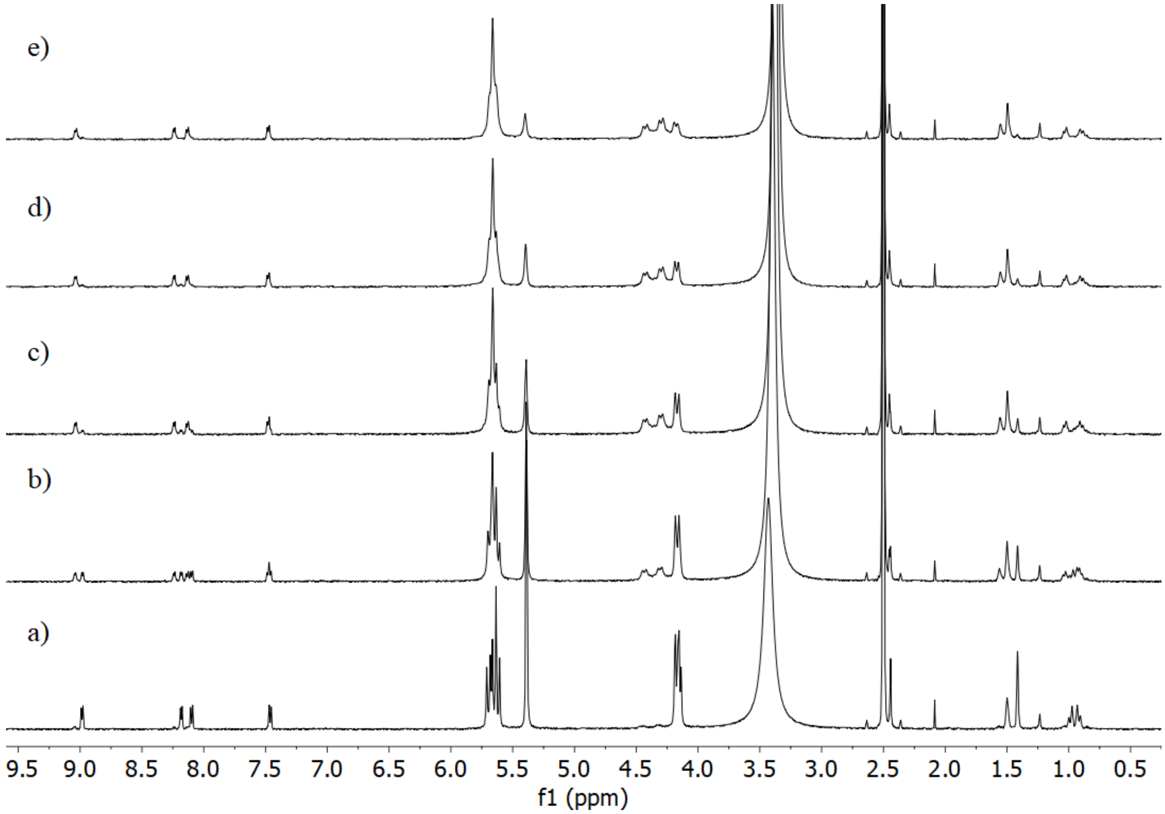

Figure S42. ^{1}H NMR spectra of: a) neat **CB[7]·1** before and b) after addition of 0.3, c) 0.8, d)1.3 and e) 2.0 equivalents of **NaCl**.

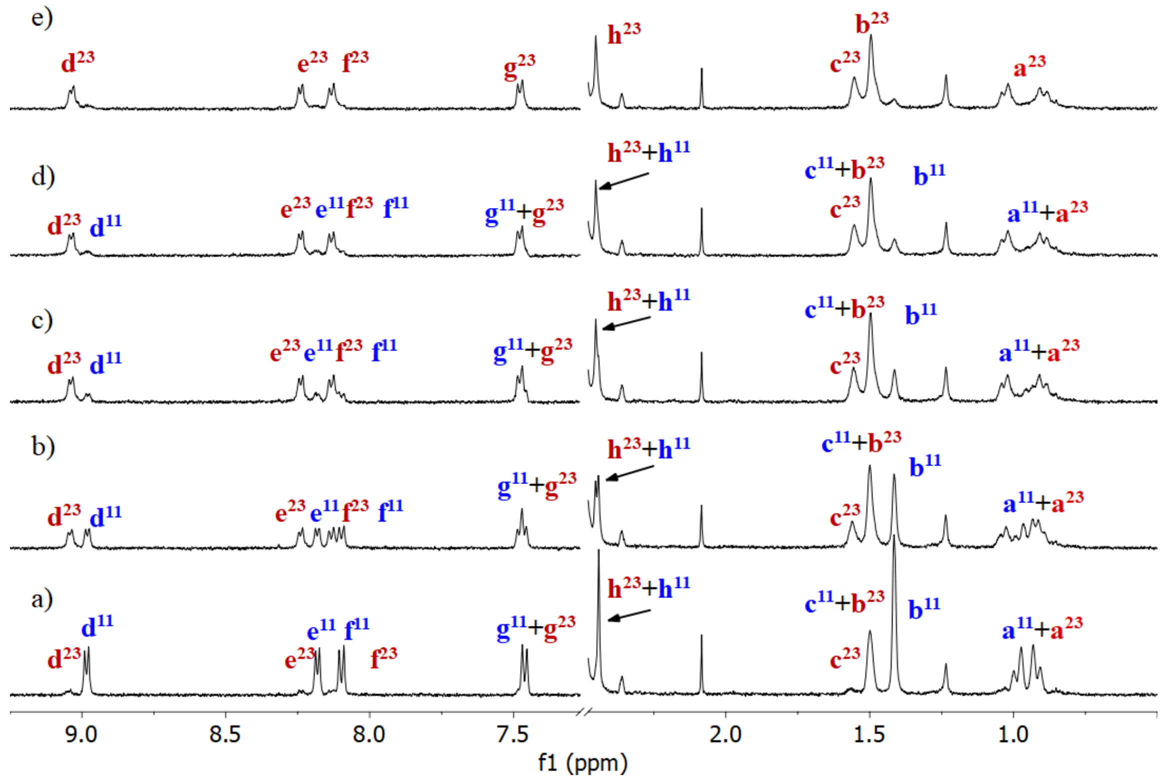

Figure S43. Aromatic and aliphatic regions of ^{1}H NMR spectra of: a) neat **CB[7]·1** before and b) after addition of 0.3, c) 0.8; d)1.3 and e) 2.0 equivalents of **NaCl**.

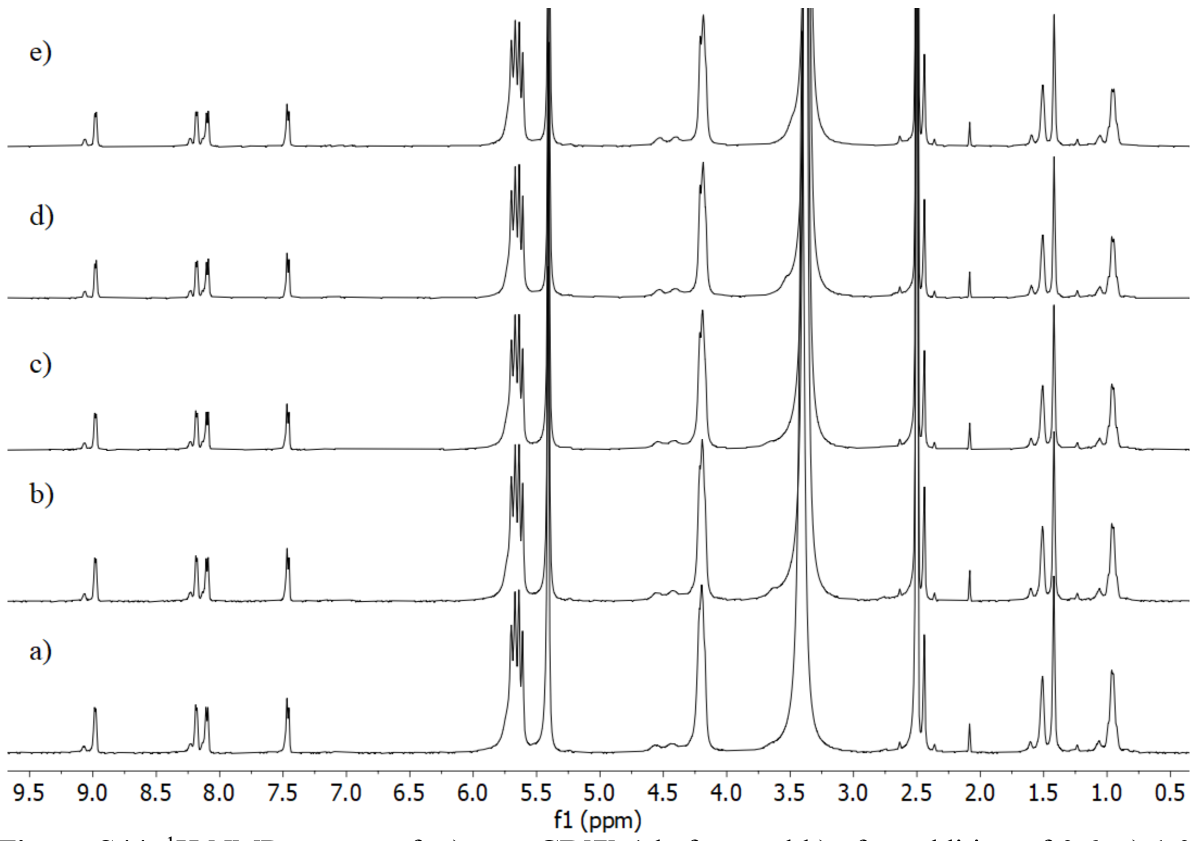

Figure S44. ^{1}H NMR spectra of: a) neat **CB[7]·1** before and b) after addition of 0.6, c) 1.0, d)1.6 and e) 2.0 equivalents of **NaF**.

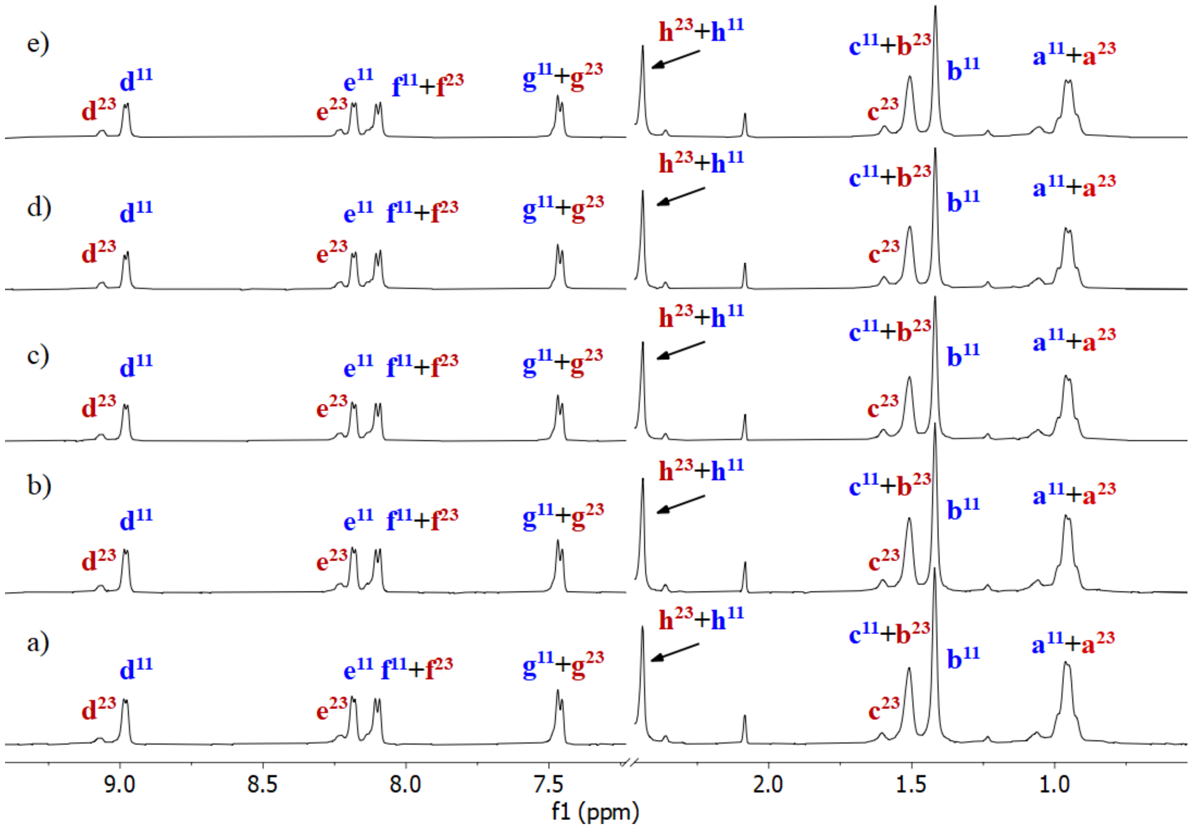

Figure S45. Aromatic and aliphatic regions of ^{1}H NMR spectra of: a) neat **CB[7]·1** before and b) after addition of 0.6, c) 1.0; d)1.6 and e) 2.0 equivalents of **NaF**.

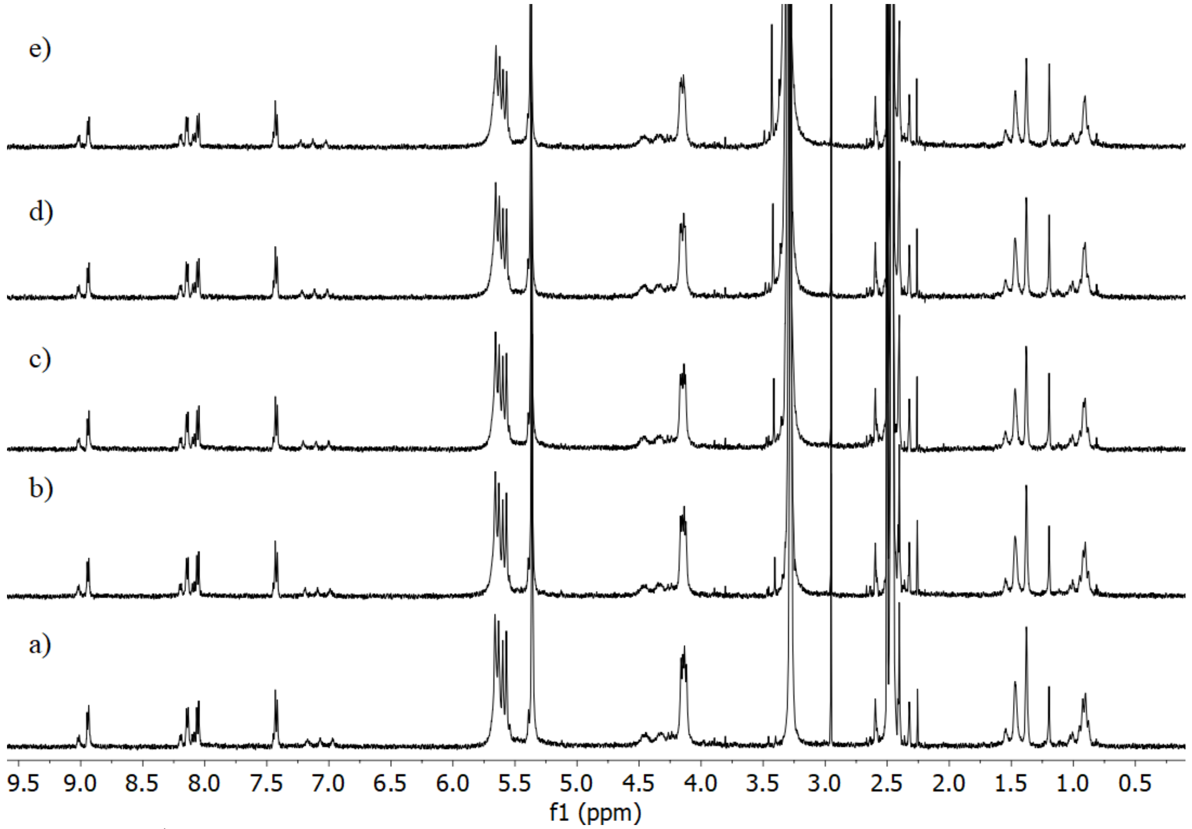

Figure S46. ^{1}H NMR spectra of a) neat **CB[7]·1** before, and b) after addition of 0.5, c) 0.9; d) 1.4 and e) 2.0 equivalents of **LiCl**.

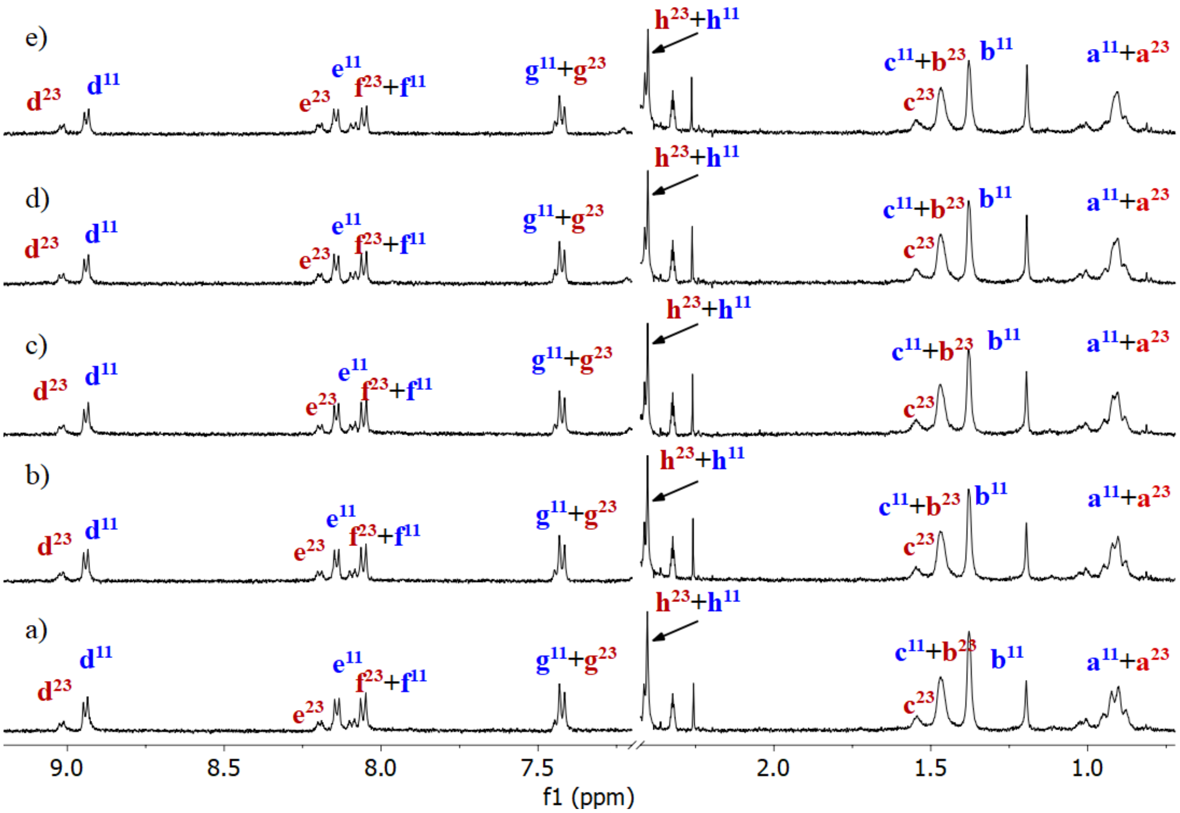

Figure S47. Aromatic and aliphatic regions of ^{1}H NMR spectra of a) neat **CB[7]·1** before, and b) after addition of 0.5, c) 0.9, d) 1.4 and e) 2.0 equivalents of **LiCl**.

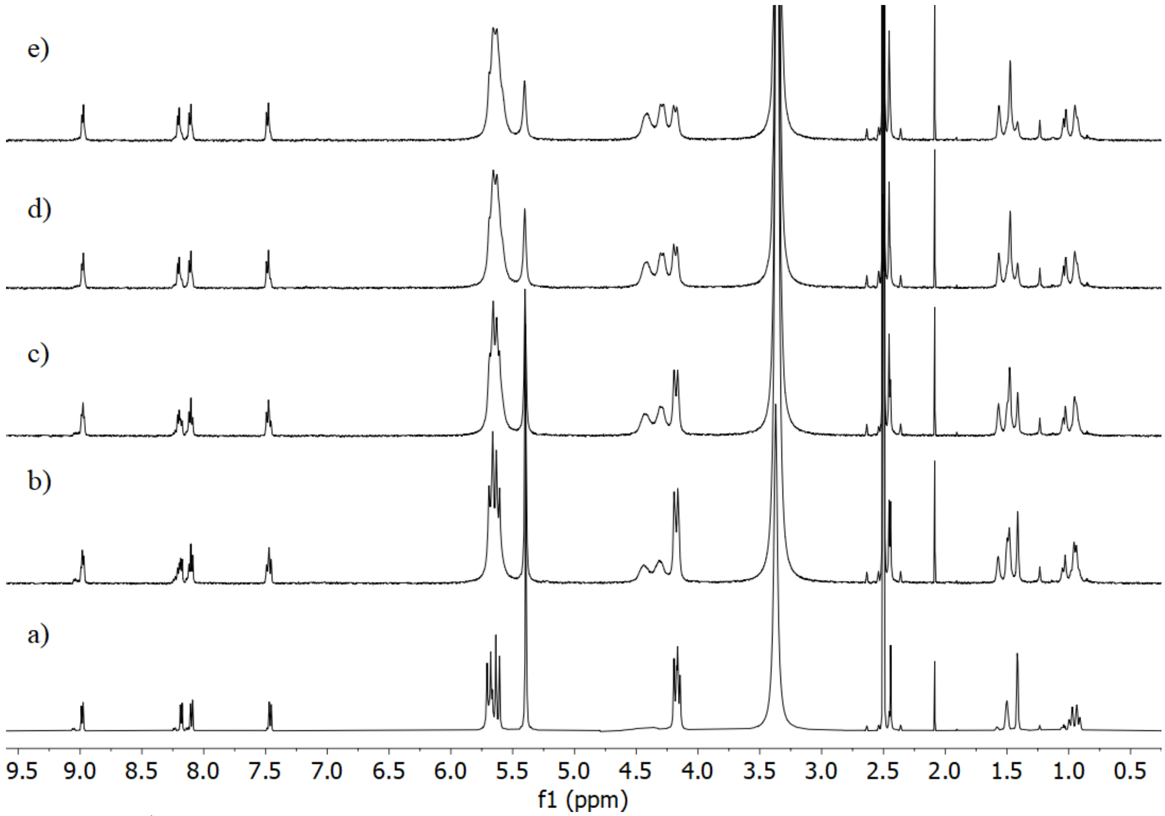

Figure S48. ^{1}H NMR spectra of: a) neat **CB[7]·1** before, and b) after addition of 0.6, c) 1.0, d) 1.6 and e) 2.0 equivalents of **KCl**.

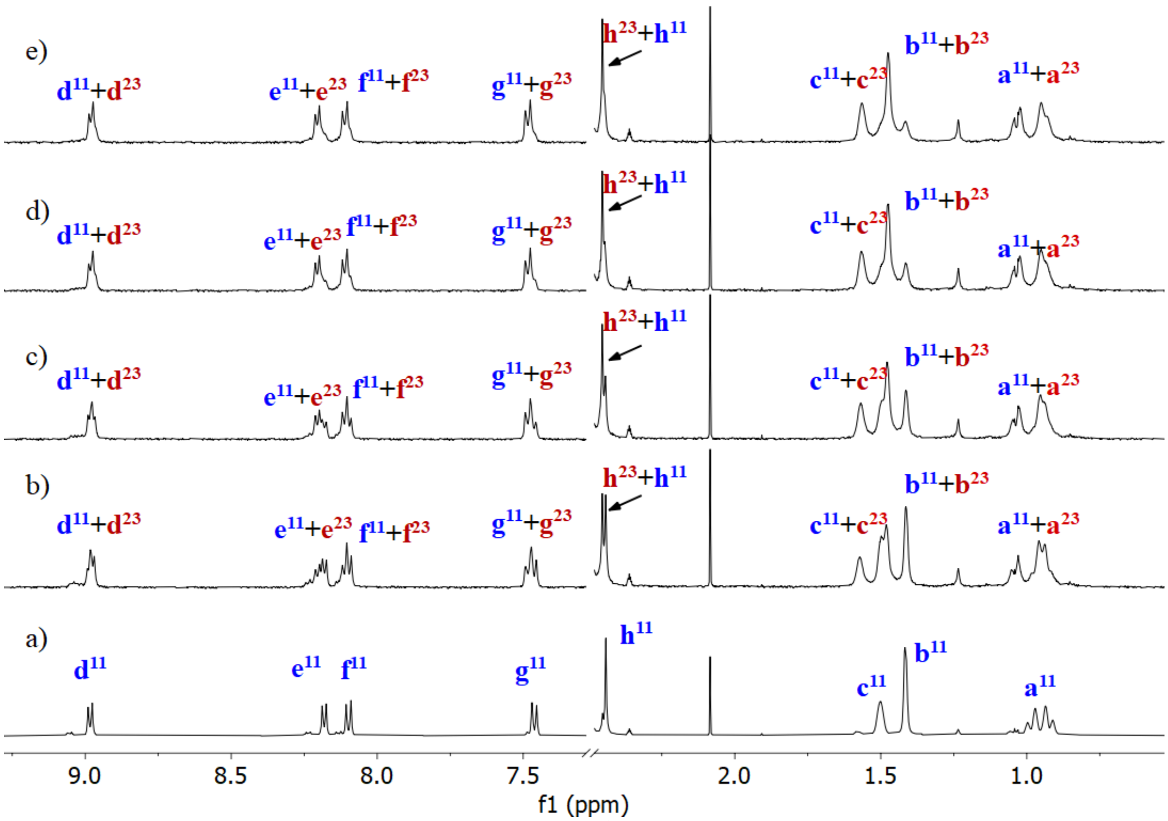

Figure S49. Aromatic and aliphatic regions of ^{1}H NMR spectra of: a) neat **CB[7]·1** before; and b) after addition of 0.6, c) 1.0, d) 1.6 and e) 2.0 equivalents of **KCl**.

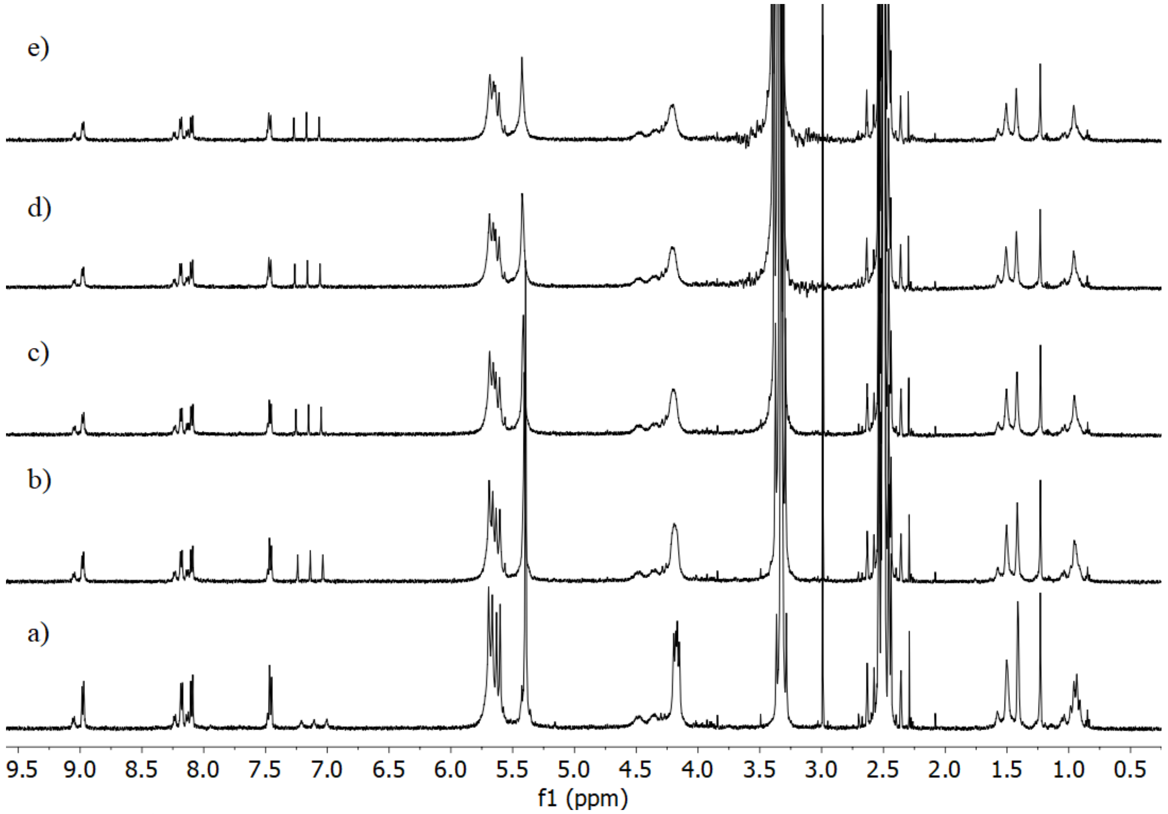

Figure S50. ^{1}H NMR spectra of a) neat **CB[7]·1** before, and b) after addition of 0.4, c) 1.0, d) 1.4 and e) 2.0 equivalents of **RbCl**.

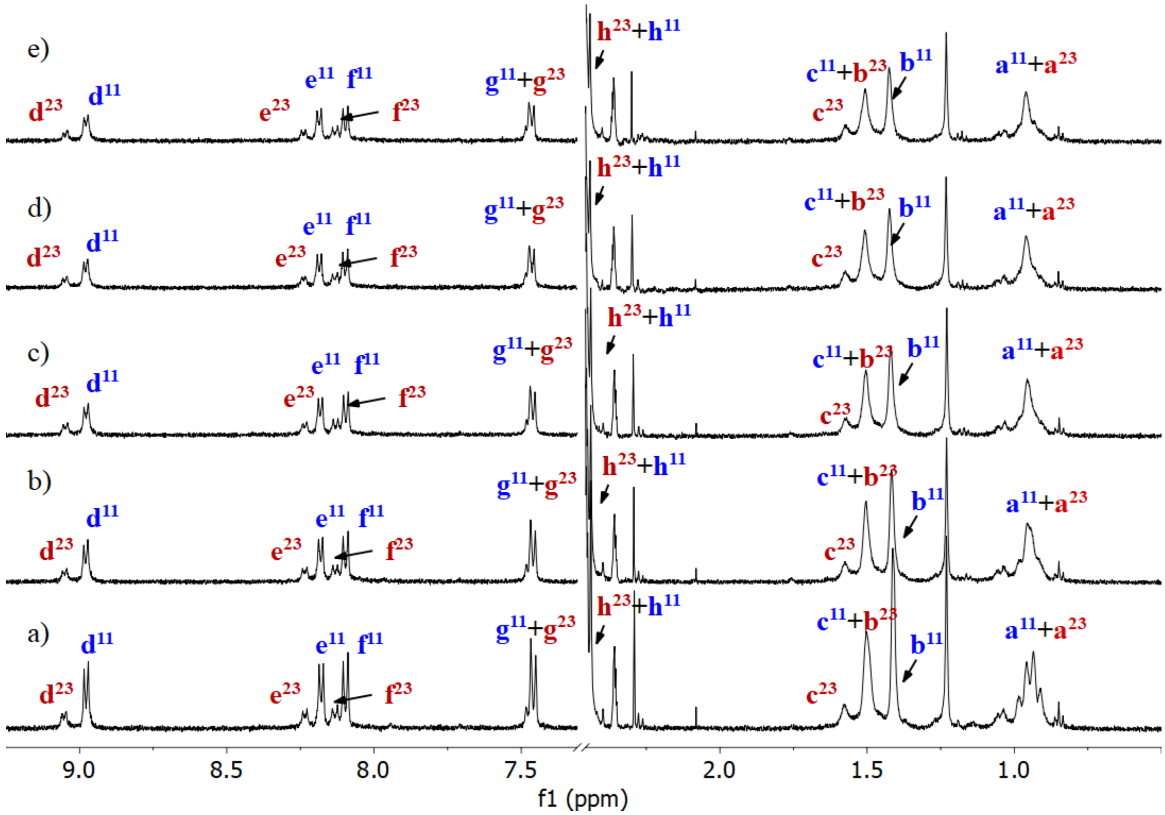

Figure S51. Aromatic and aliphatic regions of ^{1}H NMR spectra of a) neat **CB[7]·1** before, and b) after addition of 0.4, c) 1.0, d) 1.4 and e) 2.0 equivalents of **RbCl**.

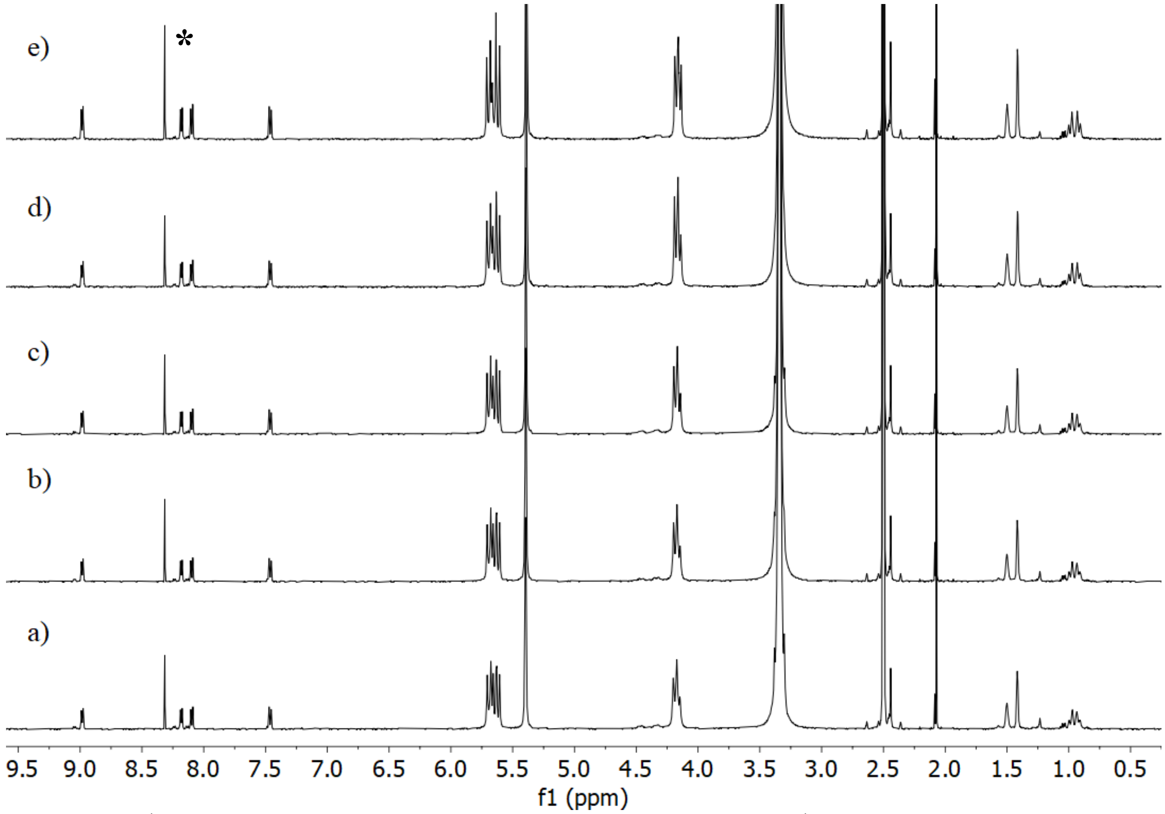

Figure S52. ^{1}H NMR spectra of a) neat **CB[7]·1** (c = 1 mmol L^{-1}) before; and b) after addition of 0.5, c) 1.0, d) 1.5 and e) 2.0 equivalents of **CsCl**. * Signal belongs to chloroform.

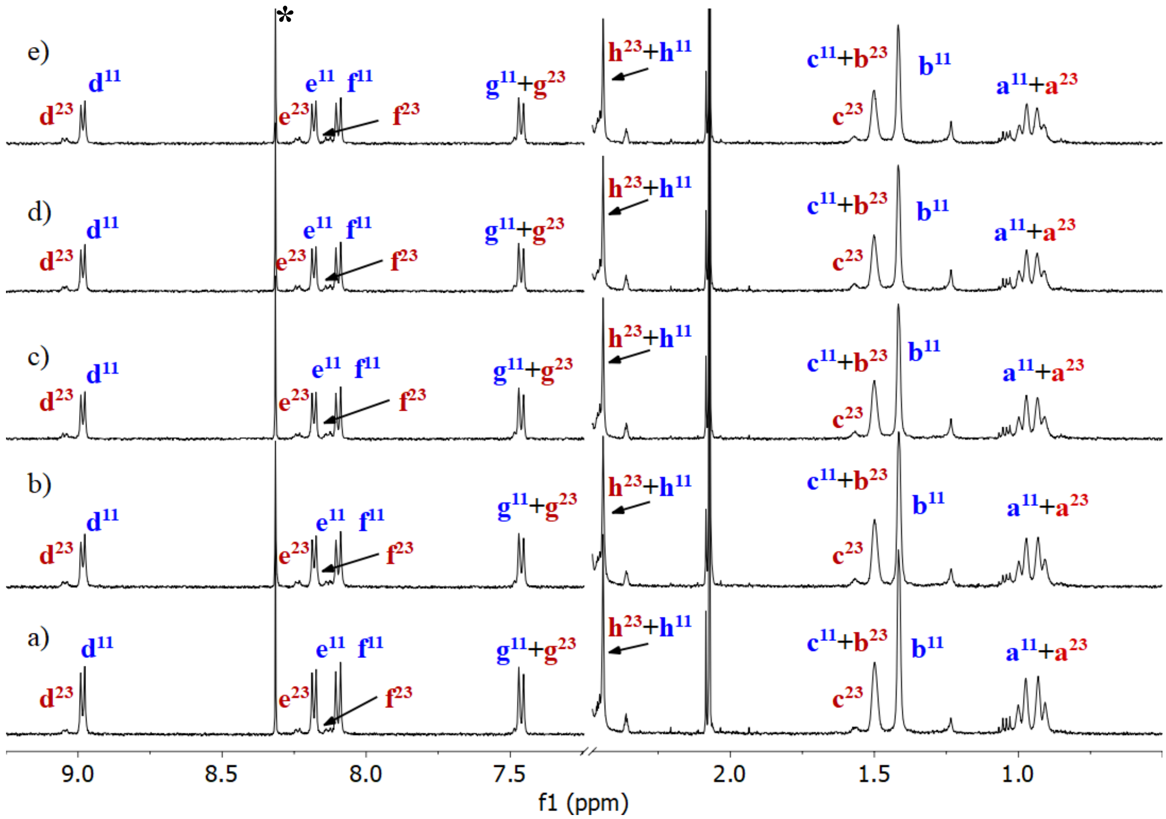

Figure S53. Aromatic and aliphatic regions of ^1H NMR spectra of a) neat **CB[7]·1** (c = 1 mmol L^{-1}) before, and b) after addition of 0.5, c) 1.0, d) 1.5 and e) 2.0 equivalents of **CsCl**. * Signal belongs to chloroform.

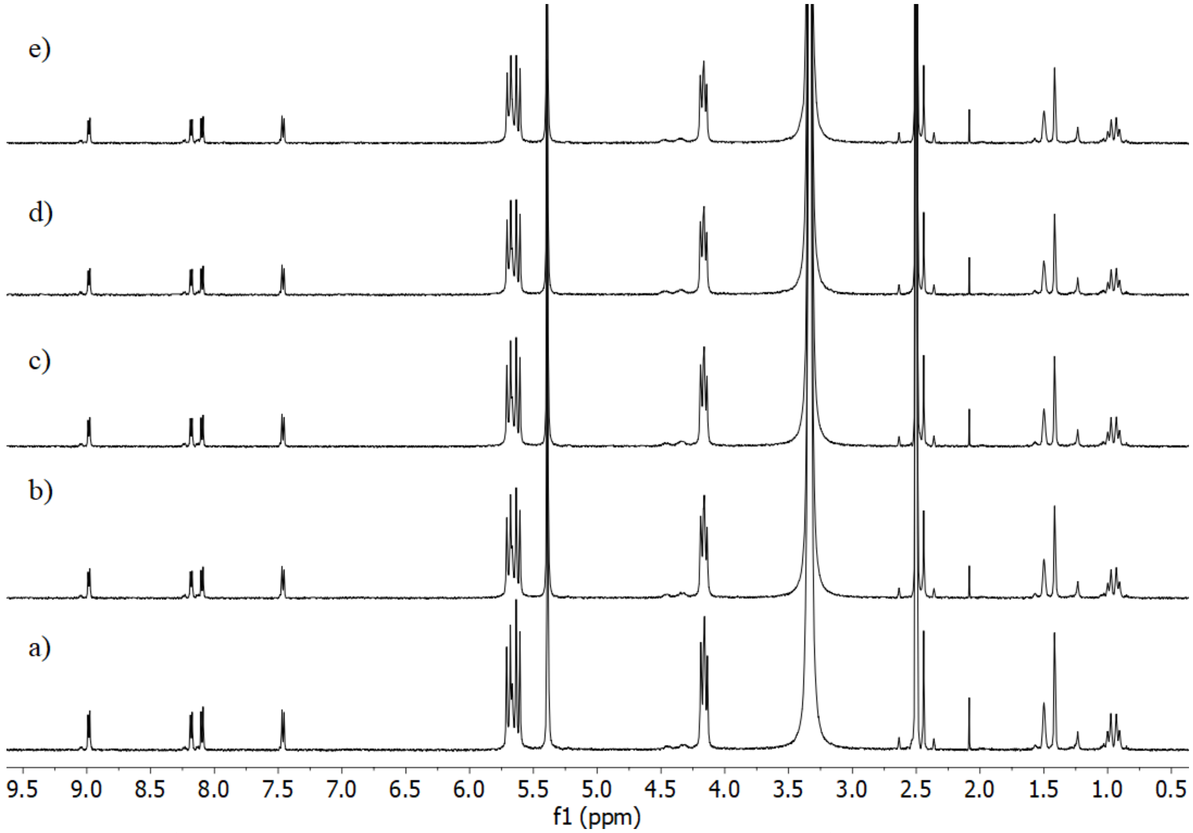

Figure S54. ^1H NMR spectra of a) neat **CB[7]·1** (c = 1 mmol L^{-1}) before; and b) after addition of 0.6, c) 1.0, d) 2.0 and e) 2.8 equivalents of **$MgCl_2$**.

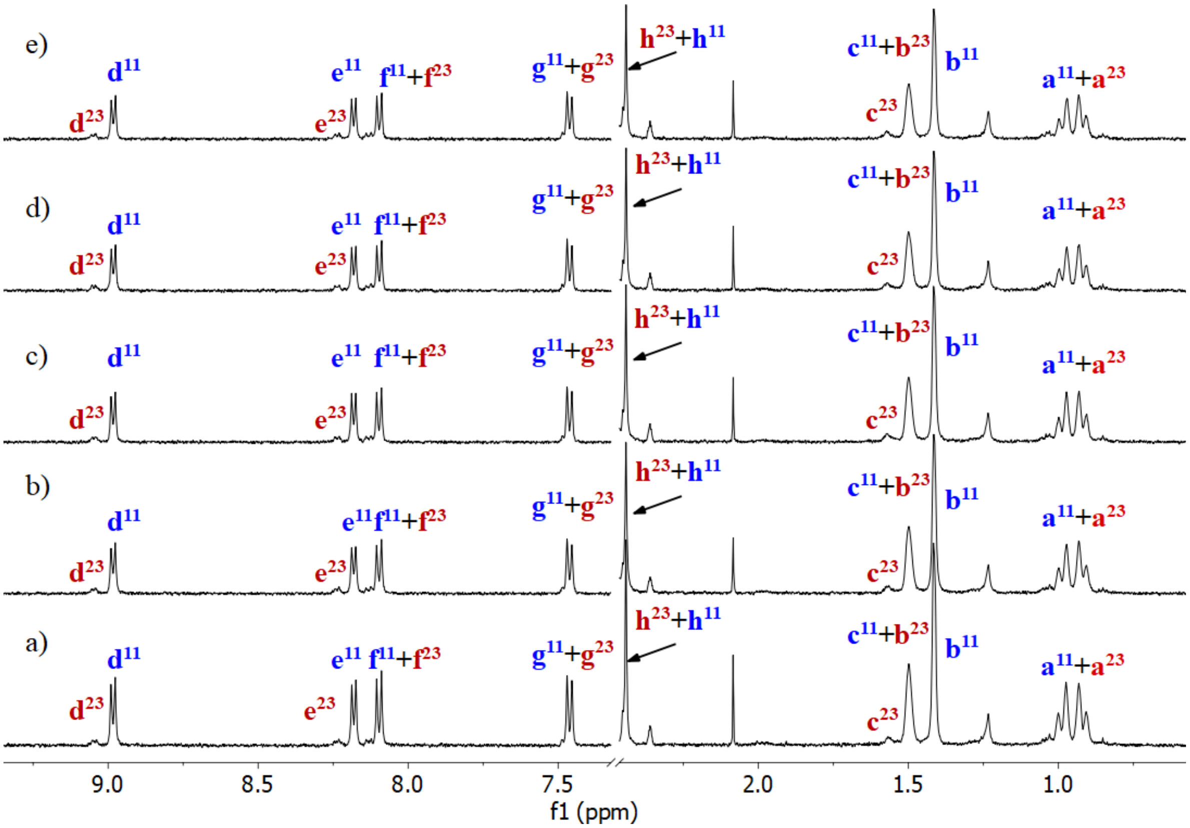

Figure S55. Aromatic and aliphatic regions of ^{1}H NMR spectra of a) neat **CB[7]·1** (c = 1 mmol L^{-1}) before; and b) after addition of 0.6, c) 1.0, d) 2.0 and e) 2.8 equivalents of **$MgCl_2$**.

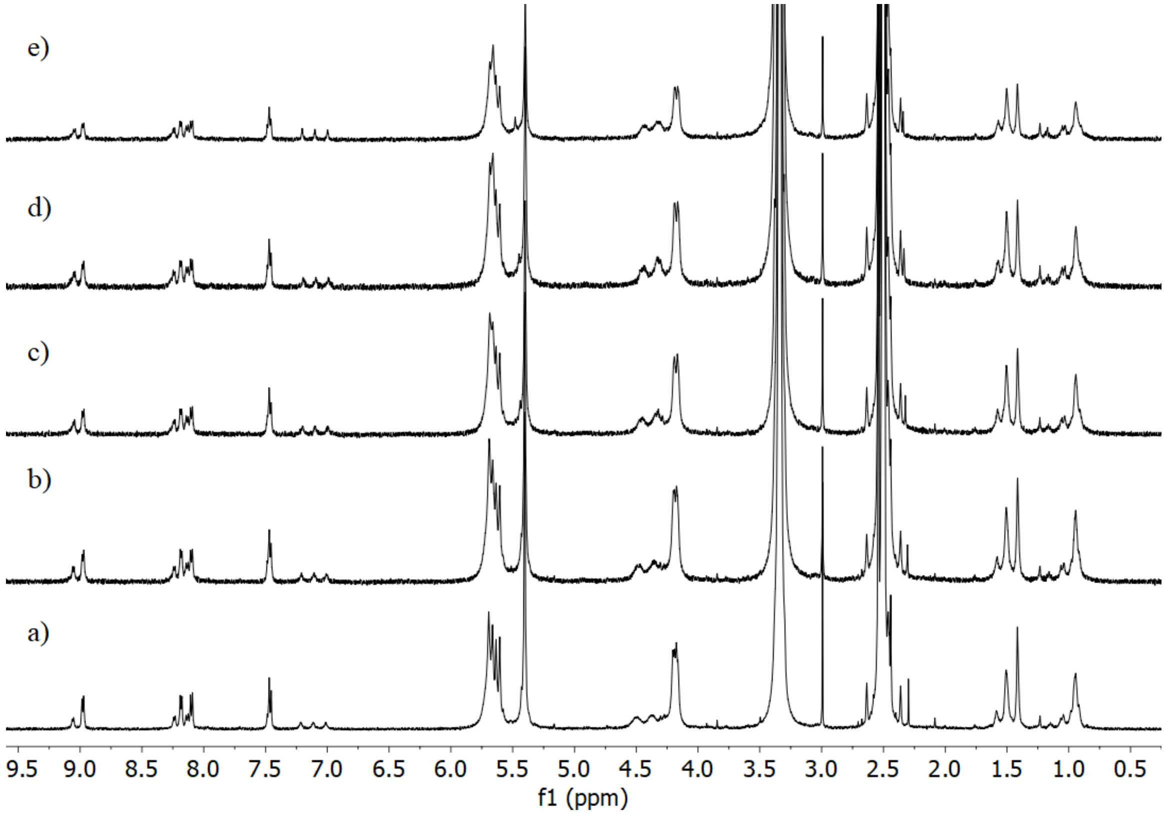

Figure S56. ^{1}H NMR spectra of a) neat **CB[7]·1** before, and b) after addition of 0.2, c) 0.8, d) 1.4 and e) 2.0 equivalents of **$Ca(OTf)_2$**.

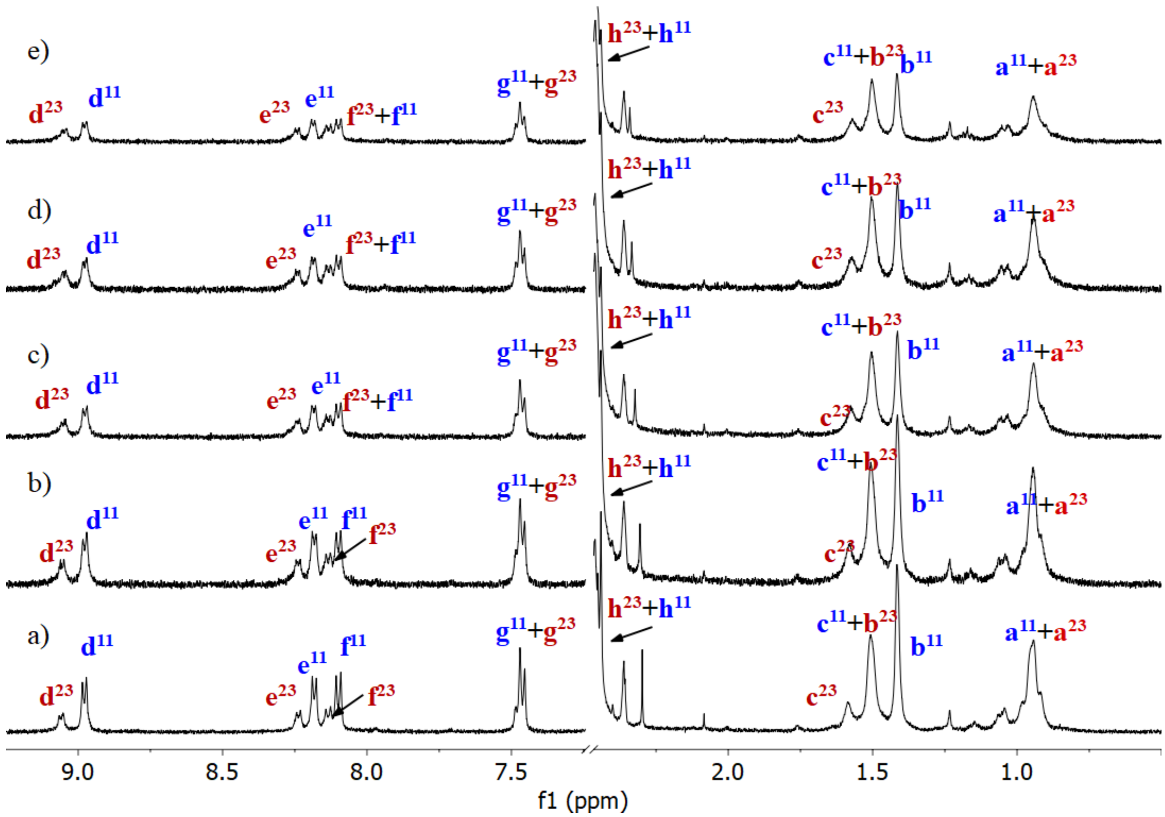

Figure S57. Aromatic and aliphatic regions of ^{1}H NMR spectra of a) neat **CB[7]·1** before, and b) after addition of 0.2, c) 0.8, d) 1.4 and e) 2.0 equivalents of **Ca(OTf)$_2$**.

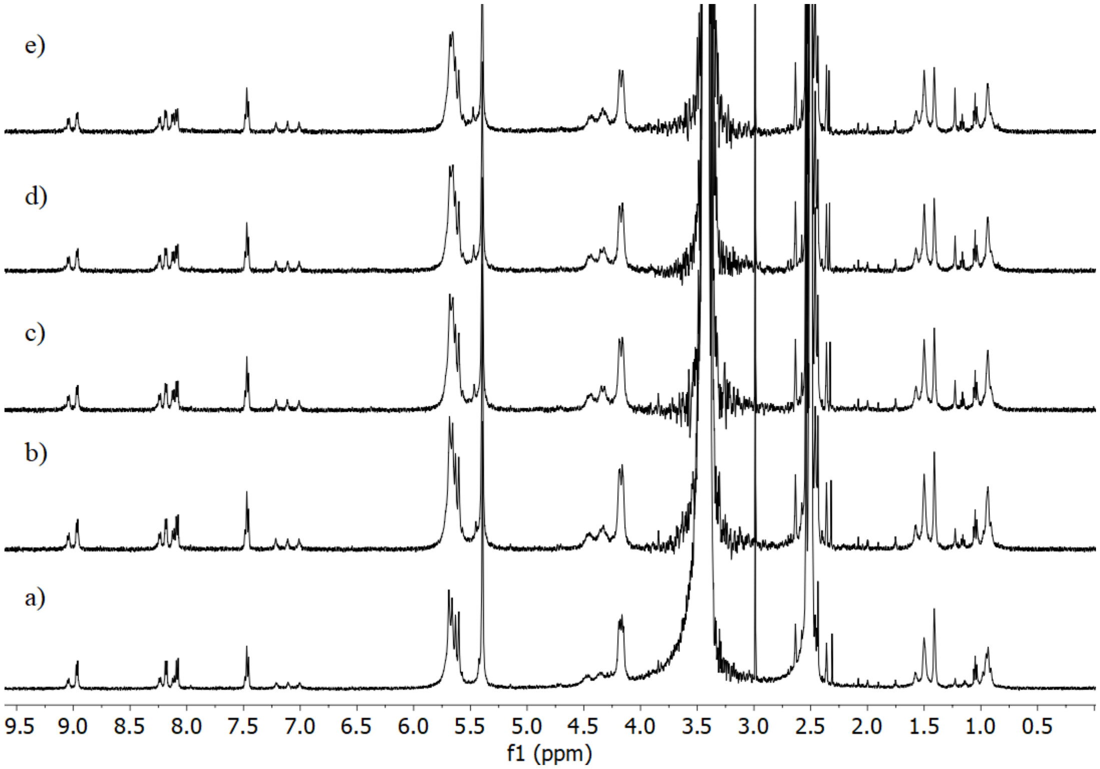

Figure S58. ^{1}H NMR spectra of a) neat **CB[7]·1** before, and b) after addition of 0.4, c) 1.0, d) 1.4 and e) 2.0 equivalents of **Ca(NO$_3$)$_2$**.

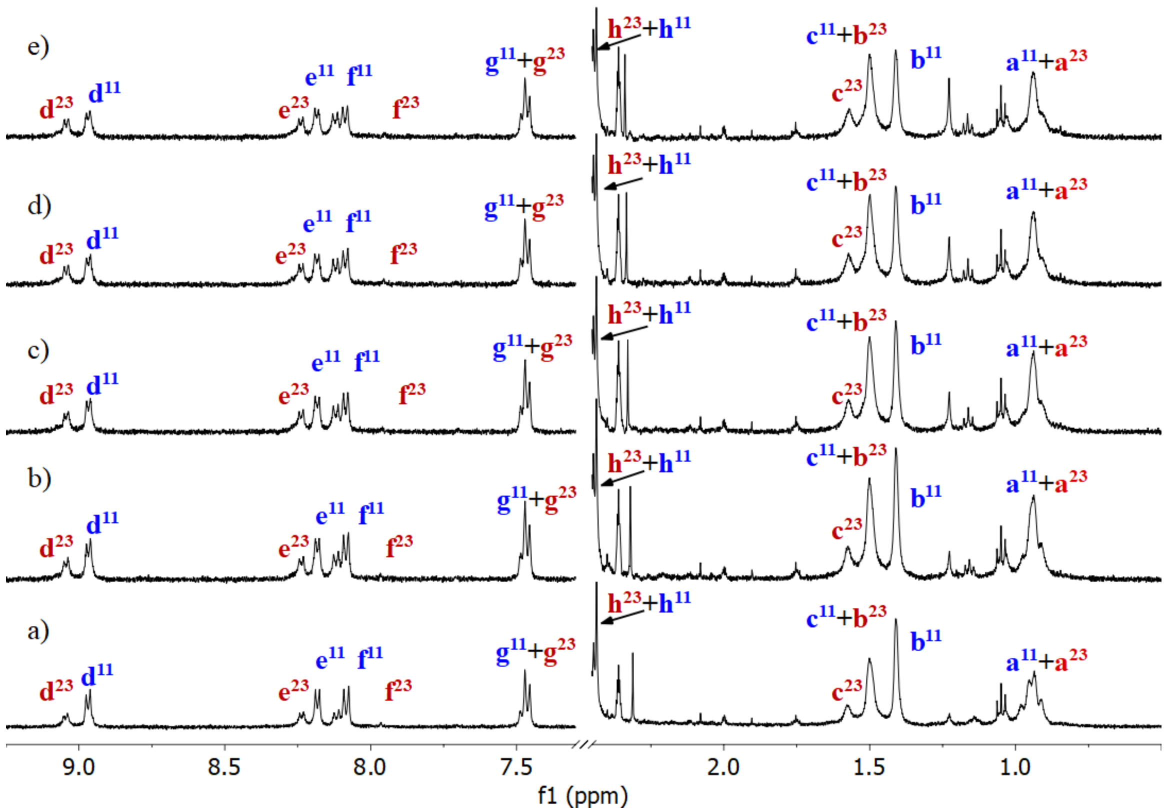

Figure S59. Aromatic and aliphatic regions of ^{1}H NMR spectra of a) neat **CB[7]·1** before, and b) after addition of 0.4, c) 1.0, d) 1.4 and e) 2.0 equivalents of **$Ca(NO_3)_2$**.

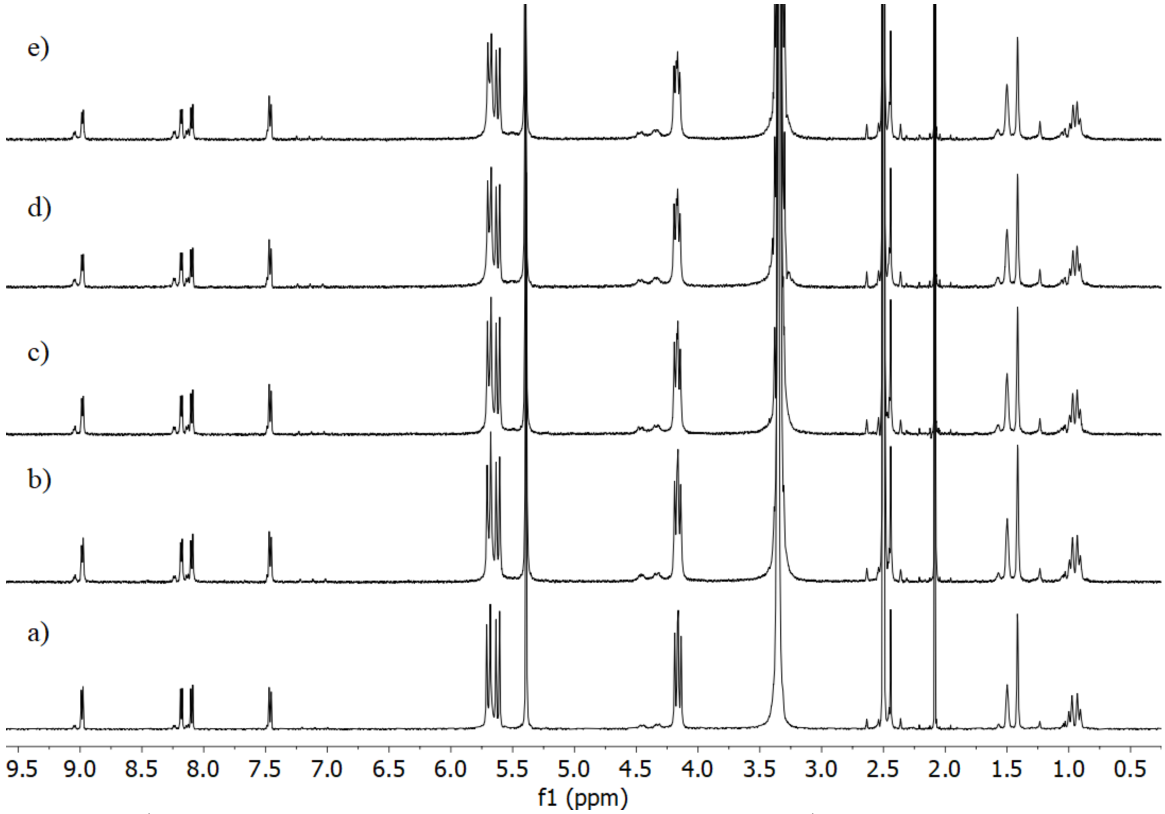

Figure S60. ^{1}H NMR spectra of a) neat **CB[7]·1** (c = 1.1 mmol L^{-1}) before, and b) after addition of 0.5, c) 1.0, d) 1.5 and e) 2.0 equivalents of **$SrCl_2$**.

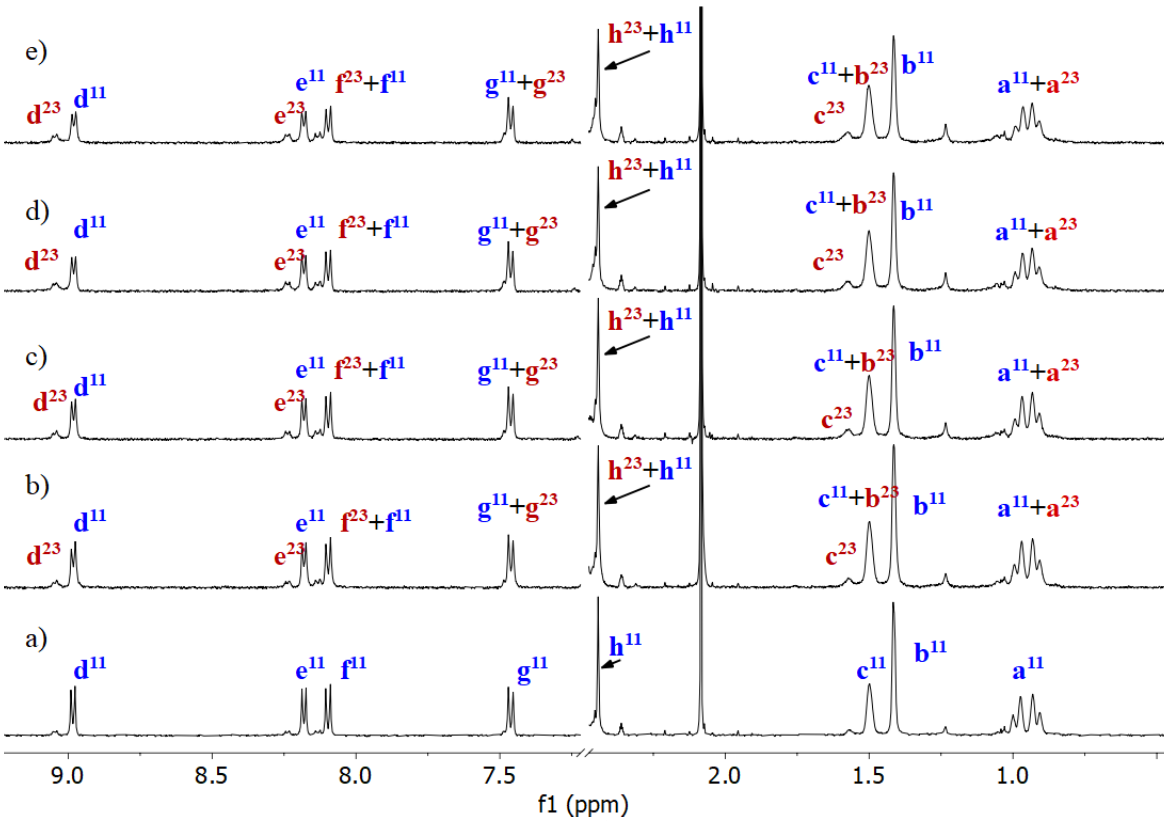

Figure S61. Aromatic and aliphatic regions of ^{1}H NMR spectra of a) neat **CB[7]·1** (c = 1.1 mmol L^{-1}) before, and b) after addition of 0.5, c) 1.0, d) 1.5 and e) 2.0 equivalents of **$SrCl_2$**.

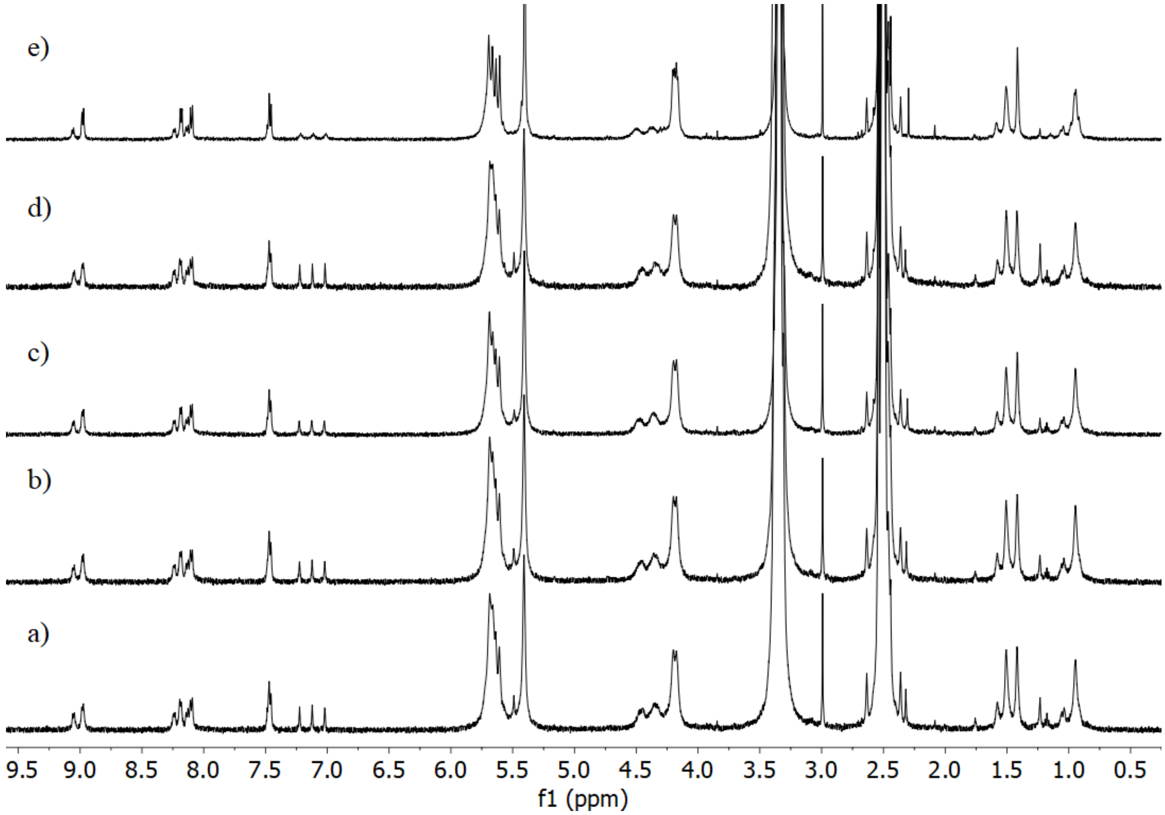

Figure S62. ^{1}H NMR spectra of a) neat **CB[7]·1** before, and b) after addition of 0.5, c) 1.0, d) 1.5 and e) 2.0 equivalents of **$Ba(NO_3)_2$**.

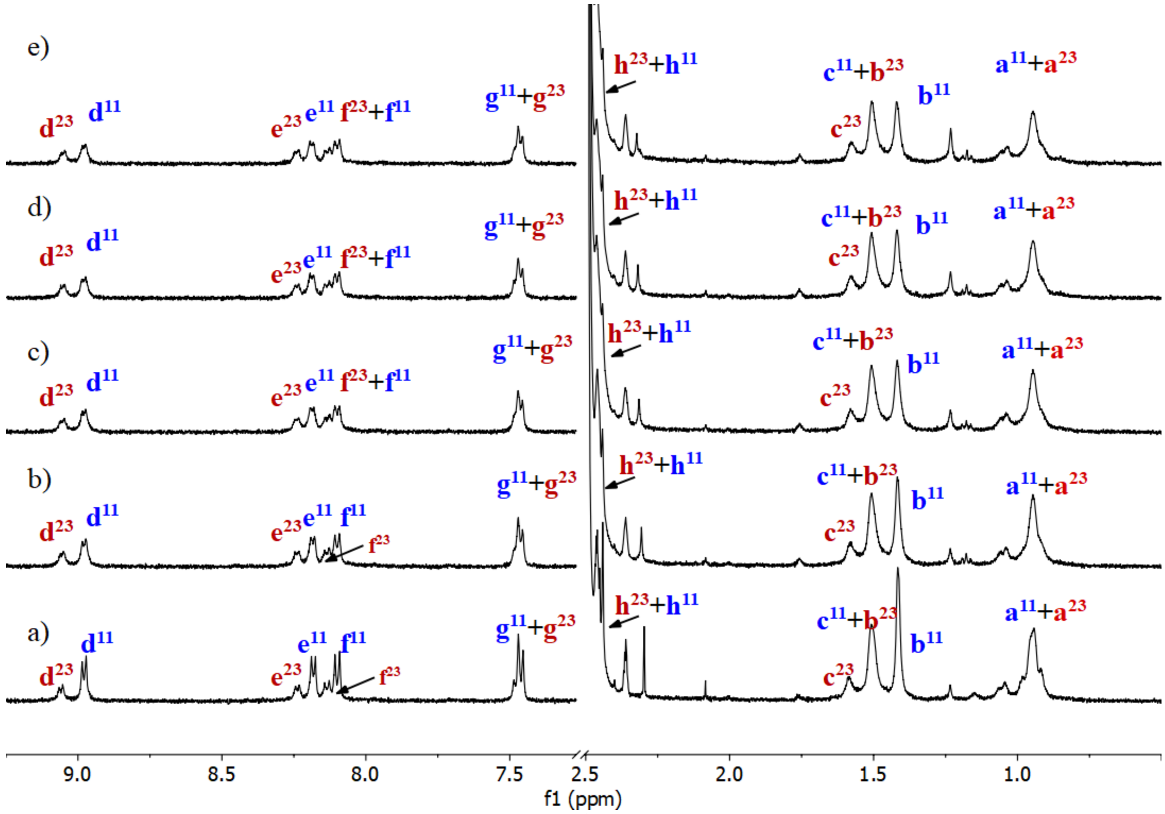

Figure S63. Aromatic and aliphatic regions of ^1H NMR spectra of a) neat **CB[7]·1** before, and b) after addition of 0.5, c) 1.0, d) 1.5 and e) 2.0 equivalents of **$Ba(NO_3)$**.

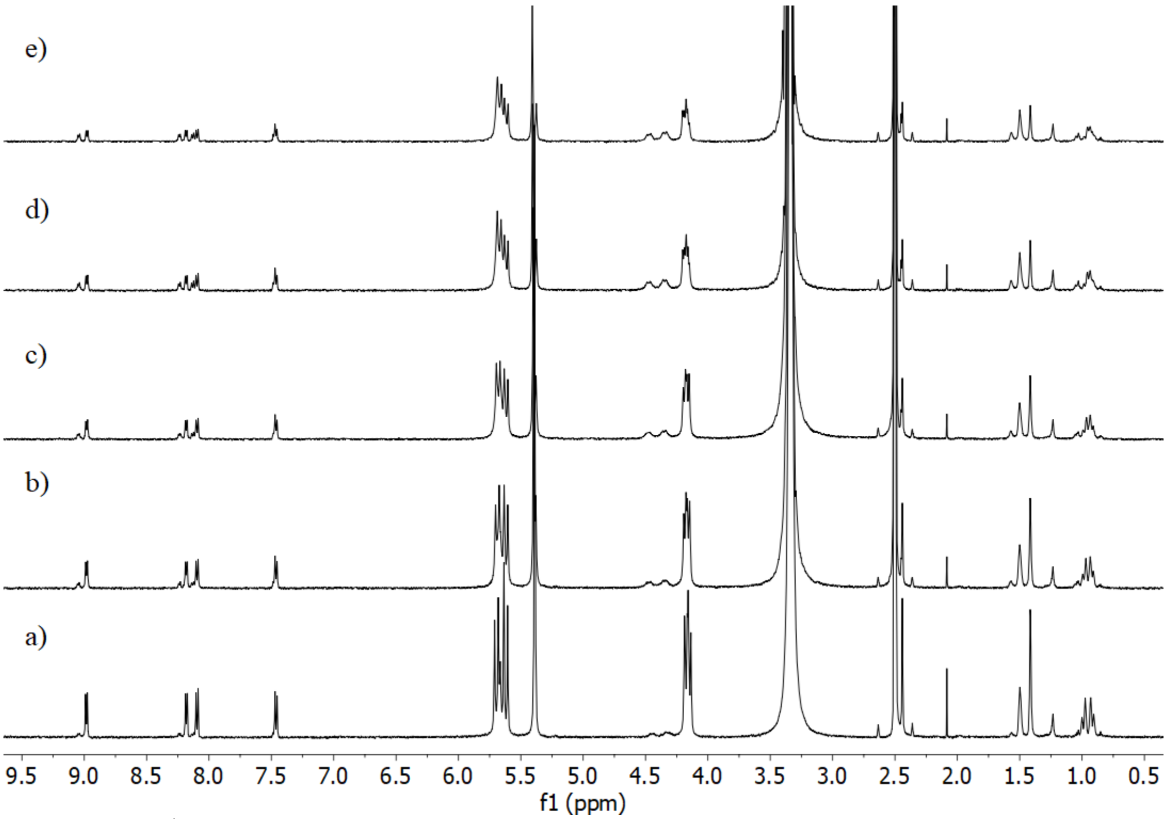

Figure S64. ^1H NMR spectra of a) neat **CB[7]·1** before, and b) after addition of 1.0, c) 2.0, d) 4.0 and e) 4.7 equivalents of **$EuCl_3$**.

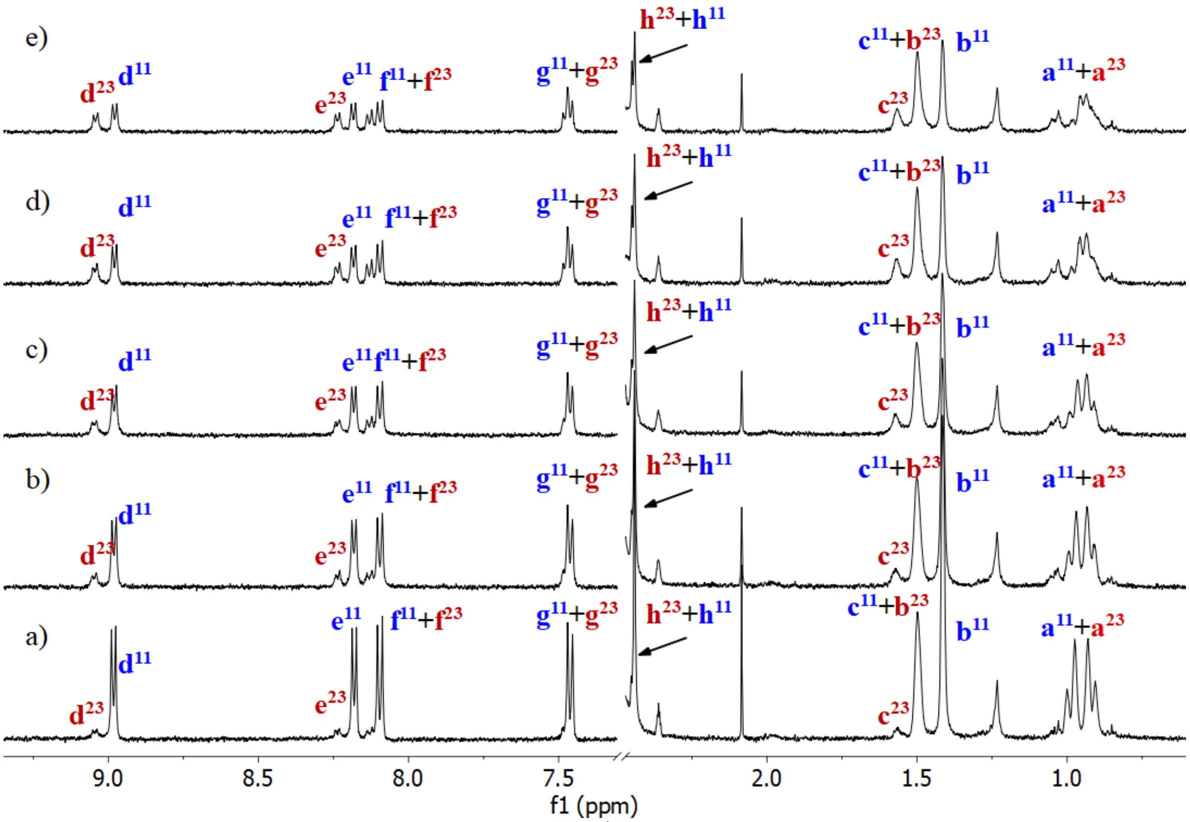

Figure S25. Aromatic and aliphatic regions of ^{1}H NMR spectra of a) neat **CB[7]·1** before, and b) after addition of 1.0, c) 2.0, d) 4.0 and e) 4.7 equivalents of **EuCl₃**.

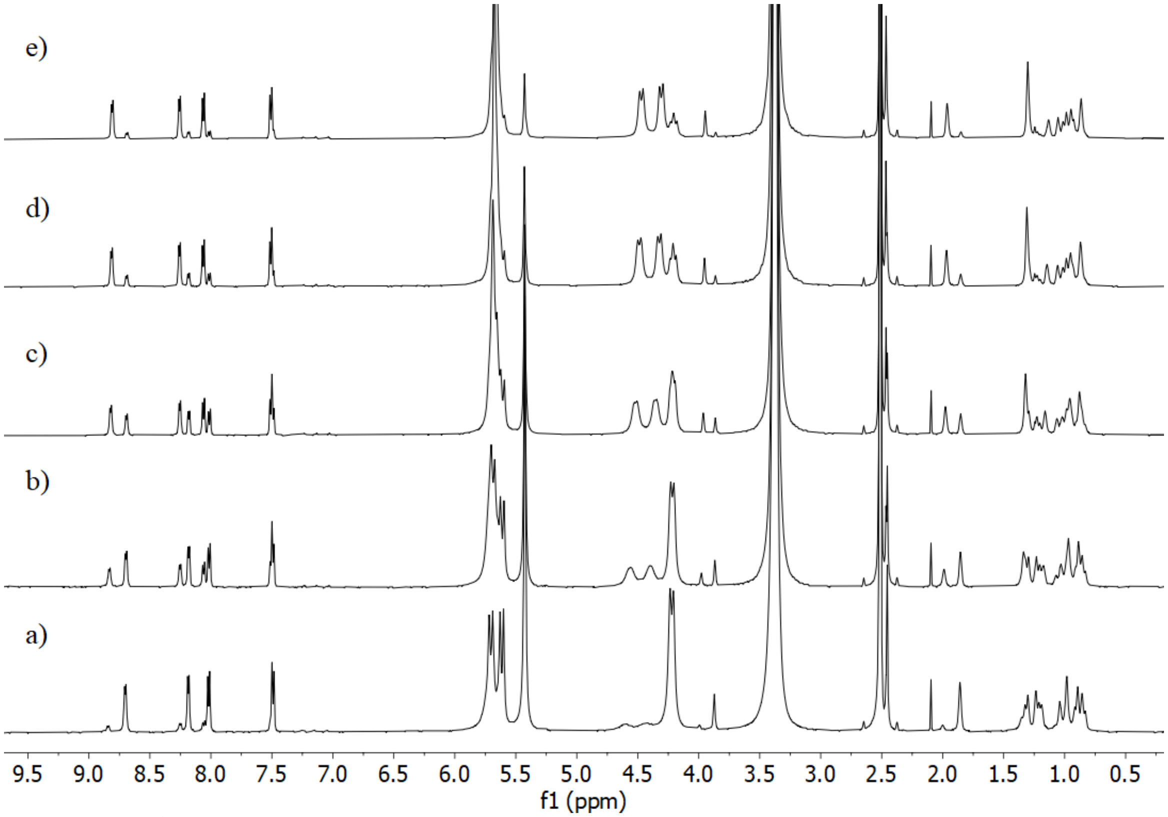

Figure S66. ^{1}H NMR spectra of a) neat **CB[7]·2** before, and b) after addition of 0.5, c) 1.0, d) 1.5 and e) 2.0 equivalents of **NaCl**.

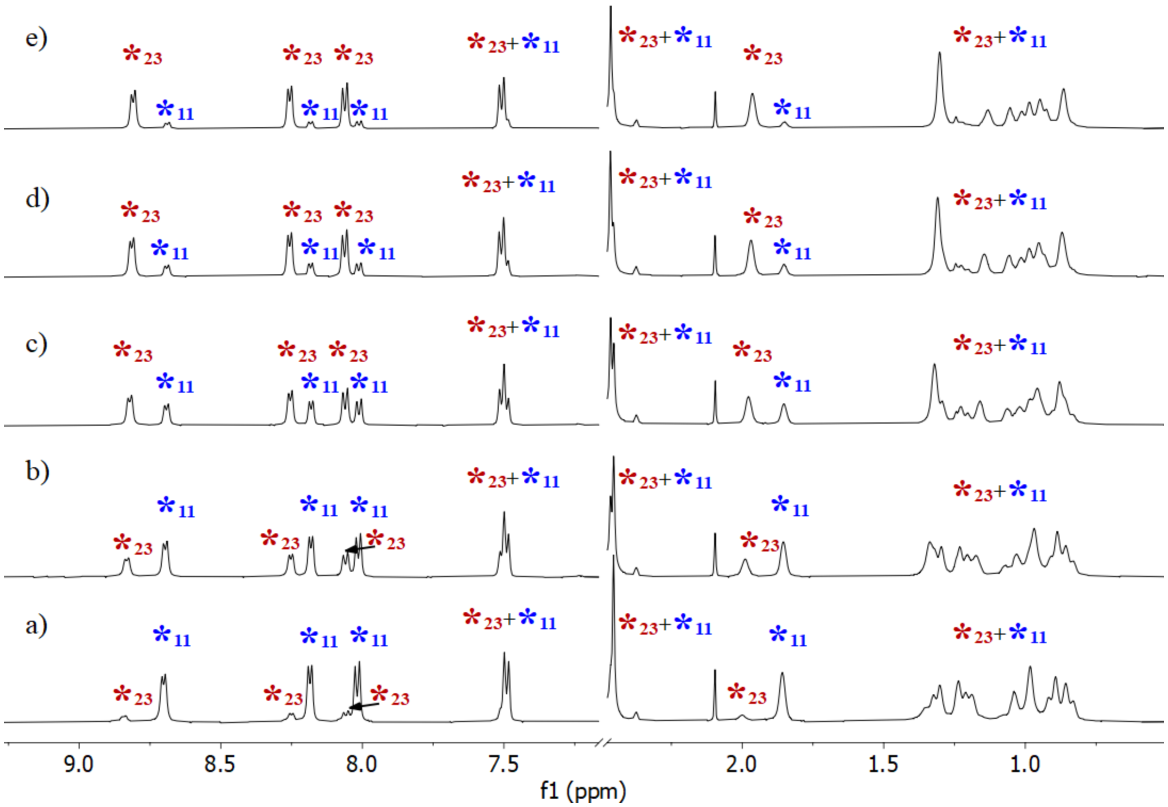

Figure S67. Aromatic and aliphatic regions of ^{1}H NMR spectra of a) neat **CB[7]·2** before, and b) after addition of 0.5, c) 1.0, d) 1.5 and e) 2.0 equivalents of **NaCl**.

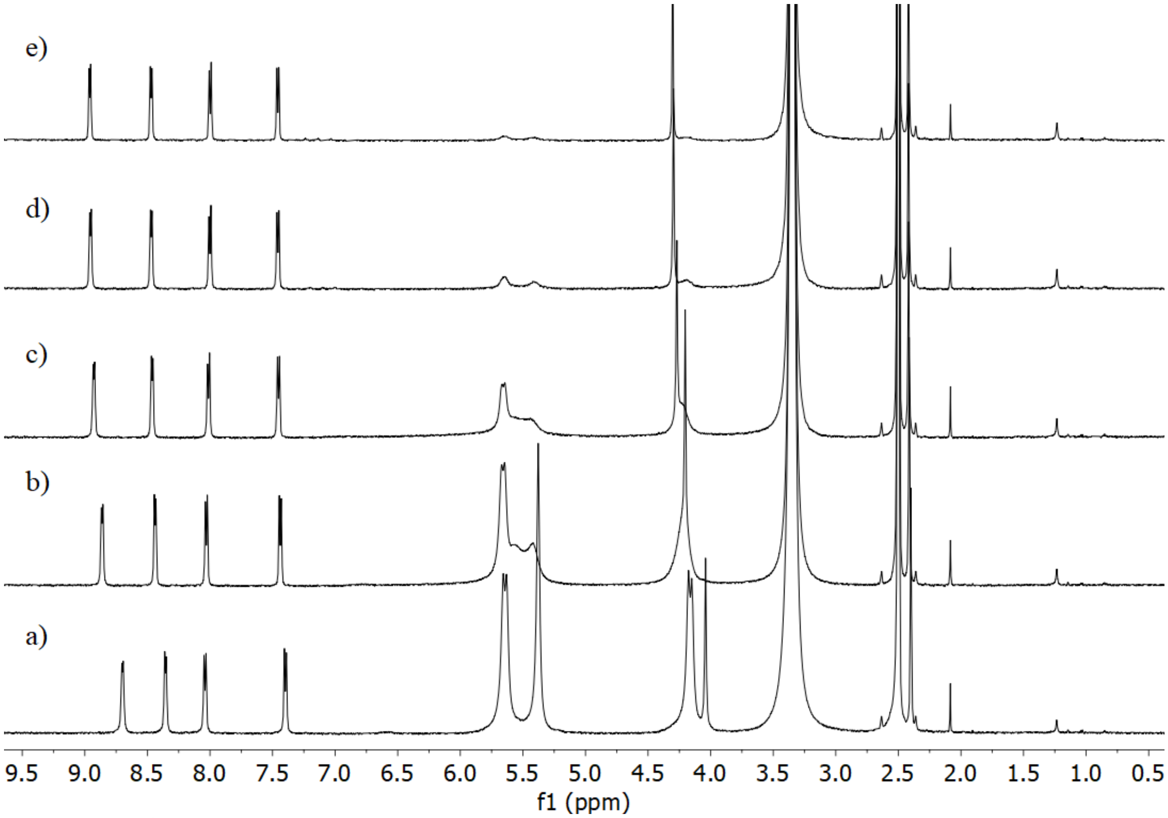

Figure S68. ^{1}H NMR spectra of a) neat **CB[7]·3** before, and b) after addition of 0.5, c) 1.0, d) 1.5 and e) 2.0 equivalents of **NaCl**.

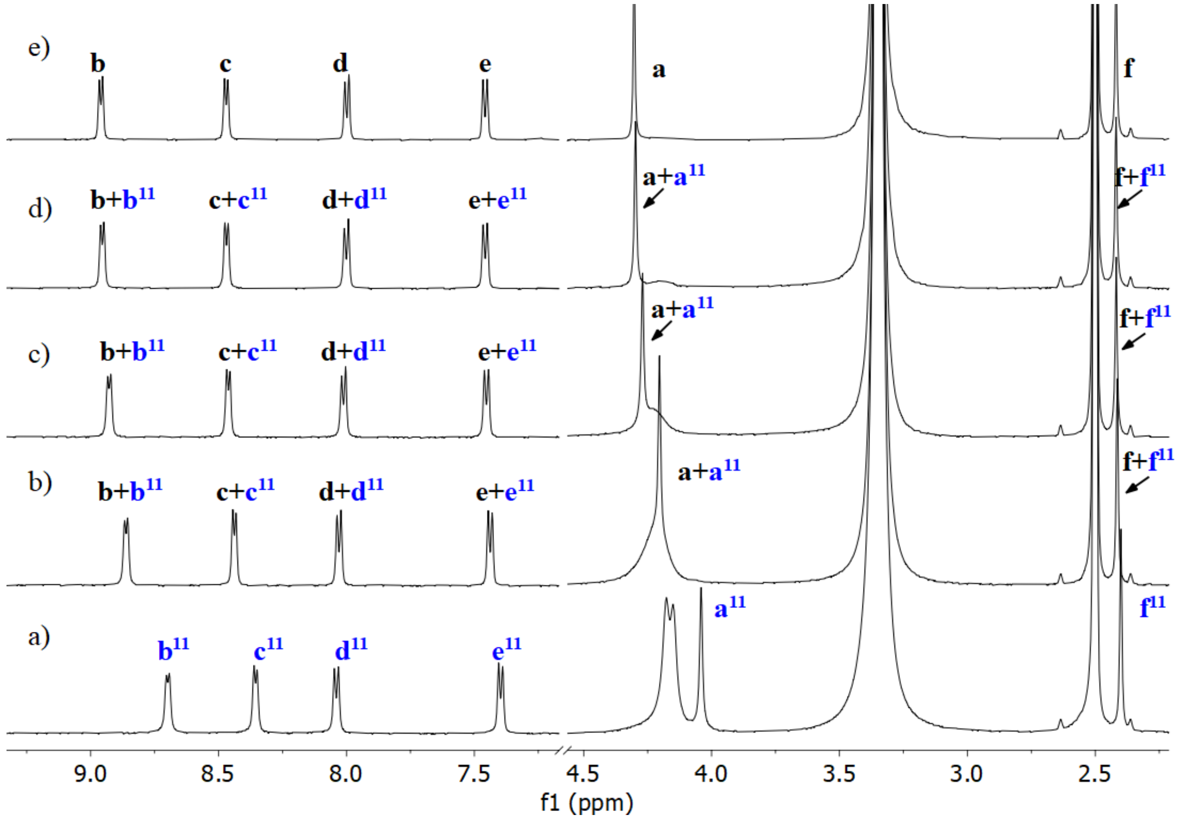

Figure S69. Aromatic and aliphatic regions of ^1H NMR spectra of a) neat **CB[7]·3** before, and b) after addition of 0.5, c) 1.0, d) 1.5 and e) 2.0 equivalents of **NaCl**.

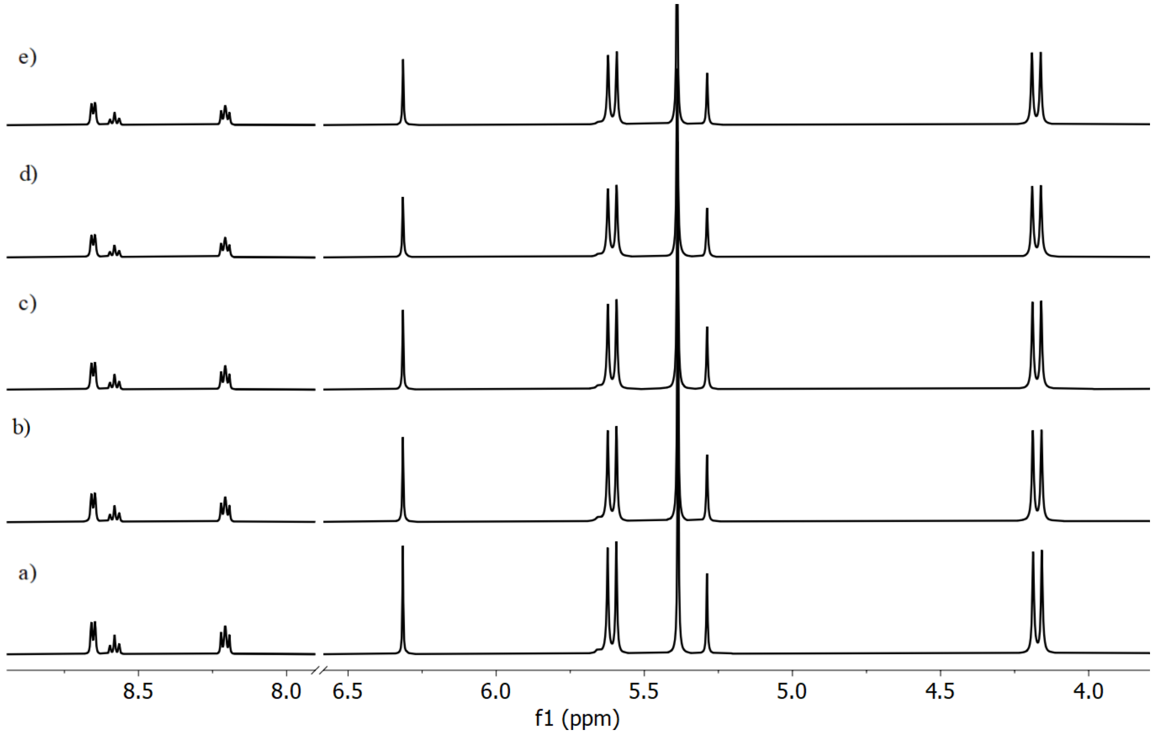

Figure S70. Aromatic and aliphatic regions of ^1H NMR spectra of: a) **CB[7]·5** (c = 1 mmol L^{-1}), and after addition of b) 0.5, c) 1.0, d) 1.5 and e) 2.0 equivalent of **NaCl** solution in DMSO-d_6.

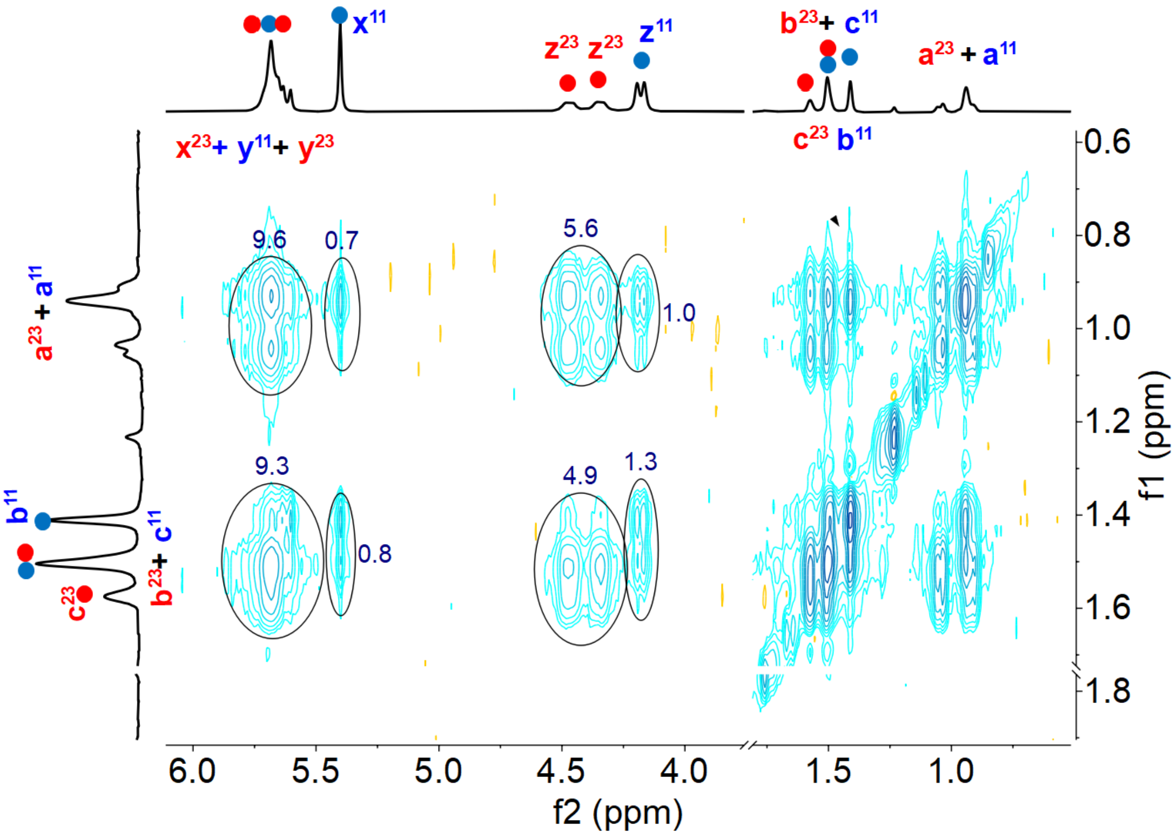

Figure S71. ^{1}H-^{1}H NOESY spectrum of a 1:1 mixture of complex CB[7]·**1** and putative aggregate (CB[7]·**1**)$_3$Na upon addition of NaCl to complex CB[7]·**1**. The correlations between adamantyl signals H_a – H_c with CB[7] signals H_x, H_y and H_z are highlighted.

4.3. ^{1}H NMR titration of [(CB[7]·1)$_3$·K]Cl with NaCl

The solution of **[(CB[7]·1)$_3$·K]Cl** was prepared by addition of potassium chloride solution in DMSO-*d*$_6$ (180 µL, 17 mmol L^{-1}) to NMR tube containing DMSO-*d*$_6$ solution of **CB[7]·1** (500 µL, 2 mmol L^{-1}). The experiment was performed by stepwise addition of NaCl solution (29 mmol L^{-1}) in DMSO-*d*$_6$ at 25 °C.

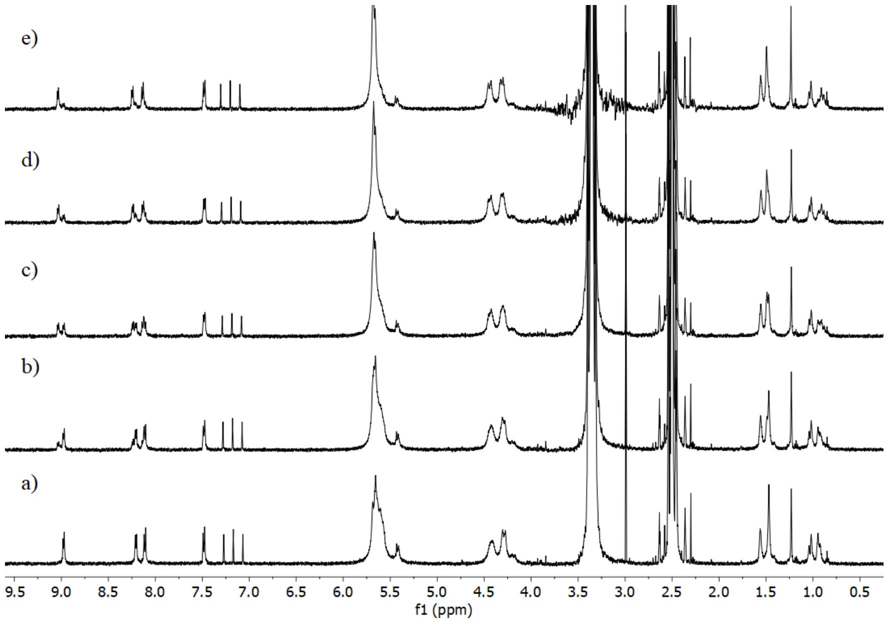

Figure S72. ^{1}H NMR spectra of **[(CB[7]·1)$_3$·K]Cl** (prepared by addition of 2 equivalents of KCl to **CB[7]·1**): a) before and b) after addition of 0.5, c) 1.0, d) 1.6 and e) 2.0 equivalents of **NaCl** in DMSO-*d*$_6$.

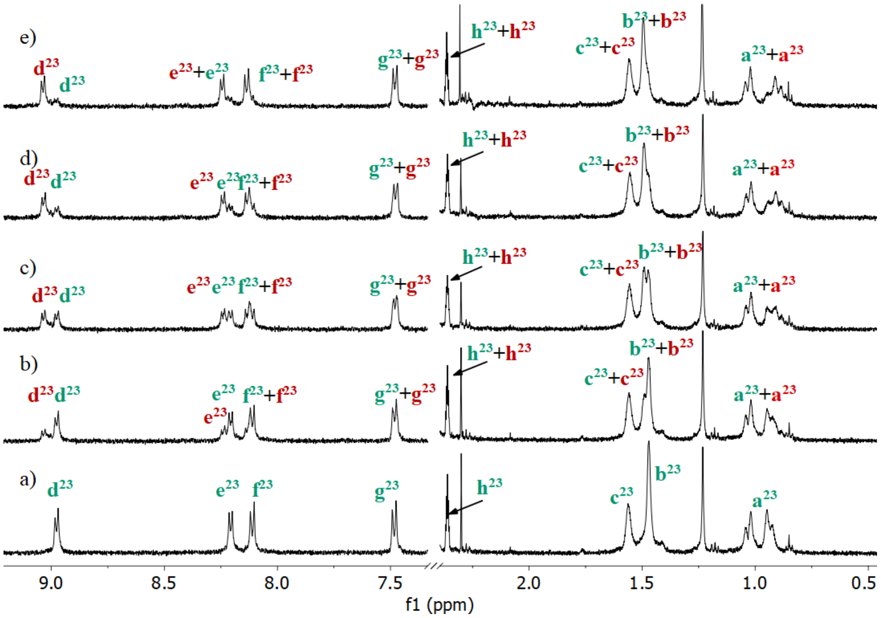

Figure S73. Aromatic and aliphatic regions of ^{1}H NMR spectra of **[(CB[7]·1)$_3$·K]Cl** (prepared by addition of 2 equivalents of KCl to **CB[7]·1**): a) before and b) after addition of 0.5, c) 1.0, d) 1.6 and e) 2.0 equivalents of **NaCl** in DMSO-*d*$_6$.

4.4 Addition of D_2O to a mixture of CB[7]·1 and [(CB[7]·1)$_3$·Na]Cl

Deuterium oxide was added portion-wise to the solution containing a mixture of **CB[7]·1** and **[(CB[7]·1)$_3$·Na]Cl** in DMSO-d_6 (V = 400 μL, c = 2 mmol L^{-1}) until the signals of the **[(CB[7]·1)$_3$·Na]Cl** were completely attenuated (Figure S73).

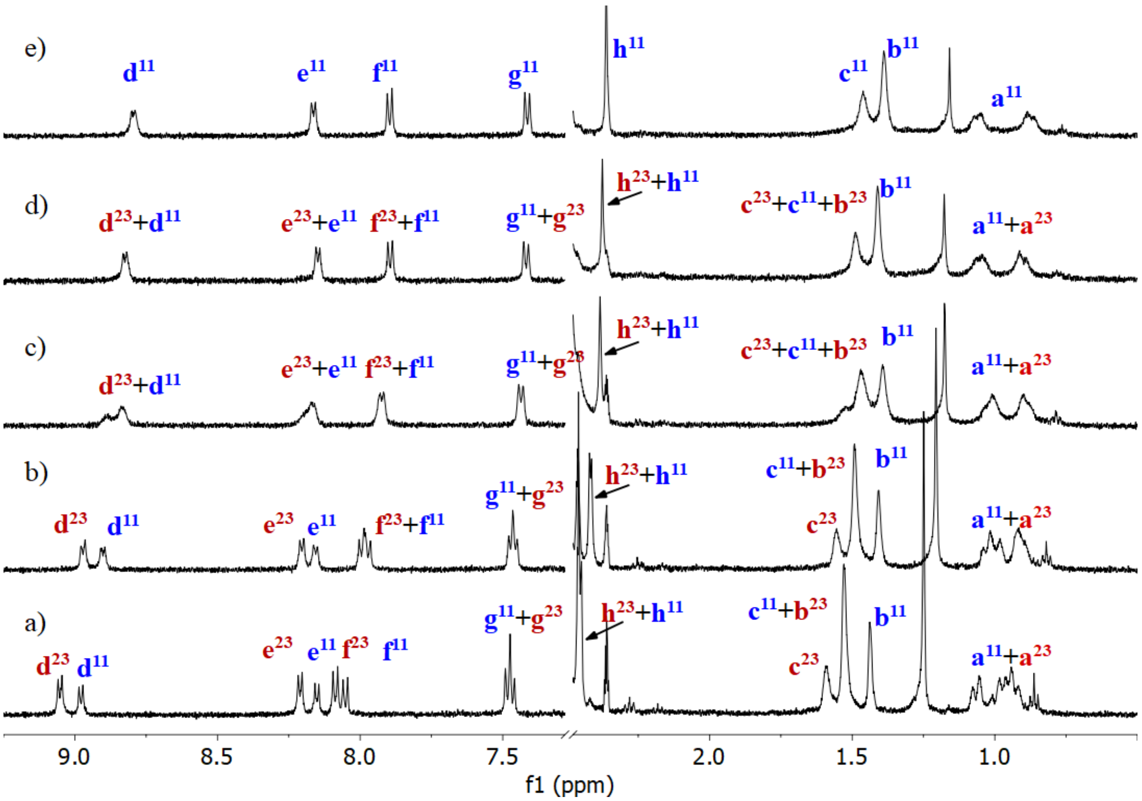

Figure S74. Aromatic and aliphatic regions of ^1H NMR spectra of **CB[7]·1** and **[(CB[7]·1)$_3$·Na]Cl** in DMSO-d_6 a) before and b) after addition of 16.7 V%; c) 33.3 V%, d) 37.5 V% and e) 44.4 V% of D_2O.

4.5 Competition experiments with 15-Crown-5

Solution of 15-Crown-5 (100 mmol L^{-1}) was added portion-wise to the solution of **CB[7]·1** in DMSO-d_6 (c = 2 mmol L^{-1}, V = 500 μL) up to the excess of 4 equivalents (Figures S74 and S75). The second experiment (Figures S76 and S77) was conducted by stepwise addition of solution containing a mixture of 15-Crown-5 and NaCl (c (NaCl) = 30 mmol L^{-1}; c (15-Crown-5) = 150 mmol L^{-1}) in DMSO-d_6 to solution of **CB[7]·1** in DMSO-d_6 (c = 2 mmol L^{-1}, V = 500 μL).

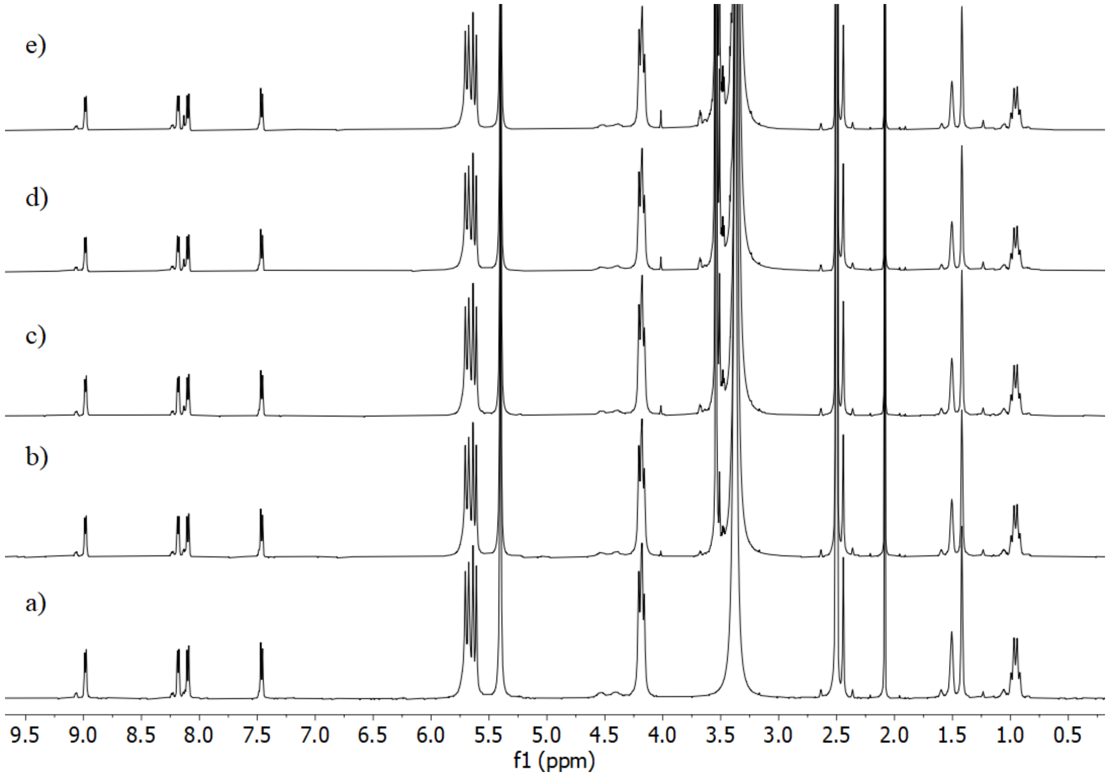

Figure S35. ^{1}H NMR spectra of a) neat **CB[7]·1** before, and b) after addition of 1.0, c) 2.0, d) 3.0 and e) 4.0 equivalents of **15-Crown-5**.

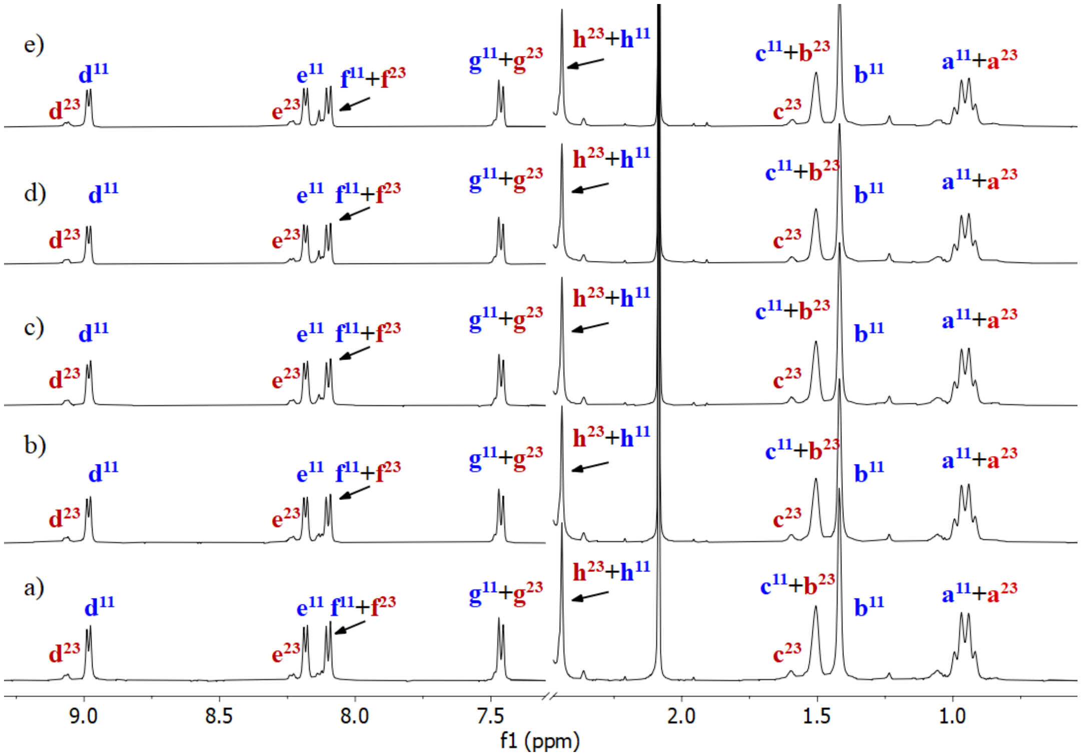

Figure S76. Aromatic and aliphatic regions of ^{1}H NMR spectra of **CB[7]·1** a) before and b) after addition 1.0, c) 2.0, d) 3.0 and e) 4.0 equivalents of **15-Crown-5**.

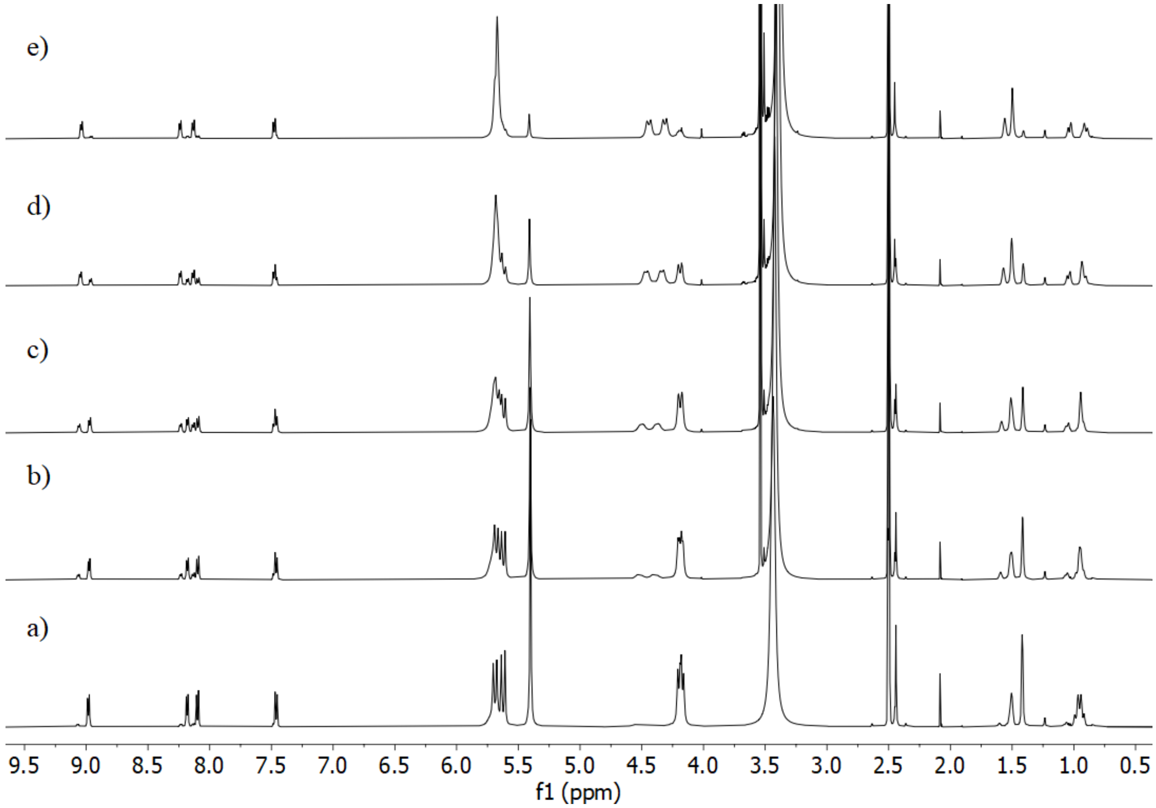

Figure S77. ^{1}H NMR spectra of a) neat **CB[7]·1** before, and b) after addition of 0.1, c) 0.2, d) 0.4 and e) 0.7 equivalents of **15-Crown-5 and NaCl mixture (5:1)**.

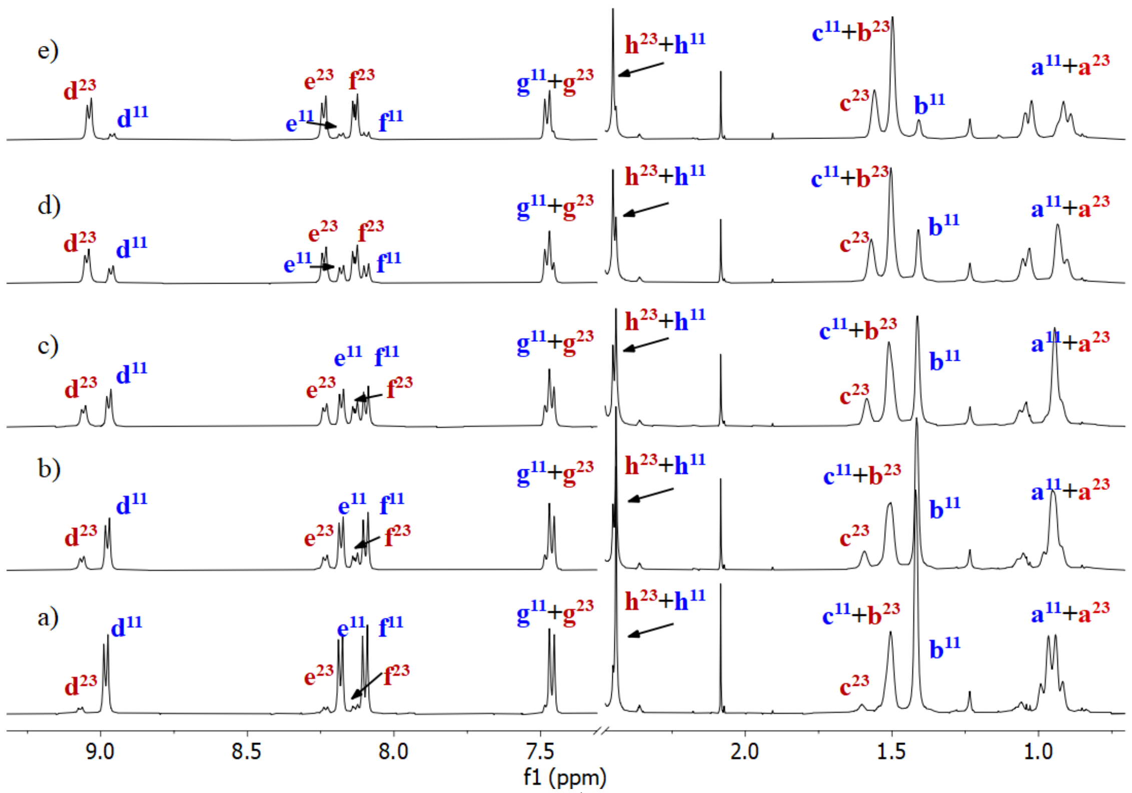

Figure S78. Aromatic and aliphatic regions of ^{1}H NMR spectra of **CB[7]·1** a) before and b) after addition 1.0, c) 2.0, d) 3.0 and e) 4.0 equivalents of **15-Crown-5 and NaCl mixture (5:1)**.

5. MS Analysis

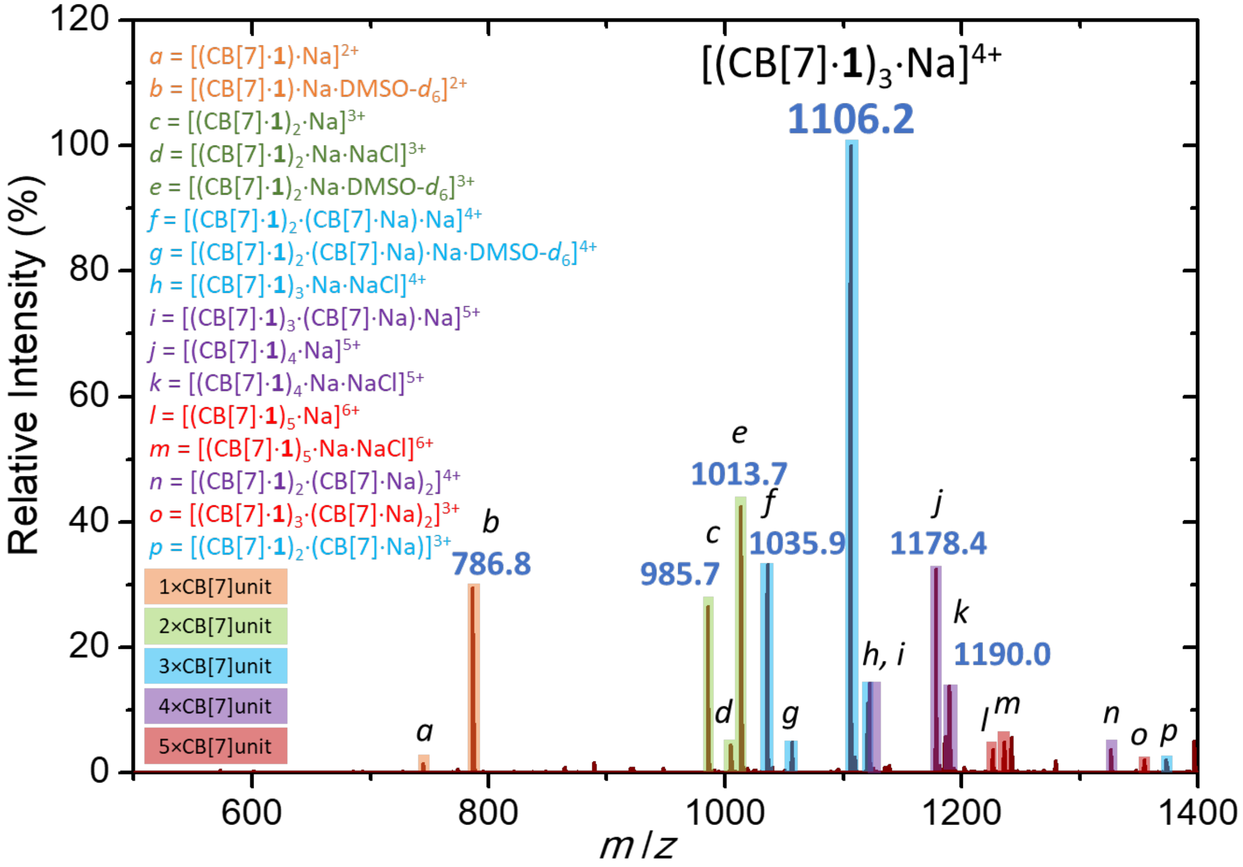

Figure S79. ESI+ MS spectrum of **[(CB[7]·1)$_3$Na]Cl** in DMSO-d_6.

MS Fragmentation Spectra

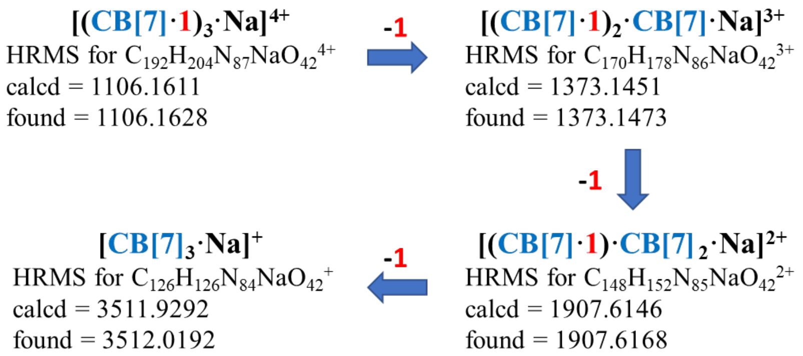

Figure S80. Schematic representation of collision-induced stepwise fragmentation of **[(CB[7]·1)$_3$·Na]$^{4+}$**.

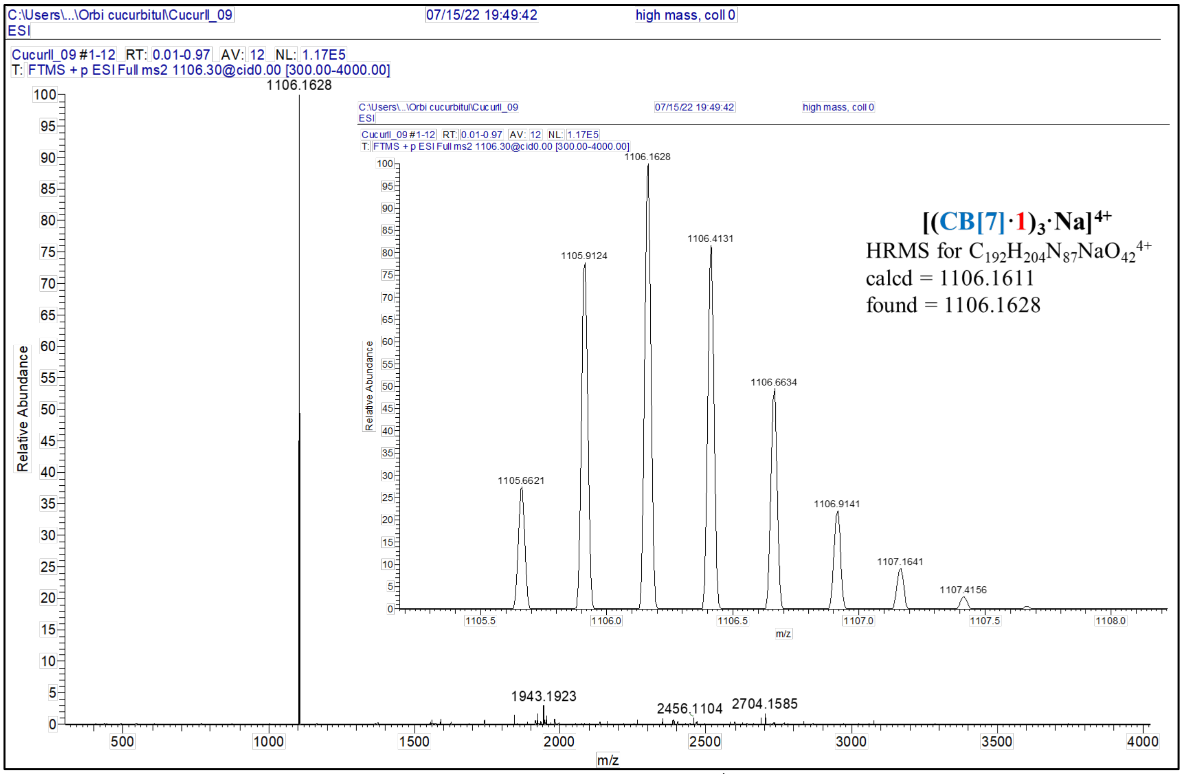

Figure S81. HRMS (ESI+) spectrum of parent **$[(CB[7]\cdot 1)_3\cdot Na]^{4+}$**.

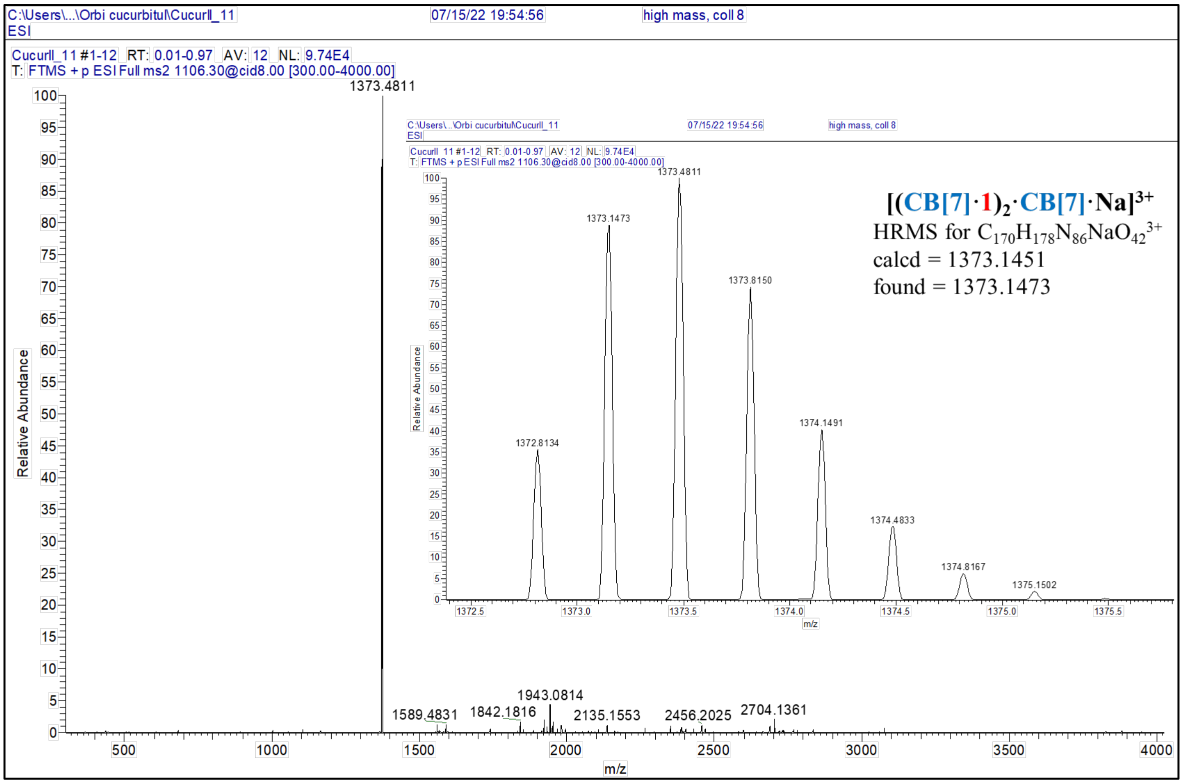

Figure S82. HRMS[2] (ESI+) spectrum showing **$[(CB[7]\cdot 1)_2\cdot CB[7]\cdot Na]^{3+}$** that is the first step of **$[(CB[7]\cdot 1)_3\cdot Na]^{4+}$** fragmentation.

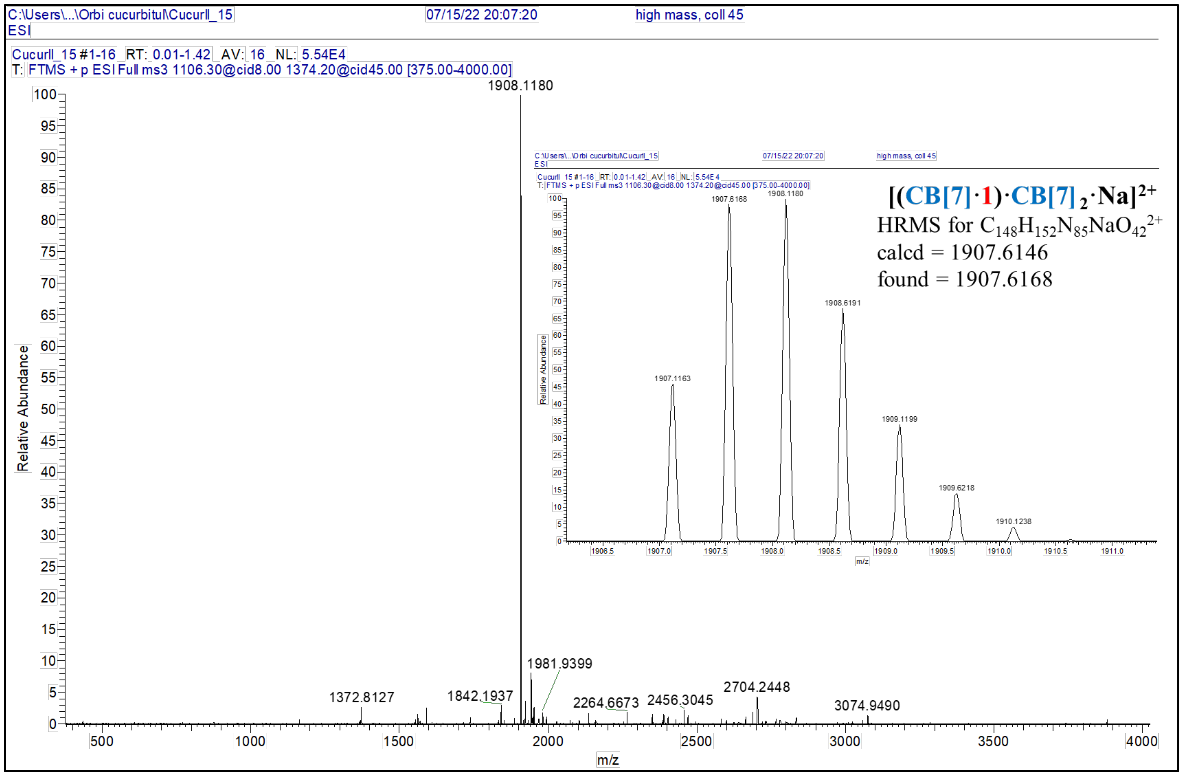

Figure S83. HRMS^{3} (ESI+) spectrum showing **[(CB[7]·1)·CB[7]$_2$·Na]$^{2+}$** that is the second step of **[(CB[7]·1)$_3$·Na]$^{4+}$** fragmentation.

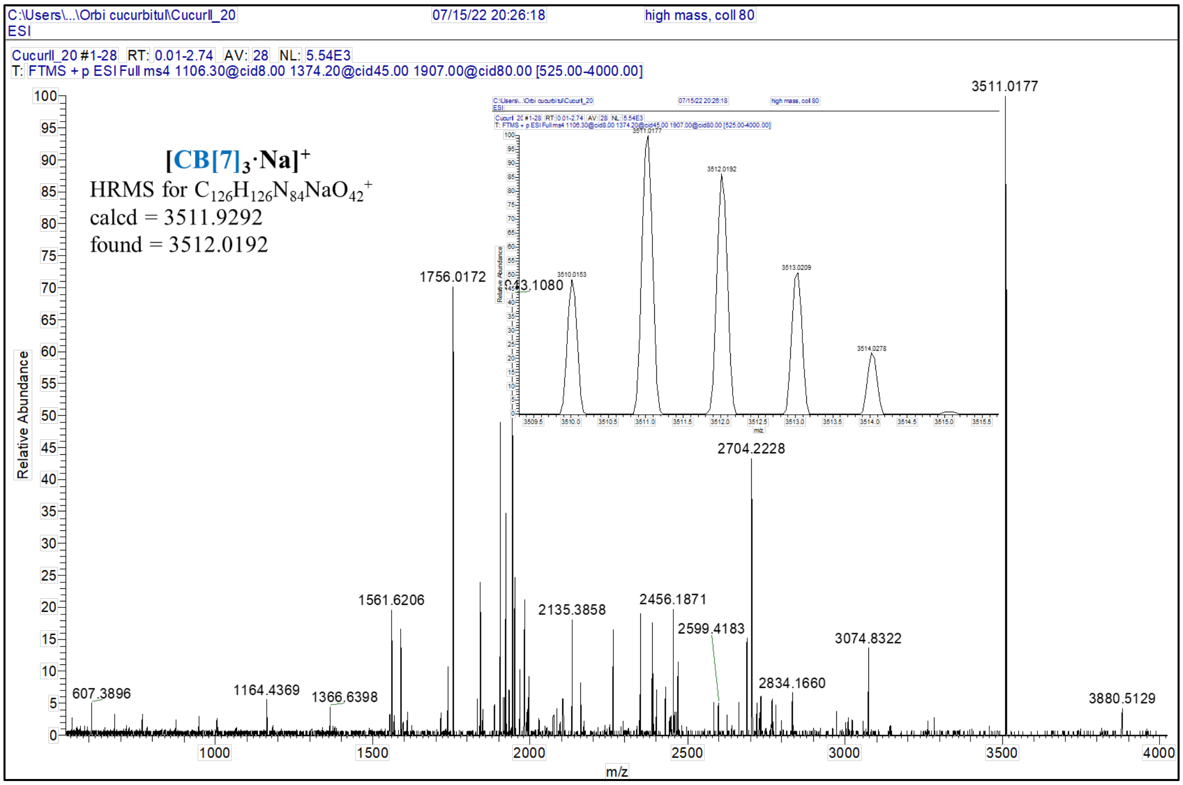

Figure S84. HRMS^{4} (ESI+) spectrum showing **[CB[7]$_3$·Na]$^{+}$** that is the third step of **[(CB[7]·1)$_3$·Na]$^{4+}$** fragmentation.

6. UV-Vis Titration Experiments

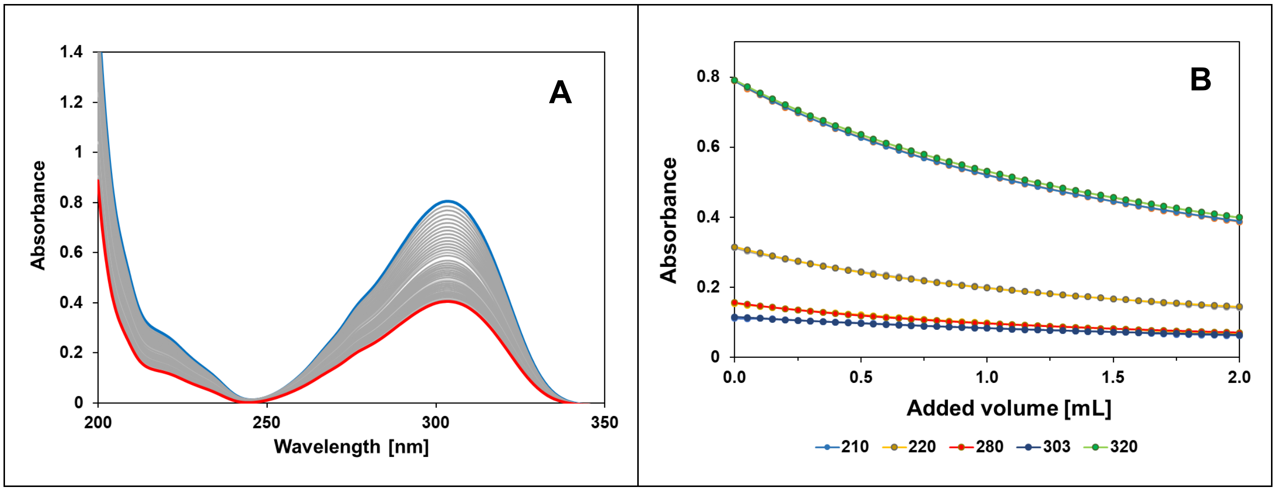

Figure S85. (**A**) UV-Vis spectra and (**B**) fitted traces for the titration of **CB[7]·1** (2.0 mL, 25 µM) with **LiCl** (10 mM) in **water**. Direction of titration: blue spectrum → red spectrum.

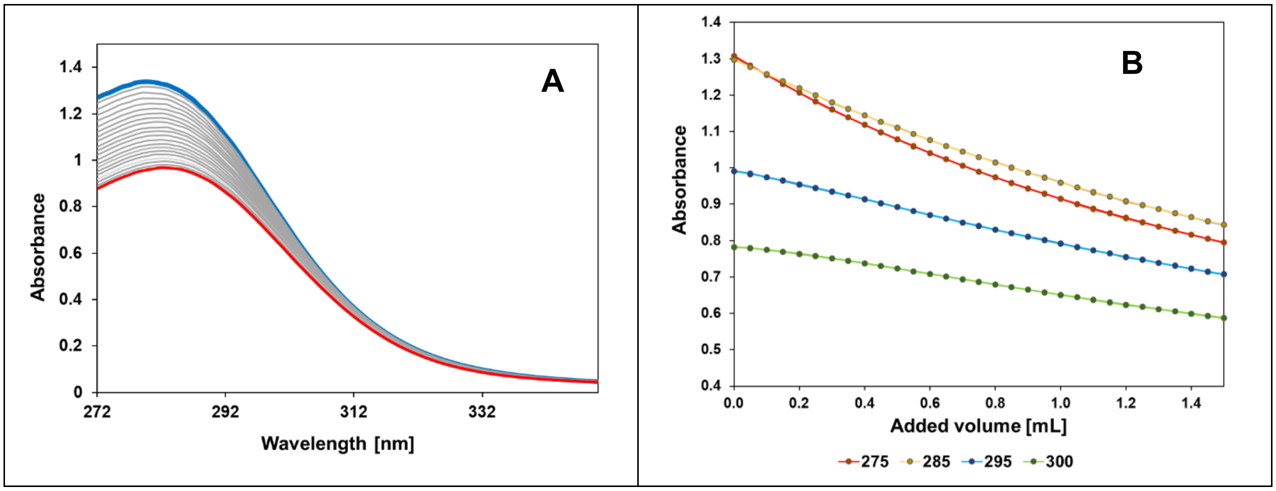

Figure S86. (**A**) UV-Vis spectra and (**B**) fitted traces for the titration of **CB[7]** (2.0 mL, 0.10 mM) with **LiCl** (10 mM) in **DMSO**. Direction of titration: blue spectrum → red spectrum.

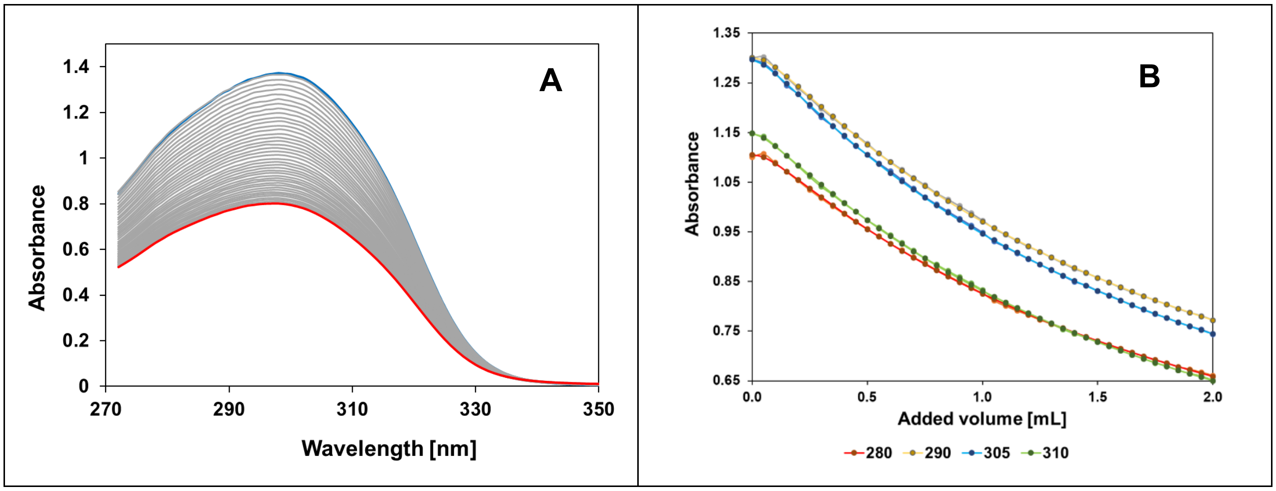

Figure S87. (**A**) UV-Vis spectra and (**B**) fitted traces for the titration of **CB[7]·1** (2.0 mL, 50 μM) with **LiCl** (10 mM) in **DMSO**. Direction of titration: blue spectrum → red spectrum.

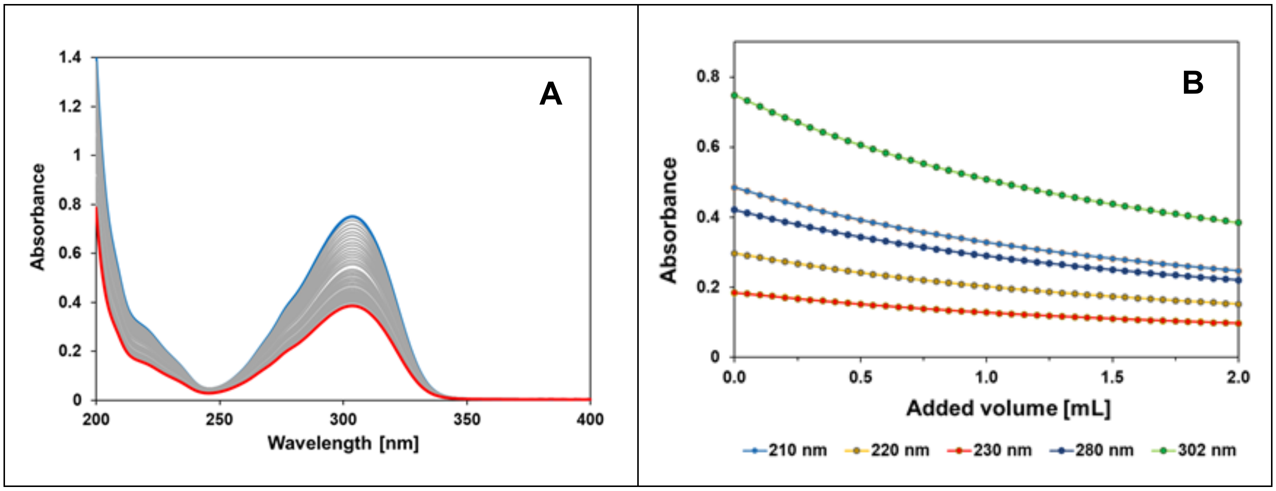

Figure S88. (**A**) UV-Vis spectra and (**B**) fitted traces for the titration of **CB[7]·1** (2.0 mL, 25 μM) with **NaCl** (10 mM) in **water**. Direction of titration: blue spectrum → red spectrum.

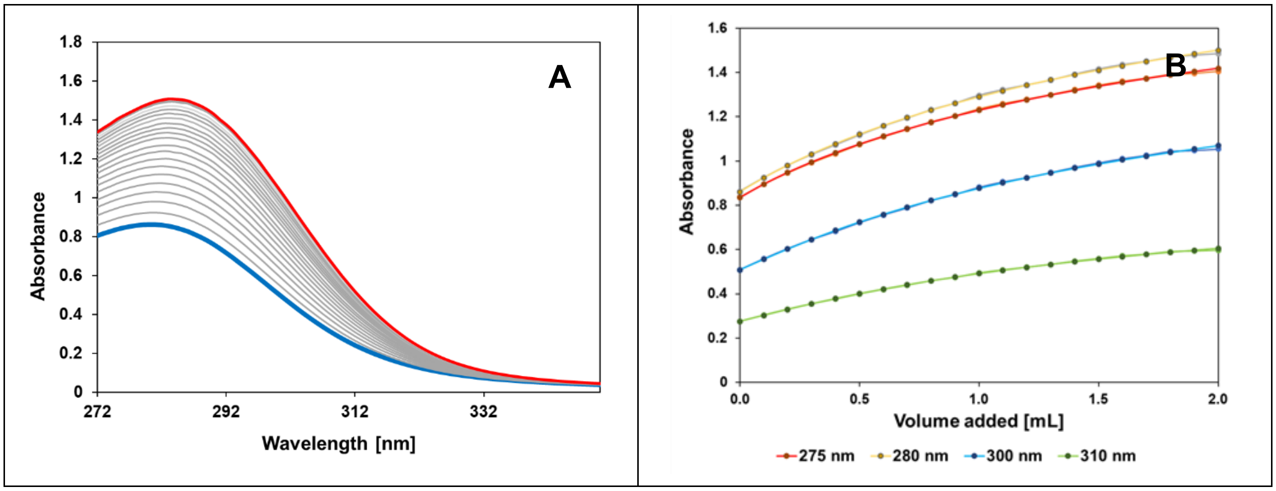

Figure S89. (**A**) UV-Vis spectra and (**B**) fitted traces for the titration of **CB[7]** (2.0 mL, 0.10 mM) with **NaCl** (10 mM) in **DMSO**. Direction of titration: blue spectrum → red spectrum.

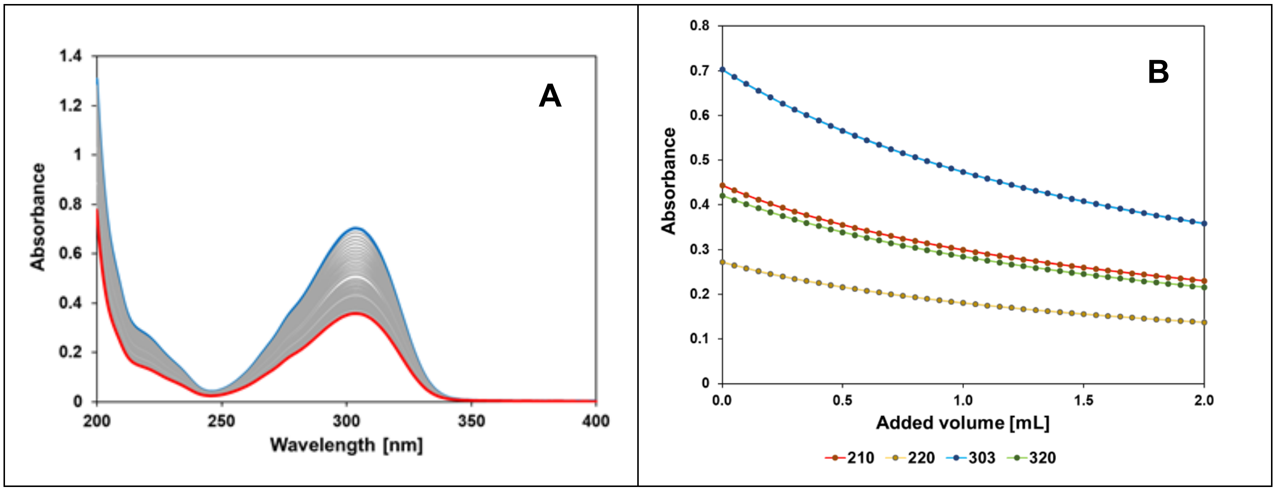

Figure S90. (**A**) UV-Vis spectra and (**B**) fitted traces for the titration of **CB[7]·1** (2.0 mL, 20 μM) with **KCl** (10 mM) in **water**. Direction of titration: blue spectrum → red spectrum.

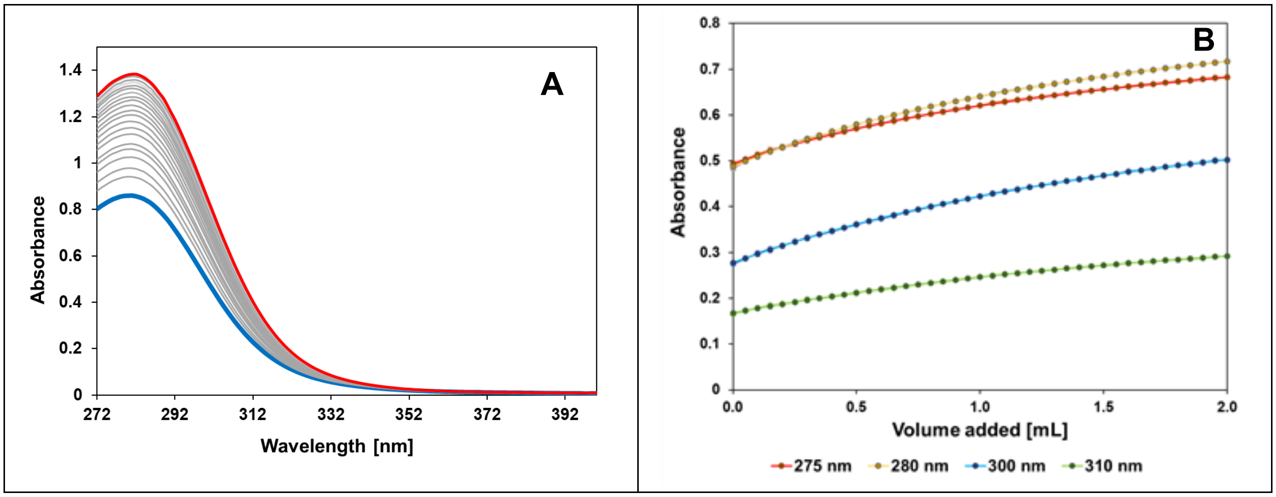

Figure S91. (**A**) UV-Vis spectra and (**B**) fitted traces for the titration of **CB[7]** (2.0 mL, 0.10 mM) with **KCl** (10 mM) in **DMSO**. Direction of titration: blue spectrum → red spectrum.

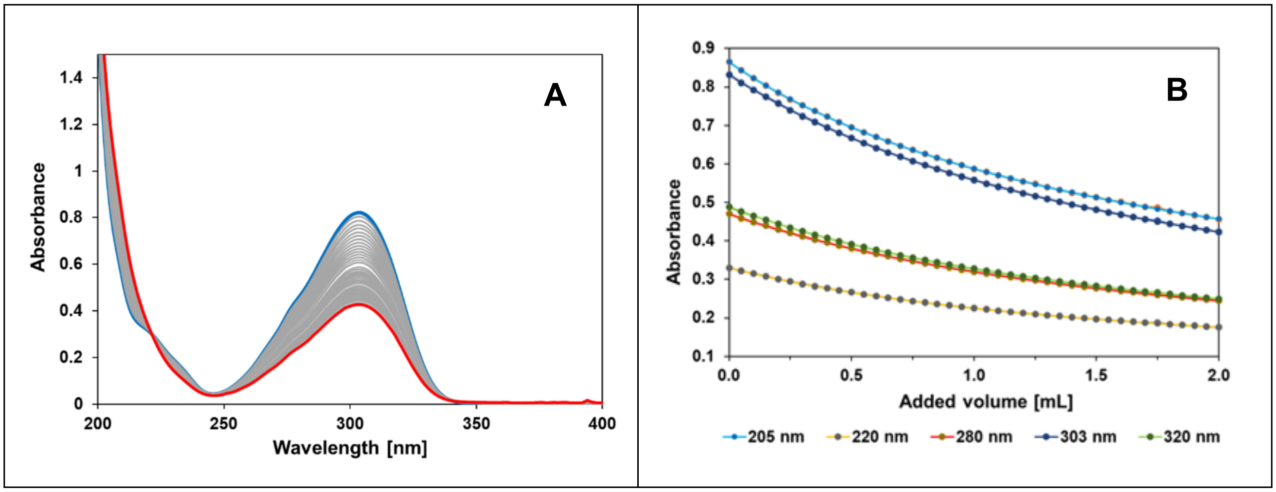

Figure S92. (**A**) UV-Vis spectra and (**B**) fitted traces for the titration of **CB[7]·1** (2.0 mL, 25 μM) with **RbCl** (10 mM) in **water**. Direction of titration: blue spectrum → red spectrum.

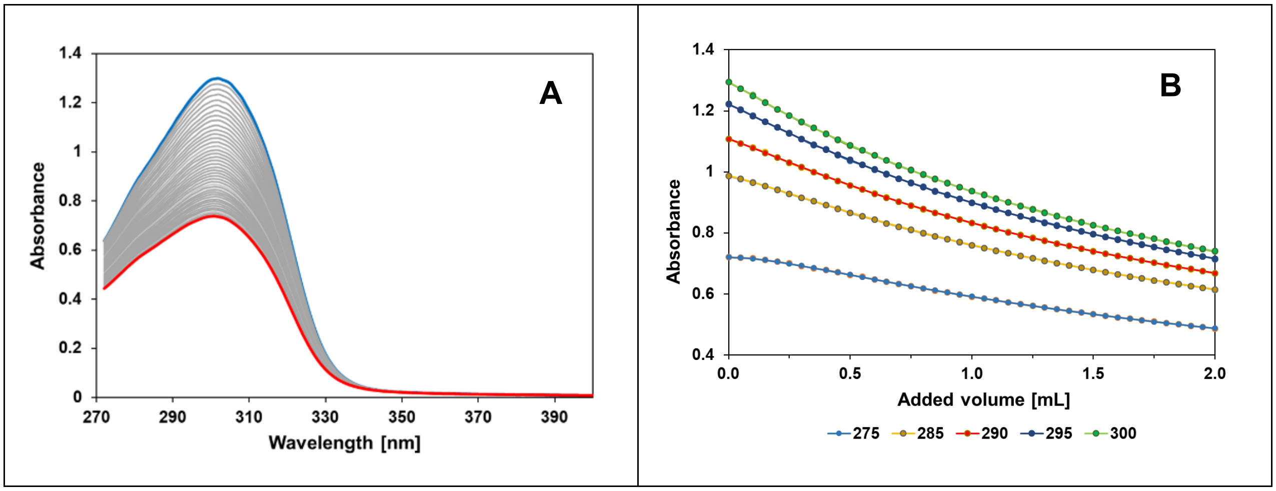

Figure S93. (**A**) UV-Vis spectra and (**B**) fitted traces for the titration of **CB[7]·1** (2.0 mL, 50 µM) with **RbCl** (10 mM) in **DMSO**. Direction of titration: blue spectrum → red spectrum.

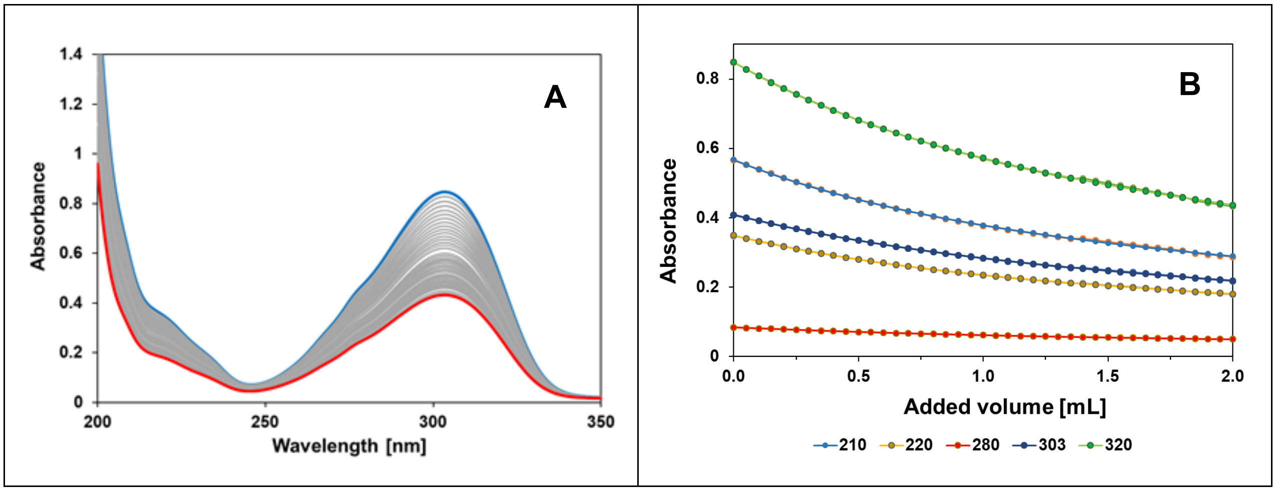

Figure S94. (**A**) UV-Vis spectra and (**B**) fitted traces for the titration of **CB[7]·1** (2.0 mL, 25 µM) with **CsCl** (10 mM) in **water**. Direction of titration: blue spectrum → red spectrum.

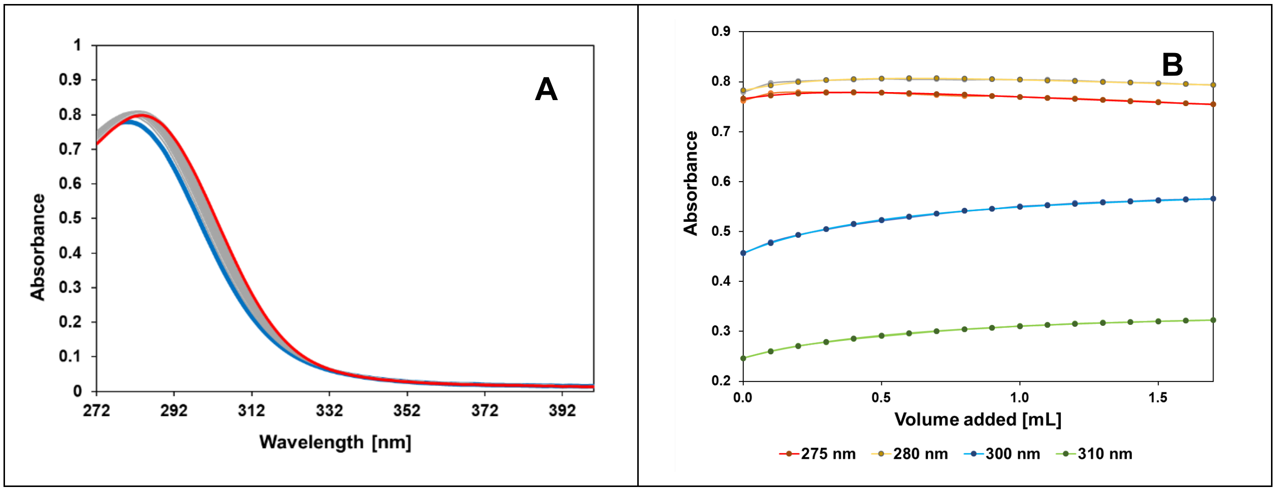

Figure S95. (**A**) UV-Vis spectra and (**B**) fitted traces for the titration of **CB[7]** (2.0 mL, 90 μM) with **CsCl** (10 mM) in **DMSO**. Direction of titration: blue spectrum → red spectrum.

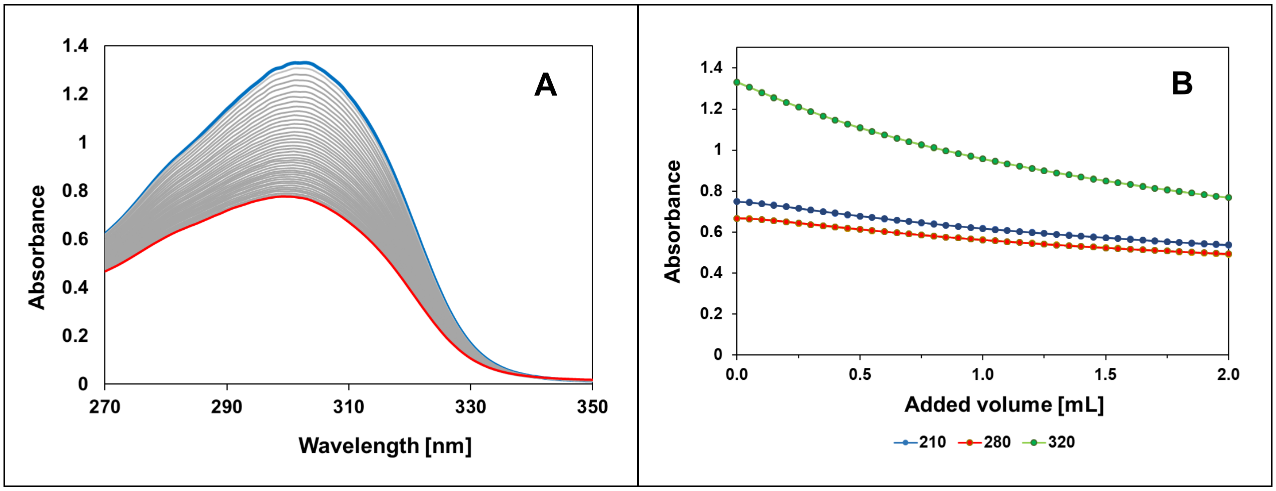

Figure S96. (**A**) UV-Vis spectra and (**B**) fitted traces for the titration of **CB[7]·1** (2.0 mL, 25 μM) with **CsCl** (10 mM) in **DMSO**. Direction of titration: blue spectrum → red spectrum.

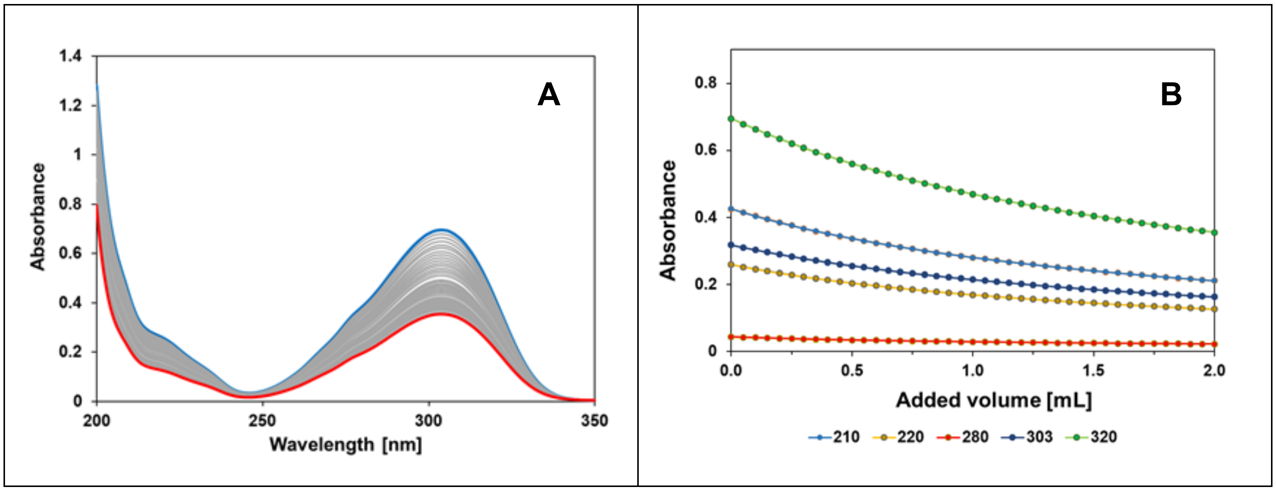

Figure S97. (**A**) UV-Vis spectra and (**B**) fitted traces for the titration of **CB[7]·1** (2.0 mL, 20 μM) with **$MgCl_2$** (10 mM) in **water**. Direction of titration: blue spectrum → red spectrum.

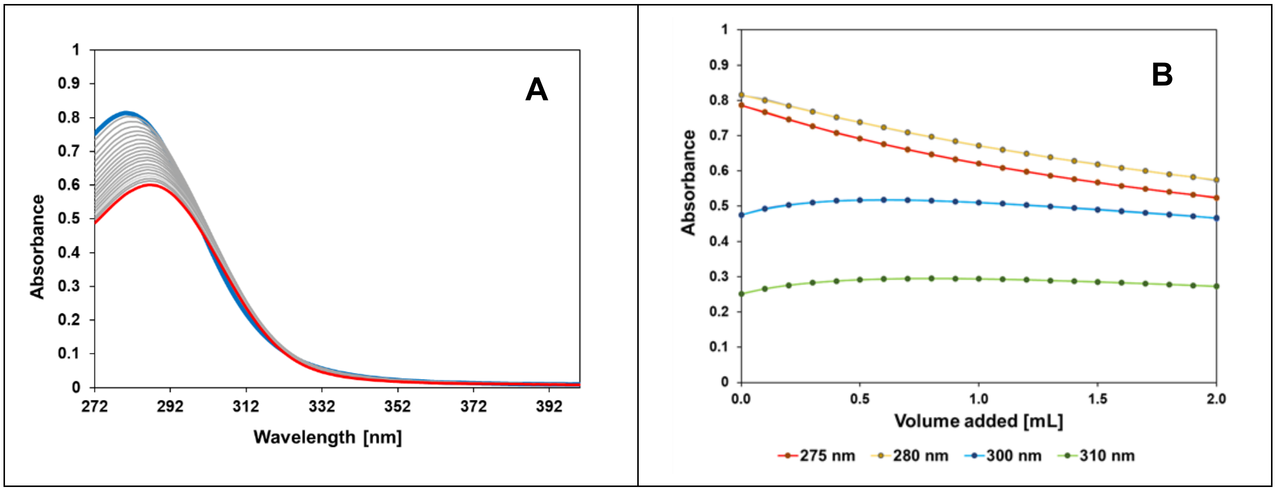

Figure S98. (**A**) UV-Vis spectra and (**B**) fitted traces for the titration of **CB[7]** (2.0 mL, 60 μM) with **$MgCl_2$** (10 mM) in **DMSO**. Direction of titration: blue spectrum → red spectrum.

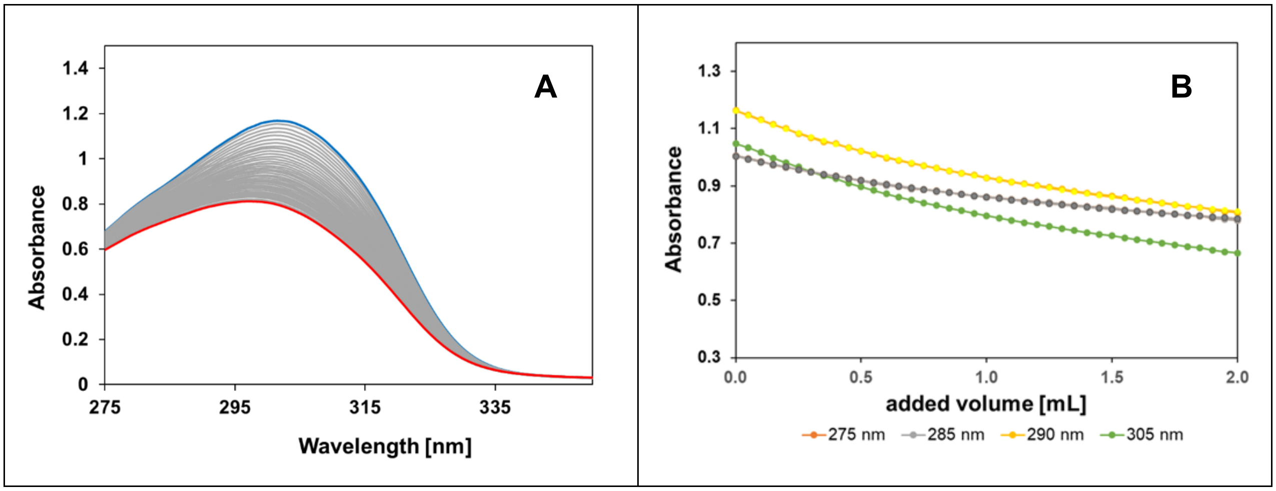

Figure S99. (**A**) UV-Vis spectra and (**B**) fitted traces for the titration of **CB[7]·1** (2.0 mL, 30 μM) with **$MgCl_2$** (10 mM) in **DMSO**. Direction of titration: blue spectrum → red spectrum.

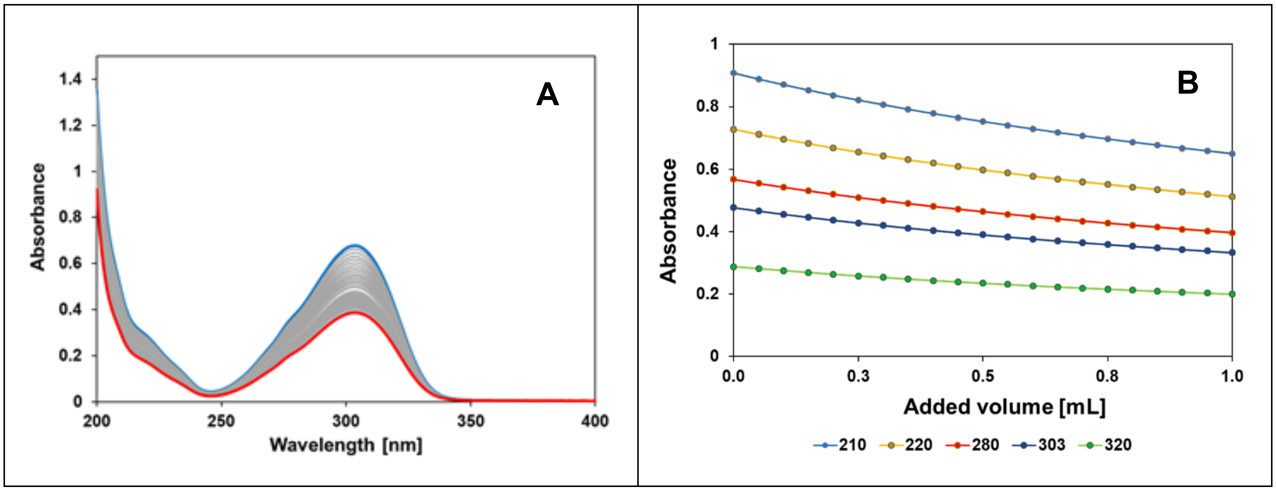

Figure S100. (**A**) UV-Vis spectra and (**B**) fitted traces for the titration of **CB[7]·1** (2.0 mL, 20 μM) with **$CaCl_2$** (10 mM) in **water**. Direction of titration: blue spectrum → red spectrum.

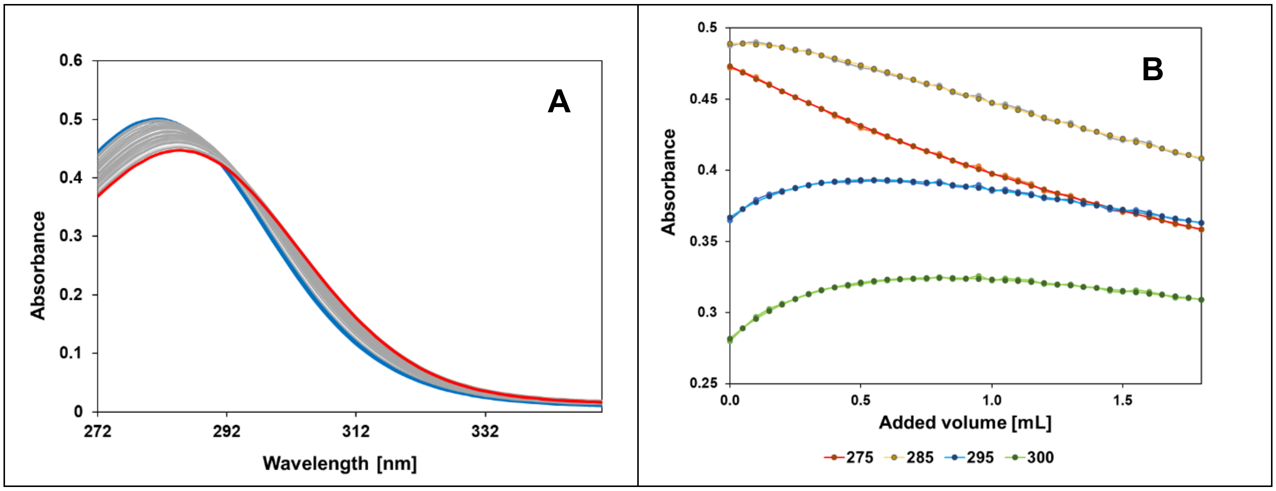

Figure S101. (**A**) UV-Vis spectra and (**B**) fitted traces for the titration of **CB[7]** (2.0 mL, 50 μM) with **$CaCl_2$** (10 mM) in **DMSO**. Direction of titration: blue spectrum → red spectrum.

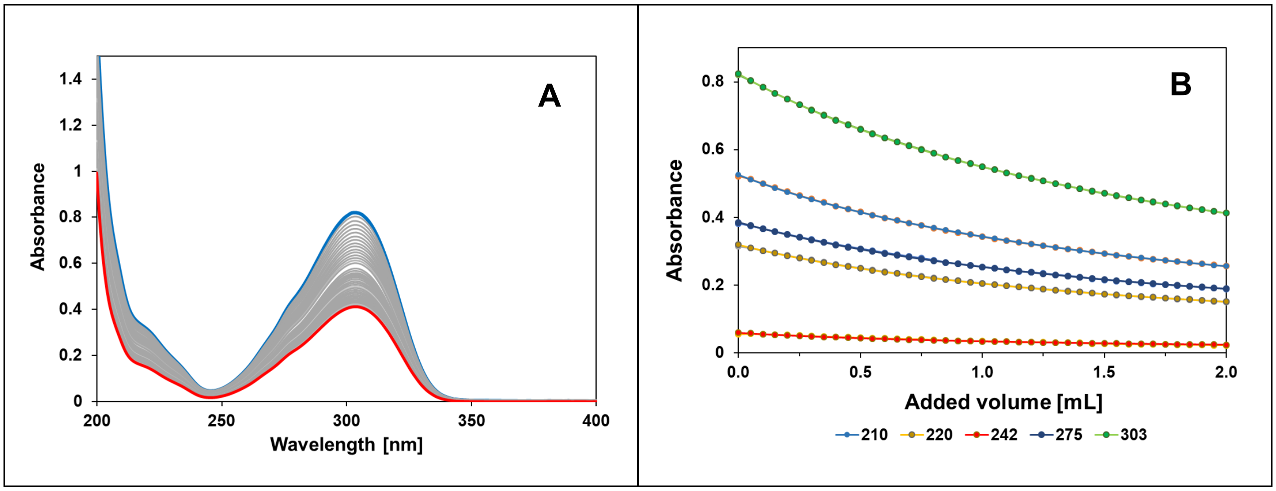

Figure S102. (**A**) UV-Vis spectra and (**B**) fitted traces for the titration of **CB[7]·1** (2.0 mL, 25 μM) with **$SrCl_2$** (10 mM) in **water**. Direction of titration: blue spectrum → red spectrum.

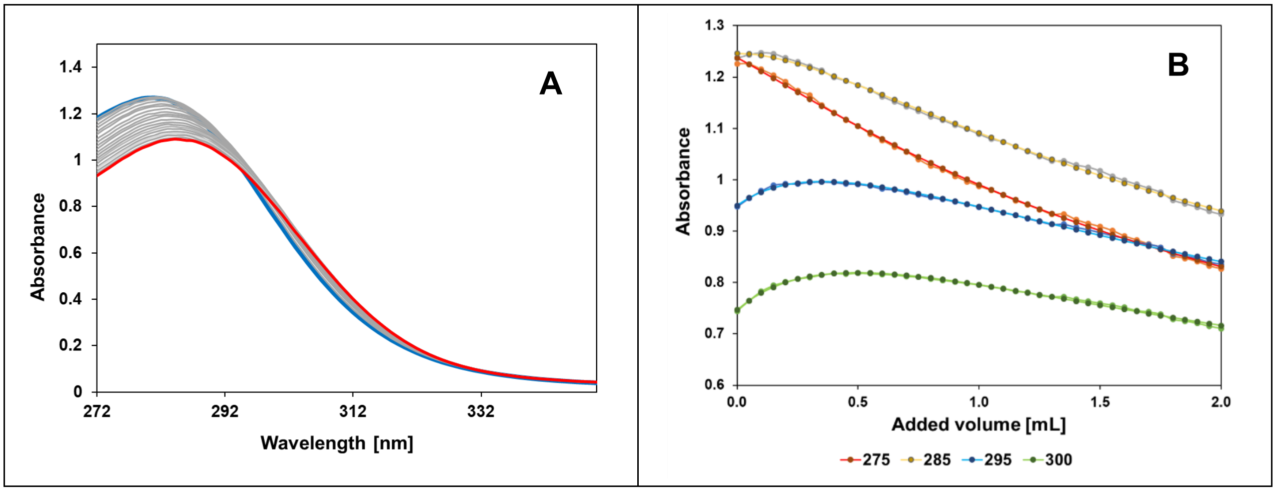

Figure S103. (**A**) UV-Vis spectra and (**B**) fitted traces for the titration of **CB[7]** (2.0 mL, 0.10 mM) with **$SrCl_2$** (10 mM) in **DMSO**. Direction of titration: blue spectrum → red spectrum.

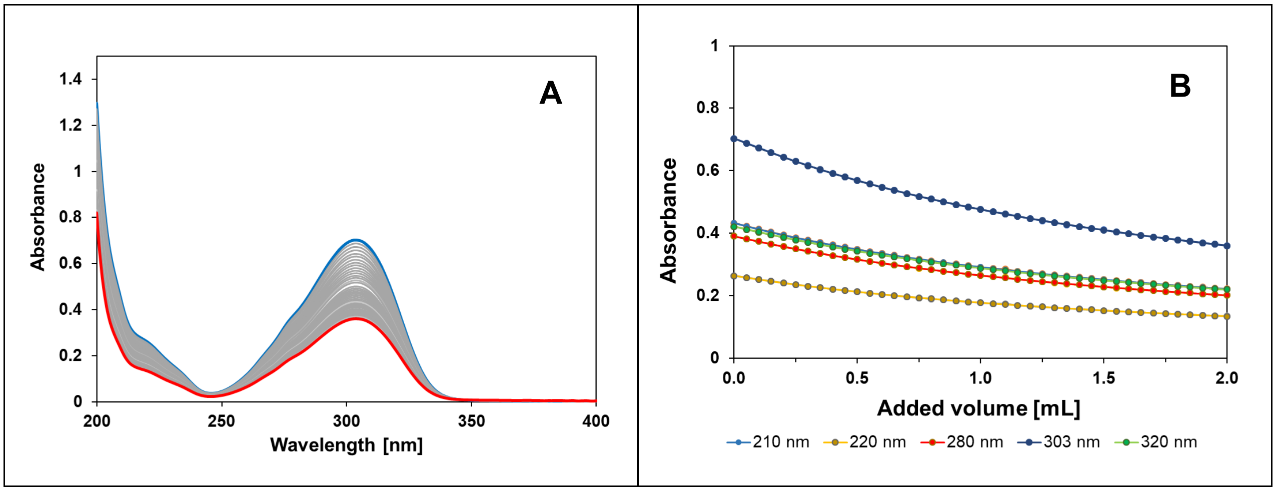

Figure S104. (**A**) UV-Vis spectra and (**B**) fitted traces for the titration of **CB[7]·1** (2.0 mL, 20 μM) with **$BaCl_2$** (10 mM) in **water**. Direction of titration: blue spectrum → red spectrum.

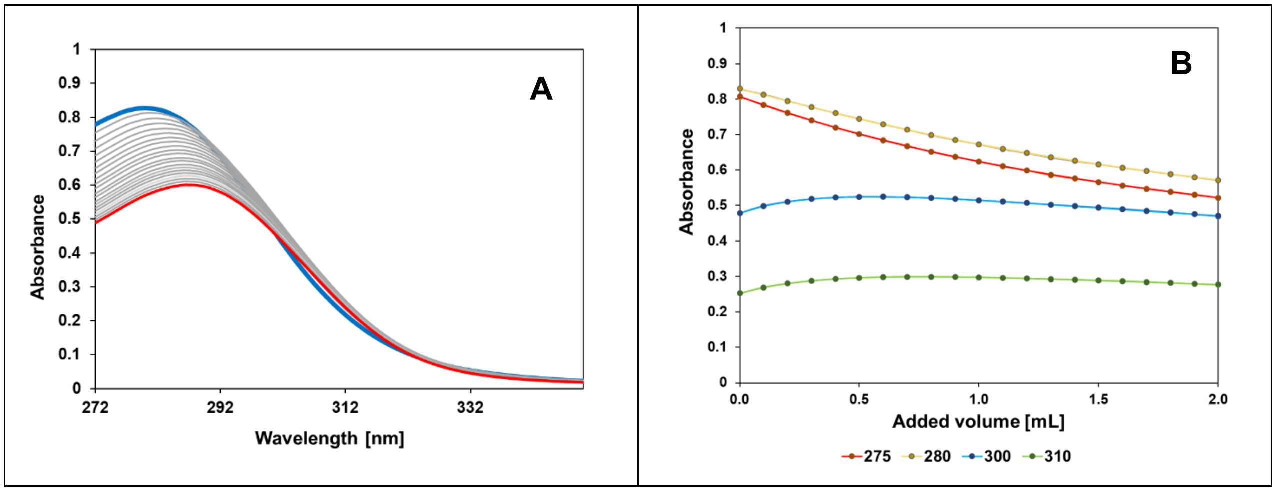

Figure S105. (**A**) UV-Vis spectra and (**B**) fitted traces for the titration of **CB[7]** (2.0 mL, 60 μM) with **BaCl$_2$** (10 mM) in **DMSO**. Direction of titration: blue spectrum → red spectrum.

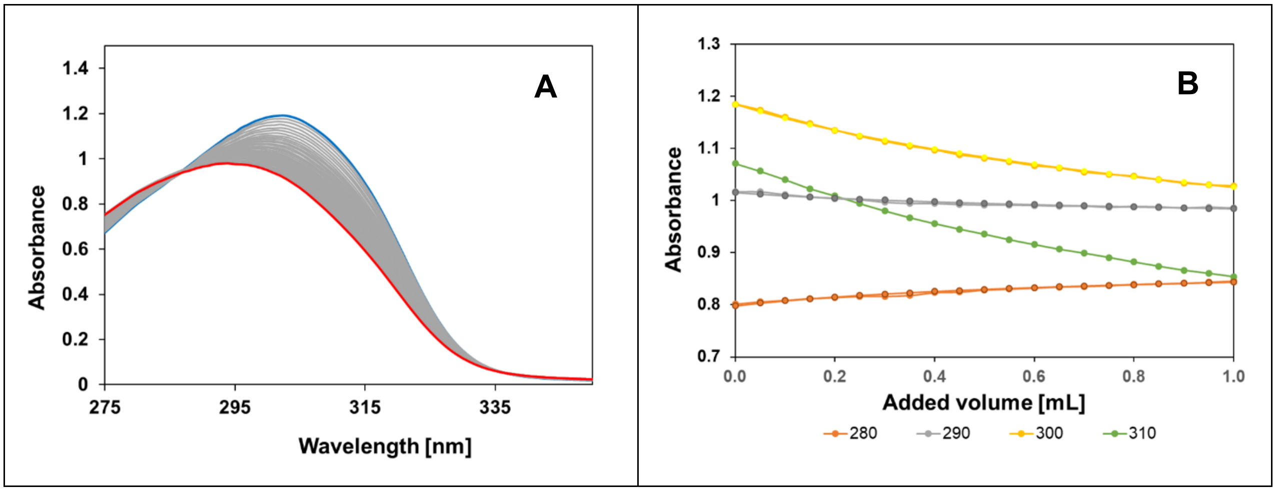

Figure S106. (**A**) UV-Vis spectra and (**B**) fitted traces for the titration of **CB[7]·1** (2.0 mL, 40 μM) with **BaCl$_2$** (10 mM) in **DMSO**. Direction of titration: blue spectrum → red spectrum.

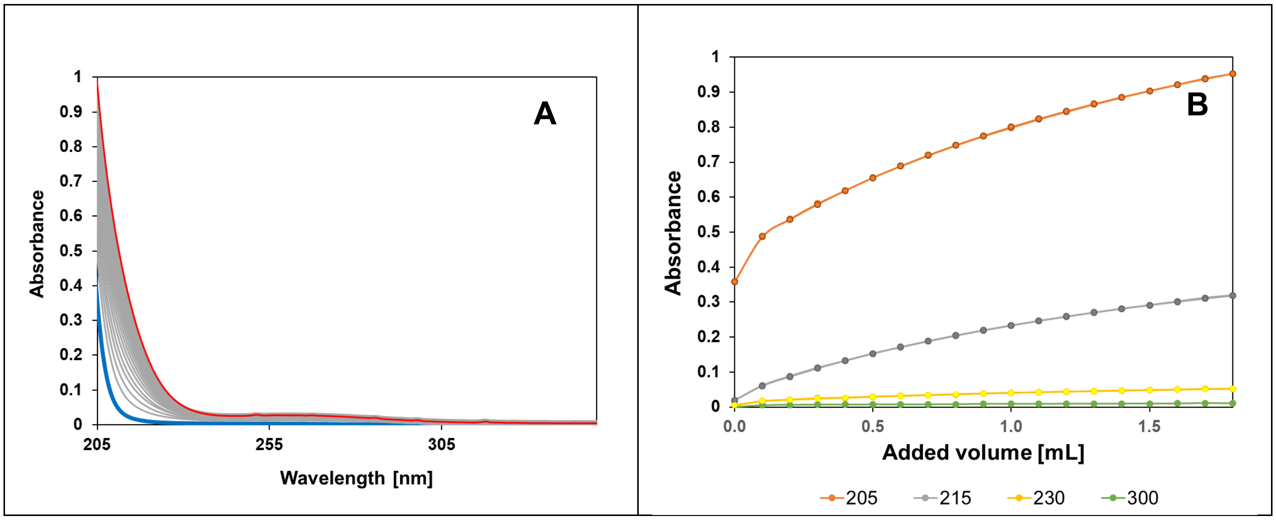

Figure S107. (**A**) UV-Vis spectra and (**B**) fitted traces for the titration of **CB[7]** (2.0 mL, 10 μM) with **$EuCl_3$** (10 mM) in **water**. Direction of titration: blue spectrum → red spectrum.

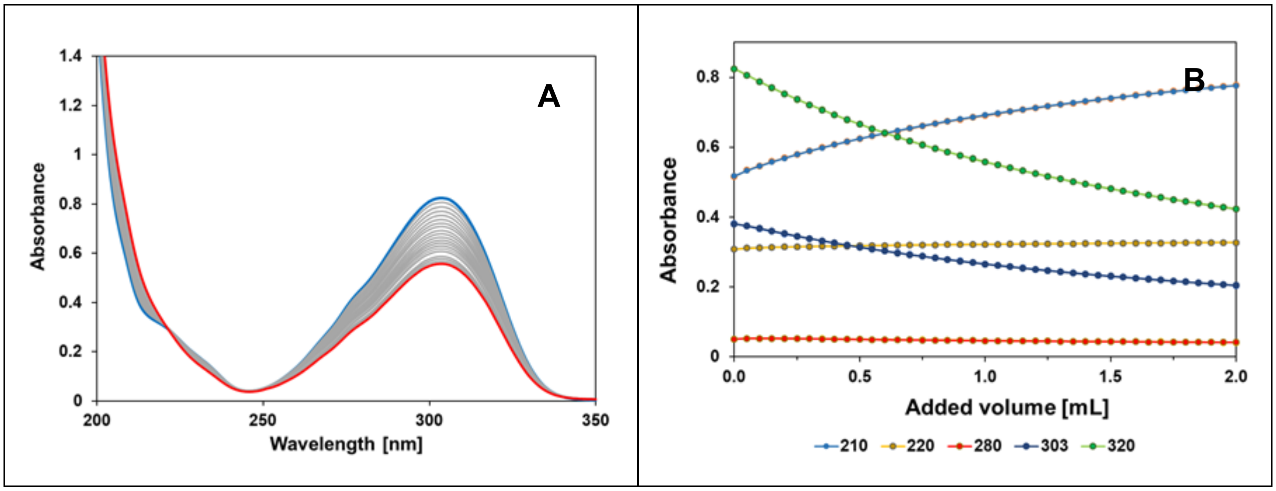

Figure S108. (**A**) UV-Vis spectra and (**B**) fitted traces for the titration of **CB[7]·1** (2.0 mL, 25 μM) with **$EuCl_3$** (10 mM) in **water**. Direction of titration: blue spectrum → red spectrum.

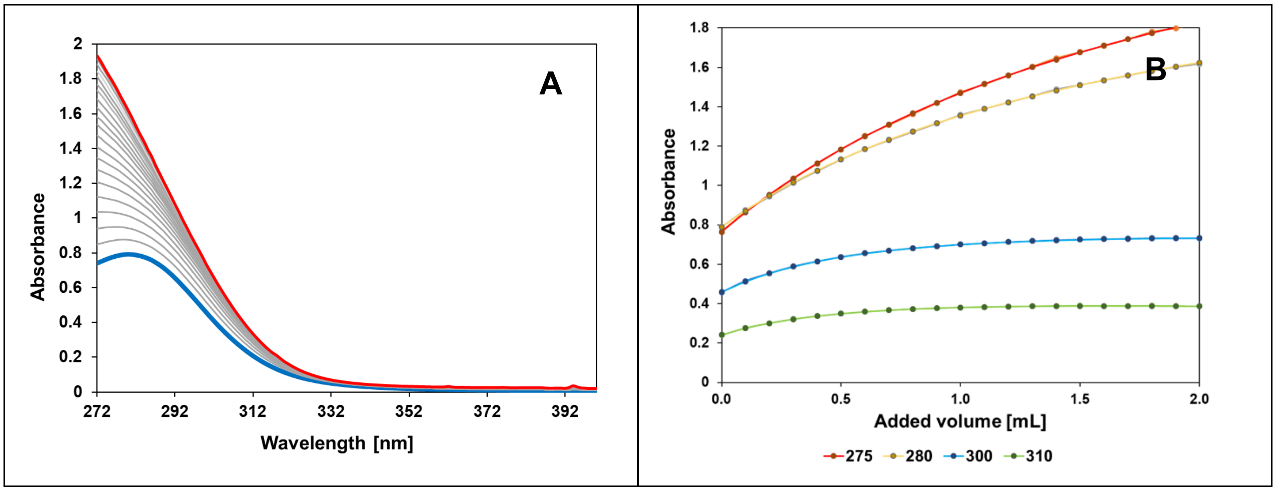

Figure S109. (**A**) UV-Vis spectra and (**B**) fitted traces for the titration of **CB[7]** (2.0 mL, 0.10 mM) with **EuCl$_3$** (10 mM) in **DMSO**. Direction of titration: blue spectrum → red spectrum.

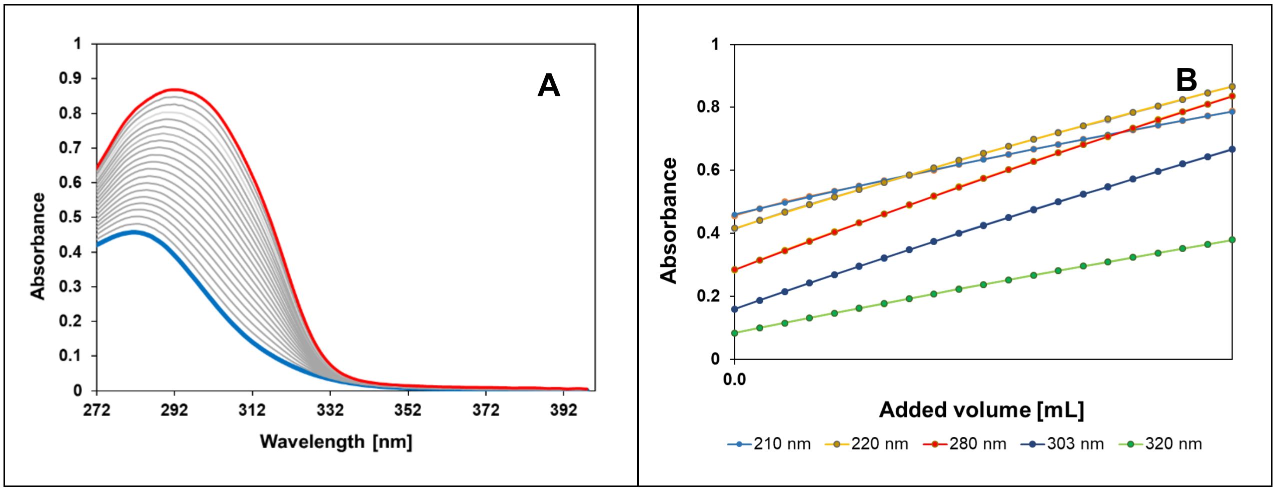

Figure S110. (**A**) UV-Vis spectra and (**B**) fitted traces for the titration of **CB[7]** (2.0 mL, 25 µM) with guest **1** (0.25 mM) in **DMSO**. Direction of titration: blue spectrum → red spectrum.

7. X-ray Crystallographic Data

Crystallographic data for **1**, **2**, **3** and **7** were collected on Bruker D8 VENTURE Kappa Duo PHOTONIII by IµS micro-focus sealed tube either MoKα (λ= 0.71073 Å) (**2** and **3)** or CuKα (λ = 1.54178 Å) (**1** and **7**) at low temperature. The structures were solved by direct methods (XT)[1] and refined by full matrix least squares based on F^2 (SHELXL2018).[2] The hydrogen atoms on carbon were fixed into idealized positions (riding model) and assigned temperature factors either $H_{iso}(H) = 1.2\ U_{eq}$(pivot atom) or $H_{iso}(H) = 1.5\ U_{eq}$ (pivot atom) for methyl moiety. The results of structure determination of **1**, **2** and **3** exhibit standard level of precision, however the results of **7** were hampered by the poor quality of the single crystal farther complicated by non-merohedral twinning. The orientation of displacement ellipsoids with their longest axis parallel to **c** - lattice vector are also suggesting a presence of stacking fault in a row of molecules.

Table S1 Parameters of Single Crystals of **1**, **2**, **3**, and **7**.

Cmpd.	Crystal System	Space Group	Cell Lengths (Å)	Cell Angles (°)
**1**	monoclinic	P 2_1/n	*a* 18.5283(4)	*α* 90
			b 9.7746(2)	*β* 98.9010(10)
			c 20.5000(4)	*γ* 90
**2**	triclinic	P 1	*a* 10.1283(6)	*α* 105.878(2)
			b 15.0534(8)	*β* 105.252(2)
			c 15.6108(9)	*γ* 108.949(2)
**3**	monoclinic	P 2_1/c	*a* 13.0447(10)	*α* 90
			b 8.5595(6)	*β* 112.440(2)
			c 11.9652(8)	*γ* 90
**7**	monoclinic	P 2_1/c	*a* 21.5911(16)	*α* 90
			b 10.9752(7)	*β* 93.449(4)
			c 7.7041(5)	*γ* 90

1. SHELXT: Sheldrick, G.M. (2015). *Acta Cryst.* **A71**, 3-8.
2. SHELXL: Sheldrick, G.M. (2015). *Acta Cryst.* **C71**, 3-8.

7.1. Compound 1

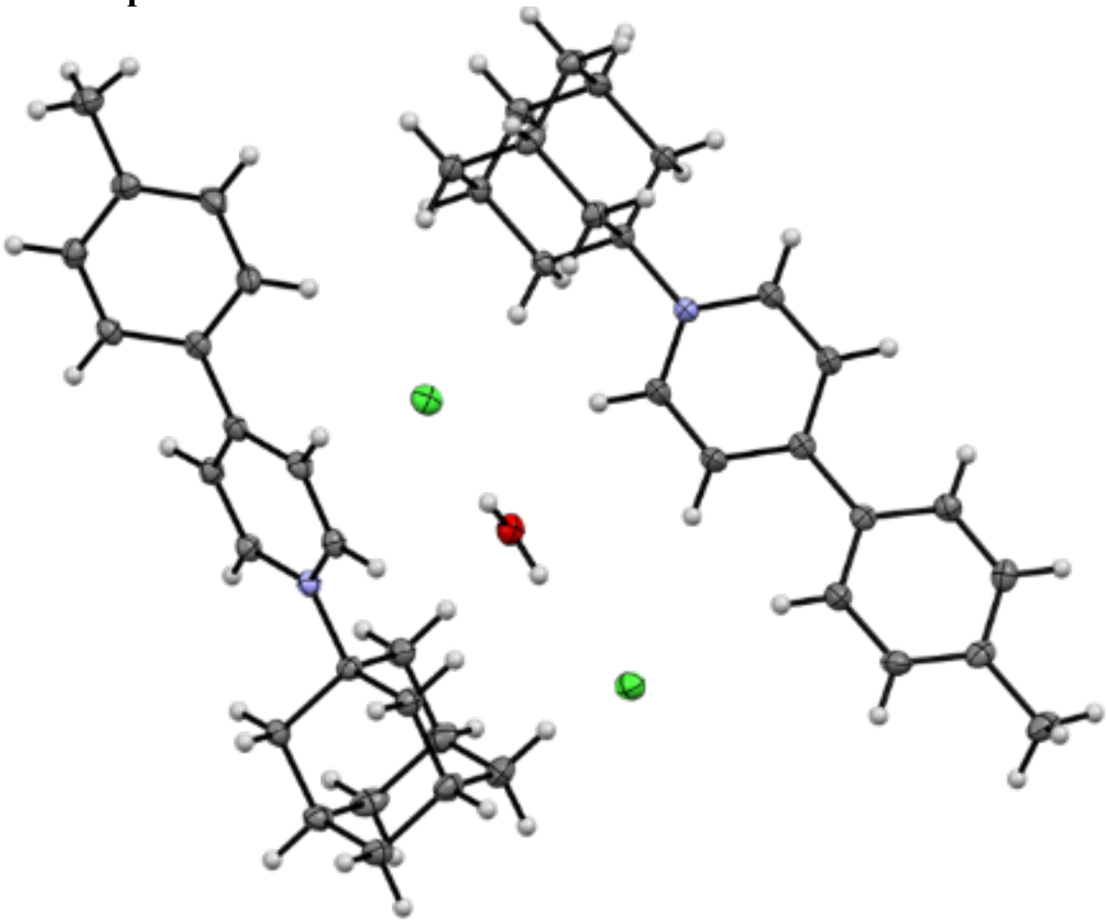

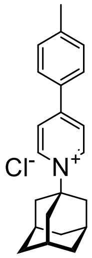

Figure S111. ORTEP visualization of **1**.

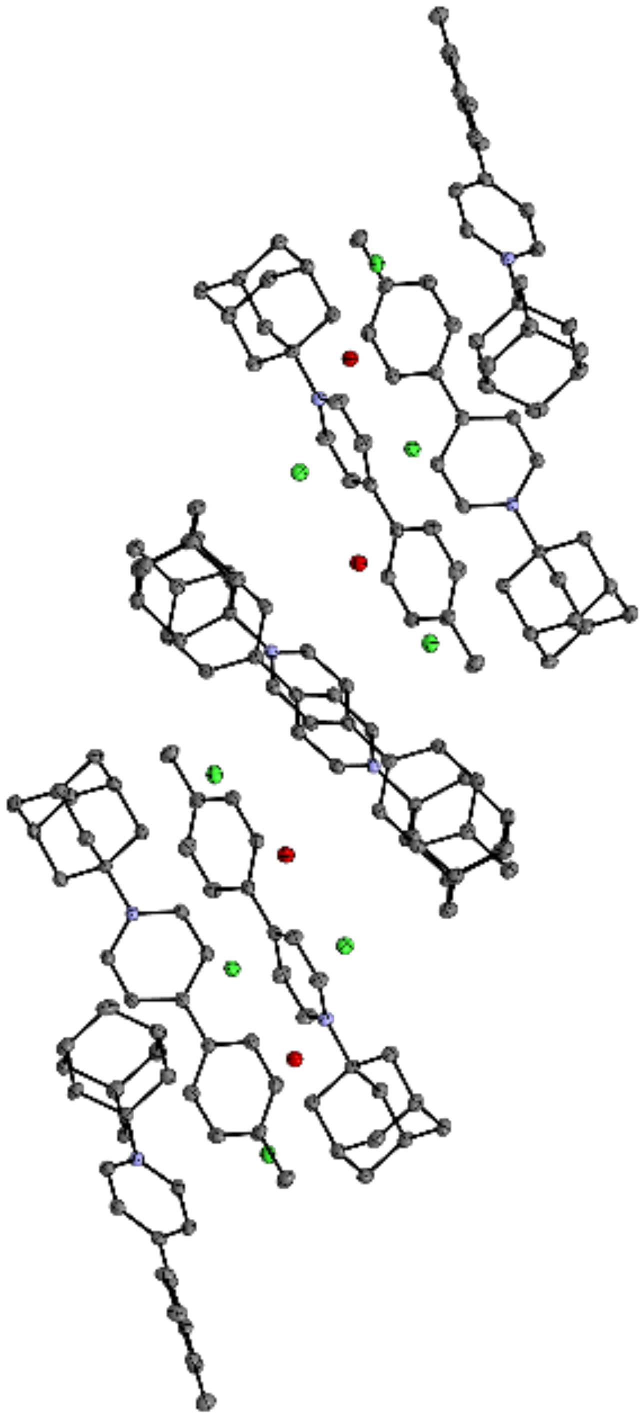

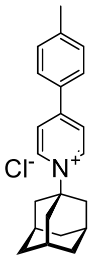

Figure S112. Crystal packing of **1**. Hydrogen atoms were omitted for clarity.

7.2. Compound 2

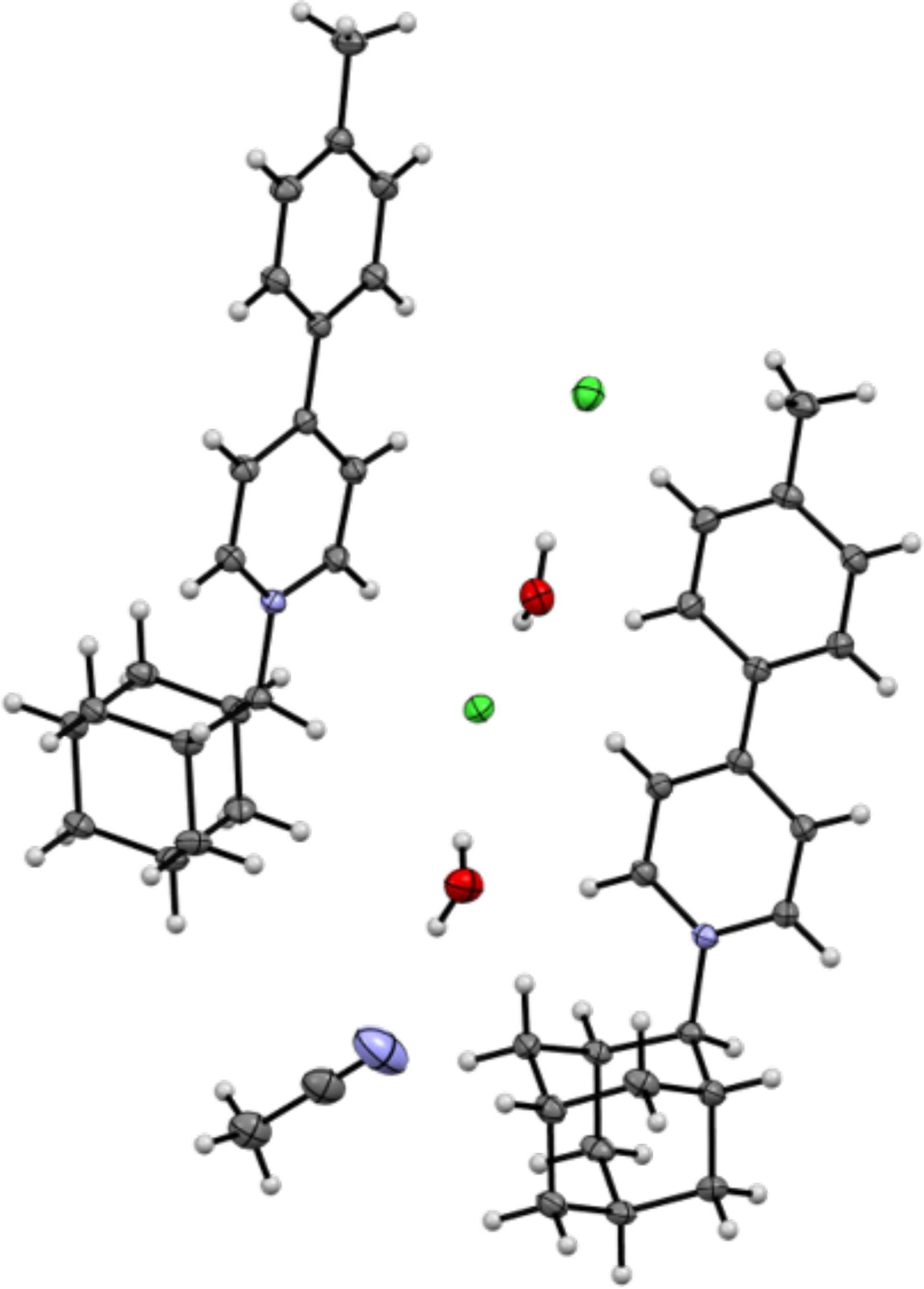

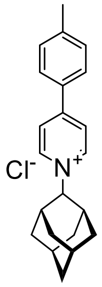

Figure S113. ORTEP visualization of **2**.

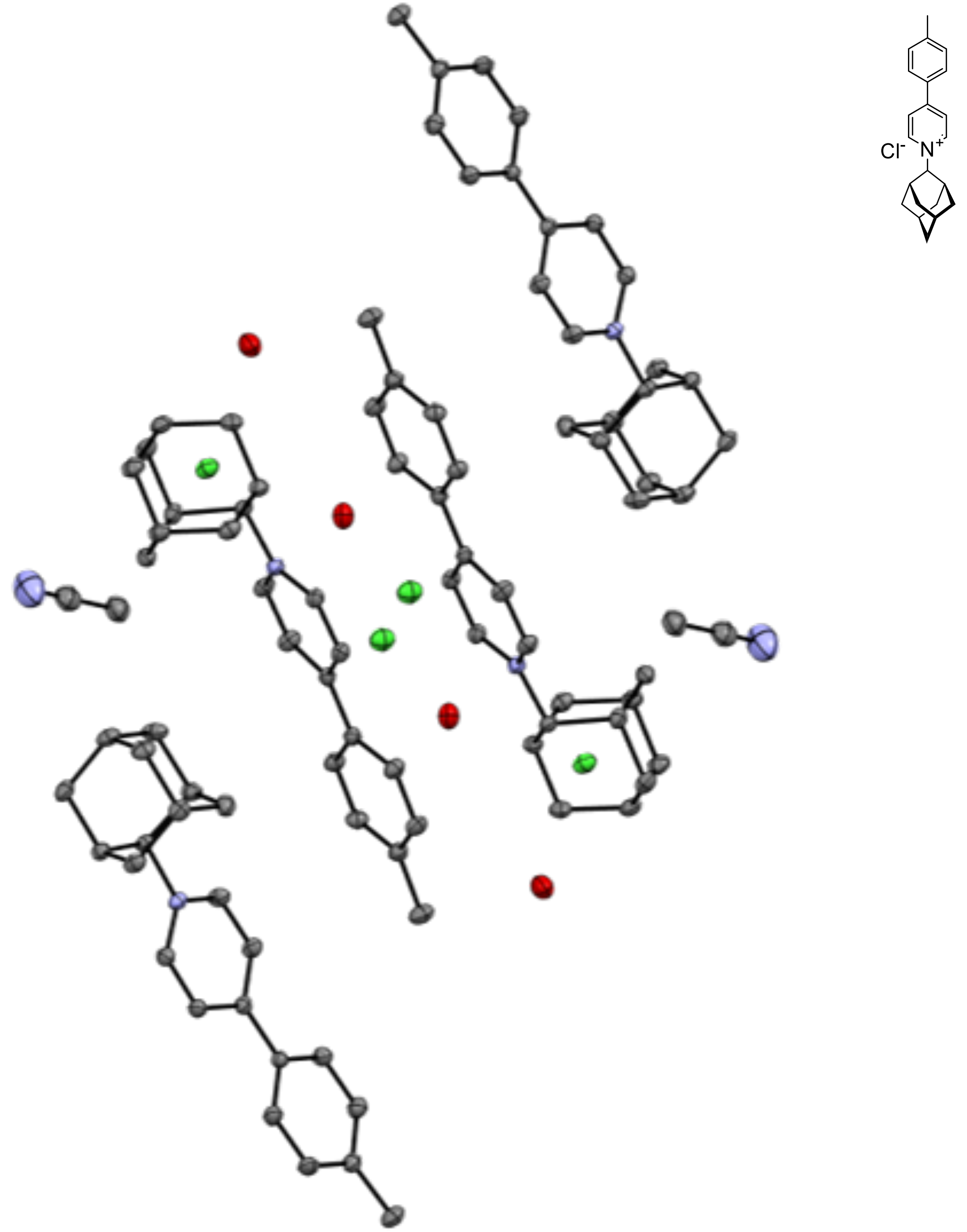

Figure S114. Crystal packing of 2. Hydrogen atoms were omitted for clarity.

7.3. Compound 3

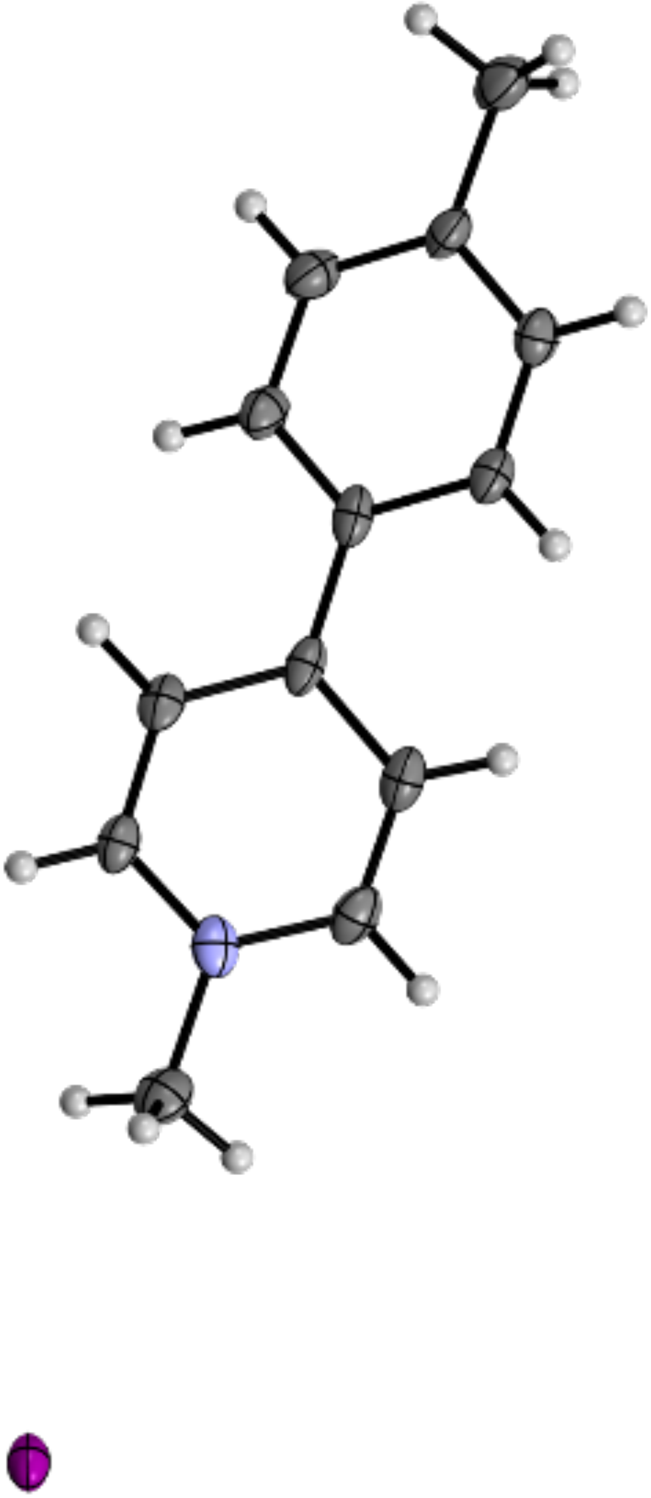

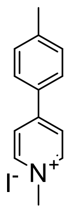

Figure S115. ORTEP visualization of **3**.

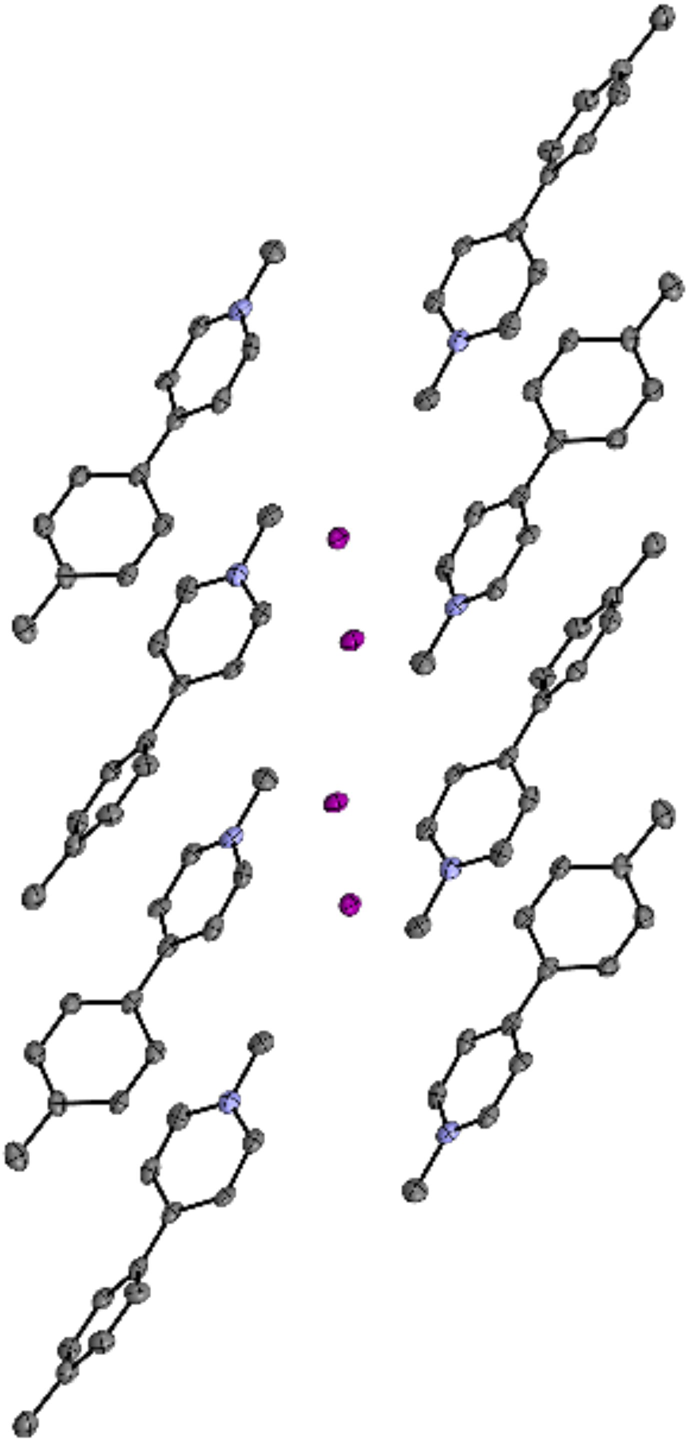

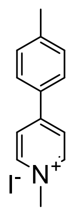

Figure S116. Crystal packing of **3**. Hydrogen atoms were omitted for clarity.

7.3. Compound 6

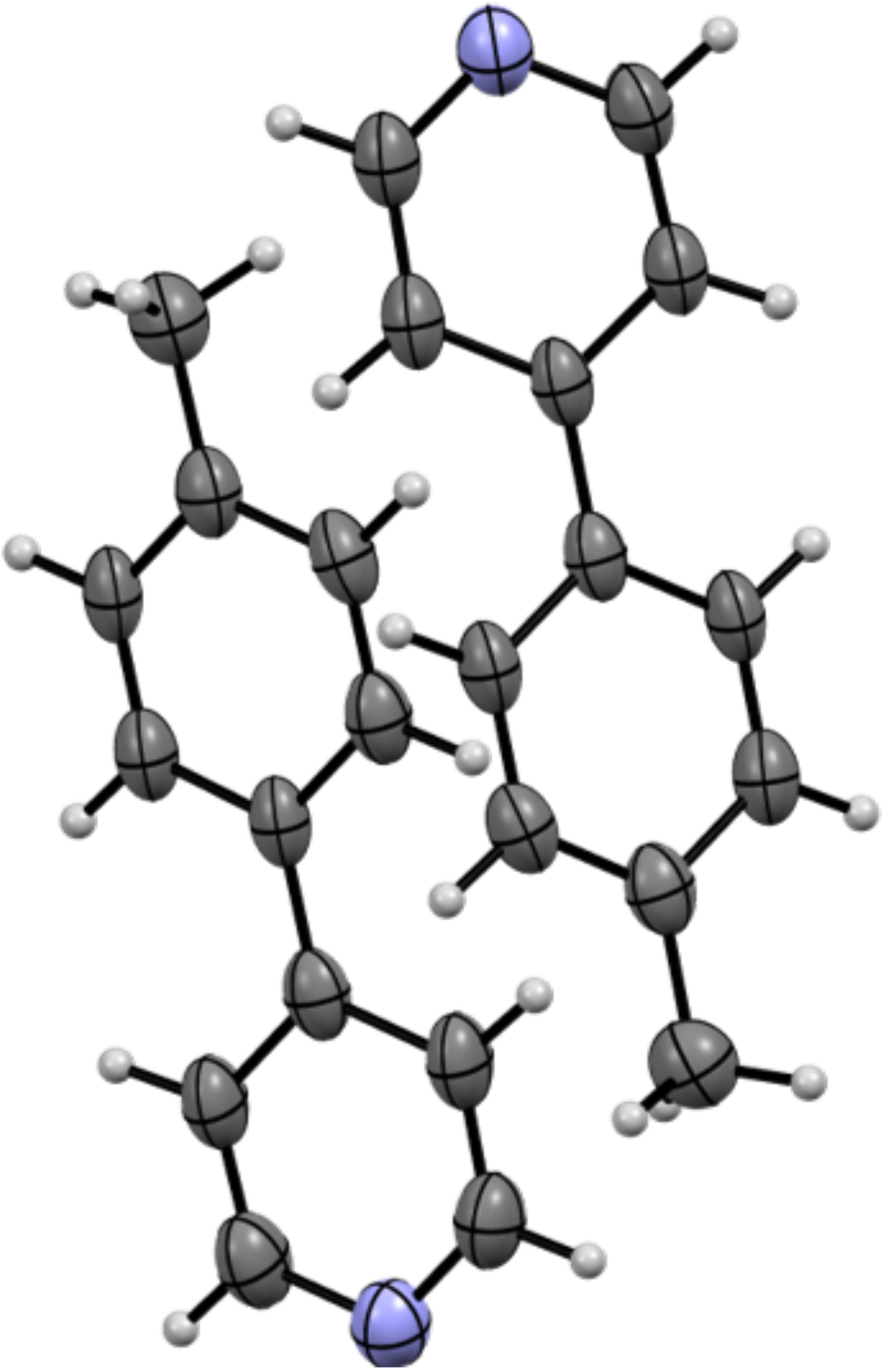

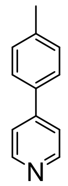

Figure S117. ORTEP visualization of **7**.

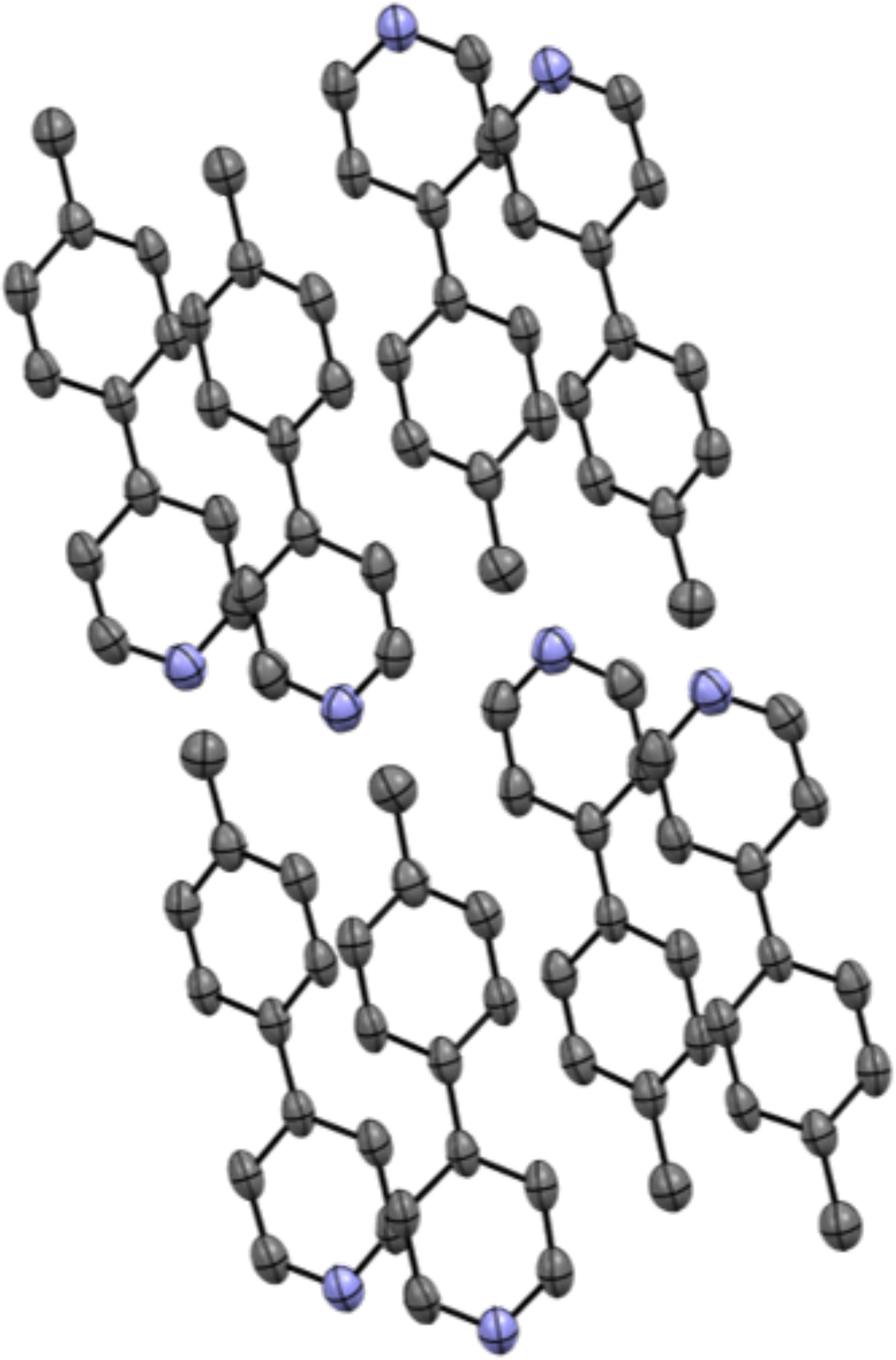

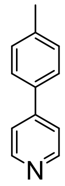

Figure S118. Crystal packing of **7**. Hydrogen atoms were omitted for clarity.

8. References

(1) *Modern Supramolecular Chemistry: Strategies for Macrocycle Synthesis*; Diederich, F., Stang, P. J., Tykwinski, R. R., Eds.; WILEY-VCH: Weinheim, 2008.

(2) Kaleta, J.; Tarábek, J.; Akdag, A.; Pohl. R.; Michl, J. The 16 $CB_{11}(CH_3)_n(CD_3)_{12-n}^{\bullet}$ Radicals with 5-Fold Substitution Symmetry: Spin Density Distribution in $CB_{11}Me_{12}^{\bullet}$. *Inorg. Chem.* **2012**, *51*, 10819-10824.

(3) Kaleta, J.; Akdag, A.; Crespo, R.; Piqueras, M.-C.; Michl, J. Evidence for an Intermediate in the Methylation of $CB_{11}H_{12}^{-}$ with Methyl Triflate: Comparison of Electrophilic Substitution in Cage Boranes and in Arenes. *ChemPlusChem* **2013**, *78*, 1174-1183.

(4) Armarego, W. L. F.; Chai, C. L. L. *Purification of Laboratory Chemicals*, 7th ed.; Elsevier/Butterworth-Heinemann: Amsterdam : London, 2013.

(5) dos Santos Pereira, A. K.; Nakahata, D. H.; Manzano, C. M.; de Alencar Simoni, D.; Pereira, D. H.; Lustri, W. R.; Formiga, A. L. B.; Corbi, P. P. Synthesis, Crystallographic Studies, Molecular Modeling and in Vitro Biological Studies of Silver(I) Complexes with Aminoadamantane Ligands. *Polyhedron* **2019**, *173*, 114116. https://doi.org/10.1016/j.poly.2019.114116.

(6) Casalnuovo, A. L.; Calabrese, J. C. Palladium-Catalyzed Alkylations in Aqueous Media. *J. Am. Chem. Soc.* **1990**, *112* (11), 4324–4330. https://doi.org/10.1021/ja00167a032.

(7) Bissel, P.; Geherin, S.; Igarashi, K.; Gandour, R. D.; Lazar, I. M.; Castagnoli Jr, N. Mass Spectrometric Studies on 4-Aryl-1-Cyclopropyl-1,2-Dihydropyridinyl Derivatives: An Examination of a Novel Fragmentation Pathway. *J. Mass Spectrom.* **2006**, *41* (12), 1643–1653. https://doi.org/10.1002/jms.1135.

(8) Wimalasena, D. S.; Perera, R. P.; Heyen, B. J.; Balasooriya, I. S.; Wimalasena, K. Vesicular Monoamine Transporter Substrate/Inhibitor Activity of MPTP/MPP $^{+}$ Derivatives: A Structure–Activity Study. *J. Med. Chem.* **2008**, *51* (4), 760–768. https://doi.org/10.1021/jm070875p.

(9) Yamazaki, S.; 進山崎; Torii, M.; 鳥居真由美; Tanaka, Y.; 田中洋輔. Onium Salt Having Trialkoxysilylalkyl Group. JP2010248165A, November 4, 2010. https://patents.google.com/patent/JP2010248165A/en (accessed 2023-02-10).
